# Pseudogenization of the MCP-2/*CCL8* chemokine gene in European rabbit (genus *Oryctolagus*), but not in species of Cottontail rabbit (*Sylvilagus*) and Hare (*Lepus*)

**DOI:** 10.1186/1471-2156-13-72

**Published:** 2012-08-15

**Authors:** Wessel van der Loo, Sandra Afonso, Ana Lemos de Matos, Joana Abrantes, Pedro J Esteves

**Affiliations:** 1 CIBIO-UP, Centro de Investigação em Biodiversidade e Recursos Genéticos, Campus Agrário de Vairão, Universidade do Porto, Vairão, 4485-661, Portugal; 2 Departamento de Zoologia e Antropologia, Faculdade de Ciências, Universidade do Porto, Porto, 4169-007, Portugal; 3 Department of Microbiology and Immunology, Stritch School of Medicine, Loyola University Chicago, Maywood, IL, 60153, USA; 4 INSERM U892 - Institut de Biologie Equipe 5, Nantes, Cedex 01, 44007, France; 5 Centro de Investigação em Tecnologias da Saúde, IPSN, CESPU, Gandra, 4585-116, Portugal

**Keywords:** Chemokines, Monocyte chemotactic protein, Pseudogene, Poxvirus, Myxomatosis, Oryctolagus, Bunolagus, Sylvilagus, Lepus

## Abstract

**Background:**

Recent studies in human have highlighted the importance of the monocyte chemotactic proteins (MCP) in leukocyte trafficking and their effects in inflammatory processes, tumor progression, and HIV-1 infection. In European rabbit (*Oryctolagus cuniculus*) one of the prime MCP targets, the chemokine receptor CCR5 underwent a unique structural alteration. Until now, no homologue of MCP-2/*CCL8*^a^, MCP-3/*CCL7* or MCP-4/*CCL13* genes have been reported for this species. This is interesting, because at least the first two genes are expressed in most, if not all, mammals studied, and appear to be implicated in a variety of important chemokine ligand-receptor interactions. By assessing the Rabbit Whole Genome Sequence (WGS) data we have searched for orthologs of the mammalian genes of the MCP-Eotaxin cluster.

**Results:**

We have localized the orthologs of these chemokine genes in the genome of European rabbit and compared them to those of leporid genera which do (*i.e*. *Oryctolagus* and *Bunolagus*) or do not share the CCR5 alteration with European rabbit (*i.e. Lepus* and *Sylvilagus*). Of the Rabbit orthologs of the *CCL8*, *CCL7*, and *CCL13* genes only the last two were potentially functional, although showing some structural anomalies at the protein level. The ortholog of MCP-2/*CCL8* appeared to be pseudogenized by deleterious nucleotide substitutions affecting exon1 and exon2. By analyzing both genomic and cDNA products, these studies were extended to wild specimens of four genera of the *Leporidae* family: *Oryctolagus*, *Bunolagus, Lepus,* and *Sylvilagus*. It appeared that the anomalies of the MCP-3/*CCL7* and MCP-4/*CCL13* proteins are shared among the different species of leporids. In contrast, whereas MCP-2/*CCL8* was pseudogenized in every studied specimen of the *Oryctolagus* - *Bunolagus* lineage, this gene was intact in species of the *Lepus* - *Sylvilagus* lineage, and was, at least in *Lepus*, correctly transcribed.

**Conclusion:**

The biological function of a gene was often revealed in situations of dysfunction or gene loss. Infections with Myxoma virus (MYXV) tend to be fatal in European rabbit (genus *Oryctolagus*), while being harmless in Hares (genus *Lepus*) and benign in Cottontail rabbit (genus *Sylvilagus*), the natural hosts of the virus. This communication should stimulate research on a possible role of MCP-2/*CCL8* in poxvirus related pathogenicity.

## Background

The Lagomorph family of *Leporidae* (leporids) originated in the New World (Neoartics/Americas), an area which still is home to the most successful of living leporids *i.e.* species of *Sylvilagus* and *Lepus*. The genera *Sylvilagus* (cottontail rabbits) and *Lepus* (jack rabbits or hares) comprise numerous species, with *Lepus* having conquered also the Old World (Paleoartics/Afro-Eurasia) [1]. In contrast, typical Old World leporid genera tend to be monotypic, inhabiting isolated areas where many of them are listed as endangered [2]. The recent world-wide success of the European rabbit (*Oryctolagus cuniculus*), which in prehistoric times was confined to the Southwestern parts of the Iberian Peninsula, was largely, if not entirely, due to human activity [3-6].

The introduction of Myxoma virus (MYXV) during the midst of last century as a method of rabbit pest control had devastating effects on populations of European rabbit with reported mortality rates approaching 100% in Europe and Australia [7]. This was in sharp contrast to the very mild pathology caused by the virus in its natural host and reservoir, *i.e.* species of the genus *Sylvilagus*[8,9]. For *Lepus* species, only few cases of MYXV infections were reported and experiments in France have shown that most individuals are innately resistant, reviewed in [7]. In nature, infection with MYXV occurs through bites by flying or jumping insects. Replication of virus starts in MHC-II positive dendritic-like cells at the bite lesions and is passed on to T cells of lymph nodes draining the inoculation site [10]. The pathogenesis of MYXV infection apparently depends upon the aptitude of avoiding the spreading of infected cells throughout the lymphatic system. Whereas in cottontail rabbits MYXV infection remains localized, in ‘naïve’ European rabbits (below “Rabbit”)^b^, the MYXV infected cells rapidly spread to distal nodes. This results in a generalized leukocyte depletion, particular of CD4+ T cells, which leads to a systemic immunodepression with fatal outcome *i.e.* myxomatosis [11,12].

Leukocyte migration and trafficking are mainly governed through interactions of a variety of chemokines with their cellular receptors [13,14]. Insights in the parasite strategies of immune evasion offer major gateways for identifying genetic components of pathways allowing Cottontail rabbit to cope with MYXV infection. Studies of different research teams have shown that this virus encodes a number of proteins that manipulate factors of the innate immune system of the host, among them proteins interfering directly with chemo-attractive functions of the CC chemokines [15,16]. It shows that these proteins have played a role during the process of coadaptation between virus and host, and most likely still do. These findings have been of cardinal guidance in the search for host genes (candidate genes) that could make the difference between susceptible vs. resistant species.

MYXV is a large double-stranded DNA virus of the poxvirus family (genus *Leporipoxvirus*). There have been indications that the CCR5 receptor might play a crucial role during MYXV infection, as it is the case by HIV infection in human [17,18], although the experimental evidence for this has been disputed [19]. However as already mentioned, the variation of pathogenicity of the MYXV among leporid species does not depend upon the fact whether or not the virus can enter and replicate in the host cell, but more likely on a constellation of endogenous factors preventing or permitting the dissemination of infected cells throughout the lymphatic system [11,12]. Studies of pathways underlying the contrasting outcomes of MYXV infection may therefore contribute to a more general understanding of pathogenesis due to large DNA viruses in mammals, inclusive humans. In view of the importance of CCR2 and CCR5 receptors in HIV infection, genes controlling these receptors and their ligands might be prefigurative of such ‘candidate genes’. This led to the discovery of a gene conversion that altered the second external loop of Rabbit CCR5. This mutation occurred in the ancestral lineage of the Old World genera including *Oryctolagus* and *Bunolagus*, but not in the lineages of *Sylvilagus* and *Lepus* species [20,21]. Although these differences at CCR5 obviously do *not* arbitrate the entry of MYXV for lymphocytes, they might affect CCR5 related pathways of signal transduction [17-19]. Note that *Bunolagus* species being highly endangered, studying their susceptibility to myxomatosis proved impracticable [2].

We therefore have taken a closer look at the main ligands of the Rabbit CCR2 and CCR5 receptors which are the ‘*macrophage inflammatory proteins*’ chemokines (MIP’s) and the *‘monocyte chemotacticproteins’* (MCP’s). The excellent recent review of the gene organization of mammalian chemokines by Nomiyama and coworkers [22], while comprehensive by extending to non-eutherian mammals (*Metatheria* and *Monotremata*), did not include *Lagomorpha* (Rabbits and Hares). Indeed, chemokine data on Rabbit are incomplete and sometimes erratic (see below). The *Rabbit Genome Project* being recently completed at the Broad Institute at 7x coverage [23], we have assessed the Rabbit Whole Genome Sequence (WGS) data for orthologs of the mammalian genes of MIP-RANTES and MCP-Eotaxin. Our analyses based on nucleotide sequence similarity revealed that Rabbit possesses proper orthologs of three MCP encoding genes (*CCL7*, *CCL8*, and *CCL13*) which are not identified by gene finder methods used by GenBank. The non-annotation can indicate that in Rabbit these genes may have acquired singularities hampering transcription or disqualifying them as functional proteins. We have searched for such traits and, at the event, verified their presence or absence in species of the leporid genera *Oryctolagus, Bunolagus, Sylvilagus* and *Lepus*.

## Results

The genes of the CC chemokine ligands (CCL) RANTES/*CCL5*, MIP-1a/*CCL3*, and MIP-1b/*CCL4* are documented for Rabbit (*Oryctolagus cuniculus*) [GenBank: NC_013687_REGION:24922000.25085000]. They are located on chromosome 19 as a syntenic group [GenBank: NC_000017_REGION:34198000to34433100] and are in every respect (chromosomal location, gene organization, sequence similarity) orthologs of their mammalian counterparts *cf*. [22]. In contrast, the GenBank list of Rabbit orthologs of the mammalian MCP-Eotaxin encoding genes is limited to MCP-1/*CCL2* and Eotaxin/*CCL11* [Genbank: NC_013687 REGION:23720000.23798000].True orthologs of mammalian MCP-3/*CCL7*, MCP-2/*CCL8*, and MCP-4/*CCL13* have not yet been identified (a print-out of the GenBank Features report is shown in Additional file 1). This is surprising because at least the first two chemokines seem to be functional in most, if not all mammal species studied [22], and in Human and Mouse are subject of intense investigations due to their importance in regulating inflammatory and anti-tumoral effects and for their role in HIV infection [24-28].

We will adopt the *CCL* nomenclature unless we are dealing with proteins. For most mammals, MCP encoding genes are organized as a syntenic group composed of the chemokine genes *CCL2CCL7CCL11CCL8CCL13CCL1*[22] (in that order; *CCL1* serves here only as a syntenic marker). In Human the *CCL2CCL1* encompassing syntenic group is located on chromosome 17 [GenBank: NC_000017.1_REGION:32582070.32690817]. The fragment of Rabbit chromosome 19 is homologous to this region [Genbank: NC_013687_REGION:23720000.23798000]. We will refer to it as “R-MCPgb”.

The pronounced interspecies similarity between the coding sequences allowed localizing the orthologs of the *CCL2*, *-7*, *-11*, *-8*, and *-13* genes in the Rabbit genome. The exons positions are listed side by side for Rabbit and Human in Table 1. The sequence alignments can be consulted in Additional files, with annotations for the undocumented genes (*CCL7*: Additional file 2, *CCL8*: Additional file 3, *CCL13*: Additional file 4) or for the entire MCPgb regions as a ‘blunt' 108 kb alignment in FASTA format (Additional file 5).

**Table 1:** **Identification of gene structure of Rabbit*CCL2,-7,-11,-8,-13,-1*genes based on Human orthologs**

***CHEMOKINE***			**P**	**GenB**			**sense**
**Species**	**orcu**^$^	**orcu**^$^			Hosa^*^	Hosa^*^	
***CCL2***							
hnmRNA	216	2031			227	2153	
CDS-ex1	287	362	0.86	v	300	375	+
CDS-ex2	1066	1183	1.00	v	1172	1289	+
CDS-ex3	1534	1717	0.89	v	1627	1777	+
***CCL7***							
hnmRNA	11534	13840			15171	17187	
CDS-ex1	11603	11678	0.99	v	15241	15316	+
CDS-ex2	12913	13030	1.00	-	16096	16213	+
CDS-ex3	13429	13534	1.00	-	16647	16752	+
***CCL11***							
hnmRNA	19375	22120			30618	33130	
CDS-ex1	19509	19584	0.89	- v	30759	30834	+
CDS-ex2	20714	20822	1.00	v v	32046	32157	+
CDS-ex3	21217	21322	1.00	v v	32535	32640	+
***CCL8-like***							
pseudogene	47126	49449			63997	66352	
CDS-ex1	47576	47641		-	64452	64527	+
CDS-ex2	48337	48455		-	65219	65336	+
CDS-ex3	48872	48977		-	65752	65857	+
***CCL13***							
hnmRNA	71995	73927			101402	103560	
CDS-ex1	72083	72158	1.00	-	101477	101552	+
CDS-ex2	72902	73016	1.00	-	102425	102539	+
CDS-ex3	73361	73457	1.00	-	102976	103081	+
***complCCL1***							
hnmRNA	74930	77383			105330	108183	-
CDS-ex3	75053	75155	1.00	-	105509	105611	-
CDS-ex2	76180	76291	1.00	-	106735	106846	-
CDS-ex1	77337	77412	0.91	-	108036	108111	-

^$)^: pos 1 corresponds to position 23720000 of NC_013687.1.

^*)^: pos 1 corresponds to position 32582070 of NC_000017.1.

The Rabbit genome fragment R-MCPgb containing the *CCL2* and *CCL1* genes is compared to its Human correlate according to the alignment in Additional file 5. The identification of the Rabbit orthologs of the Human *CCL* genes based on sequence similarity was checked for consistency using Genscan [29], which furthermore provided estimates of the quality of intron splice sites (values varying between 0 and 1 are shown under the heading “P”). Positions of transcription initiator and termination sites were estimated by homology with Human. The exons annotated in the GenBank feature file of the Rabbit sequence (Additional file 1) are marked “v” under “GenB”.

### Orthologs of *CCL7* and *CCL13* exist and are similar among leporid species

Of the two isoforms of the “Rabbit A11 chemokine” annotated in the GenBank, “isoform 2” is orthologous to mammalian *CCL11*, whereas the “isoform 1” differs from *CCL11* only by using as initiating exon a sequence located upstream between *CCL2* and *CCL11*. Sequence alignments make it clear that this exon is orthologous to the initiating exon of mammalian *CCL7* and at the same time reveal potential exons that are orthologs of the mammalian *CCL7* exons 2 and 3. The mRNA transcripts of both *CCL11* isoforms are also reported as ‘transcriptional variants 2’ [GenBank: XM_002719227.1] and ‘transcriptional variant 1’ [GenBank: XM_002719226.1].

**Figure 1:**
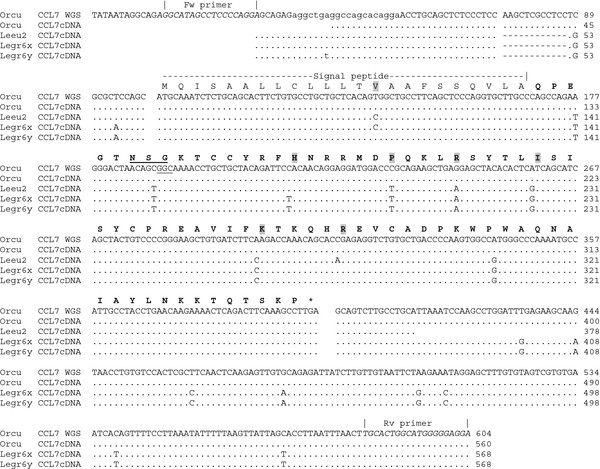
**The rabbit ortholog of human *CCL7 *gene exists and is transcribed.** The “A11 isoform 1” predicted by GenBank suggests that exon1 of the Rabbit ortholog of *CCL7* uses preferentially exon2 and exon3 of *CCL11* during transcription (Additional file 2). We show that cDNA of the leporid species (*Oryctolagus cuniculus, Lepus granatensis* and *Lepus europaeus*) contain a transcript uniting three exons orthologous to those of human *CCL7* gene. The position of missing MCP-3 characteristic N-glycosylation site is underlined (N X S in other mammals). Variable amino acid residues are highlighted in gray.

**Figure 2:**
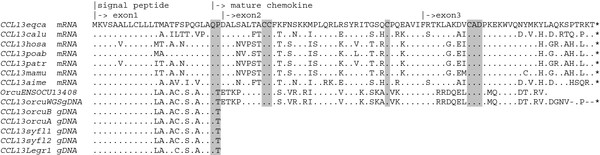
**Lacking of N-terminal X-Pro motifs in Leporid MCP **-**4/*CCL13*.** All known MCP genes encode a 24GlnPro25 (QP) motif which is required for post-transcriptional maturation and modification of the protein. The Rabbit *CCL13* ortholog is unique by encoding a 24GlnThr25 motif (QT). This particularity appears to be shared among leporids, inclusive *Sylvilagus*. Leporid genomic DNA was amplified using *CCL13* primers designed on R-MCPgb. *orcuB*: Rabbit, subsp. *cuniculus*, *orcuA*: Rabbit, subsp. *algirus*, syfl: Western Cottontail Rabbit, *Legr*: Granada Hare, *Orcu ENS13408*: [Ensembl: ENSOCUT00000013408]. The MCP-4 protein sequences of “other” mammals are derived from the *CCL13* mRNA sequences listed in Additional file 9. *eqca*: horse, *calu*: dog, *hosa*: human, *poab*: orangutan, *mamu*: monkey, *aime*: giant panda.

We have evaluated the putative functionality of the *CCL7*, *-11*, and *-13* genes, by submitting the R-MCP fragment to Gene Finder software, which did report three exons for each of five genes (*CCL2*, *-7*, *-11*, *-13*, and -1). All exons were localized exactly as previously inferred by sequence similarity (Table 1). The predicted genes were confirmed by testing specimens of *Oryctolagus* and *Lepus* for correct transcription of the *CCL7* gene by PCR amplification of cDNA. The *CCL7* gene appeared to be transcribed as predicted in Table 1 (Figure 1). Although they are not identified as such, Ensembl.org reports a Rabbit sequence [Ensembl: ENSOCUG00000013412] and its translation [Ensembl: ENSOCUT00000013408] which correspond to transcripts of the *CCL13* ortholog as defined in Table 1 (Figure 2; for detailed descriptions see Additional file 6). The fact this sequence was derived from cDNA implies that also the *CCL13* gene is transcribed. We note the minor sequencing differences at the 3’ end of the sequence which might have to do with proximity of the reverse primer used for cDNA amplification.

Transcription alone does not necessarily warrant a functional gene product. The proteins deduced from Rabbit *CCL7*/MCP-3 and *CCL13*/MCP-4 CDS sequences show indeed structural anomalies which could disqualify them as functional MCP chemokines. Rabbit MCP-3/*CCL7* misses an N-glycosylation site which is present in all known MCP-3 sequences [30]. As glycosylation of MCP-3 may affect its biological activity [31], the loss of the AsnXSer site (underlined in Figure 1) might impair normal chemokine function.

The situation is more problematic with MCP-4/*CCL13*. Mature MCP chemokines are derived from the precursor sequence after cyclization of the glutamine Gln24 residue, which is encoded by the 3’ end of exon1. In reports on the biological activity of MCP’s, the resulting N-terminal pyroglutamic acid (pGlu) is therefore referred to as pGlu1 rather than Gln24. Indeed, most reported MCP protein precursors (*CCL2*, -7, -8, and -13) are characterized by a 24GlnPro25 motif which after cyclization can further be modified by different types of metalloproteinases [32-34]. At the same time, pGlu blocks the action of serine protease peptidases, which in non-MCP chemokines recognize the ubiquitous Pro residue at position 2 of the NH_2_-terminus of the mature protein, by this way fine tuning their function [24,35]. The MCP-4/*CCL13* precursor protein inferred from the R-MCPgb sequence is particular by showing a 24Gln*Thr*25 motif instead of the canonical 24Gln*Pro*25. Whereas a Gln24 residue is not a prerequisite of chemokine maturation (e.g. in Rodent MCP-2/*CCL8* and Human Eotaxin/*CCL11* it is replaced by Gly), the absence of a 24X-Pro25 motif is liable to prevent normal posttranslational processing.

However, we found by PCR of gDNA that these singularities encoded by the Rabbit *CCL7* and *CCL13* genes are shared among the different leporid genera studied, including *Sylvilagus* (Figures 1 and 2), making a contribution to species-specific variation in disease resistance highly unlikely.

**Figure 3:**
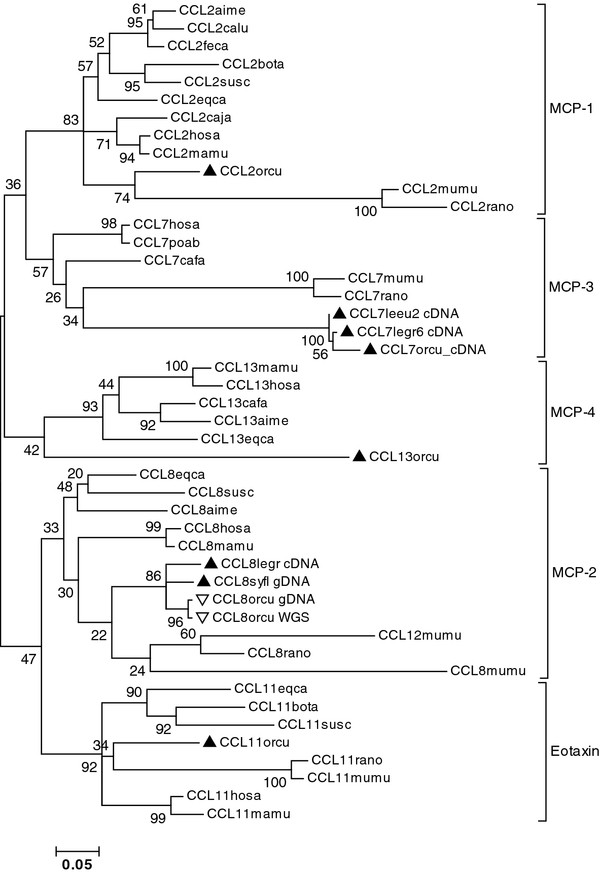
**Gene orthology of *CCL2 *,*-7 *, **-***11 *, **-***8 *, and **-***13 *of placental mammals supported by phylogeny.** The evolutionary history was inferred by using the Maximum Likelihood method conducted in MEGA5 [36]. The tree with the highest log likelihood (-4585.9908) is shown. Bootstrap values are placed next to the branches. Codon positions included were 1^st^ + 2^nd^ + 3^rd^ and were limited to those encoding the mature chemokine. There were 231 positions in the final data set. Positions of leporid branches are highlighted by triangles ▴ for functional, or by ∇ for pseudogenes. Taxon names of sequences obtained in this study are extended by ‘gDNA’ when derived from genomic DNA or by ‘cDNA’ when (also) derived from cDNA. All other sequences represent a sample of mRNA sequences listed in Additional file 9. Mouse *CCL12* and *CCL8* are located between the *CCL11* and *CCL1* genes and are likely duplicates of the ortholog of murid *CCL8*[22].

Interestingly, the two Ensembl ENSOCU sequences mentioned were either designated as RABIT CCL8 or as Rabbit CCL7 (Additional file 6). The confusion about identifying the Rabbit *CCL13* and *CCL7* orthologs is probably due to the relatively large protein distances separating them from their mammalian correlates (Figure 2). Different methods of phylogenetic reconstruction produced nevertheless trees in which the paralogous genes did cluster according to orthology, inclusive the Rabbit genes (Figure 3). Bootstrap values were however very low. Incidentally, the branch lengths of Rabbit *CCL7* and *-13* nodes were about two times larger in comparison to the average branch length of the Rabbit *CCL2*, *-8*, and *-11* nodes. Note that Leporid *CCL8* is relatively well conserved (Figure 3).

We conclude that Leporid orthologs of *CCL7* and *CCL13* exist and are transcribed but that their contribution to species-specific disease resistance is unlikely.

### The Rabbit ortholog of *CCL8* exists but is pseudogenized

**Figure 4:**
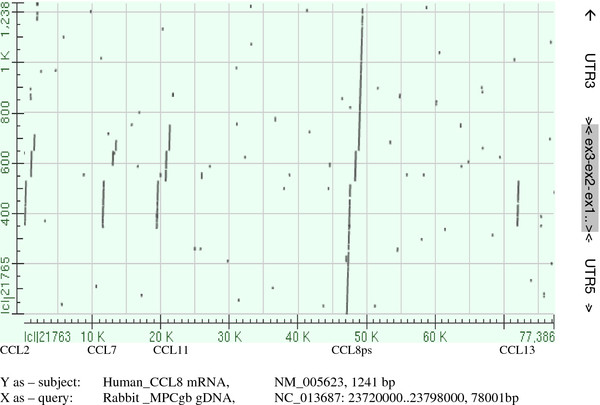
**Fishing the Rabbit *CCL8 *ortholog using NCBI Blast**-**Align.** Human *CCL8* mRNA is compared to the Rabbit genomic fragment containing both the *CCL2* and the *CCL1* region (R-MCPgb). The graphical representation visualizes the outstanding similarity of one genomic region covering the human RNA transcript almost entirely. At the same time it localizes other structurally related regions and reveals that compared to the coding regions, untranslated region (UTR’s) might be more gene-specific. The graph was produced using the online facilities offered by NCBI (http://blast.ncbi.nlm.nih.gov/Blast.cgi; option: *Align*).

Sequence alignments of mammalian *CCL8* mRNA (at least for swine, horse, cow, panda, human and dolphin) identify clearly a single region of outstanding *CCL8* homology within R-MCPgb. It is located between the *CCL11* and *CCL13* genes, as is the case for *CCL8* in most, if not all mammals for which this has been checked [22]. It is interesting that gene orthology was much better revealed when the untranslated regions (UTR’s) were included. This is illustrated in Figure 4. Whereas the coding regions of *CCL8* show pronounced cross-paralog similarity with at least *CCL2*, *-7*, and *-11*, the UTR’s are highly gene specific.

**Figure 5:**
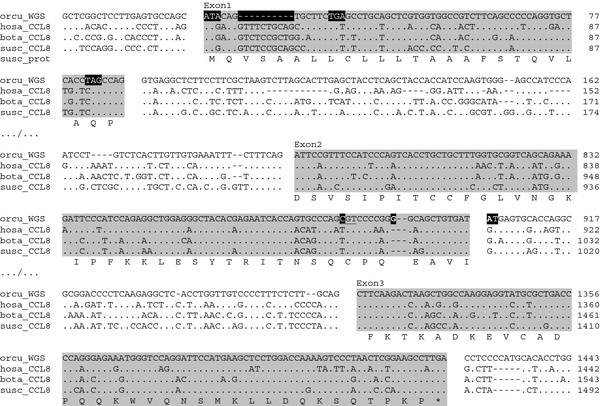
**The Rabbit ortholog of mammalian *CCL8 *exist as a pseudogene.** The “Orcu WGS” sequence shows parts of the exon embedding fragments of R-MCPgb region with outstanding similarity with mammalian MCP-2/*CCL8* gene (Figure 4). The alignment with functional mammalian *CCL8* clarifies the pseudogenization of Rabbit *CCL8* gene. Exon regions are highlighted in gray, disabling mutations are highlighted in black. orcu: *rabbit*, hosa: *human*, susc: *swine*. The protein sequence shown is inferred from the susc_*CCL8* sequence. Insertions were added at exon2 in order to maintain the reading frame. Open spaces are introduced at exon boundaries. ‘.’: identity with leader sequence, ‘-‘: deletions/insertions.

**Figure 6:**
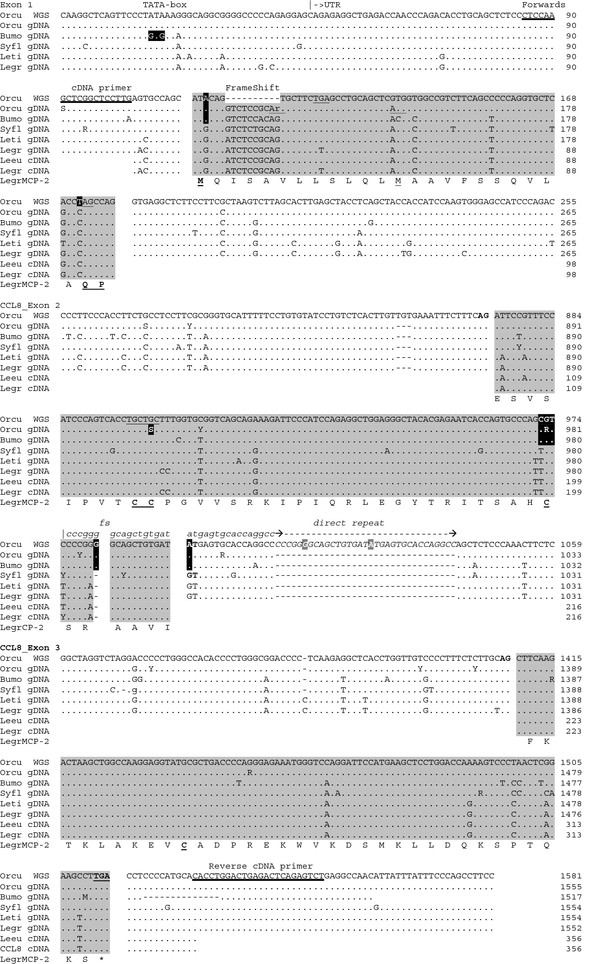
**Gene variation at the *CCL8 *locus within and among leporids.** PCR products of leporid specimens were obtained using either gDNA or cDNA and are aligned with the *CCL8ps* sequence of rabbit WGS. Potential Stop and Translation Initiating codons are underlined. The positions of the cDNA primers are bold underlined. ‘Orcu *CCL8ps*’ represents the consensus of 11 haplotypes of *Oryctolagus cuniculus* specimens of both subspecies (*O. c. cuniculus* and *O. c. algirus*), after excluding one haplotype that was identical to *CCL8ps* of WGS (OccTar109allele1) and ignoring singletons (i.e. nucleotide differences observed only once). Occ: *Oryctolagus cuniculus;* Bumo: *Bunolagus monticularis*; Syfl: *Sylvilagus floridanus;* Leti: *Lepus timidus*; Legr: *Lepus granatensis*; Leeu: *Lepus europaeus.* ‘.’: identical to leader sequence; ‘-‘: indel. The data shown are limited to the exon containing fractions. An alignment of the entire 1.6Kb gene region is presented in Additional file 7.

The comparison of the Rabbit *CCL8* sequence with its mammalian counterparts (Figure 5) reveals several deleterious mutations at exon1 and exon2, as well as at intron2. At exon1 the initiating methionine codon (ATG) was mutated into an isoleucine codon (ATA) and the CAG codon of the canonical GlnPro motif (CAG.CCA) at the 3’ end of the exon was changed into TAG. This premature stop codon is however put out of frame by a 10 base pair (bp) deletion, which incidentally transforms the in-frame LeuSer codons (CTG.AGC) into a premature stop codon (c.TGA.gc). The 3’ part of exon2 is corrupted by a 1 bp insert, while the GT donor site of intron2 is mutated into AT. In addition, the codon of the third of the four characteristic cysteine residues (TGT) was altered into arginine (CGT), or histidine (CAT) in some wild rabbits (Figure 6). At this position, a cysteine residue is mandatory for the formation of a disulfide bound with the first cysteine of the characteristic cysteine pair [37] and present in all CC chemokines.

A first question was in how far the WGS *CCL8* sequence is representative of the species, and if so, whether this situation is limited to the genus *Oryctolagus*. In order to assess the distribution and history of this apparent gene loss, we designed primers for each exon of the Rabbit *CCL8* ortholog (see Methods). By PCR we obtained the *CCL8* pseudogene (*CCL8ps*) DNA sequences of wild rabbits of both subspecies *Oryctolagus c. cuniculus* and *Oryctolagus c. algirus.* These rabbits were collected in the original distribution range of the genus (i.e. the Iberian Peninsula), where the gene diversity is much greater than in domestic and wild rabbits of the more recent areas of Rabbit colonization [38,39]. The PCR products confirmed that the *CCL8* ortholog is pseudogenized in all Rabbit genomes studied. Individual variation was observed. Only one rabbit showed a sequence identical with the *CCL8* sequence of R-MCPgb. It is interesting that for a majority of wild rabbits, the *CCL8* genes appeared as “less” derived compared to the Thorbecke rabbit of the WGS files (Figure 6). While all genomes studied showed the initiation site mutation ATG->ATA, many of them did not show the 10 bp deletion at exon1, and the vast majority did feature the canonic 24GlnPro25 codons instead of the in-frame stop codon of the WGS sequence. Moreover they disposed of one or even two Met(ATG) in-frame codons, which could possibly provide a rescue initiation site (Figure 6).

The situation is more clear-cut at exon2. The three deleterious mutations were present in all Rabbit haplotypes: (1) the altered third mandatory cysteine codon, (2) the 1 bp insert, and (3) the donor site alteration. On the other hand, at least two genomes showed the loss of the characteristic CysCys motif of exon2 (TGCTGC->TGCTCC), a deleterious mutation not present in the WGS Rabbit. The alignment with wild rabbit sequences furthermore revealed an interesting 33 bp insertion in the WGS sequences, which went unnoticed in previous alignments with non-leporid sequences (Figure 5). This insert was present in both subspecies, although not in all specimens. It results from an exact direct repeat at the junction exon2-intron2, spanning the 1 bp insert and the donor site mutation. Because both of these disabling mutations were present in all rabbit haplotypes, and repeated in the insert, the duplication is most likely more recent. This also applies to the 10 bp deletion at exon1 (*you can’t delete nor duplicate what didn’t already exist*). It indicates that the latter are consequences of a prior loss of functionality, and are part of a process of pseudogenization.

The terminating exon3 was found to be potentially “functional” in all rabbits studied.

### The Rabbit *CCL8* has been pseudogenized for more than 4 million years

These deleterious mutations are shared among both Rabbit subspecies *O. c. cuniculus* and *O. c. algirus.* These subspecies did separate about 2 My ago [40,41], implying that the pseudogenization of *CCL8* must be relatively old and possibly older than the genus. This was corroborated by the *CCL8* sequence obtained with one Riverine Rabbit, *Bunolagus monticularis*, which showed all four deleterious mutations shared among *Oryctolagus*. The pseudogenization must precede the genus split of *Bunolagus* and *Oryctolagus* which occurred an estimated 4 My ago [40,42].

We note in both haplotypes of the *Bunolagus* specimen the absence of the 10 bp deletion at the 5’ region of exon1 and of the 33 bp direct repeat at the 5’ region of intron2 of Rabbit *CCL8* (Figure 6). It further supports the viewpoint that the two occasional indels occurred after the initial pseudogenization. The *Bunolagus CCL8* pseudogene differs however from that of *Oryctolagus* by the absence of a theoretical ‘rescue’ initiator codon (ATG->ACG) in exon1, and the loss of the TATA box (TATAAA->TgTgAA), which *Oryctolagus* shares with many mammalian *CCL8* genes.

### MCP-2/*CCL8* appears to be functional in *Lepus* and *Sylvilagus* species

**Figure 7:**
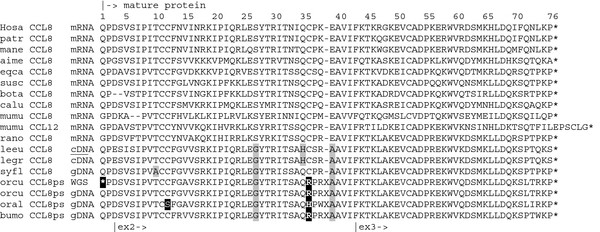
**Comparing mature MCP2 proteins of Leporid and Other-Mammals.** The consensual numbering of amino acid positions used in functional studies of MCP ligands is shown (e.g. [32]). Pseudogenes are translated according to a nucleotide alignment respecting the reading frame of functional homologs. Positions where leporid MCP-2 differs markedly from the mammalian consensus are highlighted in gray if inferred from ‘functional’ genes, or in black in case differences would be limited to pseudogenes. leeu: European brown hare, legr: Granada hare; syfl: Western cottontail rabbit; orcu: European rabbit, sps. *cuniculus*; oral: European rabbit, sps. *algirus*, bumo: South African Riverine rabbit. GenBank Accession of the underlying “Other-Mammal” sequences are listed in Additional file 9.

The next question was whether the *CCL8* gene is also pseudogenized in species of leporid lineages that do not share the CCR5 mutation with rabbit. As mentioned before, these species are also those that are resistant to MYXV. Using the primer sets designed for amplifying the three Rabbit-*CCL8*ps exons, we found that all haplotypes obtained with two *Lepus* specimens and three *Sylvilagus* specimens possess a *CCL8* ortholog which does *not* show any disabling mutations. The CDS consensus sequences are shown in alignment with Rabbit sequences (see Figure 6). The same alignment including the complete UTR’s and intron regions can be found in Additional file 7. The usual amino acid numbering of mature MCP-2 protein is shown in the alignment presented in Figure 7.

The functionality of the *CCL8* gene was further evaluated by analyzing cDNA obtained with specimens of species *Oryctolagus* and *Lepus*. Although the primers were designed according to the *Oryctolagus* sequence, the expected *CCL8* CDS sequences were obtained with both specimens of *Lepus* but not with cDNA of *Oryctolagus*. This failure was not due to the quality of the Rabbit cDNA, because successful PCR amplification of *CCL7* was obtained using the same cDNA preparation with the appropriate primers (Figure 1). Tissue samples of *Sylvilagus* specimens were not suited for RNA extraction, but the extensive sequence similarity with *Lepus* CCL8 predicts correct splicing and gene transcription, which was also confirmed by gene finder software applied to the genomic sequences of both genera.

### The Rabbit genome contains only one *CCL8*-like gene

A last question was whether functional *CCL8*-like genes might exist outside or within the R-MCPgb region studied. At least in mouse, cow and elephant the *CCL8* genes are indeed duplicated, which, parenthetically, might be a further indication of their relative importance (in mouse they are named *CCL1*2 and *CCL8*, although *CCL1*2 is more similar to mammalian *CCL8*; Figure 3). By blasting the (entire) Rabbit WGS database (inclusive the Trace File Archives) with the Rabbit-*CCL8* sequences here presented, at the exception of full identity with the actual query, highest similarities were obtained with sequences of the *CCL11* and *CCL7* genes embedded in the R-MCPgb fragment. It strongly suggests that there is no other *CCL8*-like gene in the entire genome of the specimen studied in the *Rabbit Genome Project.*

We can therefore ascertain that the unique ortholog of the mammalian *CCL8* gene is pseudogenized in the Old Word leporid genera *Oryctolagus* and *Bunolagus*, while potentially functional in *Sylvilagus* and *Lepus* species and, at least in *Lepus,* being correctly transcribed.

## Discussion

We show that both MCP-2/*CCL8* and CCR5 were altered in lineage of *Oryctolagus* and *Bunolagus* due to apomorphic mutations which did not occur in the lineage comprising species of *Sylvilagus* and *Lepus*. It implies that the species known to be reservoirs of MYXV, *i.e. S. brasiliensis* and *S. bachmani,* dispose most likely of normal (plesiomorphic) *CCR5* and *CCL8* genes. Although such deductions are in line with current phylogenetic inference, they might be worthwhile to be verified, as falsification would imply the independent alteration of one or both of these genes in lineages separated in space and time for millions of years. Meanwhile we will assume that the *CCR5* and *CCL8* genes of the different *Sylvilagus* species do not differ significantly from those shared by the New World rabbits of this study.

The situation revealed by the presented data might orientate research towards the role of chemokine and their receptors in host species of MYXV and could lead to new insights in processes of parasite-host coadaptation. In mice MCP-2/*CCL8* and CXCL12 were found to cooperate to attract hematopoietic progenitors of immune-regulatory dendritic cells [43] while Islam and coworkers [44] describe mouse MCP-2/*CCL8* as crucial regulator of T(h)2 cell homing. In Human, MCP-2 is known to bind to chemokine receptors CCR1, CCR2 and CCR5 and can act as a potent inhibitor of HIV-1 infectivity [44-46]. More in particular, compared to other chemokines, MCP-2 was found the most efficient inhibitor of the HIV protein gp120 for CCR5 receptor binding [47,48]. Studies of the role of MCP chemokines in Human and Mouse revealed that inflammation is regulated by feedback mechanisms where proteases play an important role [24,33-35]. They act by removing the N-terminal tetra or penta peptide of MCP’s which can transform them into MCP antagonists. Different research groups reported that natural occurring posttranslational modified MCP-2/*CCL8* products can completely (*sic*) block the chemotactic effects of intact MCP’s and of RANTES/*CCL5*, and have identified natural MCP-2(6*-7*6) (*cf*. Figure 7) as a potent and functional CC chemokine inhibitor [28,33,34]. These studies have highlighted the role played by MCP-2 products in the subtle agonist-antagonist interplay with CC chemokine receptors, including CCR5.

In this context our data might fuel speculations about possible reasons underlying the permanent loss in Old World rabbits of an important gene function, which has been well preserved throughout mammalian evolution (*cf*. Figures 3 and 4; a *CCL8-like* gene has also been reported in bony fish [GenBank: BT048349]). One explanation could be that functions of *CCL* products can be redundant or interchangeable, at least in leporids, which would be at odds with the evolutionary perpetuation of *CCL* gene identity. More interesting is the hypothesis of a causal link between the appearance within the lineage of Old World rabbits of the alteration at the second external loop of CCR5 on one hand, and the pseudogenization of one of its prime ligands on the other. Both events are highly unusual and none of them did occur in the *Sylvilagus*-*Lepus* lineage, nor in any other studied species. Although the argument is somewhat circular - we looked at the *CCL8* gene precisely because of the CCR5 alteration - it can offer a plausible explication for the knock-out of an otherwise prominent gene function over a period of more than 4 My (*cf*. Figure 3). If “lost by accident”, the *CCL8* gene could during this period have been repaired by back-mutations or by gene conversion with one of its neighbors. One might consider that receptor alteration occurred first, making *CCL8* either useless or even detrimental, allowing or forcing its permanent pseudogenization. Or on the contrary, the *CCL8* gene knock-out, and the consecutive perturbation of CCR5-dependent signaling pathways (e.g. due to the loss of a potential (ant)agonist of other CCR5 ligands such as Rantes/CCL5), may have favored structural change at its orphaned target (i.e. at the second external loop of CCR5). Regardless of the scenario, we could be facing an irreversible situation where a “gene knock-out” resulted in a gene “lock-out”. Indeed gene repair would not be favored by selection if the recovered ligand can no longer recognize its receptor (why repair the key if the lock has changed). It might therefore be interesting to know to what extent, if at all, the receptor mutation impairs the affinity of the different CCR5 ligands.

A further question beyond our competence is whether, when, and in which cellular environment *CCL8* is expressed in *Sylvilagus* during MYXV infection and how, at the event, it contributes to the clearance of infected lymphocytes. In this context it might be interesting to mention that MCP-2 expression was down regulated in human HIV infected brain cells by miRNA146a [49], a microRNA which we found to be present in Rabbit (the miRNA146a sequences are identical among Human and Rabbit and are localized in same chromosomal region; WvdL unpublished observations).

## Conclusions

The large number of host-specific immunodulatory proteins encoded by MYXV implies multiple levels of elaborate interactions between the virus and its natural host which can be the outcome of thousands of years of co-adaptive evolution. Identifying the constituents of this interplay remains a huge challenge*,* as host factors involved can be even more numerous. It might therefore be worthwhile to consider that the knock-out of a single host factor could severely affect this virus-host equilibrium. Given that monocyte chemotactic proteins control patterns of leukocyte migration, which in turn govern the outcome of MYXV infection in rabbits, the observation of a factual correlation between the near absence of MYXV virulence and the concurring presence of a functional MCP-2/*CCL8* and an ‘intact’ *CCR5* gene, could promote studies on the role played by this particular chemokine ligand-receptor interaction in keeping Myxoma virus under control.

## Methods

Tissue samples specimens of leporid species belonging to genera *Oryctolagus*, *Lepus* and *Sylvilagus* were provided by CIBIO Lagomorph Tissue Collection maintained by Paulo C. Alves (CIBIO, Vairão, Portugal; http://pcalves@mail.icav.up.pt). Species and sample names are listed in Table 2. All samples are from wild populations.

**Table 2:** Species names and their abbreviations, and sample names (inclusive geographic origin), of studied specimens

**Species:**	**References and sample names:**
**European rabbit:**	
*Oryctolagus c. cuniculus* (Occ):	OccTar104, OccTar109, OccAlt104, OccZrg18 (Spain)
*Oryctolagus c. aligirus* (Oca) :	OcaPan3, Ocaped1, OcaPed9, OcaMert35 (Portugal),
	Oca32 ^c)^, OcaHue54 (Spain)
**South African Riverine rabbit:**	
*Bunolagus monticularis* (Bumo):	Bumo, one sample of gDNA donated by Mathew, South Africa.
**Cottontail rabbit:**	
*Sylvilagus floridanus (*Syfl) ^-^:	Syfl-161, Syfl-162, Syfl-172
**Hare:**	
*Lepus timidus* (Leti) *:*	Leti2012, Leti2191 (Finland)
*Lepus granadensis* (Legr)	Legr2^c^, Legr6^c^, Legr2016. Legr2061 (Portugal),
	LegrCrd905 (Spain),
*Lepus europeus* (Leeu) :	Leeu1^c^, Leeu2^c^ (Spain)

^**c)**^ sources of cDNA, all others were sources of gDNA.

### gDNA

For the genomic DNA extraction of *Oryctolagus* (9 specimens) and *Lepus* (5 specimen) we used liver tissues preserved at -20°C in RNA stabilizing medium. We were privileged by the generous gift of *Bunolagus* gDNA prepared by Conrad Mathee. For *Sylvilagus* we only disposed of blood clots with mRNA not suitable for cDNA synthesis. Genomic DNA was extracted using the EasySpin Genomic DNA Minipreps Tissue Kit (Citomed) according to manufacturer’s instructions.

### cDNA

Total RNA was prepared for one wild rabbit (*Oryctolagus cuniculus algirus*: Oca32), and four hares (genus *Lepus*). The hare specimens represent two species: Iberian hare (*Lepus granatensis*: Legr2, Legr6) and European brown hare (*Lepus europaeus*: Leeu1, Leeu2). RNA was extracted using the guanidinium thiocyanate-phenol-chloroform extraction method (TRIzol) according to manufacturer’s instructions (Molecular Research Center, Inc., Cincinnati, OH, USA). Next, first strand cDNA was prepared from 5 μg of RNA and synthesized using oligo (dT) primers [50]. The putative *CCL8* and *CCL7* transcripts were PCR-amplified using a primer set located in the UTR regions.

### PCR

**Figure 8:**
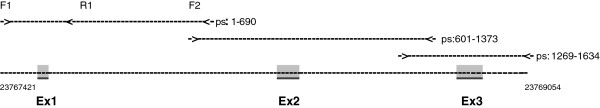
**Schematic presentation of primer positions on the rabbit *CCL8 *ortholog.** Primers were designed to cover all exon and intron regions of the proposed Rabbit ortholog of the Human *CCL8* gene (1634 bp) [Genbank NC_013687.1:REGION:23767421.23769054]. ‘>’: position of forward primer; ‘<’: of reverse primer; ‘ps’: PCR fragment length.

Primers were designed according to the R-MCPgb fragment of the Rabbit chromosome 19 [GenBank: NC_013687.1_REGION:23720000.23798000], using the online software Primer-Blast provided by NCBI [51]. For amplification of genomic *CCL8*, primers were designed separately for each of the three exons, in a way covering all coding and intron regions (see Figure 8). Primer pairs are listed in Table 3. PCR methods were standard. Details are given in Additional file 8. For the sequencing reactions we used ABI PRISM BigDye Terminator v3.1 Cycle Sequencing Kit and protocols were followed according to the manufactures. The sequencing reactions were cleaned with Sephadex™ from GE Healthcare Life Sciences. Sequencing was performed on an ABI PRISM 310 Genetic Analyser (PE Applied Biosystems). PCR products were sequenced in both directions.

**Table 3:** List of primer pairs

**Gene**	**Exon**	**Primer name**	**Sequence**	**R-MPCgb**
**gDNA**				
*CCL8*	ex1	FwPrCL8e1	5’ AGCACACGCAGGGTCTTGCT 3’	47421-47440^**Â§**^
		RvPrCL8e1a	5’ ATGGCTCCCACTTGGATGGC 3’	47692-47711
		RvPrCL8e1b	5’ TCGACCCCGTGGGCTGGTAG 3Â´	48091-48110
	ex2	FwPrCL8e2a	5’ GCATCCAGCACGGTGGCTGT 3’	48021-48040
		RvPrCL8e2a	5’ GCCAGCCCTTGCTCCTTGGG 3’	48774-48793
	ex3	FwPrCL8e3b	5’ GGCTCCAGGTGCTTCAGCCA 3’	48659-48678
		RvPrCL8e3b	5’AGTACCCAGGGAAGGCTGGG 3’	49034-49054
*CCL13*	ex1	F1CL13e1	5’ TTGGCTCTCCCGTGGCAGCA 3’	72054-72073
		R1CL13e1	5’ GGCCAGCACTATGGCGCAGT 3’	72537-72556
		F9CL13e1	5’ AGGCAGCAAGCATGGGAGCG 3’	71722-71741
		R9CL13e1	5’ GGGCCCTTTGGCTTAGAAGGCG 3’	72226-72206
**cDNA**				
*CCL8*	CDS	Fw*CCL8*_CDS	5’ CTCCAAGCTCGGCTCCTTG 3’	47548-47566
		Rv*CCL8*_CDS	5’ ACTCTGAGTCTCAGTCCAGGTG 3’	48990-49011
*CCL7*	CDS	Fw*CCL7*_CDS	5’ AGGCTGAGGCCAGCACAGGA 3’	13720-13739
		Rv*CCL7*_CDS	5’ TCCTCCCCCATGCCAGTGCA 3’	11541-11560

^**Â§**^ NC_013687.1 REGION:2376421.23767440.

### Source of data

All sequence data except those produced in this study were obtained from GenBank database of the NCBI platform [52] or Ensembl [53]. The GenBank accession numbers of nucleotide sequences used in this study are listed in Additional file 9. Sequences produced in this study were submitted to GenBank and the accession numbers are listed in Table 4.

**Table 4:** GenBank accessions of novel nucleotide sequences

**Description**	**GenBank Name**	**GB Accession**
Legr_CCL8_cDNA	CCL8_Legr2	JX000247
Leeu_CCL8_cDNA	CCL8_Leeu1	JX000248
Legr_CCL8_cDNA	CCL8_Legr6	JX000249
Leeu_CCL7_cDNA	CCL7_Leeu2	JX000250
Legr_CCL7_cDNA	CCL7_Legr6	JX000251
Occ_CCL7_cDNA	CCL7_Occ32	JX000252
Occ_CCL8_gDNA	CCL8gDNA_Occ_Tar109_1	JX000253
Occ_CCL8_gDNA	CCL8gDNA_Occ_Tar109_2	JX000254
Occ_CCL8_gDNA	CCL8gDNA_Occ_Alt104_1	JX000255
Occ_CCL8_gDNA	CCL8gDNA_Occ_Alt104_2	JX000256
Occ_CCL8_gDNA	CCL8gDNA_Occ_Tar102_1	JX000257
Occ_CCL8_gDNA	CCL8gDNA_Occ_Tar102_2	JX000258
Occ_CCL8_gDNA	CCL8gDNA_Occ_Zrg18_1	JX000259
Oca_CCL8_gDNA	CCL8gDNA_Occ_Zrg18_2	JX000260
Oca_CCL8_gDNA	CCL8gDNA_Oca_Pan3_1	JX000261
Oca_CCL8_gDNA	CCL8gDNA_Oca_Pan3_2	JX000262
Oca_CCL8_gDNA	CCL8gDNA_Oca_Ped1	JX000263
Oca_CCL8_gDNA	CCL8gDNA_Oca_Ped9_1	JX000264
Oca_CCL8_gDNA	CCL8gDNA_Oca_Ped9_2	JX000265
Oca_CCL8_gDNA	CCL8gDNA_Oca_Mert35_1	JX000266
Oca_CCL8_gDNA	CCL8gDNA_Oca_Mert35_2	JX000267
Oca_CCL8_gDNA	CCL8gDNA_Oca_Hue54	JX000268
Bumo_CCL8_gDNA	Bumo_CCL8	JX000276
Legr_CCL8_gDNA	Legr_CCL8	JX000277
Syfl_CCL8_gDNA	Syfl_CCL8_1	JX000279
Syfl_CCL8_gDNA	Syfl_CCL8_2	JX000280
Oca_CCL13_exon1_gDNA	Oca_Pan3x_CCL13ex1	JX020976
Oca_CCL13_exon1_gDNA	Oca_Pan3y_CCL13ex1	JX020977
Oca_CCL13_exon1_gDNA	Oca_Ped1_CCL13ex1	JX020978
Oca_CCL13_exon1_gDNA	Oca_Ped9_CCL13ex1	JX020979
Leti_CCL13_exon1_gDNA	Leti_2061_CCL13ex1	JX020980
Syfl_CCL13_exon1_gDNA	Syfl_161_CCL13ex1	JX020981
Syfl_CCL13_exon1_gDNA	Syfl_162_CCL13ex1	JX020982
Syfl_CCL13_exon1_gDNA	Syfl_171x_CCL13ex1	JX020983
Syfl_CCL13_exon1_gDNA	Syfl_171y_CCL13ex1	JX020984
Syfl_CCL13_exon1_gDNA	Syfl_176_CCL13ex1	JX020985

Sequences were obtained by PCR using either genomic DNA (gDNA) or reverse transcribed mRNA (cDNA). Species names are indicated by abbreviations (Legr: *Lepus granatensis*; Leeu: *Lepus europaeus*; Leti: *Lepus timidus*; Bumo: *Bunolagus monticularis*; Syfl: *Sylvilagus floridanus;* Oc: *Orytolagus cuniculus*). The two subspecies of *Oryctolagus cuniculus* are distinguished (Occ: *O. cuniculus cuniculus*; Oca: *O. cuniculus algirus*). Extensions (x and y) refer to reproducible sequence ambiguities in PCR products obtained from a same individual that can be explained by allelic variation. Legr_CCL8 is a consensus sequence of Legr2016 and Legr2061; Syfl_CCL8_1 and _2 are two putative alleles inferred from sequences obtained with Syfl-161, -162, -171, -172, -176.

### Sequence analysis

Alignments were done using the online software “Align” provided by the NCBI site in combination with online software Dialign [54] and Clustal W as incorporated in the MEGA5 package [36] and improved by visual corrections using BioEdit [55]. Phylogenetic analysis shown was conducted using Maximum Likelihood method provided in MEGA5 [36]. The probability of gene transcription of undocumented CCL orthologs was evaluated with GenScan [29].

## Endnotes

a) MCP-2/*CCL8* and similar. Read: either the Monocyte chemotactic protein type 2 encoded by the gene *CCL8*, or the *CCL8*gene encoding the MCP-2 protein, depending on context. Maintaining the MCP nomenclature for proteins is preferred because used in studies of chemokines function.

b) Rabbit and rabbit: Species name are capitalized when used to avoid irrelevant repetitions of scientific names (European rabbit or *Oryctolagus cuniculus*)*.* Thus “Rabbit genome” or “Rabbit sequences” but “rabbits were collected”. By analogy we write Human, Cottontail rabbit etc. depending on context.

## Abbreviations

MIP: Macrophage inflammatory proteins; MCP: Monocyte chemotactic proteins; R-MCPgb: Rabbit MCP-Eotaxin WGS fragment [Genbank: NC_013687 REGION: 23720000.23798000]; MYXV: Myxoma virus; MHC-II: Major Histocompability Complex Class 2; HIV: Human Immunodeficiency Virus; CCL: CC chemokine ligand; CCR: CC chemokine receptor; WGS: Whole Genome Sequence; i.e.: *In extenso*, more in detail; *e.g.*: *Exempli gratia,* for example; *cf*.: Confer; indel: Insert or deletion in sequence comparisons.

## Competing interests

The authors declare that they have no competing interests.

## Authors’ contribution

WvdL conceived the study and its design and carried out the literature research, data mining and analysis, drafting and editing of the manuscript. The most important findings of present report were nevertheless produced by SA and ALM, who provided the new data which are the core of the paper. SA did the PCR amplifications and sequencing, revealing the existence of a potentially functional *CCL8* gene in *Lepus* and *Sylvilagus* species. ALM contribution was the cDNA work, putting the corner stone to this study by showing that in these species the *CCL8* gene is transcribed. PJE is the leader and JA one of the most inspiring members of the CIBIO Evolutionary Immunogenetic Group, and played an important role in coordinating and supporting the work of ALM and SA, and by critical commenting and stimulating discussion of the manuscript.

## Supplementary Material

Additional file 1 **GenBank Features file for RabbitNC_013687REGION: 23720000.23798000.** Click here for file

Additional file 2 **Alignment of *Oryctolagus cuniculus *and *Homo sapiens *WGS sequences: identifying rabbit ortholog of human *CCL7*.** Click here for file

Additional file 3 **Alignment of *Oryctolagus cuniculus *and *Homo sapiens *WGS sequences: identifying the rabbit ortholog of human *CCL8*.** Click here for file

Additional file 4 **Alignment of *Oryctolagus cuniculus *and *Homo sapiens *WGS sequences: identifying rabbit ortholog of human *CCL13*.** Click here for file

Additional file 5 Alignment of MCP encoding regions of rabbit and human in Fasta format. Click here for file

Additional file 6 **Rabbit *CCL13 *ortholog named ‘ *CCL8’ *or ‘ *CCL7’.*** Click here for file

Additional file 7 **Nucleotide variation at *CCL8 *genes within and among leporid species.** Click here for file

Additional file 8 Detailed PCR procedures. Click here for file

Additional file 9 Genbank Accessions and Links of MCP-Eotaxin mRNA sequences of Placental Mammals used or consulted. Click here for file
