# Supplementary material for: Pseudogenization of the MCP-2/CCL8 chemokine gene in European rabbit (genus Oryctolagus), but not in species of Cottontail rabbit (Sylvilagus) and Hare (Lepus)
Source: BMC Genet. 2012 Aug 15;13:72. doi: 10.1186/1471-2156-13-72 (PMC3511233; doi:10.1186/1471-2156-13-72)
Supplement: Additional file 1 — GenBank Features file for RabbitNC_013687REGION: 23720000.23798000. [file 1471-2156-13-72-S1.doc]

Additional file A1

GenBank Features file for Rabbit [NC_013687](http://www.ncbi.nlm.nih.gov/nuccore/NC_013687) REGION: 23720000..23798000

December 2011

Oryctolagus cuniculus breed Thorbecke inbred chromosome 19,

ACCESSION [NC_013687](http://www.ncbi.nlm.nih.gov/nuccore/NC_013687) REGION: 23720000..23798000 GPC_000000259

FEATURES Location/Qualifiers

source 1..78001

/organism="Oryctolagus cuniculus"

/mol_type="genomic DNA"

/db_xref="taxon:[9986](http://www.ncbi.nlm.nih.gov/Taxonomy/Browser/wwwtax.cgi?id=9986)"

/chromosome="19"

/sex="female"

/breed="Thorbecke inbred"

gene 272..1931

/gene="CCL2"

/note="Derived by automated computational analysis using

gene prediction method: BestRefseq."

/db_xref="GeneID:[100009130](http://www.ncbi.nlm.nih.gov/sites/entrez?db=gene&cmd=Retrieve&dopt=full_report&list_uids=100009130)"

mRNA join(272..362,1066..1183,1534..1931)

/gene="CCL2"

/product="chemokine (C-C motif) ligand 2"

/exception="mismatches in transcription"

/note="Derived by automated computational analysis using

gene prediction method: BestRefseq."

/transcript_id="[NM_001082294.1](http://www.ncbi.nlm.nih.gov/nuccore/126723025)"

/db_xref="GI:126723025"

/db_xref="GeneID:[100009130](http://www.ncbi.nlm.nih.gov/sites/entrez?db=gene&cmd=Retrieve&dopt=full_report&list_uids=100009130)"

CDS join(287..362,1066..1183,1534..1717)

/gene="CCL2"

/exception="mismatches in translation"

/note="Derived by automated computational analysis using

gene prediction method: BestRefseq."

/codon_start=1

/product="small inducible cytokine A2 precursor"

/protein_id="[NP_001075763.1](http://www.ncbi.nlm.nih.gov/protein/126723026)"

/db_xref="GI:126723026"

/db_xref="GeneID:[100009130](http://www.ncbi.nlm.nih.gov/sites/entrez?db=gene&cmd=Retrieve&dopt=full_report&list_uids=100009130)"

gene 11603..21322

/gene="LOC100343840"

/note="Derived by automated computational analysis using

gene prediction method: GNOMON. Supporting evidence

includes similarity to: 3 Proteins"

/db_xref="GeneID:[100343840](http://www.ncbi.nlm.nih.gov/sites/entrez?db=gene&cmd=Retrieve&dopt=full_report&list_uids=100343840)"

mRNA join(11603..11678,20714..20822,21217..21322)

/gene="LOC100343840"

/product="small inducible cytokine A11, transcript variant

2"

/note="Derived by automated computational analysis using

gene prediction method: GNOMON. Supporting evidence

includes similarity to: 1 Protein"

/transcript_id="[XM_002719227.1](http://www.ncbi.nlm.nih.gov/nuccore/291405570)"

/db_xref=“GI: 291405570”

/db_xref="GeneID:[100343840](http://www.ncbi.nlm.nih.gov/sites/entrez?db=gene&cmd=Retrieve&dopt=full_report&list_uids=100343840)"

CDS join(11603..11678,20714..20822,21217..21322)

/gene="LOC100343840"

/note="Derived by automated computational analysis using

gene prediction method: GNOMON."

/codon_start=1

/product="small inducible cytokine A11 isoform 2"

/protein_id="[XP_002719273.1](http://www.ncbi.nlm.nih.gov/protein/291405571)"

/db_xref="GI:291405571”

/db_xref="GeneID:[100343840](http://www.ncbi.nlm.nih.gov/sites/entrez?db=gene&cmd=Retrieve&dopt=full_report&list_uids=100343840)"

mRNA join(19509..19584,20714..20822,21217..21322)

/gene="LOC100343840"

/product="small inducible cytokine A11, transcript variant

1"

/note="Derived by automated computational analysis using

gene prediction method: GNOMON. Supporting evidence

includes similarity to: 2 Proteins"

/transcript_id="[XM_002719226.1](http://www.ncbi.nlm.nih.gov/nuccore/291405568)"

/db_xref=“GI: 291405568”

/db_xref="GeneID:[100343840](http://www.ncbi.nlm.nih.gov/sites/entrez?db=gene&cmd=Retrieve&dopt=full_report&list_uids=100343840)"

CDS join(19509..19584,20714..20822,21217..21322)

/gene="LOC100343840"

/note="Derived by automated computational analysis using

gene prediction method: GNOMON."

/codon_start=1

/product="small inducible cytokine A11 isoform 1"

/protein_id="[XP_002719272.1](http://www.ncbi.nlm.nih.gov/protein/291405569)"

/db_xref=“GI: 291405569”

/db_xref="GeneID:[100343840](http://www.ncbi.nlm.nih.gov/sites/entrez?db=gene&cmd=Retrieve&dopt=full_report&list_uids=100343840)"

End of file
