# Supplementary material for: Pseudogenization of the MCP-2/CCL8 chemokine gene in European rabbit (genus Oryctolagus), but not in species of Cottontail rabbit (Sylvilagus) and Hare (Lepus)
Source: BMC Genet. 2012 Aug 15;13:72. doi: 10.1186/1471-2156-13-72 (PMC3511233; doi:10.1186/1471-2156-13-72)
Supplement: Additional file 2 — Alignment of Oryctolagus cuniculus and Homo sapiens WGS sequences: identifying rabbit ortholog of human CCL7. [file 1471-2156-13-72-S2.doc]

Additional File A2

Alignment of *Oryctolagus cuniculus* and *Homo sapiens* WGS sequences: identifying rabbit ortholog of human *CCL7*

orcu *CCL7* CDS join(11603..11678,12913..13030,13429..13534) NC_013687.1_REGION:23720000..23798000

hosa *CCL7* CDS join(15241..15316,16096..16213,16647..16752) NC_000017.1 REGION:32582070..32692000

TATA-box |UTR’3 exon1
orcu : ccccacccctcatcctttt----gcaTATAATAGGCAGAGGCATAGCCTCCCCAGGAGCAGAGAGGCTGAGgccagcacaggaacctgcagctctccc-tccaagctcgcctcctcgcgctccagc---atgcaaatCTCTGCAGCACTTCTGTGCCTGCTGCTCACAGTGGCTGCcTTCAGCTCCCAGGTGCTTGCCCA : 11673
hosa : ----------catcctctctgcttccTATAAAAGGCAGAGACAGAGCTTCCAGAGGAGCAGAGGGGCTGAGaccaaaccagaaacctccaattctcatgtggaagcccatgccctcaccctccaac---atgaaagcCTCTGCAGCACTTCTGTGTCTGCTGCTCACAGCAGCTGCtTTCAGCCCCCAGGGGCTTGCTCA : 15311

 Intron1
orcu : GCCAG-GTGAGGTCCCcccctcctccccgtgagacacagatctctccatctcctctccaggtgttcagggactgccacagcgggaggcgtcCCCACAGTCTCACTTGAACAGCTGCTTTTCCAAGCTAAGGtagctcatgggccaggagggagcccagtcacaccgcagctcc------GGGCAGAGCCTGAGCTCCAAT : 11866
hosa : GCCAG-GTAAGGTCCCtctctccttctccttgaagcacattgccccctctctgggttatcctggaccaatcaagaagacctgata------CCCACAGTCTCACTTTAACAGCTACTTTTCCAAGATAAGGtaacttagaaaaaggataaggggtgagcccaaccacacagctgctgttGGGTAGAGCCTGAACTAGAAT : 15504


orcu : TCCAGCGGTGAACCCCAcatctggctcc-TCTGGGTTTCCAACACTGGAAACACCCTCAGgcagtcgccacctagctgctgtcagtagggttcagagttgggggtgatctaagaggggaccgcaggtggcgggtgtgcagggcacttccaagatatggaagggaggagaggagacccagaatgctctgtgctttagttgg : 12065
hosa : TCCAGCTGTGAACCCCAaatccagctcctTCTAGGATTCCAGCTCTGGGAACACCCTCAGtgcagttaccactccagctgcttccagcagaatttgggatcagggtgatcaaagacaggaggcttctggggatgggtgtgcgggctgtttccagataccgggagacccagaatctggtctgtggaagcccagcttccaga : 15704


orcu : cccagcttctgcagaggcagtgggcacaggggtccccaactgctattattctctggccctgatcattggctcatgtgcccctcctatgacctcttcagaatcaccttgtttgaacagaaccttcttctagcccagcctccagcaaagtagctctgcagagacaatggatttttggagcctaaaagaggcatgtaactggg : 12265
hosa : aacagcagctctgcagaggtggtacgtatcagggaaactcatgaccaagcattgaatgctcagagcctaaaaggggatccatagttggggtacccttgctctaaggaattggattattatattagcccctcctagcaatgcccagagtagccatcaattcctcttccg-------------------------------- : 15872


orcu : gtctccctgcccccagggcattgcattattggacgacccttccTCTATCAGCTGGTGATGCTTCATGCCCCACTTATAGTCCAtgagagtgaGGGTTTAAGGAAAGATCTCAAAGAGCAGAGACATTGAAGCCGGCGCCGTGGCTCAATAGGCTAATCCTCCACCTTGCGGCGCCGGCACACCGGGTTCTAGTCCCGGTT : 12465
hosa : -------------------------------------------TCTTTCAACTGGTGATGGTGCATCCCTATTTCACAGTCCAtaaaagtga------------------------------------------------------------------------------------------------------------ : 15921

orcu : GGGGCGCCGGATTCTGTCCCGGTTGCCCCTCTTCCAGGCCAGCTCTCTGCTATGGCCAGGGAGTGCAGTGGAGGATGGCCCAGGTGCTTGGGCCCTGCACCCCATGGGAGACCAGGAAAAGCACCTGGCTCCTGGCTCCTGCCAGGATCAGCGCGGTGCGCCGGCTGCAGCGGCGGCCATTGGAGGGTGAACCAACGGCA : 12665
hosa : --------------------------------------------------------AAGGGAGTTTA-TGAA--ATGCCTC----------------------------------------------------------------------------------------------------------------------- : 15943

orcu : AAGGAAGACCTTTCTCTCTCTGTCTCTCTCTCTCACTGTCCACTCTGCCTGTCAAAAAAAAAAAAAAAAAAAAACTAATTAAAATTTAAAATTACAAAAAAAAAAAGAGCAGAGACATTGAACTTGGGGTGGGCAGCTTCTCCccctccgtctccctttctctcccgttctgccttccaactATTCCCTCCTTGACAAAT : 12865
hosa : -------------------------------------------------------------------------------------------------------AAAGGGCAGAGACATTGGGTTTGGGATGGGCAGCTTTTCCctccacctcttcctttctttctgattccttcttcttaccATTCCCTGTTTTACAAAC : 16040

 Exon2 Intron2
orcu : AAGGAGACCCAGAGAACACCCTgaagagactgc--------TTGTTTCATTGCAG-AAGGGACTAAcagcggcaaaACCTGCTGCTACAGATTccacaacaggaggatggacccgcagaagctgaggagctacacactcatcagcatcAGCTACTGTCCCCGGGAAGCTGTGAT-GTGAGTGGACCATGgccagcaccct : 13055
hosa : AGAAAGACCCAGGACACACCCTcaatggacttttcttcttgTTGTTTCATTGCAG-TTGGGATTAAtacttcaactACCTGCTGCTACAGATTtatcaataagaaaatccctaagcagaggctggagagctacagaaggaccaccagtAGCCACTGTCCCCGGGAAGCTGTAAT-GTATGTGGACGATGaccacccaccc : 16238


orcu : cagcccgcagtcctgagctctcctctagggtaacagagaggaccacggtgcccagcctgggtagagacactctcagccccggaagctttccggtgggaaagct----------------------gaagccaagc-aaaaacatggcttcctgcttcctgcttccagcttccttgctggtcttcagttcagagctcagtt : 13232
hosa : ctcacacctcagtcctaggttcttccctgggcagggaataggactagtatcagaatgagttggagtcaaatactgtgatgcatacagcatctctaaccttatcccagacatttgccagtgagaaacaatacaagtaaagaaagtggcttctcactctcagctcc--ctttccagctatcattttacatctcagttcgttc : 16436


orcu : ccttcatcccagagcccaggagacttcacccaggccgacgagagcgctgcttcccctgagg-CTCTTCCTTTGTCTTGTCTTCCTCCTCCTCCACTGGGGTAgccccacca------------GCCCAGACAGGTTTCCCACAGCATAGGTTACACCCCTCGGttaaatcctcttaagcacattgtctaaccgtcctttc : 13419
hosa : cttcatcctggaaccaagagagattcacttgggctaccaaaaagagctgcttc-tctgagtcCCCTTCCTTTGTTTTATCTTCTTCCTTCATCCCTGAGGCAtccccatcagctaggctgatgGGCTAGACAGATTTCCCATAGACTTGGTCACACTCCCAGGctgaaccctcaaggtgttccatctgactgtctccttt : 16635

 Exon3
orcu : tcc--CCACAG-CTTCAAGACCAAACAGCACCGAGAGGTCTGTGCTgaccccaagtggccatgggcccaaaatgccattgcctACCTGAACAAGAAAACTCAGACTTCAAAGCCTTGA---GCAgTCTTGCCTGCATTAAATCCAAGCCTGGATTTGAGAAGCAAgtaacctgtgtccactcgcttcaactcaagagttg : 13613
hosa : ctgctCCACAG-CTTCAAGACCAAACTGGACAAGGAGATCTGTGCTgaccccacacagaagtgggtccaggactttatgaagcACCTGGACAAGAAAACCCAAACTCCAAAGCTTTGA---ACAtTCATGACTGAACTGAAAACAAGCCATGACTTGAGAAACAAataatttgtataccctgtcctttctcagagtggtt : 16831


 * 18820 * 18840 * 18860 * 18880 * 18900 * 18920 * 18940 * 18960 * 18980 * 19000
orcu : tgcagagattatcttgttgTAATTCTAAGAAATAGGAGCTTTGTGTAGTCGTGTGAATCacagttttccttaaatatttttaagttattagcaccttaatttaacttgcactggcatgggggaggagtttgaactgtaaagccttgtacatactgtgccattttattatagaattgattattttatatgtgttattcttg : 13813
hosa : ctgagattattttaatc--TAATTCTAAGGAATATGAGCTTTATGTAATAATGTGAATCatggtttttcttagtagattttaaaagttattaatattttaatttaatcttccatggattttggtgggttttgaacataaagccttggatgtatatgtcatctcagtgctgtaaaaactgtgggatgctcctcccttctct : 17029


 * 19020 * 19040 * 19060 * 19080 * 19100 * 19120 * 19140 * 19160 * 19180 * 19200
orcu : c-----------------------------------------------------------------------------------------------------------------------------------ATTTTTACATAAAATATATTTTTGTAAAAaacttgactttgatgactttttaaaagatgaaacggaac : 13882
hosa : acctcatgggggtattgtataagtccttgcaagaatcagtgcaaagatttgctttaattgttaagatatgatgtccctatggaagcatattgttattatataattacatatttgcatatgtatgactcccaaATTTTCACATAAAATAGATTTTTGTATAAcagctgccattcatggttttttaaaaggataaagtaata : 17229

Coding regions are underlined by
