# Supplementary material for: Pseudogenization of the MCP-2/CCL8 chemokine gene in European rabbit (genus Oryctolagus), but not in species of Cottontail rabbit (Sylvilagus) and Hare (Lepus)
Source: BMC Genet. 2012 Aug 15;13:72. doi: 10.1186/1471-2156-13-72 (PMC3511233; doi:10.1186/1471-2156-13-72)
Supplement: Additional file 3 — Alignment of Oryctolagus cuniculus and Homo sapiens WGS sequences: identifying the rabbit ortholog of human CCL8. [file 1471-2156-13-72-S3.doc]

Additional File A3

Alignment of *Oryctolagus cuniculus* and *Homo sapiens* WGS sequences: identifying the rabbit ortholog of human *CCL8*

orcu *CCL8ps* join(47576..47641,48337..48455,48872..48977) NC_013687.1_REGION:23720000..23798000

hosa *CCL8* CDS join(64452..64527,65219..65336,65752..65857) NC_000017.1 REGION:32582070..32692000

orcu : CAAGGCTCTGTGGaactctCTGGGGATCTAAAGGACCCTTCCTGGGTTGGAtgcatgcaagcta--AGTGTGATGGAGAGCACAGGCAAAGCCTTTGAACCCATCtgagt-CTCCTGGTACCCACAGAGGGGCGGGTCACTGCCTCTCcacTCCACTATGACCCCGGTTTCCCTTCTTCCTTTCCTACAGAtcATTTTCC : 47255
hosa : CAACGTTCTGTGG------CTGGGGTTCTAAAGGAGCTTGCCTGGCTTAGAactgcaagtgactctAGTGTGATGGAGAGCACCAGCAAAGCCTTAGGGCCCATCcctggcCTCCTGTTACCCACAGAGGGGTAGGCCCTTGGCTCTCt--TCCACTATGACGTCAGCTTCCATTCTTCCTTTCTTATAGAcaATTTTCC : 64125


orcu : ATTTCAAAGAAATCTGATCCCTTAATAacccaatgagatcacttCCTACGCTTTGTCAGGCACAATAC-ACACCCTTTCACTTCTGCTCCACAAACTCGAAGCAAAACATGGAAACTTTGCAGCTTCCTT--------ATCTGGAAATCACCCCACG--CTGATATCTACTCAGCAAGCACACGCAGGGTCTTGCTTCAT : 47444
hosa : ATTTCAAGGAAATCAGAGCCCTTAATAgttcagtgaggtcactt---------TGCTGAGCACAATCCCATACCCTTCAGCCTCTGCTCCACAGAGCCTAAGCAAAAGATAGAAACT-CACAACTTCCTTGTTTTGTTATCTGGAAATTATCCCAGGATCTGGTGCTTACTCAGCATATTCAAGGAAGGTCTTACTTCAT : 64315

 FrameShift
 47576

orcu : CCTTCTTTGATTG---CCCTGTCAAGGCTCAGT--TCCCTATAAAGGGCAGGCGGGGCCCCCaGAGGAGCAGAGAGGCTGAGACCAACCCAGACACCTGCAgctctccctcc-aagctcggctccttgagtgccagc--ATA----------CAGTGCTTCTGAGCCTGCAGCTCGTGGTGGCCGTCTTCAGCCCCCAGG : 47626
hosa : TCTTCCTTGATTGTGACCATGCCCAGGCTCTCTGCTCCCTATAAAAGGCAGGCAGAGCCACC-GAGGAGCAGAGAGGTTGAGAACAACCCAGAAACCTTCAcctctcatgctgaagctcacacccttgccctccaag--ATGaaggtttctgCAGCGCTTCTGTGCCTGCTGCTCATGGCAGCCACTTTCAGCCCTCAGG : 64512
 TATA box |UTR’3
 47**6**41
orcu : TGCTCACCTAGCCAG-GTgaggctcttccttcgctaagtcttagcacttgagctacctcagctaccaccatccaagtgggagccatcccagacaggctgcatcttcttcattatgaagaaggagagaggccaagacaggagccaaaaaagagccacctcccatcagagccacaacttagttccgaggttCTGAGGCCAGT : 47825
hosa : GACTTGCTCAGCCAG-GTaagacctctccctttttaag--------------------------gggagaccaaaagaggaattaagaagagccattatgtcacagctcattaggaacaaaaccagaactaaaggc--------------------------tcag-gtca------------------CTGAGGCTGGT : 64640


orcu : CCCTACGATCATCCTTGACACCAGCTTTGggGAGTCAGGCTGAGTGCAGCCACTACGTTTACG--CCGCC--TTGTTTGGGGAAGTGGTACAGAAGGAAGCACCATTTCTggtcGTGGGTGTGGAAGACTTTTCACGCAGCAGTGGGGAGGAAGaagtccctgcctgtggtctcacattttgGGTTATGGTCTAGTGTAG : 48021
hosa : TCCCTTGATCTTTCCTGACCCCAGTTTTGgg-AGG-AGAC--AGTGGAGCCGCTACAGCAACAACCCTCCCATTGTTTGGGGAAATAATCCAGAACGAAGAACTGTTTCTcactGTGGGTGTAAAGGACATTTCAGGCCGTAGTGGAGAGGGAGaaactattgcctgaagcttcaaatttt-GGTTATGGTTCAGTGTAC : 64835


orcu : CATCCAGCACGGTGGCTGTGTAaggaggataagcacCCCCGAGGAATCTCAGCACATGACACAGGTTAGCTACCA--GCCCACGGGGTCGACTAGCCGAGAACCAACGCATGTGCAGCTT-TG----CA-------------TGGGCCAACAGGGAAGAGC---CTTCTTACCCAA---GTTTCCCGGGATCCA-CCAAC : 48194
hosa : CTTCCAGAACAGTGGCTGTGTAaagaggatgagga-CCCAGAGGAATCTCAGCGTATGGCATAGGCTAACT-CTAAAGCCCATGAGGATGAAAGACTGGGAAGCAAGGTAT-TGGAACTTATGTTCCCAGTGTCAGAAGTTTTGGGTTAGTAGACAAGGACTAGCTTGTTACTCAAAATGTTTCC--AAACCCAGTCAAC : 65030


orcu : A----CGG-CTG--G-GTT--GT-G--------------CGGGCGAGGTTTTACCTCTGGGGTC-------AG-----GA-CTTGGCAGGGTGACCCCT-GGCCCCTTCCCACCTTCTGCCTCCTT-C---GCGGGTGCATTTTTCCTGTGTATCCT----GTCTCACTTGTTGTGAAATTTCTTTCAG---ATTCCGTT : 48344
hosa : AATGACGGGCCGCAGAGTTCAATAGAGGAAAGAGACTCACAGGCAACATTTTATCTCTGGGATCTGGACTAAGACACTGAACTTGGGATGGTGACTTCTTGGTCTTCTCCTTCCTTCT-CTTCTTTTCCTTACAAATGCACACTTACGGTGGGTCCTAAATGTCTCATTCTTTGCAAAATTTCTTTCAG---ATTCAGTT : 65226

 FrameShift -------Dir-Repeat----------------
orcu : TCCATCCCAGTCACCTGCTGCTTTGGTGCGGTCAGCAGAAAGATTCCCATCCAGAGGCTGGAGGGCTACACGAGAATCACCAGTGCCCAGCGTCCCCGG--gGCAGCTGTGAT-aTGAGTGCACcaggccCCCGGgGCAGCTGTGATaTGAGTGCACCAGGCCAGCTCTCCCAAACTTCTCTCTGAGAAGCAAGGGAAAG : 48541
hosa : TCCATTCCAATCACCTGCTGCTTTAACGTGATCAATAGGAAAATTCCTATCCAGAGGCTGGAGAGCTACACAAGAATCACCAACATCCAATGTCCCAAG---GAAGCTGTGAT-GTGAGTGGACagtgcc------------------TG---GCACC--CCCA----TTCAAAAGTTCTGA-TGGACAACATAGAGAAG : 65394

 ---- ------------ ----------------
orcu : TGACCAGGATTCACAGCCACATGAGCCAGATAAATA-GAC--C-ATCTAA--------TCCGC-----AGGGACATTCACCCCAC-A-AAGGAGTCCACAGTCGCCCCAGGC-TCCCTTCTAGGGGCTTGGTGAgatggctccaggtgcttcAGCCAGGAGCCTGGCCGGTGTCACCTGGGCAGCAAGAGCAgaccttcc : 48721
hosa : T---CAAGATTCATGTCCATATGAGTCGGATGCATATAACTTCTATCCAAAGGGCCCCTCTACCCCATAGAGAAACTCAGTCCATGAGAAGGAGTCCATAACTGCTCTAGGATTCCCTTCTAGGGGCTTGGTGAaactaacccaatatctgtAGCCAGGACCCTGGAGGGTTTCACCTGGACAGCAAGAGCAgagcttcc : 65591


orcu : tctagaagccaccctctgcctcccctccctcactcctggaccaggcctc-------TCACCCAAGGAGCAAGGGCTGGCTAGGTCTAGGaccccctgggccacacccctgggcggacccctcaagaggctcacctggttgtcccctttctcttg---CAG-CTTCAAGACTAAGCTGGCCAAGGAGGTATGCGCTGACCC : 48910
hosa : ttctggagcttcttcctcccactcttcccctccctcctctcccgggtccgggtcctTCACCTAAGGACCAAGGGCTGATCAGTTCTAGGgaccaatggcccacagtcctgtgcaggatcttcaaagtcttccatctaattgtgccctctctcccccaCAG-CTTCAAGACCAAACGGGGCAAGGAGGTCTGTGCTGACCC : 65790


orcu : CAGGGAGAAATGGGTCCAGGATTCCATGAAGCTCCTGGACCAAAAGTCCCTAACTCGGAAGCCTTGA---cctccccatgcacaccTGGACTGAGACTCAGAGTCTGAGGCCAACATTATTTATTTcccagccttccctgggtactgtaggacagtacttagttatataatgtaccgaaaggagggttctatttaatcAT : 49107
hosa : CAAGGAGAGATGGGTCAGGGATTCCATGAAGCATCTGGACCAAATATTTCAAAATCTGAAGCCATGA---gccttcataca-----TGGACTGAGAGTCAGAGCTTGAAGAAAAGCTTATTTATTTtccccaacctcccccaggtgcagtgtgacattattttattataacatccacaaagagattatttttaaata-AT : 65981


orcu : TGAAAGCACAGTATTTCTTAGATAATATTTAATTATATtaaagttctggatgtttttgtctctttccaccatgaactcctgtgatggcaagacacaaagccctggcgatgtgtggcttttactttttaatCTGTGAGCCCAGTTAAGTACATGGCAACACGTCCGTGTTtgctttctgggctgcccgtggcgctgtgagg : 49307
hosa : TTAAAGCATAATATTTCTTAAAAAGTATTTAATTATATttaagttgttgatgttttaactctatctgtcatacatcctagtgaatgtaaaatgcaaaatcctggtgatgtgttttttgtttttgttttc-CTGTGAGCTCAACTAAGTTCACGGCAAAATGTCATTGTTctccctcctacctgtctgtagtgttgtgggg : 66180


orcu : ttcttatgaggactggcggtataTGAAACACTTCTGTATTCTTAAGGAATTGGTGCTCCTtgaagctgtgtacttttgttttgttg------------TTGAAGTTATTATTACTGACTATGGAATTTTCAAAGAAAA--------------------ATATAATTTTAAACTACATAGACTTATTTTTCATGGGGTAgc : 49475
hosa : tcctcccatggatcatcaagg--TGAAACACTTTGGTATTCTTTGGCAATCAGTGCTCCTgtaagtcaaatgtgtgctttgtactgctgttgttgaaaTTGATGTTACTGTATATAACTATGGAATTTTGAAAAAAAAtttcaaaaagaaaaaaatatATATAATTTAAAACTACTTAGTCTTATTCTTCTTGGGGTAac : 66378

Coding regions are underlined by ; deleterious nucleotide substitutions highlighted in red
