# Supplementary material for: Pseudogenization of the MCP-2/CCL8 chemokine gene in European rabbit (genus Oryctolagus), but not in species of Cottontail rabbit (Sylvilagus) and Hare (Lepus)
Source: BMC Genet. 2012 Aug 15;13:72. doi: 10.1186/1471-2156-13-72 (PMC3511233; doi:10.1186/1471-2156-13-72)
Supplement: Additional file 4 — Alignment of Oryctolagus cuniculus and Homo sapiens WGS sequences: identifying rabbit ortholog of human CCL13. [file 1471-2156-13-72-S4.doc]

Additional File A4

Alignment of *Oryctolagus cuniculus* and *Homo sapiens* WGS sequences: identifying Rabbit ortholog of Human *CCL13*

orcu *CCL13* CDS join(72083..72158,72902..73016,73361..73457) NC_013687.1_REGION:23720000..23798000

hosa *CCL13* CDS join(101477..101552,102425..102539,102976..103081) NC_000017.1 REGION:32582070..32692000

TATA-box?
orcu : ctcagcg--CCGGCTCCAGCTCAGCAGATTTGGGGCCCTCCTcctcatgacttgacctccacctaaccaggaaaaggtcttgtgagacaccccccgcccccaccgtctgCTTCCTATAAAAGGCAGGCAGGATGGCCAGGGAGGAAGAGaagcagagagcaaccctgaggctggaacttggctctcccgtggcagcagcc : 72077
hosa : ctttggtgcCCAGCTCCAGCTCAGCAGATTCAGGATCCCCCTtcatcatgacttggtcaacgccctgctcaggccaaggtcctctgagagttccaagcttctcca----CTCCCTATAAAAGGCCGGCGGAACAGCCAGAGGAGCAGAGaggcaaagaaacattgtgaaatctccaactcttaacctt------------ : 101472

 Exon1 GlnThr Intron1
orcu : cCAGC---ATGAAGGTCTCCGCAGCTCTGCTGTGCCTGCTGCTCctagcggccgcctgcagCTCCCAGGCACTCGCCCAGACAG-GTGAGGCCCATCTCTTCctccaCTCCCTCTCCTTCCTTAGGTTTCTCTcgccttctaagccaaagggccccactttatatatatatatgtatatatatatatatatatatataat : 72273
hosa : -CAAC---ATGAAAGTCTCTGCAGTGCTTCTGTGCCTGCTGCTCatgacagcagctttcaaCCCCCAGGGACTTGCTCAGCCAG-GTAAGTCACCTCCCTTCgact-CTCCCTCTCTTTCCCTCTGTTTCTCTattcaaggaagacctaagcccgagtgctcctccactttttttttagattgagtctcattatgttgcc : 101666
 GlnPro

orcu : gtatttatttatttgaaagtaagagttgcagaaagagagaaagagagagaggcagacagagagaggccttctatccactggttccctccccagatgactgcaatgcccagtgttgggccacaccaaagccaggagccaggagcttcttctaggtctcccaggtgggtgcaggggcccaagcacttgggccatcttccgct : 72473
hosa : caggctgaagtgcaggggtgcgatcttggctcattgcaaccttcacctcccaggttcaagcgattctcttgcctcagccttctgagtagctgtgattacaggcacccgccatcacgtgcagctaatttttgtatttttagtagagaaggggtttcactatgttggccaggctggtctcaaactcttgacctcaagtgatc : 101866


orcu : gctttcccaggccacggcagagagctggatcggaagtggagcagccgggactccaggtttacccactgcgccatagtgctggccctgggGCCCCACTTCTAAACCCAGCAGAGCAGCAAGGCCCCTTAGagcttcactc----------------------AACGCTGAAACCCAGATCGGAGACAGGAGGGGCCCagcc : 72651
hosa : ctcccgtctcggcctcccaaagtgctgggattacaggcgtgagccaccaggcccagccaagt---------------------------GCCCCACTTCTAAGCCCACCAGAATAGTAAGGCTCCTCAGaggttcactttaacatctaattttaaagatagAAAGCTGAAGCCCATGTTGGAGGCAGAAGGGACCCtagc : 102039


orcu : caccttgctgcctggagcccagtgtcttt---------------------------------------------------TCAGGTCATCCCGAACTCTGCAGCTCTGGGGCCACTCCtggcgcctgtcccagggctgcccctggccacctccaggcagtgcagtctgagccacaggtcacagcaggggtgctgggctcc : 72800
hosa : catccacctccaggttattgcagagcaagaatgaaacctaagcttctgactccagatttagggccttttctttgacctcaTCTGATCGTCCCAAACTCTGCAGATCTGGGACCACACCcaggacctttcccactggccttgcccgtggcctcccctagatggctgtgacatgtctccaccatgcagctgagcctttgaga : 102239

 Exon2
orcu : aggacggatgaggtctgcttctgccttcgcctctggtcacttcctgctgcactcaagcccacccgc-------------------------------------------------------------------------------------cttcagaggctttctctttgtgactttgcttctag-Aaacgaagccagc : 72914
hosa : tcctgaggcacatgtcacaggtcccacctcacctcagggtctagggtgggagtgctgggcttgggggtgagtaagatctatttcttcctctttgctttgcatcccatacag-atgctccctgctgtattcaagctgagaaaagcctaacacatcctcaaagtctttttctttgtaactatttctag-Atgcactcaacgt : 102437

 Intron2
orcu : cctgaccgcttgctgcttcagctttgtCAGGAAGAGGATCCCCCTGCAGAGGCTGGTGAGCTATcggaagaccagcaaggccTGTGTCAAGGAGGCTGTGAT-GTGAGTAGAAAA--CCCCGCCCA--TGCACCCCTCCGCAC-CCCATGCTCTCCAAAC-AAAGCTGGGCCC-AGGTGG--GCCCTGCGA--GACT-GA : 73101
hosa : cccatctacttgctgcttcacatttagCAGTAAGAAGATCTCCTTGCAGAGGCTGAAGAGCTATgtgatcaccaccagcaggTGTCCCCAGAAGGCTGTCAT-GTGGGTAGAAAAATCCCTGCTCACCTG-GCTCCTCCCCACTCCCACATTCCCCAATCCAAAGTTCTGCCCCAGGAGACAGACGTCAGACTGACTTGA : 102635


orcu : AGCC--AGG-C-AG--CT--CCTGGCTGC-TAGCCTGAGGACTCTAACTCTC-CCGCTGTAACAG-TGCAGAG-GCAC-TGGGGTCCCTTCACAGGTGGCTGCCC--ATG--CTCTGGTCT-C-CGCTTTAGTGGACACCAAGATGACTTCCCCCGC--CACCAAAAGCAGCCACGGCCTCCAGGG-CCCTTTCT-TCC- : 73277
hosa : GATCTTAGGATGAGATCTAGCCAGACTGTGTGATGCAAATCCTCCAATTTTGGCTGC-ACAACAGGTCCAAAGAGGACCTATAATTTCC-CACACCTTGTTTCCTGGATGGGCACCAGCCCACACCCTTTAGCAGATGCCAGGATCAGTTTCCCAGGGGCAGCAAGAGCAGTGGCTGCCTCCAGAGACCCCTTCTGTCCA : 102833

 Exon3
orcu : CACAA-TCCT----CC---CC---------GCTCC--CAGCC-CAG-----GGACCA-------------CCCA------C----CCTG------CCAT---GGGGT-CCACCTGACTGTGCCGGGCC-CCTTCCTCCACAG-CTTCCGGACCAGACGGGACCAGGAacTCTGTGCTGATCCCATGCAAAAGTGGGTCCA : 73417
hosa : CACACCTCCTACTTCCTGTCCTGGAGGGGTGCCCCTTCACCTGTAGTAGGTGGACCAGGCAGGTTTAGAACCCAGTGTGTCATCTCCTGGGTAAACCCTCAAAGGGTTCCATCTAACTGTGCCAGATCTCCTTCCTCCACAG-CTTCAGAACCAAACTGGGCAAGGAgaTCTGTGCTGACCCAAAGGAGAAGTGGGTCCA : 103032


orcu : GGATaccatgagggtcctggat-GGAAACGTCACTCCCTGAGGACTGCAATTCTTCCACCCacatgcagatgtgacctgagtaggggacatgattgctgcctcttctctggcttcccatgtgcccccagcctggttttcctgtaacc---TTCAAATCGAATGCTTTCTCTTTTATGATGCAAAATGcattctgtcttca : 73613
hosa : GAATtatatgaaacacctgggccGGAAAGCTCACACCCTGAAGACTTGAACTCTGCTACCCctactgaaatcaagctggagtacgtgaaatgacttttccattctcctctggcctcctcttctatgctttggaatacttctaccataattTTCAAATAGGATGCATTCGGTTTTGTGATTCAAAATGtactatgtgttaa : 103232

Coding regions are underlined by ; the unexpected Threonine residue is highlighted as Thr
