# Supplementary material for: Pseudogenization of the MCP-2/CCL8 chemokine gene in European rabbit (genus Oryctolagus), but not in species of Cottontail rabbit (Sylvilagus) and Hare (Lepus)
Source: BMC Genet. 2012 Aug 15;13:72. doi: 10.1186/1471-2156-13-72 (PMC3511233; doi:10.1186/1471-2156-13-72)
Supplement: Additional file 5 — Alignment of MCP encoding regions of rabbit and human in Fasta format. [file 1471-2156-13-72-S5.doc]

Additional file A5

Alignment of MCP encoding regions of rabbit and human in Fasta format.

*orcu_CCL2-rcCCL1*: *Oryctolagus cuniculus* Accession NC_013687.1 REGION: 23720000..23798000

*hosa_CCL2-rcCCL1*: *Homo sapiens* Accession NC_000017.1 REGION: 32582070..32690817

>orcu_*CCL2-rcCCL1*

**gcccccgcatgg--------------TTTGGTCTCAGTGGCAGAAGGATAAAGTGTCCAGTCtaca------GCTTCCTTCTCTTACTTCCTGGAAAcag**

**cccagaactccacttactcagcaaactccagactctgttacatcattccttgaggaagggcactgccatgggccaagccctcccgcccagctcaactgtc**

**ctgacttcctataaaaggcggagacaaAGCCTCCAGAAGCACAGAGAAGCTGAGACCAACTCAGAAgctgccgtctcccactgacgctcggctctcac--**

CCTCCAGC-ATGAAGGTCTCTGCAACGCTTCTGTGCCTGCTgctcatagcagtcgCCTTCAGCTCCCATGTGCTTGCCCAGCCAG-GTAAGT-CCCTACC

TCCTCCCCTGGAAGCACAGATCAGCTCGTCCCCAGGTGATCAGGGACCAGGGACCATCACAGTGGGAG-GGTCCCCACAGTCTTACTTAAACAGCTGGTT

GTGGGAGCTAAGGCAGCTTAGAGAAATACAAAGAGGGA-CCCAGTCACACAGATTCT-cCTGGGCAGAGCCTGAGCTCCAGTCCCAGCTGGGGACCCCAt

ttccaggtctccagtttgtggtgatctaagagggggcctt---------------------------------------------------------AGT

TGGTGGGTGTGCAGGGCACCTCCAGATATGCAGGAGggaaaaggaggcggctcagagTGGTCAGCGCTTCATTCATCCCAGTTTCCCGATAAGcag-CTC

TGCAGAGGCGGTGGACATCAGGGATGCTCACGCCCAtacacagatttgggggacctgaaagaaggtccg-TAGCTGGAGTCCCCAGCTGCTTTTCCCTGG

CTTTGATCATtggcttactagctcctcctaatagttacctcactttaacagccc---------------CCTTCTTTCTGTCCATTATCAACTTTGCACA

TCTATTTTAaagcccggaAAACTGAGTCAGTGTGTCAAAGATCACACTCTGGCTCTGAaattggatttaacagatcctgcctgtc---------------

CTCCTGCCTTCCCTTTGATCTCTTCTCTCAACACGTTTCTgctctgattaagaaatatctc----------------------TCTTTGTGGTTGATTTC

CCAG-ATGCCGTGAATTCCCCAGTCACCTGCTGCTATACATTCACCAGCAAGACCATCTCAGTGaagaggctaatGAGCTATAGAAGAATCAACAGCACC

AAGTGTCCCAAAGAAGCTGTGAT-GTGAGTTGAGCACACCAGCCCTCCCTGGCCcagag---TTCTTGGGGAACCAAGGCACAATCCTTACAAACTTAGA

Gaactacaccaactaatccaatagagaaaacgggggctaag---------------------AGGGAAAAAGCAACTCCCAATGCCACTCTCCAttagta

cttagatcatctcca--------TCTTTAGTCTGAAGTAAGGACGGCTTCACCTGGgacccctggagaAGCAGCTGGCTCTGCGTTCCCTCCTTCCACCT

GCTTTCCT--TCCAGCTCCCATGGCAACCCCTTGTTGCAAGAAGGGCTatacccctaggttgtgactgccaggtacttcatctaactgtgcccCCTCTCT

CCCCACAG--CTTCATGACCAAACTGGCCAAGGgGATCTGTGCTGACCCCAAGCAGAAGTGGGTCCAGGATGCCATTGCCAACCTGGACAAGAAAATGCA

GACACCAAAGACTTtg---acctCTTATTCCACAACCCAAGAACACACGACTAATTTATCTTCcacccggacaccctctactactacttcattgtaa---

ctgcaaaaagtttgaactttattgatATATGAAACACAAGGCCTTAAGTAATGTTAA-TCTTATTTAAGTTATTGATGTTTTgttttaattttatcttcc

atgaatactaatggtttttgttagaatggaaatttgggcatatgtgcttttcctctgTGAACCTCAGTTCTATACCTGGAATATTTTGAGGGTtcttgca

aggatcatgaatgcatgcatattttgttttaaaccccaagatattgctaaagtTATTATTGTGGAAATGAATATTatatataactattgaacaaaatATA

TATTTTTGTACAAAACCTGACTTttggttaTTTTCTTGAGGGAAATGACAAAGCTGGGAGTATGGGCTTGgtgatgagggatgagtATGATTTCAGAGGG

TGGGGCTCATGTTTTGGAAGAATGttttGGAATGTAGATTGGCCCTGGTCTTCactgggcagtctcaactgatgtctacagaagaacttttaaggcaggg

ccccattgggcatttcagcacaatcagtccagtgtccactctctgctctaatccaaatggccctactcagttgcttagcaacagcctt-----ACTCTCA

TTCTTTTACTCACAGTCCATGACCCCTGCccacaacccttttataatctact--CAACTGACCTTTGACTGGGCTACCCAACTCCaatctgtaccctccC

TCCAATGAATCCTCTAAACTGGAGTAATACAGACACTGtgagtgtttgggaattgggggttgaagggacaagggacTTAATAAGCATGAATAAGATAGCT

AGATTAAGATTTCATAggaaaggaaagacttaAACTTACCCTTGATGAATGTAAAAGATGTTTTTAGAAAAtgcaggatcattgagaaaaggagagagat

atgtggga---AAATACCTGGAAGTAGAAAAGAAAAAAATGTGTACAagaaagagggagaaagcctcttgggcaatggggtccaaatctatgtgagatat

gtgggaaaatacctggaagtagaaaagaaaaaaatgtgtacaagaaagagggagaaagcctcttgggcaatggggtccaaatctatgtgatgtggtaaga

attTTAGAAGAGAAGATTGAGTAAAGGATTTTGAActtgaagacaAAAAGTTAGTACTCAACTGTGTAGGTcccagagaaatgctaaaAATCTTTGGTCC

AGTGAGCCAAGATGTGCCATGTacaggggctcagtggtgaggttcagggaatgtaaactcctggcttcatgcagacatggagaagtggagacaggtgaga

gatgattgctgtgatccagGCAGGAGAAGACGAGGGCATGCATATGGAGTCAAAGAGacaacacttgaGAAGAAGGAGCTGGGCAGGGAGAAATGCTGAG

TGTggcaggcagaggagcaggac------CTTAAGATATAGAAGCCTCTTCTTCAGCCCACACTTTAgGGGGACAGCTTCCCTGAAACCAGAATGCATTT

CCCTCTATcgctacctccctccccccagtcccaggagctggggatcatcctctatgtca-------------------------ACCTAAGGCCTGTTTT

CCCTGGCATGGCATTTGACCCACtggTGAGGCATTTGGCCCACCTGGGCACTCACCCCAGGCagtgccttctcagcagaagatgctggaggggactctga

ctggttctaaaggagagcacttggattctaatcccagaaccagcacccactctctaccacactctctgagtctccatcagtaacatgtggg---------

----------------------------------------------------------------------------------------------------

-------TGATAATGTGTTACAAACAGGATCACAGAaaaacctagtttcccttccagatgtggaagaaagTCTCTTGGAAAACTCCCTTCTCTACCAGCT

GGAGTTGGAGaagaagatgggccaca--GCTCCTCCATTCTTGAGCCAGTCCAGGGT-TCTGATTCAGTTCAAGGGATTCTGCCAATGCCCCAGATGTCC

TGATACATCTGTCAGCCAATGGTTTGTTGACACCTGTTcatgggACTCACTGACCTTCATTGCAGATCCATCAGCcctgcttcctcagatctgac-----

-----------------CACCAGTGAGGAAAGAAATCTCAGATTGTCGTGGATGCCAagag-CAGAGGAGGAGTTAGGTGAAAACTGATGAGAACT-GCT

ACACAAC-----A--AGT--CCTC-TGATGCCAGGCATATTTTAAGCAGAGGCTCTGGAGGAAGA-GAAGCAAGGGGAGGGGGCATCCATGTGCTCAA--

CCCAAATCA-TCCACTCACGTGCGCCTTCCAGGCCCTCTGTATTC-TTTTCAGGAAATCTTGAATCCATATTTTATTCCAGCCAAGcatgggattccttA

CTTCTCGCATCATGAAAGCCAGCATAAAGCACTGGCAATGCTCACGCTATGTTTTAGGAGGCAACGCCAACTGTAATGCATCCATGTCCTGTTGCTGCCA

ACAGTCTGTTCCAGgaaattgagaaTTTTTCTATCGTGTACAGAATTCTTCCCATCCCTGCTCATtccccaagggcaaagccatttctaaatttttacat

gtttatgatattactgcacttctagatagatagctgagttTGTATTTGTTTCTGATTGCTATTGTAACAAATgactgtaaactcagagacttaaaacaac

acagaagtagtatctacagttctggaggtgagaaatctctaataggtcagtaggctgcattccttcaggcaggtttgcaggatgattccttttcttgcct

tttccaacttataaaagttgccttgc----ATTCCTTAGCTCAGGGTCCCTTCCTGTATCCTGAAAttttCTCTCTCTCTCTCTCTCTCTCTCttgcggg

cgcgcgcgctccctacttcctctctgattctacacccatATCTTGTCTCCTACTCTGTTCTTCTTGCCTTCATTTTAAcccttttgatttttattgagct

caccat------------------GATAACATCCCCACCTGGAGATCTTTAAATAAATCACATctacgaaattcctttggccatattatgtaaaatgcac

acattccagggactgtgtgttaca----------------------------------------------------------------------------

----------------------------------------------------------------------------------------------------

----------------------------------------------------------------------------------------------------

-------------------------------------ATGTGGACATCTTTGGGAGGGtgcattattcagactacagctagagacaagccaaaatcttat

gttagttatctgggcactgcagcctt----GCCTTTGAAATCTGGATCTGGCTTGGTGACTTAATTctgatgatcctgtgcaagtcacctttcatttgca

gctccataacttacagacttgcattgtgcatcaa--------------------------------AATCACCAGAGGTGCCTATAAAACACCAGAACAT

CCcagcaattctaatgtaggattttagaatgggggaagg---TCCCCTTCCCATTGGTATCTTGTATGAGATCCCCAGAAGAtc----CTGGTACACAGA

TGAATTTGGGACCCTGTGCTCtgtgtTTCTCTCATGTTCTTCTTATTGGAGTTCACAGCCAGTCCTgc-AATCTCTGGCTTGGGTTTTCATGGTGTCTTT

TGctgTGACCTAGACTTGGCCAATTTGGAATCAGGAATGAAAG---------AGTTCAGAAATTTAGGACCTTATGGAGTACAAAATGCCAGATGTTTTA

TCATCCCA---AG--GAAT----TTTTTATAAAGGCTTTGAATGATAGAGTATCCATAAAACattttaggtggacag----------------------G

TAAAAAACATGGCATTCAAACCACTGAAGCCAGAGAGCCTCTGTCTCAGAGCCAGCTGTGAGTCAGATGTAGAAGAGGCAGAGAAATtagtcaggagcta

catttagggagggttttgacatagcatttctggtagaccaggaaaatgctactagcttctgaaggagc-TATGCTGCCAAGAAAGTCAGAAGCCAAGA--

--GAAAGGCTTTGGTCGTTCTGGCTATAAAACAACTTCCGAATCTCCCATAAGCCAGaGGTG-GTGATGAGGGAGGCC-AG---GGACAGCAAGTCCGC-

-AATTCCACTTCCAGGGCAGCCAAGGCGAAGAAATGGTGAAGCAAGAGTCCCCCAGAgg--GGTCCCAGGGGCTCTGGGAAAGGAAGCTCC-CCTGCACC

CAGAGCAGAATAAAACCCCT-------TTCCAGAAAAGGGCTCTCCCTAAACCTACCCAGGACTGTATCAGGACAGCTGTGCATTGGTCGCTGCTGC-TG

GTTCCCAGTCTGGCCTTTG-CCAGTGACAGCGTTTACTGCAATTGCCTAATTGCTGTTTCACCTTGTGGATGGGTGTGTCAG--GAGCAGAC---TT-TT

--TTTTTTAAGC-TATCATCAGTCTTTTATTCAAAAGATATAAAATTCACTTTTATAAGCCTTCATTATCT--TTTTTAAGCTTTTCAATTTTTAAAAAT

TGTGGCTAAAAAA---AGAACATAAAATTGACTATATCTATC-TTTTTAAGGTGTA-ACTCTGGAGTT-CTGGTGTTAATAAACTTCACATTCTTGTGCA

T---------------------------------------------------------------------------------------------------

----------------------------------------------------------------------------------------------------

----------------------------------------------------------------------------------------------------

----------------------------------------------------------------------------------------------------

----------------------------------------------------------------------------------------------------

----------------------------------------------------------------------------------------------------

----------------------------------------------------------------------------------------------------

----------------------------------------------------------------------------------------------------

----------------------------------------------------------------------------------------------------

---------------------------------------------------------TGGGTCTCCAG-A---ACCT--CTTCA-T-CTT--GCAAAAC-

--CCTGTA---CCCA---TCAAACGAT-T--CT-CC--A-TTTCC-----TGCT----TTGC---C--AAACCCCTG-GA--AACCC---ACTCTTC-TG

TTTTCTT-TGTT-----CT-ATCAGTTTCAT---TTG---G-ATC--CCCTAA----GTGGG-TG------CA----G--G---G-GCCC----------

--AAGC-----AGTT-GG-G-----CCATCT-TCTGCT---GCTTT-----CCTA----------GTTACATTA--------------------------

------------GCAG-GG---A-GCT---------AG-A-T-TGGAAGT----GG--AGCAG--CA---GGGA-CA------CGA-ACCAG---AGCC-

-CATA-TGA-G----AT----GCTAGTGTCACAGG--CAGC--GAC-T--T----AACA--TGTTATGCCACAAC-ACT--GGTCCCT----------AG

T--TTTG--CTT------TTG---TTGCA-TGTGCT----T--T----TTGTA-TC-A---TGTACAAA-----T---AT---A---CA-----------

TTACCAAATCTAATGTTATGAAGGTTTTTCCCTATGTTTCTTCTTGGGAGTTATAGTTTAAGATCTTGCATTTAGATCTTTGACCAATTTTGAGTTAAAT

TTCGTATCTGGTGTAAGATAAAGGTACAGT----------------------------------------------------------------------

----------------------------------------------------------------------------------------------------

----------------------------------------------------------------------------------------------------

----------------------------------TTCAATCTTTGCACTTGGATATCCAGTCTTTCCAACATaatctgtggaagatactgt---------

-------------------CTTTTCAAAGATCGTTTGACCATAcgtataacggttttttttatcttttggctctcctattctatctttcctttctatcct

attaacctatctttatactaatagcatagtagcttcattaccatatatTTGTAATCTATTTTCAAATCAGGAAGTGTGAGACCTTCtagcttaagcttta

tcaagatta----TTTTGACTATTTGGGGTACCTTGATATTCTAGACAAATTTtaagatggaatctttttacttctgcaaaagttgtcattggaattttt

ttttttttttgataggcagagtggacagtgagagagagagacagagagagaaaggtcttccttttgccgttggttcaccctccaatggccgcggctgcag

ccggcgcaccgcgctgatccgatagcaggagccaggatccaggtgcttttcctggtctcccatggggtgcagggcccaagcacctgggccatcctccact

gcactccctggccatagcagagagctggcctggaagaggggcaaccgggacagaatccggcgccccgaccgggactagaacccggtgtgccggtgccgca

aggtggaggattagcctattgagccacggcgccggctcgtcattggaatttttatataAGGATTGCACTGACTCTATAGATCACTTttaaaatttttatg

ttttgaagaggcagagatagagaaagacagtgagacagagaagaagggagagggacagggagagggagaagaagagagaggaagagggagcgagagacag

agatcttctgggtgactcctcaaatgcaacagctgagggcaggctgagctgaagctggtagctgggtattcagtctaggtctcccacatgggaggtacca

actgaattacttgagccatctcactgcctcccaaagcctgtacttgcaggaagctggagtcaggaaccagagccagtgaccaaatccaggtattctgata

cgggaatcagacatcttaaccagcatcttgacttctaggctaaatgcctgtccctgaagattgctttaagtactattaacAACTTACCAATATTAAGCCA

TCCAATCCATAAACATtttcttttcagattttttcactgtaagtgtatagaaatacaattattgattttgtatactgt----------------------

----------------------------------------------------------------------------------------------------

---------------------------------------TACTTTGCTACATTCTTTTATTAGTTGTAACAGGTATTTTaggggtac--AATCTTCAGGG

TTTCCTACAAATAAGGACACATCActtacagaaagatagttttatgccttcctctttgatgtgggtggtttttatttattcctccttgtttgttgcagct

aggacttttaggactgggttgactagaaatggcaagagcaggcattcttgtcttagaaatagtgcctttagtcttgcccagttgagtagctgtcagCTGT

GGGTTTCTCAAACATCGTCTTTATTAAATTGAGATAgtgtccttctatttccaaaaatacttttctcattTGTTGAGTATTCTTATCATGAAAAGATGTT

GAATaTTTTCAAATGCTTTTTTAGCATCAATTGAGATAATAGTGTgacttttgtccctcatctgtcaatgtggttgattatatttactgattcctgtg-T

GTTCAACCATCCTTCTATTCCAGGAATAAATCtacttaatacaa-GTACATAATCCTTTAAATGTGCTGTTGAATTCAGTTTGCTaataTTTTGTTGAGG

GTTTTTGCATCAATATTCATTATGAATATttgtctatag-TTTCCTTTTTTGTAGTGTCTTTGAATGGCTTTGATATCAGgctgatcctaGTCTCATAGA

GTGAGTTTGAAAGTCTTTACCCCTCTTCAAta-CTTGGAAGAATTTGAGGAGGGTCAGTGTTAATTCTTCTTTagatgtctgaTAAAATTCTCCAGTAAA

GCCATCTGATACTGGGCTTTTCTctgttgggagacatttgttttaaatttttatttacttgaaagacagagagatagagactgagaccaagatggacaaa

gagagatttttcatcaatctactggttcattccccaaatgtctacaaaaggcaaaattgggccgggttgaagccaggagccttgaattcaagccagatct

cctacaagggtggcagggacccatctatgttagccatcacctgctgcctcccagggtgcacattagcaggaagccagaatcagaaacagagcaaaggcgc

caacccaagcactccatataggatacgaactaaccaattggtatcttaactgctgcaccaagcgcctactcttgttgggaggcttttgactaggaatata

atttcctcACTAGCTATAGATCTGTTCAGATTTTCTGTTTCTTCATgattgtgtattactaggattttacttgttttccccttaggttatccagtatact

caCATGTAATTATTCAGAGTACTGTCTTATAATCCTTTcttctatga---CATTAATTGTAATGTCCCCTCTTTTATTTCATATTTTAGTgatctgagcc

ttctttttctcttcagttaatctaagagtttgttgatgatttcaacaaaccaactctcaatgtcattgattgctttttctatttttaaaaatgccctgct

taattgatctctgctctagttttgttttcttccttctacagctagttttgggtttacTTAGTTTGTTCTTTTTCTAGTTTCtcaagtgttata--TTAGA

GTGCTGATTTGAGATCTTTCTTCTTTTTTAATtaaacatttatcc-CTATCAACTTCCCTCTTCACATTGCTTTCTCTACATCCCAgggttttggagtgg

tgtgtttttcagatttttatttttgggCAAATTTTAATCTCACAGAAAAGTTGCAAAAATAGTACAGtgagaatccatatatatttcactcagctcctct

aatgttagaatcctacttgctccatgtcctgg--AACTGTGCTGTGTCTCTTGCCCATGGGTCTCCTCCATTATaagacccaagctgAAAGAGCACTCTC

ATCTGGGTCCTGCCATCCTatagttaagaggaagggcagccatggtgagaggctactagtttctgttcagagacagcacttgtcactgtatctcacatct

gattgtccaaagctagagtgctggtg--ACACAGAGAATGTTCTCCCTGGGCTGAGTGGTCAAAATtccatcaagcCCCAAAATACTTCACATCTGGAGA

TCTCCCAGAACTTCTAgaaactcttctctcctccattactgagatactaaccttccacctttatgtccagcgtagtacttcctggaagccccccacttcg

-----ATGTCTGTTTTGAACAAACAGGACAGACTTAGaatc-ACTTGTTCAGAAGGGTACCAGCATCAACCTGAGTCCCTCCccagtttgtccactactc

aacttttttgtttttaaaaggtttatttatttgaaagacagagtaacagaaagagagggagagatcctccatctgctggttcactctccaaatggctgca

atggccagggctggagtagactgaaaccaagagacaggaactccatcctggtttcccacatgggtggcggggactgtcatacttgagccatcatctactg

cttcccaggcacattagcaggaacctggatcagaagcggagcaactgtgacttgaagcagcactgcaatatggaatgccgatatcacaagccctgaccta

actagctgtgccacaacgatgtgctctgtatgccttagccttgctcctggggggctggaagctagccagtgtaaattatctaacgtaatagagtacaaat

aaatgcaaagtgtatgaacaaaattgacattttaagattcactgattggtttacagcccttgtctctactgttgaggaacagtgttatttctgcttacta

tttgctgctgaatcctttacttagtgtaagggtaatcttatgagtataaaataaactgaaagtagatgattgtaaaaattaaaagaataagaaaggaagg

agagtggcagcatgggcgggagggagggtagggtgggaagtatcactttaaaactaaataattaattt--------------------------------

----------------------------------------------------------------------------------------------------

----------------------------------------------------------------------------------------------------

----------------------------------------------------------------------------------------------------

----------------------------------------------------------------------------------------------------

----------------------------------------------------------------------------------------------------

----------------------------------------------------------------------------------------------------

----------------------------------------------------------------------------------------------------

----------------------------------------------------------------------------------------------------

----------------------------------------------------------------------------------------------------

----------------------------------------------------------------------------------------------------

----------------------------------------------------------------------------------------------------

----------------------------------------------------------------------------------------------------

----------------------------------------------------------------------------------------------------

----------------------------------------------------------------------------------------------------

----------------------------------------------------------------------------------------------------

----------------------------------------------------------------------------------------------------

----------------------------------------------------------------------------------------------------

----------------------------------------------------------------------------------------------------

----------------------------------------------------------------------------------------------------

----------------------------------------------------------------------------------------------------

----------------------------------------------------------------------------------------------------

----------------------------------------------------------------------------------------------------

----------------------------------------------------------------------------------------------------

----------------------------------------------------------------------------------------------------

---------------------------------AAAAAACTAACCATGTGGATGAACCCCTGAAGAGGTtggtgatgaggccagg---------------

----------AGAGAGAGAGAGAGAgagagagagagagagagagaggagagcttCATCTGGTCCAACCCCTTGCTGTTCCTGTCCTC--AGCCCAGACAT

CAGACATTAAGCAAAGGCTTTGCGATgatcccaatcttaaccatcatccaactgcaaagataggaaagaccttgagcaaatgccatctagctgaagccag

tcaaccctcgtaattcagagcaaagtaaacggtcgctattgatgcAAGACACCTGTTTCTAATGGGTTGTTATGCAGAAATAGATgaatagAAAAGACCC

TACAGTTCCAGGTAGTATTATTCTAgtgccgaatcctgtca---TTCTGCTCACGTTCTGTTACTCTATTTTTCTC--AATTTGAGACAGCTGCCGGAAC

TTAAAGAGCGTGAAGCCTCTCTCGATGCACTTT--AGAGATCAGTTGGA-TTTCAGTGCCTCCTTCTGCACTctggggtcctggggatattgcccgcaag

gaaaagtgaggacATCTCTGACGTCTCCTTGGCTTCAGTGTCCCTGCTTCCTTccgcTTTTCTAAAGAAAGTGTTCCATTTCAAAGAAGTTGAGCTCata

acacagccgtggagcgctgtgctctgcccggacactgc-----CCCTGCCTCACCAGCCTGTTCTGGTCTCAGAGGGAAAACAgctcactccattcacac

ccacactttccttctcttttctaggcagggggccagGCTCTGGCTCAGCAGATTTAggactgtatcctgtgattcattcagaatttgcaaagctctcttg

ccccacccctcatcctttt----gcaTATAATAGGCAGAGGCATAGCCTCCCCAGGAGCAGAGAGGCTGAGgccagcacaggaacctgcagctctccc-t

ccaagctcgcctcctcgcgctccagc---atgcaaatCTCTGCAGCACTTCTGTGCCTGCTGCTCACAGTGGCTGCcTTCAGCTCCCAGGTGCTTGCCCA

GCCAG-GTGAGGTCCCcccctcctccccgtgagacacagatctctccatctcctctccaggtgttcagggactgccacagcgggaggcgtcCCCACAGTC

TCACTTGAACAGCTGCTTTTCCAAGCTAAGGtagctcatgggccaggagggagcccagtcacaccgcagctcc------GGGCAGAGCCTGAGCTCCAAT

TCCAGCGGTGAACCCCAcatctggctcc-TCTGGGTTTCCAACACTGGAAACACCCTCAGgcagtcgccacctagctgctgtcagtagggttcagagttg

ggggtgatctaagaggggaccgcaggtggcgggtgtgcagggcacttccaagatatggaagggaggagaggagacccagaatgctctgtgctttagttgg

cccagcttctgcagaggcagtgggcacaggggtccccaactgctattattctctggccctgatcattggctcatgtgcccctcctatgacctcttcagaa

tcaccttgtttgaacagaaccttcttctagcccagcctccagcaaagtagctctgcagagacaatggatttttggagcctaaaagaggcatgtaactggg

gtctccctgcccccagggcattgcattattggacgacccttccTCTATCAGCTGGTGATGCTTCATGCCCCACTTATAGTCCAtgagagtgaGGGTTTAA

GGAAAGATCTCAAAGAGCAGAGACATTGAAGCCGGCGCCGTGGCTCAATAGGCTAATCCTCCACCTTGCGGCGCCGGCACACCGGGTTCTAGTCCCGGTT

GGGGCGCCGGATTCTGTCCCGGTTGCCCCTCTTCCAGGCCAGCTCTCTGCTATGGCCAGGGAGTGCAGTGGAGGATGGCCCAGGTGCTTGGGCCCTGCAC

CCCATGGGAGACCAGGAAAAGCACCTGGCTCCTGGCTCCTGCCAGGATCAGCGCGGTGCGCCGGCTGCAGCGGCGGCCATTGGAGGGTGAACCAACGGCA

AAGGAAGACCTTTCTCTCTCTGTCTCTCTCTCTCACTGTCCACTCTGCCTGTCAAAAAAAAAAAAAAAAAAAAACTAATTAAAATTTAAAATTACAAAAA

AAAAAAGAGCAGAGACATTGAACTTGGGGTGGGCAGCTTCTCCccctccgtctccctttctctcccgttctgccttccaactATTCCCTCCTTGACAAAT

AAGGAGACCCAGAGAACACCCTgaagagactgc--------TTGTTTCATTGCAG-AAGGGACTAAcagcggcaaaACCTGCTGCTACAGATTccacaac

aggaggatggacccgcagaagctgaggagctacacactcatcagcatcAGCTACTGTCCCCGGGAAGCTGTGAT-GTGAGTGGACCATGgccagcaccct

cagcccgcagtcctgagctctcctctagggtaacagagaggaccacggtgcccagcctgggtagagacactctcagccccggaagctttccggtgggaaa

gct----------------------gaagccaagc-aaaaacatggcttcctgcttcctgcttccagcttccttgctggtcttcagttcagagctcagtt

ccttcatcccagagcccaggagacttcacccaggccgacgagagcgctgcttcccctgagg-CTCTTCCTTTGTCTTGTCTTCCTCCTCCTCCACTGGGG

TAgccccacca------------GCCCAGACAGGTTTCCCACAGCATAGGTTACACCCCTCGGttaaatcctcttaagcacattgtctaaccgtcctttc

tcc--CCACAG-CTTCAAGACCAAACAGCACCGAGAGGTCTGTGCTgaccccaagtggccatgggcccaaaatgccattgcctACCTGAACAAGAAAACT

CAGACTTCAAAGCCTTGA---GCAgTCTTGCCTGCATTAAATCCAAGCCTGGATTTGAGAAGCAAgtaacctgtgtccactcgcttcaactcaagagttg

tgcagagattatcttgttgTAATTCTAAGAAATAGGAGCTTTGTGTAGTCGTGTGAATCacagttttccttaaatatttttaagttattagcaccttaat

ttaacttgcactggcatgggggaggagtttgaactgtaaagccttgtacatactgtgccattttattatagaattgattattttatatgtgttattcttg

c---------------------------------------------------------------------------------------------------

--------------------------------ATTTTTACATAAAATATATTTTTGTAAAAaacttgactttgatgactttttaaaagatgaaacggaac

tgaagtgtccc-----CAGAGTGGCTGGGAGAGGGGCATAGTTGCAGAGTGTGCGgctggtattgaggagggacaagcataagacagaccctgggcttcg

tcccaggcaggccggtgccaagctgagcccgctgtgcggccccacgtgca--------------------------------ACATGAGCTGCAGACACT

TGTGCAAGGACCTCTCTTTCTTtgcaaagttattca-----TCACCATCACATACCCTACCCCAAAGTGTCTGCCTTCATCcattatgtcaacctcatgg

ctggttagtgtttctcttcatcaatcctctaatccagagtaatgcaaataccatctgcactgagaaaccaggaaagaacccacagatgattctgcaactc

ccttacggagcagaaaaccaggagtctttaaaaaaaaaatgtcggccggcgccgcggctcactaggctaatcctccgcctagcggcgccggcacaccggg

ttctagtcccggtcggggcgccggattctgtcccggttgcccctctttcaggccagccctctgctgtggccagggagtgcagtggaggatggcccaggtg

cttgggccctgcaccccatgggagaccaggaaaagcacctggctcctggctcctgccatcggatcagcgcggtgcgctagccgcagcgcgccggccgcga

cggccattggagggtgaaccaacggcaaaggaagacctttctctctgtctctctctctctcactgtccactctgcctgtcaaaaaaataataataataaa

aaaaaatgtccatcttgacaagttagggaagaaggaggaaggtgtggatcctgcactcaatgtgcgaccctgacaaaccgttgatagcttcaggctatct

tcttagagacagaatttccatcaaagccctaggtagacttctccagatgtcaagaggaatcagacacgtttgtggcaagcagacaatttactggtgatca

tttagtgccCAGGCTGTGTCCAACAGTATTTTCTGCCCCTGCTTCTGCCT-TTGGTCTCTGTCCTCACACACCA--GT-------GAA----GAGCTT--

---AAT-----------CTCAG---ATT-----ACAGAG----AGAACAA--A--TGGGAA-GCA--GAC-----ATG-CCAGGA--GT---AGT-A-GT

AGG---GGATATGAGT---A--CACGCTGCA----TCTTAATG--AGATGCAG--CAAGC-CCAC-----------------------------------

----------------------------------------------------------------------------------------------------

----------------------------------------------------------------------------------------------------

----------------------------------------------------------------------------------------------------

-----------------------------TGATGCAGGCCAGCATCAAGCAGAGTATCAGAGAGTAGGAc----AAGGAGAGAAGAGCTCCATGTGCCTA

AGCACCAACAGAATtGGCTCCAGTTATCTTTGTCAAGCTGTGTCTCTTTGTTCTCAAGGAATTTTAAACTTATTTGTCCTTAGGCAGGCCtaggaccctt

ggTCCCCTATAGGCATGGAGAATTAAAGAGAAATCCCAAACCACAACAGG--AGT-----ACTGGGCACCAAGTTCAATGGGATTCACAGATTAGccagg

aacaccact-AATGTGCCTGGTAAACTTGGGCAAGTGACTTCACCTGACTGGGCCTGCGTGGTAGACCAGCAGCTCTCATACTTGTGCCAAC-A-TC--A

CCCAAACACCAAAAAACCCCACAGATGTCCAGAATAATACAAACACTACCTGCGTGGAGAAGTTAGGTAGAGGACTGGCAGGTGACCCAACACGTTCCCT

TCCAAAGGTTCTGATAGGGAGTATTGTGTGGGGA-GAGGCCCCTTCCTCCTTTTTCACTGGTCCTCAGCTGTGTCT-GGGACCAGTCACAGGATAGACTT

AGAGGCACCCTGATTTTCTCGCCTGCGGACTAGTGTAGTTTTTTAACTTGGCCTGGGCCCTCTGGCTTGCACACAAAGAGTGACTTCTGTTTAAACTTTC

TCGTCTCTGAAGCTTGGCTGTTGGACATGcactagccctgAAGTAGCACAAACCGTAAATGTCCATCACATAgccaTGAGCCCCTCTACATTCCCCAGAC

TAAATTGCATTTtctg-GGGGCCATGTGGCTGAATACTAACCAATTAGGTGAAGGTGAAGTTTGAAGTAATGAAAGCCTCTTCCAG-CCATGACACCTAT

AAATAACTCACACTTCCAATCTTTTCTTCCACTAACAGTAACTTTGGGGACACTC--TTCTATAAAGCTCAACTTCAAGGTGGAAGCAGTCCATATCctg

aagtcagtggtcacTACTTGGAGGACAGCTATCTGGTCTGTCTTCAACTGTGACA-GAGATA--AATAAG--TCTGTTGTGTTAAGCCACTGATGC----

-TTGGAGCCCAGTTTAATCCCAATTAATAGAAGGATTgataccataatcat-TGCTACCATTAAAAACCAGAAGAG-G-GGC-----C-AG-TGCT--GT

GGCA--CAGTG-GG---TTAACACCATGGCCTGAAG-------------C--GCCACA-----TC---CC----A-----TAT--G--G-----GCA---

CC--GG------TTC----AAG-T-C--CAG--GC-TGCT--CCACTTCTGATCCAGCTCTCTGCTGTGGC-CTGGGAAAGCAAT------AGAAGATGA

C-CCAA---GTCCTCAGGCCCC--TG-----CA------CCCACA--T--GGGA----A-ACCG--GGAAGAAGCT-CCTGCTCCTGG-TTTCAGAT-C-

--GGCATAGC-TCT--GGCTG--------TT-GCAGCCAA--T---TGGGGAGTGAA------C-C--AGC-GAATGGAA-GACCT----C-----TC--

-TC----TC-T-GCCTCTCCTCTC---TCTGTGTAACTCTGACTTTCAAATAAATAAA-ACAATCTTAA--AAAAAAAGA--GAGA--GAGA--GAG---

----------------------------------------------------------------------------------------------------

----------------------------------------------------------------------------------------------------

----------------------------------------------------------------------------------------------------

----------------------------------------------------------------------------------------------------

----------------------------------------------------------------------------------------------------

----------------------------------------------------------------------------------------------------

----------------------------------------------------------------------------------------------------

----------------------------------------------------------------------------------------------------

----------------------------------------------------------------------------------------------------

----------------------------------------------------------------------------------------------------

----------------------------------------------------------------------------------------------------

----------------------------------------------------------------------------------------------------

----------------------------------------------------------------------------------------------------

----------------------------------------------------------------------------------------------------

----------------------------------------------------------------------------------------------------

----------------------------------------------------------------------------------------------------

----------------------------------------------------------------------------------------------------

----------------------------------------------------------------------------------------------------

----------------------------------------------------------------------------------------------------

----------------------------------------------------------------------------------------------------

----------------------------------------------------------------------------------------------------

----------------------------------------------------------------------------------------------------

----------------------------------------------------------------------------------------------------

--------------------------AGAGAGAGTTGGGATAAAATAAATGTCACTGTGTCagtgtctctttaaagcctatctaggggccggtactgtgg

cgtagtgggtaaagctgccgcctgcagtgccggcatcccatatgggcaccggtttgagtcccagctgctccacttcctatccagctctctgctgtggcct

gggaaagcagtagaagtggcccaagtccttgggcccctgtacccacatgggagacctggaagaagctcctggctccacaggctttggattggcacagctc

tggctgttgcaaccaattgggaatgaaccattggatggaagacctctctctctctctctgtcttgccttctatctctgtgtaactctgactttcaagt--

----------------------------------------------------------------------------------------------------

----------------------------------------------------------------------------------------------------

----------------------------------------------------------------------------------------------------

----------------------------------------------------------------------------------------------------

----------------------------------------------------------------------------------------------------

----------------------------------------------------------------------------------------------------

----------------------------------------------------------------------------------------------------

----------------------------------------------------------------------------------------------------

----------------------------------------------------------------------------------------------------

----------------------------------------------------------------------------------------------------

----------------------------------------------------------------------------------------------------

----------------------------------------------------------------------------------------------------

----------------------------------------------------------------------------------------------------

----------------------------------------------------------------------------------------------------

----------------------------------------------------------------------------------------------------

----------------------------------------------------------------------------------------------------

----------------------------------------------------------------------------------------------------

-----------------------------------------------------------------------AAAATAAATAAATCTTTAAAAAAAAATAA

AGCctctctaatgctctacagctttataaaaaatctggcgcagagttagaaaataaagccaggagccctgacccctggcatctgtcctaccgacccctca

ctgttccttgg-----------------------------------------------------------------------------------------

----------------------------------------------------------------------------------------------------

----------------------------------------------------------------------------------------------------

----------------------------------------------------------------------------------------------------

----------------------------------------------------------------------------------------------------

----------------------------------------------------------------------------------------------------

----------------------------------------------------------------------------------------------------

----------------------------------------------------------------------------------------------------

----------------------------------------------------------------------------------------------------

----------------------------------------------------------------------------------------------------

----------------------------------------------------------------------------------------------------

----------------------------------------------------------------------------------------------------

----------------------------------------------------------------------------------------------------

----------------------------------------------------------------------------------------------------

----------------------------------------------------------------------------------------------------

----------------------------------------------------------------------------------------------------

----------------------------------------------------------------------------------------------------

----------------------------------------------------------------------------------------------------

----------------------------------------------------------------------------------------------------

----------------------------------------------------------------------------------------------------

----------------------------------------------------------------------------------------------------

----------------------------------------------------------------------------------------------------

----------------------------------------------------------------------------------------------------

----------------------------------------------------------------------------------------------------

----------------------------------------------------------------------------------------------------

----------------------------------------------------------------------------------------------------

----------------------------------------------------------------------------------------------------

----------------------------------------------------------------------------------------------------

----------------------------------------------------------------------------------------------------

----------------------------------------------------------------------------------------------------

----------------------------------------------------------------------------------------------------

----------------------------------------------------------------------------------------------------

----------------------------------------------------------------------------------------------------

----------------------------------------------------------------------------------------------------

----------------------------------------------------------------------------------------------------

----------------------------------------------------------------------------------------------------

----------------------------------------------------------------------------------------------------

---------------------------------------------------AAAATGCTTAAGAAGCCAAGATTTTGTTTTCATAAtagaGGACTCATTT

TCTGCCCATCTTTCAGGGTCCTTCCTacg-AAGTCTCCCCATTCATCAGGTGTCAAATCACAGGCtgctttaggtcatgctgggatgtgagcttccaagg

aaaacaggaaaagtgagccagcaggattccctcactcgcccaagagagccaacaactggatggatagaaccgttgcttct---------CTTGGATGTCT

GAAGATTTATTTTATTTGT--CC-CTG-----CTGAGATTTACTGGGTTTGAAATAATTGTGAAG-----TGA-----GGG----------AGGCAA-CA

CAATAAATG--TAGTCATTAGCTAATAGGGCCCACTCAACTTCCTGC-AGGACTGTCAGAGTATTTCCTA-TGCAGGAAACA-TCACTCAACTAAGGCAG

CCAAGGCACCT-TGAGGTCAAGAGCATTCCTTCCGAATGTCTTGCATTGTCAAGG--TCTCTGAGATTTCACCTCTCCCATGTGTGTAAC-CAGAGAATT

CCTGGAAACTGTCTCCAGAGTAGCCGATCTTTACTTGGAATCA-TGGGGAA-TTTGGGAGGATCGGCAGAGGAAGTCATCTTCCCAGA-TGTGGGGTGTC

TTTCTAGCATCTCCTGGTCCACAGAAATGTGCCACATTTCCTAATAATGACA-AGTATTA-TCT-T---CCAT-TTGCTGTGTACATACGG-A-GATCTG

GTCACTC-TTCTAAGCACTGTATATATTATTTTTTAAG-ACTTATTTATTTATTAGAAAGAGTTACACAGAG-AGAGGAGACGAAGAGAGAATCTTCCAT

CCGTTGATTCACTCCCCAGTTGGCCGCAATGGCTGGAGTTGTGCCAATCCGAAGCCAGGAGCCAGGAGCTTCTTCCAGGTCTCCCACGCGGGTGCAGAGG

CCCAAGGACTTGGGCCAACTTCCACTGCTTTCCCAGGCCACAGCAGAGAGCTGG-ATCAGAAGTG-GAGCCACTGGGTCTCAAACCGGCGCCC--ATA-T

GGAATGTCGGCA-CTTCAGGCCA--GGGTGT-TAGACCACTGCACCACAG-CGCCGGCCCCCA--CTGTATATAT-TATTAACTCATTTAATTCACACCA

TGAGCCTCTAAGACAGGCATGATGATCATCC-ACATCTTAACCTAACATGGGATAAGAAAGTTAGTGAGAAAGCATGGTAAGAAAGTTGTCTGTGGTAGC

AGAGCTGGTCAGTGGCACAGCCATCACCCAAATTCAGGCATTCTAGGCCTAGAGTCTCCATCGGCCCACCTTACCTAGAGCTGAGCAGCAAAGAACAGGG

AAGGCAC-AGGTGCCTGGCC-CTACTTGGT--CCCT--TTGA------AGGTGATTAT-------T----CTGGATGAACACTCAGAGACCTTGCAGGCC

AGCAAGGGCACTCTTTAATCCTGTGCCCATGA--GACATGACCAGTCAACCATGACACTGCCTTCAACTAA-----GTC-----CTCAGAGTCCCTCTTA

ACACACCTGTCTGTGAAACTGTGCCAATCCATGAGTTTGATGTAGGAGTGTGCAGGAAGAGCCAGGAAACAACTATGTAATGCTTCCACAGCAGTAACCA

ATGTTGTAACTCACCCTAGTGAGTTAAGTGAAGGTTTTATCTCCTCTTGCACTCTCTCCTCCTAGGGGGCTTAAGTACTATGCACAAGGCGATACCTGTT

GGTTGTGGTGGC-CCTATCTCTT-GATGTTTTTAGCTATCAGACACTTTGCC----ATGTG-GTCCAG-GAGCTGAGGATA-AGCCAGATGGCTGATTTT

CCAAGAGT-TCTCCT---TG-GATCCTTGGGGTGTGAGGACACAGGGAGCCACTTGTTCTGCTACAAACAAACCCACTCCCCTAC-GTGTCATCAATGTA

TAGGAGCTTGTTTGGATTCTAATTGTAAAAGGGTGTTAGAAGTTAAGCAAATAGTTTAAAAGTGCacagGAAGTCAAGGTCAGTAGAAATTCTCCAGTTC

TGACATCCTgggatgcttaggttcccaggacactaaggttaaatcagaggggtaagcccctctttgctccccttcctggttctcagagttgaaaagcagt

gatgagtggctgag---------------CTCTCTGCCTCTGGGTGAGAGCCTTGCAGAAGGTTCAGGGcccctgcctggcctccca-ATTCT-GGG--C

AC--TGC---CGC-----T-GAGGAAGGTCTCTAGATCAGCTCACCTCAACATGCCCTGAGCTTCGCTTGCTTCCCTCCCCA-CAGCAGATGTTTTGTTT

------------TA----------TTTCAT------TTC----ATCACA-A-GCT--AC--CAC--CAT-----CAT-------TCCTACTCCAAGTC--

---CCAGAATAGATGAGAGAG-ACGCCAAGTCCCACCCACCATCTGGCTTC-CTTA-ATTCATATCT--CTTCCCAAGAACTGGAATTTGGTCAGCAATT

CAATACGCTCTCCACTCTCCACATCCCAATCTGATTTGATAGTGACTTT-ACTTAGG-AAAGACTTCCCTGGAAGAGCCCACCCTCTCTGCTCCCTATAA

A-GGCAGGCAGAGTGATCAAAAGTG-ACAGAGACAGAGATCAATCCAGAAGCCTCCAACTTGTGCTCCAC-GCTCATCTGCTT--GCCCTCAGC-ATGAA

GGTCTCTGCAGCACTTCTCTGCATGCTGGTCACGGCGGCTGTTTTCAGCTCCCAGGTGCTTGCTCAGCCAG-GTAAGGCCTT-GTCTCCCTA-AGAGAAA

-TC---CAACCACCTCTAAAACTCTCAGGTCAGCAAGTGTCTTCAGAGACTCACTGCAAACTCCGCACTGGGAAGG-GGGCAAGCAGATGCGGCAGGTGG

A---GAAATGGCCTGACCTTGCATCCAGTCAGTGGAGAAGCCAGGACCAGAAGCCTGGGCCATCTCAGTGGGTCCCCATGCGCTTGCTCTGACATGAGTA

TTGAGAGAGATGCTGGGTGCATTCGTG---GG-------TAGAGT--TTGGGGATGGGGCACAGAAATGAATGCCCTCCTCTTACTGTGAG-ATGG----

--------------GGAGAAAAGGAAAAACCTGTTGGC------------------------CTCAGGGACATCTTTCAGAGACAGTGATTAGGAGAAGg

tgattggatcctacaggaATCTCTGGAGGTGATTTATGTTGATTCTGCTTAGGAAAaaatatctaagtATTTGTACTATAACTCATTTTCC-AGCCTTTG

CTTCCTTCTGCCTATGGAGTGTGTCTG-CCCAA-GATGCTACAGCATGCTGTG-TTACAAACAATCCTAAATGATCAGTGGCTTCACACAAgaaatgAAA

TTAACACATGTTAATTTCTCACTTGTGCCAAATgatggtcATGTCTAGGCTTGGGTTTCTGCTTCACATACTCAGAATTCtgttctgatgcagctattgc

cca----CAGGAGCACTGCCAGTTACTGCAACTAAGGAAGAGAAAGCTCTTGAGGGTCTTGCATAGGCAACACACTCTCGCTCAAAAATGACACatgtct

ctctgtctcctcacttattgctcagtagtagttagctgcctgAACCAAACAAAAGTAGTCAAGAggaattagtttactgtgtgt--C----CAGGAAGCA

GAGAACA----GTG-------CCGTGGTTATCGCAGGGACACATAG--CT-------------GCTCGTA---ATCACATCAGCCAAGCTTCTTCCCTAC

CCTCACCGATGGTGATGGGTTCCCCCTGCACCAGGGAGCATGCCGG-GTCAGAGG-CAA-AGGC-CCAGACA--T----CT-CATTG--GATGGATTCTA

CCTCTACCTCTGACACATCCTCATTTTCTCCAAACTTCCTTCCTCCCTTCCAAAGCTGAGTTTG-AAATATCTGTGTATCATTA-GTAGCTCACCCTTT-

--CTG--CAAC-TTTTTCCCCCAAATCCAG-CTTTTGTCCCAACCACTTGCTGcttctctatggcc---aaGAAGATGCCCCTCCAGCGGCTGGAGAGCT

ACAGAAGAATCagtggcagcaaatgcCCCCAGAAAGCTGTGAT-GTAAGTAGACAAAGCTCACCCTCt-CCAGACCCAAGAGTCATTTCTAGGATGCCAA

GTGAAGAAGCATGTCAGAATTACTTGGAGTCATAGGCTCactcacTTTGACCTCAATAATCTAATTCATGAACACTCACAAtaggaaaTGTTCACTCTCA

GCTCCCTGTCTGGTATGACTGGTAGTGTGTTCAGATCATACCAGTTCCACTGAGGCAAGAGCAATGATGACTTTTCTGGGACATCAGGAGCAGCAGTCAG

TTCCCATCCCCTC-TCCAATCCCTTCTCTCCCGTAGCAGCCTCTTCCCCCATGGCAAATGGTTCTGGAAGATTTGAGGTTAAATGGACAGAATTTTCAAC

CAGGATCCTCCTAATTGCtttctttctctctccctgACAG---CTTCAAGACCAAGTTGGCCAAGGAGATCTGTGCtGACCCCAAGGAGAAGTGGGTCCA

AGATTCTATAAAATAcCTGGACCAAAAATCCAAAACTTCAAAGCCATAg-------TCACCTATTCTGAGACCAAACTGAAGTTTGAGAAATGCTTCATT

TATTTTCC-TTCTCAAGATGCGTTCTGAGGGGCTGGAGCTGCAGCACAGTGAGCTAAGCCTCACCTGTGGCATCCAATATGGGTGCCAGTTCATGTCCCG

GCTGCTCCTCTTTCCATCCAGCTCTCTGCTTATGGGCTGGGAAAGCAATGGAAGATGTCCCAAGTGCTTGGGCTCCTGCACCCATGTGGGAGACCTGGAA

GAAGTTCCTGGCTTTGGACTGACCCAGCTCCAGCCATTGCAGACATCAAATGGACGACCTTTCTCTCTGTCTCTCCCTCTCTTTCTGTAATTCTGCCTCT

CAAATAAATAAATAAAATATTTTTTAAAGAAAAGATGCATTCTGAGATGACTCCATTATCATTCCAAAAGGaaaatgacttaatatttttaaatattatt

ttt-------------------------AAGTTATTGGAGTTCTTGAATGCATCTTCCATGAATATTAGTTGTCTTTAAAATATAAAGCTTTGagcatgt

gacttgtcttgtctGAGCCCCACTTCAACCCCCTGCCAAATGTGGGCAATGcagcacctctcc--ACCATCTCCCTGGAATCTT-TAAGGGTCCTGGGGA

AGAAAATCA-CATTATGATGTCTTGTCTTTTTAGGAAAGCTTTGTTCTTATGAACCTAAGATGTGATTCctaatgTTAAATGGAAGTAAATa---TTTTT

GGAATACGTAAAATAAATGCACATTTTATTAcaatcagtaATTTGTGTGTGTGTTGGTGGGGGGGTGGATCCAAA-TAAGG-GAGGGAGA-AAA-ATGTC

AGTGGGAATGAGAG--TC--CCAGGGAACTG---ACTGTGTTCCTCAGAAATGGCCCTTTGGCTTTGCTGACCTtcCCCAGGGCTCAGGGTGAAACTGAC

AGGACCAGAAAATCTACTGCAGCCTTCTGAGTCCCGCTCCCAGCTGTGCTTCCCAAGACTCCACATATTGGGGTCACTCAACCAGCAGGAGACTCACTGC

CATTCCTTTCCTCACCAGCCTCATGCTCATCCCGAGTCTCTCCCATGACCTCTTGCAACAGCTTCGAGGCCtTCTTCCTCTCCCAGACCTTACTTCTTTG

TCTTCCAACC-----TAA-CA--C-TGA-ATA-ATC---AATCGGAGGAGGGCTAGAGGACCCTCAGCAAGATGGTTAtataaaggCTTTAGACTCCAGC

AAGGTGGAACGTAAACTTGGTCATGGTGAATGTACAG-GTGGAGGGCCCCTGTTGGAGG-----CAG---------AGGATA--GGGC-TTGCTTGAGAA

AAATGCCTATCTGTGGTGATGATCTTGATGTACTTACCAAAT-GGATGGAAGCCATATTCAGATATGTCAGGGTCAAGGCATGTGTGGAAATTATAGCCT

TTATGTTGCAAGTGGTAGGGACTTGCAGGAGGAAGGTCAAGGCCAG-CTGCCAAGGTCAGTGGATTTAGTGCTCTATTACATAGCAATAGGAAGAGGTGA

ACCTATAACTCTTTATCACCCAAATTAGAAAG-TTTTGCAAAGTAACAGA-TGGCACGGTTGATAATTATTTTTGGAACTACAGATC-AAATTAACACTG

CCCCAGGCAAACTGGAGTGTGTGGTCACgccagtattaacaggcaccgaaagtttcaattcagtgggGAAGAGAAGTGCTGTGAC-----TA-GAAGTTC

TGCAGGAAGAGTAGCCAGGTGGCtgtgc-CTGGGTTGGGAGGGGAAGAGAGAAGCCATTAG-GCATTGTCACAGTGTACACAAGAGGCACCAGGGGAAGG

AACTAGAGCACTGGTGGGTGGGAAGGGGAGAGGGTAATGGTAAGAAAAGC--AGGCCCCAGCATGG--TG--AAATGGTGACAATTGTGGATG-GGGACA

AGTAAGATGGGTAC---CCAAACTCCACAAGGCTGCAATTACAAGGTAGCTGCCTCATCCCAGAAAActcacttcctctac-CACTCTCCCCTTATACCC

TATCTTTACAAGCTTGAGAGC-TGCCATAAAATACACAGGA-----TCCTCC-----AAGCCTCTAAAAGCAGCA--CCCTT-CAGCAGATGAAATCAAC

TTGAACTTCTGTCAACCTTGTGAGCCC-TGTACCCAGAAACAGAGAACAACTATGTCTCCAAGGGgcAGACAAGGGTCCTAGATGCCATACTGGTATAAC

TgtagaaagtctgaatctgcctATCTGGAGGGCTGATGTCCTACTGTGCCACTCTATGTCCAGCAGGGTCATTGCACAGCAGGTCTCCAAGAGG-ACTCA

TTCATACTAAACT---CTCTTTGGTCCACAATATGTGTTGGTCATGTCACAAGGCAA-ATAATTGGTAGCATGAAGCATTAGAAAGAGCACTGATATGGG

A--CAGGT-ACC--AGTTGTTGG-GTGGTCCCAGGTACCGATATGGGACAG-GTACCAGTTGTGG----GGTGGTCCCAGGTACTGATATGGG-ACA--G

GTACCAGTTGTGGGGTGGTCTCAGGACAGCTAGCTCATGGAGATCAC-------AAGA-CTCTGATTCCAGGAAA--ATC-CTGGAGAAGAGGTCAGTGA

AGGAGAGTAGAGGAGTGAG-GAATCTTTCATTTATGAAAGTCTAAG-CAGGCCGGCTCCATGGCTCAATAGGC-TAATC-CTCCACCTGCGGC-GCTGGC

TC-ACGGGGTTCTAGGCCTGGTTGGGGCGCTGGATTCTGTCCTGGTTGCTCCTCTTCTAGTCCAGCTCTCTGC-TGTGGCCCAGGAGTGCAGTGGAGGAT

GGCCCAAGTGCTTGGGCCCTGCACCC--A-CATGGGAGACCAGGATAAGTACCTGGCTCCTG--GTTT--CAGA--TCAGCACGGTGCCCCAGCCACAGT

GCACCGGCCATAG--CAGC-CATTGAGGGGTGAACCA-ATAGAAAAAGGAAGACCTTTCTC---TCTGTCT-CTCTC---TCTCACAGTCCACTCTGC-C

TATAAAAAAAAAAAAAGAAGAAGAAGAAAGAAA--------------GAAAGTCTGAGCAGAATTTGTTCACATCCCCTttgtcaaatattctctcttcc

ttttcaagaaatctggaactcaaattacctccaactggtaacttaaatgccttgGAGCATGAAACTGTGGGCACACAGCACTGAAGATATTTAAtcTTTC

TGATGGCACACATATCCCAACCAGGAAcCATGGATGTCACCAACTTATCTCTCTGGTAAGCAAACCCTGAAGTGGAAATGTTTGTGGGCAGTGT--AGTG

GAGAGTGTGGA-AGGCAGGGCTGAGCAGAGGCGGAACCTG-TATTGTGATACAGTTGCAGCCTAGGTCTCAGcCAATCCCACTGGGAGCTTTAGAACTGG

ataacc-CAAATAGGAATGAAGGGCCATGCTTTTAATCCCTCATCTcaccctatCATGACTGCAGACTGCCCCTGGAGCAGGACTTGACTCTGGctaagg

caggagacttctgtccagggcatGTCCCACAGAGAAAATTCAGCACAGAGTTCTTGGCTGCCTATCAGCACTGTATGATGACACAGCCACTGTCAGCAGT

TGGTGAAGGAGTCCTGGAGTCCTGGAGTCCTGAAAGGGAGAAATCTTGGCAGCATTCCA--AATTCCACCAA-ACCCACCCGTTGTGCTGCTGGAATTCA

CGTGTTTACGTAATGAGATCCAGAGAATGCGTCTAAGAGAAAGCCTCTCAGACTCTGTTGAGCTCTGTGATCT--GGAAA-TTGCAAGGAGAAAGTCACT

GGAGTGAATAACACCCCTCCCCCAGCTCCTACAGATGAACTTGAGGACATCAATGCTG-TCATCAACCC-CTTCTGGAA-ACCGATTTGTGACGATCCT-

GGCAT-AGC--AAGTCTATG--GGCACCA--T---TTGTCCAACAGCACGTACTCACTTCCT-GTCGCTGAGTGGCTAGTGTCACATGGTGGCTAGTGTC

ATAAT--TTT--GCACACCTTTGTA-TTATTATTTGTCATC--TTTGATGTTACAATTGGAATTGTTCTGAGGC-TCTA--TGAGTCAACGAACTGAATG

--AATGTGTATATTTTGACTG-TTCTACCAACTATCCCTTTTCCCATCTCTCTCCTTCT--CTGGCATTC-TTATTCT---CTGAA-AAT--TCTCTTAA

AGG--TAGGCCA--AGTTA-TAACCCTACAATGGCCTCTAACTGTTCCAGTG-AA--A-GGAAGAGG--TACACATCTCTCACTTTA--AATCAAAGGCT

GGACATGATTAAGTTTAATGATGAAGGAATATGAAAAGCTGATTGAGGCCCA------------------------------------------------

----------------------------------------------------------------------------------------------------

----------------------------------------------------------------------------------------------------

----------------------------------------------------------------------------------------------------

----------------------------------------------------------------------------------------------------

----------------------------------------------------------------------------------------------------

----------------------------------------------------------------------------------------------------

----------------------------------------------------------------------------------------------------

----------------------------------------------------------------------------------------------------

----------------------------------------------------------------------------------------------------

----------------------------------------------------------------------------------------------------

----------------------------------------------------------------------------------------------------

------------------------------------------AA---GC--TG---GGTCTCTTGTG-TCA-AACAG----CCAAGT-TG-TGATT--CA

AAGGCA---AAGTTATTGAAGGAAATTTAAA---ATGATCCTCCA--GCAG-ACA------CATG----A-ATG-GCCAAAA------AATGAAAT--AT

CCTTATTG---AT--GAG--GA----AAGTCTTAGTGGTC-TGAA-GA-G-----ATGAA---AC-----CA---GCC---A---CAACAT---------

TCCCTTAAGAA-ATACCTCATA-TG--A--GC-AAGCCACGAGCTGTCTCCAATCCTA------TGAAGG----CTGCA--GA-----A-GGTGAG-GAA

GTAGCAGA-AGAC--A-CGA-GGCTTAAGAA-G-----------CCAC---CTCTA-AACA------------TGA------AA-GA--GC------AA-

-GGTG--AGGCAG-----CA-AGT--T----ATCTGGA--------AGGGCTGGC--TGA-GACCAT-----CA-GTG--------C-AC-TAA--G-C-

------AAGAAAT-T-TGCA-ATGT-----A----------GATGCAATAG---A-------CTTATA--TTCG--GGA---------AGATGCT--AT-

CCAGAC-TTT--CAGA---GCAAGAAA-GAAA-------CCAATGCC--AGGCTTCCAAG-CAGCC-AAGGACAG--------GCTG---A----CTCTC

TGAG--GGTTAGGG---ACT----AA------C-------GC-A--GCA---GGTGACTTGAAGGTAAA-TCCAATGCC-AT--TTACCATTCAGAA---

-AAC-TCTAGGGA--C--CTAAGA-----A-------A-----AAC---TAAACCT--ACTCTGCCT-G---TG--CTCTGTA-AA--TG---GGAAAAA

AAACAA----AGCCTGGTTGATGCAAC-ATCCA---TTCATAATATGATTTACTGAATAG----TTTCAGCCCCACTG-TTG--------AGACCCACT-

--GCTTAAAAAA----AGGT---C--C--CATTCAT-A-----CTATCGCTGC-CCATTGA-CAGTG-CACC----TGGT--CA-C-CCAAGA-GCTCTG

---A-TGGAGA--TGTAG-A-A---TGAGGT----TAACGTGGTG--TAC-G---TGC-CT----GCT-A--AC--AC--AGC-ATCCA-----TTCTGC

AG--CCATG-GA---TTGAGG-AGCAATTTC-AACTTT-C-----AAGTC-TTATTA-TTTAA-GAAG--GACATTG----TGTGA--------GGATAT

TACTGCCA-----A-GATAGTGATTTTTC-------TGA---TGGA----TCT-----------GCA-C-------A-------AAGCAA-A--TTGAAA

ACCTTCTAGAA-AGG------A-GTCACC----------ATTC-TAGATGTC--ATTAA----GAA-CAGTCATGACTCATGGGAGAA-A---ATGA---

AA------AT--ATC-------AACAAACAAGAA-GCTTGGCAAAAGTTGTA--TCAATCC------CTGAGACTTTGGTGAAG--GA--AGTAACT---

GAAGATAT--GGTAGAAATAACAAGAGAA-CTGGA-ATTCAAA-GTGGAGCCTGCAGAT-GTGAC----TGA---------------ACTGA-TGCA---

-AT------CTC--A--TGA-------GA--AGA--TTTTAACAG-ATG------AGGAAT--TG-------------CT-TCTTATGGCAGAG--CAAA

GA-AA--C--CCA-TT----CC--TTGA-TATGG-A--GACT----GTTCCTCGTA-A-AGCTGTTATGCACATGGTTGAGAT-CAT---AACAA-AG--

GATTT--TGAG---TATTA-CATAAA---CT-TT-A---TTGATAAAG-------CAGCA-CAAGATTTGAGAGGATCA-AC-----TCGGATTT--CT-

--TTTA-A----AGATT-TATTTATTTATTTG---AAA--GG--CAGACTTACAG---AGAGGCAGAGACACACACACACACA-CAGAGAGGTCTTCC-A

T---CAGCTGG-TT-ACTTCCCCAG-ATG--GCTGCA-ATGGCCACAGCTGGGTTGAACCAAAG-CAGGAGCC-AGGAG-CT-TCTTCTAG--GTCTC-C

TGCATGG-----GTG-CAGG--GGT-CTAGGCCCC------TGGGCCATCTTGCACTGCTTTCCCAGGCCATAG--CAGAG-AGCTGGATCAGAAG---T

GG-AGCAGCTTCAGGGCTGGCAC-TGTGGCCTAA--CAG--GTAAAGC-AGCTG--CCTGTAGT-------GCCGGCATCCCAT-----ATGGGCACTGG

TTTGAG-TCCTGGCTATT---CCACTTCCTA--TCCAGCT-CT--CTGCTATGGCCT-GGGAAAGC--A-GTG--GAAGAAG--GCCCAAGCCCT-TGGG

CCC-CT-GC--ACCTGCATGGAAG-ACCTGGAAGAAGCTCC--TGGC---------TTC----GGATCGGTG-CAG------CTCTGGC------TGTTG

TGGCCA----A---------------------ATGGG----G--AGTGA---ACCA---TTG--GAT------GGAAGACC---TC------TC-----T

CTCTC-----------TGTGCCTCT----CCT------CT-CTCTA-T----GT-A------AC--TCTG--A-CTTTCAAATAAATAAA----------

--TAAATCTT--AA----AA----AAAG----AA----GAA--------G--------TG-GAGCA------GCTG-GGA-----CTCGA---ACTG---

-G--TGC---CCATA-----TGGGATGCTGG----CACTG----CAGGCAGTGGCTTTA------CC--CA---C--TATG---CCA---------CAG-

-------TGC-CAGTCCCT-TA-ACTTGAAT--TTTTAA-TGAAGTTCT-A---CTGGG----AGTAAAAT-GC----TAGT-----AAACAGC--ATTG

CA---CC----CT---ACA------GAGAA-A-TCTT--TT------ATGAAAGCAA-----GA----GT-CAG-ATGATGC---AGCAAATTT-CATT-

-GTTTATTT----TA-AGAA----GT---TGCC-AC-AG--CCACCC--T--AAAGTTCAG--CAA--CC-----A-CC------ACTCT-TATCAGGCA

G--CAGCC-A---TCAACACTGG--TGCAAAAGCCGCC-ACCAGCAA-------AA--C-AA------TTACAGTTCACT--GAA---GCTT--------

--------CCAGTAGTGGGTAGA-GTTTGTT-AG-A-AGTTAGGTATTTTTTAGTCAAGGTCTGCGTCCTCTT-TTATTAT-TA-T----TTTTTT-TTT

TTTTTGA-CA--GGCAGAGTGGA--CAGTGAGAG-AGAGACAGAGA--GAAAG----GTCTTCCTTTG----CC----G-TTGGT---TCACCCTCCA--

-----AT---G-GCCGCCGCG-GCCGGTGCGC-TG---CGG-----CT-AGCGCACCGCGCTGAT---CC--GA-----TGGC-AAGA-GCCAG---GTG

-CTTCTC-CTGG----TCT--CCCA----C-------G--------G----G-GTGCA---GGGCC---CA--A----GT-AGTTGG-GCCATC-CTC--

CA-CTG---CA----CTCCCT---------GGCC---ACA-----GCA-GA----G----AGCTGG--CC---TG-----------G-------------

--AAGAG--------GGGCAA-CC-GG----GACAGAATC---CGGCGC---CCCGACCG---GG-----------------ACT-A-GAA-C-------

--CCGG-TGTG-----CCGG--CG-----------CCCCAAGG-CG--GAGGATTAGCC------------TAG--TG-AG-----CTGC-G-G----C-

GCTG---GCCTG-TG--CCCTCTTTT-AG----ACCAGA---TGCTAT-TG-C-ATACTTGATAG----------ACTGTAG-TATAGTACAAAC--ACA

ACATATATGTACTGGGAA--ACC------AAGAA--A-CTC-----C---CTC-G-ATT----G---C--AA---TAG------TTGCTATATTGGGGTG

G----CCT--ACAACGAAACTTGCAG--TATTT-CTGA---GG---CA----------CACTTGCATGTATAT---TCACA-GTAG--TTGGTAATGATA

TA-AACCAC-TAT-----GG------TACTGAT--CTATGTATT-TGCTATAC-CTATACTT---TTATC--TTCGT---GTT-AG--CAT-----AC-G

CT----CC--TACTT--AGAAAAAAG--TCTACTG-TGAGACA---GTGTGCT----GT-G--CT---GC--TGG----CAGC------AGCCGCAT-A-

-CA--TCTC---AT--GTT----TA---ATGCAT-CTCCTGA--TTGCATCA---TTTTC----TCTTGTGC--TTGATTT-A--GTCTTT---T-GTT-

---GT--TTTATTCATCATG--GCC--CCTAAC-CA---CA-CAAA----------ATCCA-CT----------GCTGAT-A-----T---TTCC-----

AGTA--AGAGG---TCTG-GTC---GAGTGACTGG-------GAAATG----------AAATTGGAAGTG--AT-TAA----GGGCT---ATGAAG--GA

GG-A-AAATCAG------GCATGGCTGTTGCTCATCGGTCAGGCATGTCTCAGTCCAACA-----------CAGCTACAG---TGTTAGAA------G--

--G-AAAG----AATAAG----TGCTGGAAG----CT----GTCAAG--------GGATCAG----C-----CTCA--TG-GAAGGCAGTGAGACCAA--

--CAGGACTTTGACATCGTG--AAGTGGACAAAGG-TC------GAGTGACTAGACTGGAAGAC--AATACAGCAGCGG--A-T-CC--CTTTCAGC--A

CCAGGATGATCCTGGCC--AAAG-TAAAA--TGT--TTGC---CAG-C-------AA-TAT--TG-----CA---AGGAAA-----GGCTA----G---A

CTTGA-C---CTTG----ATGTTGAATGTACCACC---AGCTTTG--GGCATCT-GAAGT--GAT-TCAGGAATCATT-TATT--CA-TCA-TGTAA-T-

-GTG-----AAAATCTTCAAGTGCTAAG-GTGACA-GCAA---CA-GAAGAAT-GTTTGG-AAAC-GACAT--AACCTG-AATGT--GGA-GG-AA-GAT

T--ACTGACCAGA---GTAAATCA----------------GGTAAGAGCAATC--TGGCA----GAACATC-------AGGC--TACCTCGTCTAGGTTA

GG-T-GTGCA--GTACGC--------TG---T------ACTGTCTAG-GCT---T-GTGAAATACGCTCTGATAT-TCACACCATGATG---AAATC-CC

CAAACACCACATTTCTCAAAATG--AGCTGCCTTATTAAAG-TAATGCT-T---GA-T-----TGCATTCTAACCTCATTCTACCTGTATTTCTCTATGA

GTGGAAGACTAGC---AGAG--TTTCCCATGTTCTATCCATCCATCCAGAGGATTGCTCCTCACTGCTGTCATTCAAGG---TCCCCAAGTTGATCT--A

AAACTGTGGT--TCACTCTCAGCT-GTGTCCACACACTGAGCTGAACCATCTGTGACCCAAGCTTAATCTTTCTCTTGCTCTATC--------------G

GCTCCC------CTATGGTGGTGGGAAATGAAGGAGTGGCACGGCAGTGTGGCTGGTGTGGAAGTC--AGCCCCCTGCTCTTCTGGCT-GCTGT---AT-

---TCTG---CT--TGTGCCCAGTCCCAGTGGGCTGCTTCG-TCTACAGTGCGTTGCTGTGGAGCCCATCTGGAACTGTGATTCAATGGGTTTGATGGAA

CTCGGTTCATGATGGGCA----CT-------TCTGGCTACAAAGG--A----A---CCCA-----ATGTCTTCT-TCTGTGCAAGAGTCCAACAACCCAC

TGGAAA-TTGTCCCTCAA----T-CA-GTTCTTCTCTTCATGTGGC-TGGCTTTGTGCCTGAACCCCAGGTTCTTCCTCAGAACCAGAGACTCCACAGAG

CACAG--GATACTTTTCGCCCA-CTGCGGTTAGCACTAATGTAGTAAAGTCCACCTGGTCTGATGGCccaagctgctggatcactcacatctcag-CTGG

ACCCATTGCAGAGACCCTTCCTGCTCAGGGTCC-AGTCACAATGGACAGCCCTTTATGGATGTGGGTCAGAGTAacctccccaagtattgatgagctgcc

tgccaagtgctggggctcctgtttaccacaggaggtgtgaacagcagtgatcagtctcttatttcagaaggaatgcctaatcctatgggatgttctggac

tggtccttcagaattttccaaaattaaaattaggtcactaatccttcacatgccgctatcaaccaagattggtgagtgtgg-------------------

------------------------------------CCTTGAGGGGGACAGCTGTCTATAGCCAAGGGCAATTCACatggagcccacagtgaagacctgt

tggccaccaatgctgccaaagatgggaaagtgactgccttcctctgggttca-TAGGAAGGGCTCTAGGCAGAGCAACACAACAttcattacaagacgca

gcacctggtcctgaggcacagtcgatgagttccggctttgtgcTGTAGCTATTTTATAACATCACCTTTGGAAACTCATGTTTcctccttggactTTAGT

TTACCCAACCATAAAGCAGATTCTTGAACTAGATAgacagcttctaatcgactgc--ATTACAATCACCAGGGACGTGTTGAAGAGCACAGATTCTAagt

gatcttcccccacAGATTCTAGTTCAATGAATTGGAGTGGGAGTGGGGCTAaaGGAACTTATTTTACCTCCTGGACTTGCTCCAGCTCCGGgtagcttcc

caaactgttcaatctcactggtagaatgtagataaagctgctTGCCACCAGAGGATTATGTGAAATAATAGATTACTC-CAGTGTGAATAAAGGCGTGTG

AGTGT-TA---GGCCTGGTTAGAGGGCACAACCACGGGCTTCCCCATGTGAGGGAGGCA-TTGAG--CTCTAAGTCGCAATTCTTTTCTCTTTGTAATGA

GCACTATAAAAGCTGTGCACACCTCAGAGATTGCTCTAGAT-GATTAAATTAGATCACACACATGAACTCCATAGTCCCTGGcatggagaaca-------

-CAGGCAAC-ATCATCAGTTACTAGGCATCTGCCAGCTCAAGGCTAG-TGCAATGCCGGCCTCTCTAGGAAAATGA-CTTGGTAGAATGTTCCTTCCAAG

GAGCCCCAAACAGCTGTAGGAAAGatcaggggaaaatcctggttggttctcttgt--AGAAAGAAC---TGAGAGCCTGGCATGAGAAAGCCCACAACAG

TAGAAGGCTCATGGAACAGCTCAGAGGATCTGAGCATTGATTCAAATGGACCT-CTAA------TAC----CCTGCATGG-TTT--GGCTTGTGCTCCTG

-----GGGGCTAGGTTTTCTCTCTGAGCAAAGACAGGGTGGAATACAAGGAGTTCTCAAGTCCTATTCA-TTCTTTCTCTGACTATGGGGTCTCAGGGGA

AATCTGAGAcaGAACCACAGAACAGAGCTGGG--TCCA-GATTCCAGGTTTACCCCCACCTGATCATTTTGTGTCCCTGGGAGCCAAGTAGGAGACCAAG

AGTTCCAATCCCACAGAGGGTACCCATTTCCAGCCCCAATCAGagttattttcaattc-CAGGTTTCTCATTCTCCAGCAACAGATGATATGAATTGAAt

cCACGGGAGGTGGTTGCTGTAGGAAAATACCAAGAGAGAGAAACACAGAAAAAAAAAAAAAAAAAAAAGCCAGAGAGCCACTCCCTCTGCACAGAAGGGA

G-TGAGCTGGGCAGAGCGGCCTCCAAATCCAGATGGTCTGAAGCATTGGCCGAAGATG----------------GCAGGTAGCACCAAGCTGAAGTCCCA

GGGAGG-AATTAGCCAA-TGAGAATAACAGATCGGCATCCTGAGGTC-CAGGAACACTTGCCCAGTTATGGCACTGCTGTCGCTGGGAGACTGCTGTccc

tatactatgtc--CCACATTCGTGTGGAGCCCAGAGCACTCCCAACATCTGAcCCTTGCCAAGAGACCTGGGACCTCTTCCCTCTAACTGGCAtgcctca

ccatcactgttatatgacgcaaggcgtttctt-----------------ATAACACATCTCAATTTATAGGCATGAGTCATTATTTGAGAGGAATTGTGA

GAATTTCATTGTCGTGT-CTTTTAGTCCTTTCCCATTGTCCCCTGGCCATG--TCTTCTAAAGTTCTGTGAAA-TAATGACTACTTCAG-TTACTGAAAG

CGTACAC--TGTGACTGTCACTGATCAAAGCACTTCTCATGTG-CAATCCATTTAGCCCTTACCGCAGTGTTACCCTGCTCTTTCCAAAGGTG-AGACC-

AGCTGCAGCTTCAGGTAGTCTTAAACCTCTTCCATACCTGGTATTTGACAAGCACAGGGCATCTGTCAAGTAGACACAAGTGGGCATTT-CCCTGCTCCC

ATGACAAGGACA-CTGTAAGACTTTACCTTGAAGAAATTCAGTTGAAGGTCATA-GCCCCTGATCTA---TTGATCCAACATCCAAAATAGGCATGC-AG

CTCCCTTTGGGAAGGGCCcctgtgcctcttttcttttcttttttttttttttttacaggcagagtggacagtgagagagagagacagagggaaaggtctt

ccttttgccgttggttcaccctccaatggccgccgcggccggcgcgctgcggccggcgcaccgcgctgatccgatggcaggagccaggagccaggtgctt

ttcctggtctcccatggggtgcagggcctaagcacctgggccgtcctccactgcactccctggccacagcagagagctggcctggaagaggggcaaccag

gacagaatccggcgccccgaccgggactagaacccggtgtgccagcgccgccaggcggaggattagcctagtgagccgcggcgccggcCCTGTGCCTATT

TTCATGCCCACGTGTCATTCACAGGATTtaggtgatgactgttggtccctggggagttTGAGTAAAACCCCATCCGTTGATTAGAAAAGAAAgtgcaagc

ttgactgGGCAGATTTACCATCAAACAAGAGAAATGCACAAttggagtggcagaagcactgtggtctagcataaaatctgagcaatttaggaagtcaaat

aatttctccat------------------------------ATTTGGAATAGTGGAGAATAAAAgacaagcacttgaggagctggtggtgagaaacagca

ggagcctagtat-------------------------AGGGACAACTTGACAATTGTCTCTGAAATGCCTCCCAGCTggtgtccaaggcaatgtttttct

ccagaactatacactaaagaatttcccaCCTGCACCAAGTGTCCACTAGAGGAAGccaatcttcaaaaagaagaagccaggtctgggttaggttcacacc

TTGTCCATTACTCTTGCATCAGACACCTTGTCCTGATgtaagtgttgtggggagggatttttactgggtcagtgtgaaatgcactgtgttttccctgga-

--------------------GCCACCAGCTCCTGTGTTGCATGGACCACTCCACTTGCCttgggatctattgat-ATTCCCCTGTAATTTCACTCAAGGA

AATACTATGACAGCTccagtgtaaata-AAAACCACAGCTGTCCAAGATCTCCATGTGtcagttttacatcttctgatgctaagatccaagagtgttaac

tgtgggagttggagtccctagcttgctaaatgctggtttctcttctgtactctggcttgcttgcttgcctgctgtaacgccatgataagcctcaccctgg

accaggaaggaggcaggccgggtccttgagaacaaagaaagtaagctcccctggcatttatgaccttctgccccccaccagctcccctggcatttatggc

ccaatgcttccctccagttcctccctgtgccatctccttggtgtggccttcccgccacatagcgtatatagtctttacgtttaaatgaaccagtgaacat

gttaggtcccaataccttgccatatagagaataagaaggtaaactacgcaggagatgacctgcactcttgtaagtgtgaggctataaaaacctctcttat

gcaaacgagagggccttttgcctgttcatggctaaaagactgtgtgtgtgtggggggtgggtggttcccagccggctggtaacaaataaacctctgcttt

tgcatccttaactgtgccttgtgcattgattgggaaattcggacacaacatTAACTGTAGGGTTTCTGGAATCCATCACTGAGCCAAgttctgatttaac

accatcccacactgcaaacgaatgagcaaggtttagacaggcagagacttttcaacccc-------------------CTGGTTGGAATCCCAGCCATGG

TTTAAtccatgatggagatgctgcccgatggcagggatgggagaaattctggatctgcagtttcctttctacagaaggcatcttctcc------------

--------------------------AAGTGGAGACTATAAGATGATGCAACTGAAAGTATATTCcgttcttagactgagcatacctactctgtccctgc

cactctgtccc---------------ATGGATGGAAAATGTGCCCAGTCCCAGCCCACGGGTTCCCctttttgcttcctggaaacttcttcagggtacac

tgtctactatttcaaatcttattt-----TCAACATTCCTTGACCATGACCCTGCCAAGGAATTCCCTttgatctctccgatttctactcccctgtacaa

tgataaatcattactctac-AAAAATCTGGTAGAGCAGCCCCAGGAGCAGGGAGGCCa----aatcc-agaaacctccagctcat--cctccaatgtgaa

gatctc---------tctgtgcctactgaacc-cc-tg----ccc-----tctg-taaagcaatgttaa-cc------------------------CCTC

CAGAGCAATCAGATCAATAATGGTCCTCTGAATG--------------------------------------------------aaatgcaga-atgtt-

---gggt--ctggcgttg-tg---g-tgcaa-tggg-tt--aggct------gtca--ct-tgcaac----cccagcaccccatatctg---aat--gcc

-a----att-ca-ag-tcc-aggct-----g----c--tctgctt-----------c---tgatccagc-t--t---tc-tgc---taatgcacc----t

ggga-----aggcag-tggat-gtttg--ccca----tttg--------tt--------tgg----gccc-----ctataa----c-----------cca

--tgtgg-ga-g---acctg-----------------ga---tg------gag--tt-cctg-gc-------tt----ctg------gctgtg-----gc

c--t-------agcct---------agtg----ct--------ggc---t---gtcatgg---cc---atct-----tg-----ggaat-gaa-------

-c-------taggg-----agtggaat-a-----tt--c--tctcc----ct-----ct-c--------tctc-tct-c-tcttt-ct--c--t---c--

-t----ctctctctcttcat---tt-caaat-agataaa----t----------aaataaatatttgtt-g--atga-tag-gaaat-g--t-------a

tt-tc-g-gg-agggac---ttga-----agg--g-tcacac-----cctgtggtctga--agt--ccagg-cca------ccag--atctctgggctct

-ttct--tagac-accagctttgaaaacag-gtctactccag--attatt-gtatg----attct----gggat--t-tcca-ag--agtgcattt----

--tgtttataa-aa--ttaggt--agctac-----attttattt--tggggca------aca---tgaa---g------------------CTGGAAATG

-TGAAGCTAGTAGAGTG--G--GAAGCTGGGGGCACAGTTTTAGGAA-TGGGGATTATGAGCCCAATGAGTGGCCTTTTTACTTTGTTGGTCTCCTCACA

GGTACAAGCCAGACaaggctgctgctccgtgtgcctgatctcagcttcctgggacctgagggcttcagtcattatc----------CAGCATCGCCCCAC

AGCCATGCCTTTCCTCTCCCGCCCACtgtgtcctctc-----------------TCCCACACAGTCAATTACAGCAGCCTCTGAAATGTTTTCCtggagt

ccttcactgggtctttcccaattcatcctccacccttgagaaaaactactgtgtatacattgagagctctgtggaagagcccacgccaacagactgagtg

gc--------CAGTGGAGATGAAGCCTAAGCAGGACGTGTCTTAAAGGAgagagtgaagccggcgccgtggctcaataggctaatcctccaccttgcggc

gccggcacaccgggttctagtcccggttggggcgccggattctgtcccggttgcccctcttccaggccagctctctgctatggccagggagtgcagtgga

ggatggcccaggtgcttgggccctgcaccccatgggagaccaggaaaagcacctggctcctggctcctgccaggatcagcgcggtgcgccggctgcagcg

gcggccattggagggtgaaccagcggcaaaggaagacctttctctctctgtctctctctctcactgtccactctgcctgtcaaaaaaaaaaaaaaaaaaa

aataaaggagagaGTGAGATCAAAGGAGAcagttctgtggagccagcttaggatggagaggagaacAGTAGGCAAGAAGCAGAAATCTCGTCTGGATGGA

ATTTGGtacaggcttaagagaaaagtttagaaatttcc-----------TGGAGTCACCTTTTGCAGGATCTTGAATGTGAAGATGGACttc--------

-TTCTGAAGGGCACAGAGAGATAGTGAAAGTGTTTGAAccagcagggaagggaaaggaagtggcctgatcagaggtcaggaagaggaagctggtggctgg

ggcaggacagagcag-GGAAGGGAGGACCCATGAGCAGATCTCTGAAATGGTCCAGgcaggaagtggcagggatGATGGAGAGGAAGTGGGAGCTTGTAG

GAAGAATGGGacaaagtgagacttcattaagatacttagcatcacaggtggtgcttcaggacagacccagccca--------------------------

-------------------------------AGCCCTGCGTTGACTCCCCTCTTTCACTATCCCTTTA-GCCCCAACTACAGAGGATTGGGAACATGGAT

TTTAAGAGAtagggacccctctcttttgtgacctcctgagattccactgcagctcctccttccaagctggaacctactggcagggaaaccagctgtatac

caagtgtaagaccaaccaggagggctcctgcctgtgggtcttggggaaagacagggggctttggatagagcagtacactcagacacatgggaagcagcag

acagcgctgattcca---------------------------------------------------------GATTGGCTCTGACTCTTGGTTCATTGGG

GCATATCAAGTT---CTTTTGGGTGTCAGTCTCCCTTCCCATTAACCAGGGATAAgcCTCTATTACAGACAGAAATTGAGGGGATCCGGAAGATGActtt

acccacaagctcctccagctctaggagccgtgtctcttggcaagcattattatgtgccctccctgggagagagtaggagggaagggatttcccacatgta

ggagtgcatggaaactggaatgaaattgactaagatgccaccatacatactgatagg-------------------------------------------

----------------------------------------------------------------------------------------------------

----------------------------------------------------------------------------------------------------

----------CAGAAGACCCTCTGGCACTAGGCAAATCTTGAGCAGAAGacagggaatggtaatagtcagggagtggtgaggtgggtcctagtgccagac

ctcaaacaaaacttgcttgccctccctttgcTCTTTATTTGGAAGAAGTCCTCAGTCCATTTGTCTTCCAccTCCTACAGTATGAACCTGGAAACTCAAA

GCAATGGAGAcattggaacctctgatccagttcccagcagggaccagggacaaacatgggtgtgctagtcatcactgagcaccacgacctcagctttctc

ctttggccatgcctcttcactaaatcagtggagcacgtgcaaaccattttccttccaagggactcagccatatatgaatatacttggcacgagatcaatg

gtctccaaattggctgcaaactcatattgccaggattatttctgaaaaatgtttatttccgtGCCTTATGCCCAGATTCCTAGTCAGAAAGTTGAGTTGT

GGgaaagcgtcccatctgggttttcaacgttttccctggggacccccgggttctagcttgagaccagaatgtgggatatga----TCTGAGCTTCCTGCG

TGCTTTCCTATTTGGATTCGGAttccactgtcggaAACTTTCATTGTGGTCCTTGTCATGACCTTCCCAACTCCTccgttggccgctgattgttc-CCAG

CCCTGGGTCACCATGGACCAGCTGTGtataaagcacttcctctctttcttctcacgtctctttcagttctacctaggattttgtagc-----CTGTGAGT

AAACATAGGACAAAGCTGAAGCATGTAGCCAgaatctaatttcctgctat--------ATTCTTCTACCTTGACCTCCTACACGCCTTGGGAGTTACTtg

aaAAGACAGAGCACAGCTTCCcgcactagagggctcatccCTTTCCCAATGCTAATCTACCCCTGTCcaccaggaacccATGGTCTAGACTACACTCCCA

CCATTCACCAATTCCTAAActgcagcCACTTTTTCTCATCAGCTTTAGATAACTGCTAATCAGGAAgccgctgagagggaaagagaaggcagaagagcca

attctctgtagactgcagccgggcagctgcagggaactgcagaacc-------------TGGGCACCCTGAAGTTGCATTTTAGCTTTTCACACTGAGat

ttctcaatccagactaaaactccagaccagacgaagggaagttgtaaaatcaaagggacctgtgggcacaatctctgaactccctgcagaacttgggcaa

tgatgtctgtctggcctggggtcgttcccagtccc----------TTCTGGGACCCAGAGCACTCAGCACCGTTGTGTCATGTGAgggTCCTGGGGCCTC

ATATGCCTCTGTTTCCAGGAGACTCTatttcctcctctacagcctgacattcctgaaggg------CTTCTCTACTACATGTCTTCATAGGGAAGCACGA

GGgtagctgagggatccttctgtccaatggtccaggcCCTGGGTCTCTTCCTAAGGGCCTCTGATCACCACATTCCCctccttgtcaatactcgtactac

ctgGTGTAGTGAATACTTAGCATATGCTAACA----ACT---CAATGTTTTTCACCTGTGTCAGCTTGTCCAGTCCGCACCCATGTCACCAGTAGGGAAA

CGGAAATGCAGAGAACTGAAATGGG---------------AGC--CCGAATCCTGGCA-TTTGGCTTCAGAGTC--TCATCCTCACCATCATCCT-CCCG

TCCTAGAGCTAAGCAGCAAAGAGCTCTTCTCTGCCGAGTCCCGAGGCCCTAAACACCCAGGCCTCAGACCAGGCTCATTTCCAGCAGGTGATCG-TGGTG

GGAGAACGAAGC--AGAGGGA-GCAG-CAG-CTCCTGGCCTAGCGA-CAGCCGACCTGTCACAGAGCTCGGGCTCACCCC-GGCCCTGCAGCCAGGGAGG

CGATCAGTCGAGAATCACATTGCTATTctgtttttaaaaaattatttattcatttatttgatgtatttgaaagacagtgagagagagagagagagatctc

atcgtcatgattttttt-ttaaaaagatttattt-atttacttgaaagtcagagttacagagagagagagatccccttcactgattaacttcccaaatgc

ccatcaaagccagggctgggccaggctgaagccaggagcccagaactcaattcaggcctcccccatgggtatcagggacacaactacttgcaccactgtc

agttgcctcccagggtgcacactggcatgaggctggaatggagagcaggaccaggactggagcctaggcaccctgatacgaagtgcaggcgtctcaagca

gtgtcctaatcacagagtcaaacaccagctccacccctacttctcacagacagcataccttc-tcactgtcttttccacaaacacctgggctttgcactt

gcaacctgtttgtcctcctcgatcttgttcatccccctcaagtcatatgtgaaaccgtggacctaacagaatctacttaggccttctggctacttagcag

gcgagccaggatttcccctgcagtgctgcgaacccgctgtgcttcctcaccatgaacgcagggctgctatttctcttttcctgatgcagctctgtgtcag

gccacgtgcccgcagaaggaaaatgctctgggtgatccccacgggggcctgcacatggactgtgggtttgtatgaacttaggtcctgacaaaactcaacc

aggtgctgccgctgagtcacgcagtaggcgggggacagtcaagtgctttatgaatgattTAAGTTCCCACTCACGAGAGGAAATCCTGAGAGCCAGGG-T

-TGTTTGCCTTTCTGTCACTTCTGTGTTGCTGAAAG--GAACAACAGTGGTTTTTTTTTTTTTCTTAAATGGAATAAACAAGAAGT------TCTGAAGT

CTCATATATACAGA---AGAAAAATCATAAAGATGATCTAAGCTAAGGAAATGATCCAATAGCTCACAAGGTAAAA-CA--AACTAGTGAGCATGCACAG

AAAAATACCAACCAATAAACATTTGAACTGATCTTAGAATAATTTTTGTTACTTGATTTATTCCCTT--ACTTATTTGAGAGAGAGAAATAGAGGCAGAG

ATGGAGACAGACAGACAGACACAGCTCTTCTCCACTGGTTCACTGCCCAAATGTCCACGACAGCCAGGACTGAGCAGGGGCCAGAGCCAGGAGCTGGGAA

CGCAATGCCCACATGGGAACTGTCTCTCATGAAGGTGGCAGGAACTCAATTACTTG-AGA-TGTCTCCAATCACTGCTGCCCCCCCAGAACCTCTACCTT

AGTGTGAAGTTGGAGTCAGAACCCAGAGCCAGGAATCAAACCCAGGCTCCATGTAGAATGTAGACCAAATGCCCAACCCCTTACTCACTTCTTCCAATGC

AGTATTTTCCTACAGTAAAGTTCAATGAAGTTAAGTACTATATATATATATATATATATTTCTATGCCAAGAACCACCGCCTGAGACAA-AATTTGTGGA

ACACTTCTGGCCCCCATGCCTCCTGCCAGCTAG-AAGCTCCCAAAGGGGCCGACGCTGTGGCACAGTGGGTTAAGCCCCCTCCTGCAAGGCCCACGTGCC

CTGTGAGTGTCAGCTTGAGTCTCAGACACTGCATTTCCAATCCAGTTCCCTGCTAGTGTGCACAAGAAACAGTGGAGGATATC-CCAAGTACTTGGACCC

CTGCTACCCACATGGGAGACCTGGCTCTGACCTGGCCCAACCCCAGCCGCGGTGGGCATTTGGGAAGTGAATCAGCATATGGGAGCTCCTGTTCTCTTTC

TCTTTCTGTATGTGTGTGTGTGTGTCTGTCTGTCTGTCTGTCTCTGCCCTTCAAGTAAAAACTTTTTCAAATATAGAAGAAAAAATTTatcttgacataa

agataataaaatggtttcattcaaagtagactattgtatttgatcttaaatttttaaaaatgctgctgtaagcaacagtgactattttctgtgtgacaca

gaaatttcagagcataatggctcatttacggagctccacagatgctaaaccacaggttgtaatcttcaga------------------------------

----------------------------------------------------------------------------------------------------

----------------------------------------------------------------------------------------------------

----------------------------------------------------------------------------------------------------

----------------------------------------------------------------------------------------------------

----------------------------------------------------------------------------------------------------

----------------------------------------------------------------------------------------------------

----------------------------------------------------------------------------------------------------

----------------------------------------------------------------------------------------------------

----------------------------------------------------------------------------------------------------

----------------------------------------------------------------------------------------------------

----------------------------------------------------------------------------------------------------

----------------------------------------------------------------------------------------------------

----------------------------------------------------------------------------------------------------

----------------------------------------------------------------------------------------------------

----------------------------------------------------------------------------------------------------

----------------------------------------------------------------------------------------------------

----------------------------------------------------------------------------------------------------

----------------------------------------------------------------------------------------------------

----------------------------------------------------------------------------------------------------

----------------------------------------------------------------------------------------------------

----------------------------------------------------------------------------------------------------

----------------------------------------------------------------------------------------------------

----------------------------------------------------------------------------------------------------

-------------AGCTTAATTGTGACCTCAAAAATGATGGAATAAAAAtatcacccaagtacaaatgaacacctctgtctattgaggacttc--AATGA

TTTCCCACTTTCCTGAGCTACTGTGGATGACccgatgagaccatcactagtaccatctgCTGACATTTAGATAAAAACCAGTTGTGGGTTTataggggtt

gttttaaccaaaacaataaaatacacTCTACTTAGACTTCTGGGGAGTACAGTGAATGTTACAAAcccacgTACACCCACCACTCTGCCTCAGATGAAAA

TAAAcacattttttcaaaatgagtgcactgccttcctgtgaagtgcttgagtgtttagggaaggaacacccccgcgggcagtcactacctcacAAGGCTT

CACGGTTCCCAAGCTGGCGTGGATAGCACGGAAc-TC--GCAGC----AG-------TGA-----CCAGTGCTTGACTTTCTGTGCA-GGGGT-GGG---

-AC---CA---CA-C----AAGCC-A-------TGTGGCCCAGCTGGGACAATAGCAGG-AAGCAGAAGTGACATTTGTCATTCCAGGCAGAggccttca

gatgaggacaggagtctcctgtggcttgcacttgccacagtgaccagggacgttctcagggacagagactccaacagccgagcaagcaaagggaagagca

gaccccctgctcttcactagtggggagctctggcatctggaccagaggcaagcgtgctaggctgccccaggctGTCCCACACCGTTCTCTGCATTGCACT

GGAGACGAGGAGCcagggc-AGAGCTTGGCCCTAACATCCAGAGACTTGGGTGCTCTGTTtaatgacacctaggcacactaattctctgtcctgttcctt

gcctaccgcaccaataacacagagatagg-----ATTTAGGAAAGGACAGCAGTCAGTGGGCAAACTCCATTTcatctttaactccccgattcatgtctc

tcaGACCACACCACCTGGGCCACCAGTGGGGACAGAGTGGcatgcagcagaatgcacacagccaagccaacgaggaacccagggtcatggcagatatccc

cacaaacaccagcagaacaggcaggagacttccacacc-------CATCCCAAGAGCGCTCTCCCAGCCCCAGGAGCAACTGGACcactcaggaccacta

gctgtctaagagctcctaacagaccagtcctgcctcccctccctctcacacctggcccttatccccagtacagcactccggagtttatcctac---AATT

CCAGTTTATTCCATGGAGTTACAAAGGACAAGAATAaacgccagctcatggtttactgagtctcacagtgttCCCTGTACCCAGCAAATAGGAGACAGTG

CGTGGGGATTGctgatgcaagggtttgatggggtcagctccatggcccacaagcacacagaatctggccacactccttttacccagcaatcctctcgaat

gttttactttccatgtgggtcagt-----------------ATTGCCTGTCATTGCAACGTGAACTTGCAGACACAAAAccctggAGATTTCGGAGCCTC

CCTACGAACAAAGACCCTAGCAGTTtccaaggaccagccagggaggccccatgcatccccagctccgaattctggtttctgc---------------TCT

TTATTTAATTGTCAAAGTGGCTTTGAGAAAATCATTTccccaccttatatCTCAGTTTATGCATTTGCAAATGAGGCATTTGAGCTAGtcaaggctacca

agttcatg--TTGGTTAAAGATAAGCTGGTCACCTGGAGCACTTCctaagcagagaatttgagacatcatcttctaggattctgtaggtttgggcaaaga

caccctcccca----------CATTCTTAATAATTGCTCGGGACACCTTGGTACTACAGGcaagcctggggactggtgtattggatagtcgctgagattc

ctgggcggttggtcactatggttttccgcctggtcctt----CCCTCTAATTGGAATTACAATGGGACTCTTGTTCTGAttgtccactagctggcctggc

ttattgtggagtaactgtgcaacctgtgacaaatctgtacacctctgaattcctctctcatttataaacggacaagttaaatccaaatcc---------C

CTCTTTCGATTCATTCTGTCACACTGTGAcactggga-----------CACAGCTGAACAATGCGGGCAGAAATCCAGATTCCCAGaactCTGTCCCATC

TAATCATGTTCCTTGAGAAGTAGGCCAGAggccaggagctggattttcccagcaagataccactttcctgccctCTACTCCTTGCGAAC---TGCTTCT-

-TCCC--------CTCCATTAGCCGTTGAGCCGTGGGCA-A---G----A-A---------------AGC----AAATCT---TTGGGGA-TT-------

-------CATCAAGGAGAAAACAAAGAGAAAAGGCAGAAAGACTACTctccaaacaacacagctggGGCCCCTGCCCAGAACAGCTGCTGCATTTGCATA

GCCTAtagtttccTTTTAACTTTCTACACTGAGATTTCTCTAACTCAGTTTAAaaacaaAAGGGAGGGAAATTGTGTCATCAAGA-GGCTATCTGACTGA

ACTCTCCAAACTTCCTGTGCAATTAGA-TCCTG-TGTGCTTGGGCACCTGTCAATGCTGGCTGGCATCGAGGTCCTGTTCCCTCTGATGCTCACA-AGCC

--CCACTCTTGAGATCCGTGGCAGGATCCCAGCAGCTC-CTtgtctgctgtttctgggaaatagctttatttcttggtctcccttatatgcattcccaga

ggc-CCATCCCTGCAACGTGTCTGTGATCATTTATCCTCTGGACtccatgcccgcctgcctggatgcttgattAAAGC--G------GATTACTCACCAC

ATCCT----ACTCCTCAAAGCAAGTTTTTGGTGTTGGATGAT-TCGATA---TTCACAA---CAAAC-CCTATTTTCACATCAGAAAATGAAAAGATGGA

AAAGGCATTTGCATATAGAGAAGCTAAATA-TCT----------TGACAGAGATATTAACTAGTGTTTCCTGGAGCCAAAT-----CTCTCATTCCAGCC

T-CTAGGCTTCTCGGCTTCTCCTGG--T-ATAGCTCCTAACA-TCCTC--AC--CTCC-AGCA---GGGTC-CTGTGC-CAACAATTGTAGACAAAT--C

AACTTGTTACAGA-GACCTGGGGTGG-GAAAA-CTCCC-T-CCATTCAAA-CAGGCACCAGAGACAGTGGAGGAT-CCAAGCAGTTTATTAC-GC-CTGC

CAGTGGGC-TCAGCTGGAATCATTTCCCCAGAACTG-AGCCACAATCCCAAGTGTCTTACAATATTAATAGGTTCATCAGCATCATACCTT--AGCCGTT

ACAGCATTGGTGGGTT-TGAA-TTGCAGCCTATGTGACTTAGGACCAC--A-C-TTCCAATGCTTGGTGGGTCCAGTATATTT-GTAAAAGAG-ATACCA

GGGGAAGATTTATTAGGACACATTTATGGCTAGAGTTGACAGAAGCAGAACTTAGGACATCTTCCCTCCCTAGAA-CAAGG--GGGC---AGCtgcttcA

CTAGGTAATTGCCAGCAGCCACTTTGCAAGGTTTGTGTTGGGAGG-AGGGGTC-CGCTTTTC-T--CT-CTT-TGTC-TCTGACTGCAGCCGTATTTCCT

CTACAATTGGGCGCCAGTTT--gagtcttggctgctccacttcccatccagctctctgctatggcccaggaaagcagtagaagatggcccaagtgcttgg

gcccctgcactcttgtgggAGACCTAGAAGAAGCTCCTG-ACTCCTGGCTCTGGATAGGCTCAGCTCCAGCTATTGTGGCCATTTGG-AGAGTGAAGATC

TCTCTCTTTCTCTTTCTGCTTCTGCCTCTCTG---TAACTC---TGCCTTTCAAATAAATAAATAAATCTTTAAAAAAAAAGAATAGATACCACACA---

-AAATATGTT-CT--TAGA---TCACAATGG-AATT-----AAACTAGAAAT-CAGTGAAAGATAAATGGCTGGAAAATCCCAAAATAGTTGAAA-ATTA

AAAAC--A--TAGTTCAAATAACA----CATGATT---CA-GAGAAGTCTCAAGAGAAATT--CTAA-A-AGATTTTCA---GCTAGACAAGAATAAAA-

ATT--CCACTTACTAA--AATTTTTTG----GATAAAGCATAACATTGAAAGAAAGTATTAG-ATAATA---AGAAAGGTACAAA-ATT--AATCATCTA

-AGTCCC-TG--TCCTATAAAACTAGGAAAAGAAAGCAATATAA--ACCTAGTACAGGCAGAATAAAAGAAGTAAAAAATTAGAATGGAAATTGATGACA

GAATAGGAA-AGCAGTAGAGAAAATAAATG----AA-----ATTAAAGT--GATTGTTTAAAAAG--AACAAGAAATGAACAACTGCTAGAAAGAAAAAA

CAAGGGGAAAAAAGAGAAGATGCAAAT-TACTAGCATCAGAAATGAGAAATACATGAATATGCCTGTA-TCTATTAACTAAATTGGATAAATCATTAATA

ATCTTTTTTTTTTTT--TTTGACAGGCAGAGTGGACAGTGAGAGAGAGAGACAGAGAGAAAGGTCTTCCTTTGCAGTTGGTT-CACCGCCCCCCCCCCAT

GGCCTCTGCAGCCGGCGCACCGCACTGGTCCGATGGCAGGAGCCAGGTGCTTCTCCTGGTCTCCCATGGGGT-GCAGGGCCC-AAGCACTTGGGCCATCC

TCCACTG-CACTCCCTGGCCACAGCAGAGAGCTGGCCTAGAAGA--GGGGCAACTGGGACAGAATCCGGCGCCCCAACTGGGA--CTTGAACTCGGTGTG

CCGGCGCCGCTAGGCAGAGGATTAGCTTATTGAGCCGCGGCGCCGGC-CATTA-ATA-ATCTTCTAAAA---GAGAAAGCATTAGCCCAC--CCAAATGG

T-TTATCT-TGTGAATTCTACTGAATATT--TGAGCAATAAAT---G-A--TACCAATTCTC-TA------TAATTTGTT-T-----CAGGA-GA---TA

GAATAGAAATCAATTACTAATTCATT-CTATAAG-GATAATTATTATCCTTACATCAAAACCAAATAAGTCGGGCCAGTGCCGCGGCTCACTAGGCTAAT

CCTCCGCCTTGTGGCGCCGGCACGCCGGGTTCTAGTCCCAGTCAGGGCTCCGGAAGAAGCTCCTGGCTCCTGGCTT--TGGATTGATGCAGCTCCGGCCA

TTGC--GGCC-ATCTGGGG---AGTAAACCAGCGGATGGAAGACCTTTCTCTCTGTCTCTCCCTCTCACTATATATAACTCTACCTCTCAAATAAATAAA

AATTTAAAAAAA--AATTTAA-A-AATTCAACAGCCATTCATGAC--AAACAA-------CTGAGTAAACTAGGAACAGAGGGACACTTGGTCAACTTAA

CCAACAGCATTTATGTAAGATCT---G-AAA-GT------TAATGGTGAAAAATGAGGTATTTT--------GATC-----CAAGGCACGAATGTTCCCT

CTCTTCACTTCCAGTCAACATGACACTGGAAATCCTTGTTAAC-TAA-AG-ACAAGATAAGAAAATAAAAAGATATGTAGATGGGAAGGGAGAAA-GAAA

C-AAA---G---GAAGAAGG---GA--G---G-G-AGGGAG----GGAGGG--AG----GAAGGAAGG--ATG-----GA-AGG-AA----GG--A-AGG

A----AGG--AA-AGA-AGGG---AG---------G-------G----GAG-AA--AGA----GAGA--GAGA-A-AGT-CT--TTGCAG-AAGAC--AT

GA--T----TGTCT-----TT-GTGGATAAT-CACAA-ATAAAT------AACCACAAAAACCCT--TGTAA--CTAAATAGCAAT-TG--TAA-TGAAG

---T--------TGTA-GGATA--TAAAGTTAAT------A-CATAA-AGTT--TCATAATTTTAT--GT-----CAGCA-A--C--------AATC--A

G-A----CTT---GAA----AT----TAAT---AAC----ATAT--A----CCA----TGTA---T-GTTAGCAC----C--AAAAAG--T-----GAAG

-C--AC-TTAC--GGGTT-AA--TTTA---TTA--AAACA-------AG----TGTAATAT--ATGA--G----GA-AAAT----CA------CAAAATT

G-------T-GACA----AA---ACAC-TAAA---TATT-TA-A-ATA-AA------------CAG----AGAGATAGGC--CATATT-TATG--G----

GTAG--GA----TGAAAT--TT--------TGG-----G---G----AAAAGAAACTT-CTACATGATGG-A-T-ACATG-GG-AG--AG-GCA-T--GG

AT--GA--CA-TCAGCA-----------------------------------------------------------------------------------

----------------------------------------------------------------------------------------------------

----------------------------------------------------------------------------------------------------

----------------------------------------------------------------------------------------------------

----------------------------------------------------------------------------------------------------

-----------------TTTACAAACTTGGTTCCTTCTctttttgctaccctcccctgcctcactgggtatgaacctggGAGTTGGGAGGCCTTTTGCAA

ACAGAAGCATTCTACCTGgatctgcaggacacattgcacacCTCCATATGATTTCACTTGGAAAGAGACTTGAACTTCTAtataaaa-------------

CCTTG----GAGACCACCATG----ATCTA---CCTGCCC---ACAACACCAATATTCCAGGGCAGCAGGCCTGATGATTCCTGGGATCTATCATTCTGC

CAAGGCTCTGTGGaactctCTGGGGATCTAAAGGACCCTTCCTGGGTTGGAtgcatgcaagcta--AGTGTGATGGAGAGCACAGGCAAAGCCTTTGAAC

CCATCtgagt-CTCCTGGTACCCACAGAGGGGCGGGTCACTGCCTCTCcacTCCACTATGACCCCGGTTTCCCTTCTTCCTTTCCTACAGAtcATTTTCC

ATTTCAAAGAAATCTGATCCCTTAATAacccaatgagatcacttCCTACGCTTTGTCAGGCACAATAC-ACACCCTTTCACTTCTGCTCCACAAACTCGA

AGCAAAACATGGAAACTTTGCAGCTTCCTT--------ATCTGGAAATCACCCCACG--CTGATATCTACTCAGCAAGCACACGCAGGGTCTTGCTTCAT

CCTTCTTTGATTG---CCCTGTCAAGGCTCAGT--TCCCTATAAAGGGCAGGCGGGGCCCCCaGAGGAGCAGAGAGGCTGAGACCAACCCAGACACCTGC

Agctctccctcc-aagctcggctccttgagtgccagc--ATA----------CAGTGCTTCTGAGCCTGCAGCTCGTGGTGGCCGTCTTCAGCCCCCAGG

TGCTCACCTAGCCAG-GTgaggctcttccttcgctaagtcttagcacttgagctacctcagctaccaccatccaagtgggagccatcccagacaggctgc

atcttcttcattatgaagaaggagagaggccaagacaggagccaaaaaagagccacctcccatcagagccacaacttagttccgaggttCTGAGGCCAGT

CCCTACGATCATCCTTGACACCAGCTTTGggGAGTCAGGCTGAGTGCAGCCACTACGTTTACG--CCGCC--TTGTTTGGGGAAGTGGTACAGAAGGAAG

CACCATTTCTggtcGTGGGTGTGGAAGACTTTTCACGCAGCAGTGGGGAGGAAGaagtccctgcctgtggtctcacattttgGGTTATGGTCTAGTGTAG

CATCCAGCACGGTGGCTGTGTAaggaggataagcacCCCCGAGGAATCTCAGCACATGACACAGGTTAGCTACCA--GCCCACGGGGTCGACTAGCCGAG

AACCAACGCATGTGCAGCTT-TG----CA-------------TGGGCCAACAGGGAAGAGC---CTTCTTACCCAA---GTTTCCCGGGATCCA-CCAAC

A----CGG-CTG--G-GTT--GT-G--------------CGGGCGAGGTTTTACCTCTGGGGTC-------AG-----GA-CTTGGCAGGGTGACCCCT-

GGCCCCTTCCCACCTTCTGCCTCCTT-C---GCGGGTGCATTTTTCCTGTGTATCCT----GTCTCACTTGTTGTGAAATTTCTTTCAG---ATTCCGTT

TCCATCCCAGTCACCTGCTGCTTTGGTGCGGTCAGCAGAAAGATTCCCATCCAGAGGCTGGAGGGCTACACGAGAATCACCAGTGCCCAGCGTCCCCGG-

-gGCAGCTGTGAT-aTGAGTGCACcaggccCCCGGGGCAGCTGTGATATGAGTGCACCAGGCCAGCTCTCCCAAACTTCTCTCTGAGAAGCAAGGGAAAG

TGACCAGGATTCACAGCCACATGAGCCAGATAAATA-GAC--C-ATCTAA--------TCCGC-----AGGGACATTCACCCCAC-A-AAGGAGTCCACA

GTCGCCCCAGGC-TCCCTTCTAGGGGCTTGGTGAgatggctccaggtgcttcAGCCAGGAGCCTGGCCGGTGTCACCTGGGCAGCAAGAGCAgaccttcc

tctagaagccaccctctgcctcccctccctcactcctggaccaggcctc-------TCACCCAAGGAGCAAGGGCTGGCTAGGTCTAGGaccccctgggc

cacacccctgggcggacccctcaagaggctcacctggttgtcccctttctcttg---CAG-CTTCAAGACTAAGCTGGCCAAGGAGGTATGCGCTGACCC

CAGGGAGAAATGGGTCCAGGATTCCATGAAGCTCCTGGACCAAAAGTCCCTAACTCGGAAGCCTTGA---cctccccatgcacaccTGGACTGAGACTCA

GAGTCTGAGGCCAACATTATTTATTTcccagccttccctgggtactgtaggacagtacttagttatataatgtaccgaaaggagggttctatttaatcAT

TGAAAGCACAGTATTTCTTAGATAATATTTAATTATATtaaagttctggatgtttttgtctctttccaccatgaactcctgtgatggcaagacacaaagc

cctggcgatgtgtggcttttactttttaatCTGTGAGCCCAGTTAAGTACATGGCAACACGTCCGTGTTtgctttctgggctgcccgtggcgctgtgagg

ttcttatgaggactggcggtataTGAAACACTTCTGTATTCTTAAGGAATTGGTGCTCCTtgaagctgtgtacttttgttttgttg------------TT

GAAGTTATTATTACTGACTATGGAATTTTCAAAGAAAA--------------------ATATAATTTTAAACTACATAGACTTATTTTTCATGGGGTAgc

atgtaacacacagcaatgggtggcagtttggggcctctccaagaacgcctttggctgggggtgactcagttgagatcagggagagtgggcagggccagcc

cacgcagccctagtccttggcctccagtccccagcgcccctcaccctgagtcacgagggtttatatttcgaagtcatctctcaccacaccagtacttgcc

tgaggactctctcctgatcttcagtctttaaacttttctccatctctcaggtttttctccatctaattagctctcagaccctgagcaacatcccaggggg

caactgggccagtgaagaaacttggtatctggatcccagatccatacagggcttttcattgacaacgagtacccaacacacctagtctttttttttttta

agatttatttatttatttgaaagtcagagttacgtagcaagaggagaggcagaaagagagagaggtcttccatctgatggttcactccccaattggccgc

aacagctggagctacgcccatcagaagccaggagccaggagcttcttccaggtctcccacatgggtacaggggcccaaggatttgggccatcttctactg

ctttcccaggctatagcagagagctgaatcaaaagtggggcagccgggacttgaacaggtgcccatatagggatgcaggcactgcaggtggcggcttcac

ctgctacgccatagtgccagccccaatgcctgatcttaaaggtagtgtgtctgatacagagcacgtggagcgagctcctcagagatcgtgcaggaggaag

ggggatacatagagagggaggaatcccacccacctgggctctgaagcagtggaaatacagattggagggctgatgtggcacagtgggccaggcagctgcc

tgcgatgcctacatcccatataggagtgccagcgtgagtcccagctgctgcacttctaattcagctggaagccatcaggttatgactcaagtgcttgtgc

cgctaccactcacgtgggaaatccagatggcatttctggctcctggctttggcatggcccagccctggttgttacaggcattgaggaagagaaccagcag

atagaaagctctctctctctctctctcaatctacttttcaagtacatgagaataattttttaaagatttaatttattttattggaaaggcagagtcagag

agagagagagaaggagaagggagagagaagtcttccatccactggttcactccccaaatggcctcttcaaccaaggctgggccaggccaaagccaggagt

caggatcttcttccgagtcgcccacgtgggtgcagatacccaagcacttgggccatcctcccctgctttcccaggagcattagcagggagctggattgga

agtggagcagctgggtctcaaattggtgcccatatgggatgccagtgtgaaataattgttggagataaactgtagggggtcttgatttcagagtgggaag

taatagggttcccgaaagatccgaaataacaaagaaggagagacactctgaggcatgcataaggagacacctgatgtctgagtgcaggtgaatgagggag

ggacagcttgggagaaagccatgggggtgatgcccaagagaccatgaggatctacgtggggatgaagcttaggaaaatgcagcaggcagagggatgacat

gttggatgttgtgcaagaggggaacagtagacctgagacataggcccatcccttacaacctcacaaagcagctcctaattagcttatcttggggccaatg

ctgtggttcagcgagttaaaacccaggcctgcagcactggcatcccatataggcaccagtttgagatccaggtgctctactcctgatctagctccctgcc

aatgtgcctgggacagcagtggaggatggcccaagtgcttgagcccctgctcccatgtgagagacctgggctcctggctcctggctttggcctggctcag

ttctggctgttgtggccatttaggggatgaactagcagatgaaagatacctctctgtctctctctctctctccctctctgcctctgtctctctctctctg

tgtgtgtgtgtgtgtgtgtgtgtgtgtgtgtgtgtgtttccttctctctgtaactctacctttcagataaataaatcttttcttaaaaaagaattagctt

ctctctgctatggcatgaatgactatgcccctacaaagctcatcctaatctaatccccctcatcctagtccacctggggttgccattcctaatacctgag

tttggggactgcataaaaaaatgaggggtgggagctgtggcatagtgggtaaagccacagcctgcggtgccggcatcatgtataggaaccagcctagtcc

cagctctctgctatggcctgggaaaagcagtagaaaatggcccaagtcttggggccctgctcctgtgtggaatacccagaggaagctcctggctcctggc

tcctggctttggattggcacagctccagacactgtggccatctggggagtgaaccagccgatggaagacctccctctctctctgcctctgcctctctgta

actctgccttccaaataaataaataaatctttttttaaaagagaaacaaacaaataaataaatgaggattattcggctcatgattctggtggccagagtc

tcaggccgcatcacaccatgacagcaatggctacagctgtgtggagacgggccccacactgcgacacccagcccactgcacaagcccagcaccgatccgc

tcatgagcatggggccgcatcacccccatttccagtcctcctcttaaggttctgccaccaccacaccccggcgctggagagcaagcttgcagcgcgtgcc

aaccgccctggcagcagttggcagaggcgtcagggggcagtgaggcgccgaggggtctgccctcctgagtgacgccagtgcatttataaagaaggcctgc

gggggccggcgaggacacgcggaaggcgccatctgtgcagagcaggcccctctctggcccccggtccgcggcagccttcctcttggacctcccagccctc

agaactgggagcagcatgtttgcgttgtctgtaaatgaccccgtgtcagctgcagaaaggattgagacactctccatccccacacctccagccggaccct

gagagcttttgccggtatccattcaaaggcacacgcctcctcttcccggCAGCTTCTTGCGGTCAAGGTCTCGCTGAACTTCTTGAGCTcagccgcttct

cctgcagacgtgtgactcaccatggtgaggcacccggacaagactccccacggagagaagggagcttcctatctcgtgagccagctcctcagggcctgcg

gtccaagacggatgtgcagaggctcaacaggcttggaattcagcacgctgggaagcagaggcggcgggagacctggatcctcatctccaagctgacccaa

ctctgagtcactcaggctaagtggacatccccgcctgaccttaggatggcccctcccgcagtgagggtgacccctggccacaggccggagccgcggaatg

ggcggtctctgacaagtctctctgacttcacgcccatgtctcttctcttggaaacacggcctgagctgttggctgggcagagtgggaagttaaaatgtct

gca------------------------ATTAAAACCCTGGAGCCAGCCAGGACCTAGATTATTGAAAacaaaaacaaaaacaaaaacaaaaacagctcac

atagcagacagacctgcagataagctttcagaggtcactccagggagctcctgaagccccaagagcagtaggcagaggagggccctttccaatcctaacc

tgtctcttccgcagcttccaggaccaatgggacgtggagatgggggtccagattttcacaaatccca-------------------------------GA

CCACAAATCCAAAATCCCAAGGGCAGGAAGACTCACtctgtgctgacaccacctgttggttttctccaaacc------------------TTACCCAGCT

GCACTGTAGAATTTTGTCATTACATTTAAAtcccagcagcgttaaccactgcagaactcaggtaccacgtcatcattttaaatgatgcttcactgccctg

accagaatagcagtatttttag-AATCCACATCCTTACGTAAACTGGTTGGAATCTGTTAGCtgccagtcactcccttccttgtcgaccaagggcaaaga

agctgggtggtctcgggaaggcgacagggcctctggggctcggtggatggcttgcggcaggctgtgactcactctactctcttcacatcagctctcagcg

cccctcacgccttgctggtggtgcacaggctccccctggtgccctgtcaggtcaggagtgtgctcggcctgtatacttgcctgaggcaggaagttcctga

aagagagcacgctgggaggcagccatccccccaggcagggcagggacagctgctttctgctctcctgcctctggctgttgattctggaagtcaggctgtg

ggctgcccacagtgtccctggaggatcggtgcgcagcgctcaccacgttgaccctgaccatacaacttgccctggcttctcctccatcctcatctcagtc

ccgatctctgaggtgccccagtgaactgtctgcatgcaggtcctcactccgtctctgtttcacagattcatctgttcattattct---------------

----------------------------------------------------------------------------------------------------

----------------------------------------------------------------------------------------------------

----------------------------------------------------------------------------------------------------

----------------------------------------------------------------------------------------------------

----------------------------------------------------------------------------------------------------

----------------------------------------------------------------------------------------------------

----------------------------------------------------------------------------------------------------

----------------------------------------------------------------------------------------------------

----------------------------------------------------------------------------------------------------

----------------------------------------------------------------------------------------------------

----------------------------------------------------------------------------------------------------

----------------------------------------------------------------------------------------------------

----------------------------------------------------------------------------------------------------

----------------------------------------------------------------------------------------------------

----------------------------------------------------------------------------------------------------

----------------------------------------------------------------------------------------------------

----------------------------------------------------------------------------------------------------

----------------------------------------------------------------------------------------------------

----------------------------------------------------------------------------------------------------

----------------------------------------------------------------------------------------------------

----------------------------------------------------------------------------------------------------

----------------------------------------------------------------------------------------------------

----------------------------------------------------------------------------------------------------

----------------------------CCTTCTCCTTGGCCAATACCTGGATCTCAAccaggaggttaatggaggcCAAGGTCAGGAACTCAGCTGGAC

ACGTGATTGGTGTGTTg-CCAGGGCTGGGACTGGAACTAGGACTGGAGCCTTACCCAGtgtgtttttcagggccaggctgagttcaagagagtggcccac

aagtcggggctg-------------------------------------------ACAGGGCTGGGTCTGAGTTAGCCACATAGCTAGGtgacataaaca

tgggttgagacaacattgacatctgtcaagataacggaagccaactcagtgctggagaagcttccaaagcaaaatatagaaatttggaacattggggaaa

actttgtgatgttatctggaattaggtattgtatgaactcatagtgttaaggatatagattgattaatagataaatgattgataggaggaaggagggagg

gaggaaggatggcgagggggaagaagggagcaaatggagagagagacaaaggtagaggtagagatgagcgatgcacactga-------------------

----------------TTATGTTCACTGGAAAAAGCTAGAGACAATGACACATGagtatcagcgtatacatcaagcGCCCATTTTATAAAAACCATTCTC

CACTAAAGAAACCAGAgatccttcgatccacGGCTCACTCCAGAGCTGGAATATCTGGTTGTGTCAAAagagaaagaagttatttttaaaagtaatgaaa

tgtgtcaaagacacatagaacccaatttgaagagact---------------------------------------------------------------

----------------------------------------------------------------------------------------------------

----------------------------------------------------------------------------------------------------

-----------------------------------------------------------------------CACTGGCCAATCTGGGACAATCTAATCAC

CAAAATAAATAcaaatttaaaaaaccatAATAATTTATATTAATCTATTGAATAAAGTCAGAGAAGggAAAGTTCTAACTTAGAGTAAAATGCTAACCAA

aaaacagaagaaagcatggagtcagacaatcaccatttggaag--CCATCAGAGTAAAAAATTATTCAGGCAGGAATCATCTATGaATCCTAAAACAAAT

GGTTGAAAATTTGATTAggaacagactattgATAGCCTCAAATTATCTCCCAACAcaaacaacataacaatcaacctgtacaatgaagagaattgcagg-

-----------------------------------CAACCCTTTAATCACATAATAAAAGAGGCCATCACCAGTAgcagaacaagtgtacctcctggtac

aatgcactgagaacaaacatgtcttcagc-------------TACTCTATTGCTAACAACACGTAAACTAGATCTAATCgctaggaaacaaaacaaatca

aactgagaaaaatcctacctagaaaccggcctcactgtcaaggtcatgagacg--------AAGACTGAGAAACTGTTTCACATCAATGGAGACTAAAGg

accatgacagcgtggtctttcattggatccggaagctatacagaaagtcactgggaaaacgagcaacactggaatggagagtttgaacagatggaaatgt

tgggtctgtgtggacatcctgattttgatgtttggaccattgttccagagaagagtattgaagtggtaaggtgttattagtcgtt---------------

---------------CATGTTGTCTGCAGTTTAATCTCAAAGGGGTCAAagaaaaaaaacctctctacgaatagatacatagatctgtgtcatataaatg

agagtgcttccaaaagtccctggagtgctgggtgcttgctgcagccattaagattctgcttggaacacctgtgtcccttgtcagagaatctggctctggc

tctgattctggctccctgctgatgcacacctggcaggcagcaggtgatggcgcaagtggttgagtttctgccacccaagtgggagactcagattgatttc

ctacctcccaggctttggggtagtgaactagcagatggtcagtctgtctgtctctctctcataaacaaaatattttaaattagctttttaaaaattcatg

gaaaacagaattcaaagataacggtcattttccatgattgttttgaaacaccctcatacgtagcagatacacacacacacacacacacacacacacacac

agttacttccaaaagtttgtggaggcctggcattgtggtgtaacaggttaagccgccgcctgcaatgaccgcattccatatggactcgggtttgtgtcct

ggctgttccgcttccagcccagctccctgttaatgcacctgggaaagcagtggaggatggaccaagtactcggacccctgtaccctcatgggagacccag

atgaagctcctggctcttggcttcagcctggcccagccctaaccagccattacctccatgtagatagtgaactagcgatggaagacctctctctctctct

ctctctctctctccttttctctctctctataattctgctttccaaataagtaaaataaatcttttttataattcttggaaaattagtattatggaaagaa

aattcatgaatttcgaattttttttgcaccaaaataaactttttattccattttccacaaactttgggaagccccctcagtaaaggtatttgtaaaagtt

tgcaggaaaatagaattgaaagggcaaacatcccgtgaactttgtggagcccctgtgttcacaacaactcaagctgttgcacccctggtgctgcagccct

gtgtagttgtttagacttggctgttctgatcatgtgtgtggttccacctcagtgtgatttaaattcgcatttcattccaaggaggttgagctctatcaag

atgtttgcagaatgttctcttttatgaagcattcatgcatatcttttgtctacattagagTGTCTGTCTTTTAATTACAAATTAGAAGTGTTTTCTACAc

tctaaataggaatgctttgccaggcatccaaatgaaacatcttctcatagtgtacaaattgcctttcattcacttcaggatgctttttgatg-------A

AAACAGATCATAATGTTCATATAGTCCATCAATCATtaggttcctttatggttggtgCTTTTGTTTTTTGTTTAAGAAAACTTTTTAAActtt-ATATCA

TGAAGATATTCTCCTGTGATGTCTTCTAGAAgtttttttttt-------TTCGCAGTTAGAGATACAATCCACCTGGGATTAATTTTTGtctgaaaggaa

agggccaagattttattattccacataactaatccactgacacagcactagtgtttcaaaagacttttcccccaactattcc------------------

----------------------AGAGTGCAAGTTTTGTTGTAAACCTGATGTCCATATATGTagagtcctacttgtgtaagtttaccggttatat-----

----TTCTCTACTCTTGAAGCAATGGCATGTGTTTTAACAACTgtagCTCTCTGATAAATGTTTATATCTGATAGCATAAGTTCTAtagttttttt----

----TTTTCTTGATTGTTTTTGGCTTTCTATTTCGCCGTAcaatgtttcgaatcagagtgaggcgtttggcacagcagttaagttgccacttgggacacc

cacattcttcatcaaagtgcctggattgaagtctcagttactccacttccgagccagcttgctgctaatgcacaccccaggaggcagcaaatgctggctc

aagtacttgggtccctgtcccctctgcgggagacacagtaggtgctcctggctcctggctccagccctggctgctgcaggcatttagaaagtgaactagt

ggatggaggacctcccccatttctctctctctccctctctccctccctccctcctttttatcccctccctccctcatctctccctccctttctctcttct

tccctccctccctttaaaataaaaagaaaatagatcaattttaaaaataaaatatagaattAGCTTATCAATTTCCACAAAATACTTGCTTGGATTTTAc

tgggtattgcattgaagttggggaagagcttacaacattggtatcaacttttccaaattctgtgtgtggcatattcaagtctcct------------TTA

ATCTCTCTAAATAATGTTTAATAGTTTTCTACATATTataaaggtcttatcatcaggTTTATTTTTAGGTATCTGGGGGGTTTTTAGCTTTTGGAAAtca

tgttaagaaattttcttttaaatttttacttgatttatttatttgaaaggcagagagagaaacagcttccatccactgactcactcctcaaatgctggca

acagccagggctaggtcaaacagaagacgggagccaagatctcagtccaggtctcccatgtgggcagggacccagttgctggagtgatcacctgctgtct

ctcggcattagcaggagtctggagaaggaggctggtcttgaatgcagacactcagatacgggatgcaggcatcccaagcaggacttgaccactgccccag

atgcctgccccaagaagttagaatagcctgatctgtagactctttcagtaacatttcttgtaagcaatagagGCTTTACGTCATCCTTTGCCATCACTAT

AACTTCAGTTCAgaatatcctggtacatttgctaggacctcatgggcgtggc------------------------------CCTGTTCTCCAAGGGAAC

GTTTCACGTTTCTACggaat---GTCACGCTTGCCCCAGCTATTCTGTAGATGCTATTTGTggggTTAAAGAAGTTCCCTACATGTGCTAGCTTGTTTAG

GGTTTtcttgtgccgtaagtggtttttgaaatttaacaaatgattc------TTTGCATTCAGATGACCATAAGATTTTTtttctgCTTTACTCTGTAGT

TGAGGTGAAATGCATTCATTGTTTTTccacttcagttagc--------ATTTTAAAAATCAAACtaggggctggtgctgtggcaaagtgggtaaagccag

cacctgcagtgccagcatcccatatgggctccagttcgagtcctggctgctccactttccatctagctctctgctatggcctgggaaagcagtggaagat

ggcccaagtccttgggcccctgcacgcatgtgggagacccagaagaagcttctggctcattggctatttccagatgccattgctgacatgtggttaccta

tgattaggaaagacacaggtccacatcttccttccggaacaTCCCTCACAGAATTTGGCATCAACATTGCACTGATCTTAgaaaacgaactgaaaagtgt

cccttgttttattttcctcaaaaatacttttgcaaac--TTCATGTTTTTGCTTCCTTTAATGTTTGGAAGCAAATCATcctgggatttctctataaAAA

GATTTTTAATTACAGATTCAACTACTTAATAGAtattgaagtattggaatgtgctatgcattattggttaactttacttaggtctgttcccCTCAAAATT

TATTCATTTCATTAAATTTTCAAATACATgggtctagagttgtacacagtatcTTTTTATCATTTTATAATGTCTGTGGTTTCTGtgcTAATAATATCTC

CTTTTTCATCTCTGATATTGCTAACTTGtgagtcacaga-TTTTATTCCTTGATCAGCCTTGCAAAAAAATTAACATTTTag---TCTTTTCAAAGAACA

AACCTTTAGTTTTGGCATTTTTtctcattgtgagtttgctttg---TTACATTAATTTTTGCTTTTATccctgttttttatctcattctactttcagggg

gcttgattttctatcttctatttttaaacaaatgtacagattatgGACTTTAAGTCTTTGTTTTTAATGTACACATTTAATTtctttctttct-------

----------------------------------------------------------------------------------------------------

----------------------------------------------------------------------------------------------------

----------------------------------------------------------------------------------------------------

----------------------------------------------------------------------------------------------------

----------------------------------------------------------------------------------------------------

----------------------------------------------------------------------------------------------------

----------------------------------------------------------------------------------------------------

----------------------------------------------------------------------------------------------------

-----------------------------------------------------------------------------------------------TTCTT

TTTTTTTTTTTTTgcttcaatcattgcttaaaaaatcttcaaaacaccttagtgaggctcatgacagtgccagggcgtacggagatatagccctctgcag

ccttctagacactgtcacaccatctctgactacgcccttggccttctccaggtgtagaccagcaggtgtgcatcctttcagacaagctgagccctgcctc

tgccagttggcgtaaggccctgcttctctcagtagtcaccataagtcctaaccctaaccctaaccctaaccctaaccctaacaccataaggcagtgatag

tgaatacacatttaatttcacaaaccttctatacacttctatagttcactttaagacataaattttgatgtgacatgttttattataatttattt-----

----------------------------------------------------------------------------------------------------

----------------------------------------------------------------------------------------------------

----------------------------------------------------------------------------------------------------

----------------------------------------------------------------------------------------------------

----------------------------------------------------------------------------------------------------

----------------------------------------------------------------------------------------------------

----------------------------------------------------------------------------------------------------

----------------------------------------------------------------------------------------------------

--------------------------------AAAATATATTCCCATTTCTATTATGATTTCTTCTTTAATCcatatgttgtttagatgaacatgactac

attttcaaatttagctatattgcaagaagtaccCATTTTGTTATTGGTTTTAATTTAATTCtgc-ATGGTTAAAGAACATATTTTATagtttttagactt

tttctatccactgggacttgctttgtagtacagcatatAGTCAGTTTGATAAATGTTCCctctttttctctctgcctgtctctctgtctgcctataaaat

aagtaaaataaaataaaataacttttctaaaaagaacatttcttaagtgatattatgtccaatagctacaaaactcacaaaagccatttgataaatgttt

catcctaaagctgttagatgctggcatgtcatgtggatagcttctgtgaatagagtagttctaatcttaAATGACATTTTTAGAAAAAAAAAAAAAAGCa

agtgaatcttgtt---------ACAATACTTCACTGTAAGAAAAACTAAAGGcttaagaGAAGTGATTGCACTGGCGACACAGTTCTTCAGAAAGTAAga

-------------------AGGAGCAAGGCCTGCAGGGTCTCCTATGTCTTTTttttaaactatttatttatttatttatttgaaagtcagagttacaca

gagaaggagaggc---------------------------------------------------------------------------------------

----------------------------------------------------------------------------------------------------

----------------------------------------------------------------------------------------------------

----------------------------------------------------------------------------------------------------

----------------------------------------------------------------------------------------------------

----------------------------------------------------------------------------------------------------

----------------------------------------------------------------------------------------------------

----------------------------------------------------------------------------------------------------

----------------------------------------------------------------------------------------------------

----------------------------------------------------------------------------------------------------

----------------------------------------------------------------------------------------------------

----------------------------------------------------------------------------------------------------

----------------------------------------------------------------------------------------------------

----------------------------------------------------------------------------------------------------

----------------------------------------------------------------------------------------------------

----------------------------------------------------------------------------------------------------

----------------------------------------------------------------------------------------------------

----------------------------------------------------------------------------------------------------

----------------------------------------------------------------------------------------------------

----------------------------------------------------------------------------------------------------

----------------------------------------------------------------------------------------------------

----------------------------------------------------------------------------------------------------

----------------------------------------------------------------------------------------------------

----------------------------------------------------------------------------------------------------

----------------------------------------------------------------------------------------------------

----------------------------------------------------------------------------------------------------

----------------------------------------------------------------------------------------------------

----------------------------------------------------------------------------------------------------

----------------------------------------------------------------------------------------------------

----------------------------------------------------------------------------------------------------

----------------------------------------------------------------------------------------------------

----------------------------------------------------------------------------------------------------

----------------------------------------------------------------------------------------------------

----------------------------------------------------------------------------------------------------

----------------------------------------------------------------------------------------------------

----------------------------------------------------------------------------------------------------

----------------------------------------------------------------------------------------------------

----------------------------------------------------------------------------------------------------

----------------------------------------------------------------------------------------------------

----------------------------------------------------------------------------------------------------

----------------------------------------------------------------------------------------------------

----------------------------------------------------------------------------------------------------

----------------------------------------------------------------------------------------------------

----------------------------------------------------------------------------------------------------

----------------------------------------------------------------------------------------------------

----------------------------------------------------------------------------------------------------

----------------------------------------------------------------------------------------------------

----------------------------------------------------------------------------------------------------

----------------------------------------------------------------------------------------------------

----------------------------------------------------------------------------------------------------

----------------------------------------------------------------------------------------------------

----------------------------------------------------------------------------------------------------

----------------------------------------------------------------------------------------------------

----------------------------------------------------------------------------------------------------

----------------------------------------------------------------------------------------------------

----------------------------------------------------------------------------------------------------

----------------------------------------------------------------------------------------------------

----------------------------------------------------------------------------------------------------

----------------------------------------------------------------------------------------------------

----------------------------------------------------------------------------------------------------

----------------------------------------------------------------------------------------------------

----------------------------------------------------------------------------------------------------

----------------------------------------------------------------------------------------------------

----------------------------------------------------------------------------------------------------

----------------------------------------------------------------------------------------------------

----------------------------------------------------------------------------------------------------

----------------------------------------------------------------------------------------------------

----------------------------------------------------------------------------------------------------

----------------------------------------------------------------------------------------------------

----------------------------------------------------------------------------------------------------

----------------------------------------------------------------------------------------------------

----------------------------------------------------------------------------------------------------

----------------------------------------------------------------------------------------------------

----------------------------------------------------------------------------------------------------

----------------------------------------------------------------------------------------------------

----------------------------------------------------------------------------------------------------

----------------------------------------------------------------------------------------------------

----------------------------------------------------------------------------------------------------

----------------------------------------------------------------------------------------------------

----------------------------------------------------------------------------------------------------

----------------------------------------------------------------------------------------------------

----------------------------------------------------------------------------------------------------

----------------------------------------------------------------------------------------------------

----------------------------------------------------------------------------------------------------

----------------------------------------------------------------------------------------------------

------------AGAGAGAGAGAGAGAGAGAGGTcttccatcagagaaataggaccaatcgaatatataaaatacacagagaaagatttactatacaaac

ttagttcacgtgattatcccaaatctgcagtgagtcagccagctggagatgcaggaaagtcgatggtacagatccaactcccaggactcggagccagcag

atgtggagatgtggagccactctgcaagccagccagctggagaccttggaaatgccaacctttcagctccagtgtcaaggcttggggaaagaataaataa

aataaaagctggtgccctaattcagaggcagttggatggaagaattctcttctacatgggggaagagtcggccttttatatctactcagatcttcaaccc

actgggtcaggtccattcatggggtggaatgaggaggtgaaaaggTCACATTAGGGGAAACAATCCACTTTAttcagcgtgaaactgtgtttggatttca

tctccatttcctggcacacagctcctaaaaccctcggaatctctaaagtggtggcatctctttggatgccaacgagttgtctgacagctggttgcctctg

ggcagcttcaggatggggcatggtcaccagaaaacccagggcaagattagaaggttggggctttcagctccatcccctgccttctagggaagggagaaga

gttcaagtttaagttgatcatcagtggcccgtggtttaatcatgtctatggaaagaagatccaaaggaacacacaaatagggctcaggggacttccagat

cacagaacacgtggaggctccgggagggtgggggtctcccgggaaggcatggaaactccatgatcctccccccaaccctcgcccagtcatctcctcacct

gcatcttttgtgataccctttataataaccaggaaacctaaacaagtgtttctctgagttctgtgagctgctcaagcaaattattccagctaaggaaggg

ctgtaggaactcagtttattgcacaggtaaagctaaagcctgaagcttgtgattggtatcggacgtgggatggagcagtcttggggatttgccctccacc

tgtgggatctgatgctgcctccagctagggaaagaatcgagttgaattgggggacacccagctggcatcagctgcagaattgcttgcttgctgagaggta

gaaatctctgtatttttgaggacacggaagtgttctgtgttgactgttgtggtgtgagagcagacaaaaagctgtttgtttttctgatacacataagtgt

taatctcattaaaaagcaccattgTAGAAACACCCAGAACAATGTTTTACCAACTATctagatACTCCAAGGCCCAGTGAAATTGACACATAAAATTAAC

CATcatcatgtgcatgcacacacacacatacacacacacagatatgtgcacATTTGTTTTTTGAAGAAGTTGCCAATGCAATAAAGAAAAAgaaggccgg

cgccgcggctcaataggctaatcctccgccttgcggcaccagcacgccgggtcctagtcctggtcgtggcgccggattctgtcccggttgcccctcttcc

aggccagctctctgctgtggccagggagtgcagtggaggatggcccaggtccttgggccctgcaccccatgggagaccaggaaaagcacctggctcctgg

cttcggatcagtgcagtgggcaggccgcagtgcgccggccgtggcagccattggagggtgaaccaatggcaaaaaggagggcctttctctctgtctctct

ctcatactgtccactctgcctgtcaaaaataaaaaaaaaaaaaaaaaagAAAAGTATCTCAAGCAAATTATACTGGAGCAAGCAGACATgggaaaatgta

aacctgtgccttgtatcgcactacacatacataaaatta---AATTTAAGATGTATCAGAGACCCAAATATGAAAGCTtcatccAGAAAACACCCAGAAG

AAAACATGGAAAAGCTTCCTTAtcacttagaatttttttttaaaaaaagattttttaagcgaaacttaaaacatctaatcatcaaagaaaaaatacatgc

aaactggacctcATCAATATTGAGAAGAACCATTCATCAAAAGATACCATtaacaaaaagacaaaccaacacctacgtgaagacacaccaaatagtttat

ccctcattatatgaagattctgacacattagtaaggaaaagggaaaaaatttaaaagtgggcaaaaaaatgtgacaggcatttcacaaaag---------

----------------------------------------------------------------------------------------------------

----------------------------------------------------------------------------------------------------

----------------------------------------------------------------------------------------------------

----------------------------------------------------------------------------------------------------

----------------------------------------------------------------------------------------------------

----------------------------------------------------------------------------------------------------

----------------------------------------------------------------------------------------------------

----------------------------------------------------------------------------------------------------

----------------------------------------------------------------------------------------------------

----------------------------------------------------------------------------------------------------

----------------------------------------------------------------------------------------------------

----------------------------------------------------------------------------------------------------

----------------------------------------------------------------------------------------------------

----------------------------------------------------------------------------------------------------

----------------------------------------------------------------------------------------------------

----------------------------------------------------------------------------------------------------

----------------------------------------------------------------------------------------------------

----------------------------------------------------------------------------------------------------

----------------------------------------------------------------------------------------------------

----------------------------------------------------------------------------------------------------

---------------AAAATATTCAAATAAGCAGTGAACACATGGGAAGGAGCgccacactgtgtgtaatcagggaaatgcaagtggagggaactcaaag

cacctccaccctccgcgatggctaagtcagaaaagatgcaagatgtccatgtggctggcgtggaaaaggtggaaataccaagtgcagatggacacgcaga

gcaaacagagggcctctacagtgccgggaagccgcatgtcagtttctagcaaagcTAAGCATATGCTTGCCCAATGGCTCAGCAATTCCTCTTtaagtaa

acagtttagatagaggagtcaacacatacacAAAAAGGTTTGAACAAGATTAGTCATAACCGCTCTGTTCAcagcagctcagctctggaagcaccccaaa

cgtccagagcgggaaagcaagctgtgtatatcgtaaagtgaaacatgtttttggcagaaaaaatatcaaagtattcacatgttttttatttagttatttt

ttaaaggtttattttatttatttgaaagacagagttacagagagaggtagagacagagagagaggtcttccatctgctggttcactccccagatggccac

aatggccggagctgcaccgatctgaagccaggagccaggagcttcttccaggtctcccacatgagtgcaggggcccaaggacttggggccatcctccact

gctatcccaggccacagcagaaagctggataggaaaaggagcaactgggactcgaaccggcaaccatatgggatgccggtgcttaaggccagagctttaa

cccactgtgccgcagtgccggcaccttatatgttttttaaaaaccatgacactgaggggccagcactatggcgtagtaggctaagcttctgcctgcagta

ctggcatcccatatgggcactaattcgtgtctcagctgttcttcttctaatccagttctctgctgtgatctgggaaagcagtagaagatggcctaagtgc

ttgggcccctgcagccacatggaagacctggaagaagctcctggctcctggctttggatcggctcagctccagcccttgagacttttgtggagtgaacta

atggatggtagacctttctctgtctctcggtctctgtctctctctctgtaactctgccctccaaataaataaataaaatcttaaaaaacaagcagtcaca

cataaaagactatac-------------------------------------------------------------------------------------

----------------------------------------------------------------------------------------------------

----------------------------------------------------------------------------------------------------

----------------------------------------------------------------------------------------------------

----------------------------------------------------------------------------------------------------

----------------------------------------------------------------------------------------------------

-------------------CCATGTGATTCCACTTACGTGAATTTCAAGAACAggccatctggttggcgctgtggctcactaggctaatcttccgccttg

cggcgccagcacaccgggttctagtcctggtcagggtgccggattctgtcccggttgcccctcttccaggccagctctctgctgtggccagggagtgcag

tggaggatggccaaagtccttgggccctgcaccccgtgggagaccagcagaagcacctggctcctgccatcggatcagcgcggtgcgccggccgcagcgc

gccggccgctgcggccattggagggtgaaccaacggcaaaggaagacctttctttctgtctctctctctcactgttcactctgcctgttgaaaagagaga

gagagaaagaaaaaaaaaaaagaacaggccatcaattgctgatggaattcaaagtagttgaattaaggtgaagcgtggcctgggaaaggacacacaagag

cttctagggaggacggaaagtccaccatgatgcgagtgagagctacacattgtataacagatctctcggtaaccaagctgcacctggaaggtcggtggat

ttattgtatgttatttatatactatatattatttatctaaaatatattatttatttaaaatcagtcttaatgtatgtagtaacccaaaatgattcacatg

gttgacccacaagcccacgtccaggtcgatgcagggggtggtgatgttagggaagggttcttacatgcactattgcgtcagctggaatcccagaggagca

gaccaaacgcccaacacgtggtgtggtttaggtgagttatggtgctgctacataacgatcactccttgacctttaagaaaatagtgtttgtcaaacaaat

taaaaggaattatatcctactatagtttcaatgatttttgtgactttgttaaaatttactttatatgagcaaaattcatttgattattttttttaagatt

ttatttatttatttgagaggtagagttatagatagtgagagggagagacagagagaaaggtcttccttccgttggttcaccccccaaatggctgttacgg

ccggcaccgcgccgatccgaagccaggagccaggtgcttcttcctggcctcccatgcgggtgcagggcccaagcacttgggccatcctccactgccctcc

caggcctcagcagagagctggagtggaagaggagcaaccaggactagaacccggcgcccatgtagaatgccggcaccgcaggcggaggattaacgaagtg

agccatgctgctggcccccatttgatcattgtttatagcccttgcctgttttcctgctgttctattgtctttttattttttatttgttcaactctttatt

taataaagccattaagcctttgattataatgtaaattaaaaatgttatctcaaaaaatcgataaaacagttgaaaaacaaagaaattaTTATTGAAAGAT

GTAAAATGACTTTTGAtaatatttttgacatgatagtaactaacaaatcaagactaacccatatttgataaaggccctgatattttcacataaaaggaaa

acattttccctttagctctgtttcagtgtgGAGTGTGCAAATTGTTTCATAATGCTTTTATacgtcttaagtattttccaagta---TTCTAAGTCAGCA

TAGATTGTTTT---TGAAAATATAAAaacaTTTCTACAAAATGCTGCCTCAACAGAAAAGCTTCctttttttactc-ATTCTCAGAACCAGCGTGGGTCC

AATACACACTTGTTCCTgtcggtgttttccgttgagtc-TGGAAAAGGGAAGCGCCAGGTGTCCCCCCTGGGAGTTCCtccgaAAAGAAAGAGCAGCAGA

AGCAAGAAActgttctgaacagttctagactgttaatgtggcacctggcaagatgccaggccccacggctcagggagacggcacagcactcacTGCCTGG

GACAGCCCTACTCTATCCCTGACTGtttgcaaatgcatgggagagtccccgcgcttcttggtctctgggggtcgtcagtcagtac---------------

--------TCAGTCTGGGCTGTGTGTGGGCCTATGGAATGCCCAGAAAggtgacctgctttcctgagtagaggtagtaaggccttggagccagcgttgtg

gcacagcagtttaagccgcgattgcaacaccagcttccctgataagccggttcaagttccagctgctccatttccagctgctaatgcacctgggaaagca

ggagaagatggccctagtgtatggacctctgcacgcatgtgggagaccgagatggaattccaggctcctggcttcagcctggcccaggcctggctattgt

ggccatatggagagtaaaccagtaggtggaagatctctctgtctctctgtgtgtctccccatctctctgcgtataatctacctttcaaataaataaacta

aatcttaaaaagtagcgaggcatgtcctcaaccatttatttcagagtaagatttggtcccctatgaaaatccaTGTAACCTGAGAAAACTTCACAGACAA

GACATGCTTTAAAtctggccttagatagctcctgctcagaccaaacggaaggcacattagaggcgggtgggacagaggaaggtggggagggaagaagtga

ggcaggtgccctggcagtgtgttcacgggtgtttcctccagcatctcaatgccggcagtcgcttcctgggcagcttgccttgctctgagcactcccaggc

ttgggggcggggaataccaac---------------------------------TAGGGCTGACCCCCTGGGCTTTTCGTTAGTCACTCCAGCAtggagc

cctgagacggaaaggctgcactgtctccaccctctttggagcctcagagagtgaggagagagccacggagaagtgtgaagt-------------------

-CCAGCTCTGCTTCCCTGAGCAGTgccagtttcttttctttttcttttttttttttttttaagatttatttatgtatttgaaagtcagagttacacacac

acacacacacacacacacagaaaggtcttccatccgctggttcacttcccagatggctgcaatggccggagctgtgcagattcgaagccaggagccagga

acttcttccaggtctcccacgtgggtgcaggggcccaaggacttgggtcatcttccactgctttcccaggccacagcagagagctggatcggaagtggag

ccaggactagaaccagcgcccatgtgggatgccggcgcttcaggccagggcattaacccactgagccatagcgccggccccgccaatttcaatctgttgt

atatcaggacttccccattagttattctctgagagttGACTTTAGCAGGTAAACAAACACCTGAGAACCACAAtgctgcacacaatcaccagctccactc

tgcacagataactaaggtgctggggcacccaggattgcccagttccatggaaggcAGAATGCAGGGGGCGTGGGTGCCACGGTTCTACAGCAGCAtgcta

ggtcat--------GGCTTCCACAGCTCTAGGAGACTTCTGTagtgattgct------GGGTTGGAAACAGCTGGCTGCCCTGCTCTTCTGAACATGAtg

gtctaACCAGCACAGGACCT-CCAAAGCTCCCAGTCTGGCTGGTGAACCAAGGTGAGATTTCAGGTCTccacagaatactgTCCCTTCAGAGCCACCATC

AAATAATCCCAGGAAGTCTCaat---------------------------ATTTGTGGCAGCGGGTGCCCCTGGAGAAGGCTTGGcggggggacctactg

taaaAGACCTTTGGGTTGCTACATTTGACAAAAATACTGCAAGgagagcgtctat-----------------------atgaaaaatcactccgtgatgt

tctgaaatttgactttaaggatcctgcacctcgtctggcagcccacccactgcagggccatttctccatggatcctggagtccctcggtggatcaggagt

tgaggTTGCATCTGACAGCTGGGTAAGAGccgtgagtctcttctgcaggTGTTCATCTGGGGTTTCTGCCAAC-AGTCAAACTGCACCACAGTAAGCATC

TGATCTTACTTCATTTCTAGGGGGTTCCACAATCATCGCCACACACCCCTGCCAccaaaccaccctgatTACCAGCTCTGGCCCTTTGGgcccccgggtt

caccatgcccgccttgttaaatgggcatctccatttggcttctgctaccttgattccattctagcaaaccccactccatctcagtggccattcttgcagg

gacagggacagggagaaggagagggagaggaaagagagggagagggagctccaggatcatctgagtacttaccagtgtcaatgtaagttatggacactat

gaaaggaccacctggtcgactcgcccaacatcacaggaaggtagcaagtagcagctgatctctaggctcagggggtaagccatttgctccagatcacgca

accagcaatggcagcctgtgtctgacccagatcccaggctcccagccacactctcctatgtgatgttcatcaggtcctgaagtagtggg-----------

----------AAGCACAGGCAGCAGTCCTTACAGTGGAGAGGATTTGAATggctcctgcccactccctttcc------AGATTGGGACATGTGTGTGGGA

GTGGATTTTtctttgaagatttatttatttttgattttgaaagtcagagatctatagagagagggatagacacagagagcttttatatccgctggttcac

tcctcagatggctgcaatgaccaggactggaccaggtcgaagccaggagcttcttccgggtctcccacatgggtacaggggcccaaggacttgtgccatc

ttccactgctttcccaggccatagcagagagctggatcagaagtggaacagctgggactggaactggtacctacatgggatacagtcatcataggcagtg

gctttacacagtgcaccacaatgctggcccctggaagaggcttctaagggtgtcaggctgagcagagtaatccagatttggtgggctgctcctGTACCAG

GGAGAGGTGTTAGTCGTCAGCTAGATTGtggatgggggcctaggcttcaa--------------------------------------------------

----------------------------------------------------------------------------------------------------

----------------------------------------------------------------------------------------------------

----------------------------------------------------------------------------------------------------

----------------------------------------------------------------------------------------------------

----------------------------------------------------------------------------------------------------

----------------------------------------------------------------------------------------------------

----------------------------------------------------------------------------------------------------

----------------------------------------------------------------------------------------------------

----------------------------------------------------------------------------------------------------

----------------------------------------------------------------------------------------------------

----------------------------------------------------------------------------------------------------

----------------------------------------------------------------------------------------------------

----------------------------------------------------------------------------------------------------

----------------------------------------------------------------------------------------------------

----------------------------------------------------------------------------------------------------

----------------------------------------------------------------------------------------------------

----------------------------------------------------------------------------------------------------

----------------------------------------------------------------------------------------------------

----------------------------------------------------------------------------------------------------

----------------------------------------------------------------------------------------------------

----------------------------------------------------------------------------------------------------

----------------------------------------------------------------------------------------------------

----------------------------------------------------------------------------------------------------

----------------------------------------------------------------------------------------------------

----------------------------------------------------------------------------------------------------

----------------------------------------------------------------------------------------------------

----------------------------------------------------------------------------------------------------

----------------------------------------------------------------------------------------------------

----------------------------------------------------------------------------------------------------

----------------------------------------------------------------------------------------------------

----------------------------------------------------------------------------------------------------

----------------------------------------------------------------------------------------------------

----------------------------------------------------------------------------------------------------

----------AAGAAGCCTGCAGTTGATGAAGTTGAAAgggtcaaaacctccttggatgttggaatggaggccaggctcagggacaaggcttgcactccc

agggaagcccaggggacccctcctcttcagaggcatcagggagtgcttggatgaggtcatgagacttcgagggctgcacccccaagagctctgggtgctg

ttctcttgcactggcggtgagtgtggaggacgccccatctctgcggttctgcaccgtttatgcccagcatgagcagctggggacctggtaagtgtagtct

cccgtgctcaatgccaacacaggccctggcctgggccctttccacagtgctccgaaagtggaggcacagcccgatcctcgaatcccgttggaaggcctag

ttccagaggccacttcagggtacccaggcctgtgtcacccagggcctcccttcagctgggaggatatgagcatggcccagaagagacactggttccacgg

atagaggtgtgggcctggagaggatggctccccacatctggatggggccagtccccaggag---------------------------------------

----------------------------------------------------------------------------------------------------

----------------------------------------------------------------------------------------------------

----------------------------------------------------------------------------------------------------

----------------------------------------------------------------------------------------------------

----------------------------------------------------------------------------------------------------

----------------------------------------------------------------------------------------------------

----------------------------------------------------------------------------------------------------

----------------------------------------------------------------------------------------------------

---------------------------------------------------------------------------------------------CCACCTG

GAGAGAGGATAAAGCCATCGGTTGAGGGCTGccctccagtgaggcccacggGGGAGACAGAAGAGACAAAGTGCCTCAACCTAACCCTTCTccccatcca

gggccccagcccggtgtggagccaggcagccagcaatcccgtcgttgcgatcctcgcaggctggcttccaggctgggaacggacggaccgtgtacctgaa

ggaacacgctactcgtgctcagttcttgtaccgctggtggccagcttttgtctcactgctacgctctgtgtcagcgttatgtaagactcagaagggctgg

acggagtcctcttccctggaagccattgtgcaagcttg------------------------------------------------------GGAATTAG

CTGTCAAAGCCCTCGGCCCCCAGGGGATATTTttcaggggaagattttcagtgacggatttcgactgctttaatagtttgcgaattgctgtggtttgtcc

ttattccatcttgcaatctgttagttctggTTTTTTGGAAATCGATGCATTTCCTCTAAATTAAATgcgttggcacaagattctttctttctctccagtg

gctgcgggctctgtggggacgttgtcctttttatccccctcaacccgtccttcatttgtgcctatttttatggctttatttgtgccttttttgcgaggtc

agtctcagggagttttagcttcatggatcaatgttggacctcatcagtcctctcccgtatgtgttgttttcaagttttgctgtttctttcgccttcgctg

ggtgtgaaagacttgcttgccagagg--------------------------------------------------------------------------

----------------------------------------------------------------------------------------------------

--------------------------------------------------------------------------------GGAAGGCAGAGAGGAGAGCA

TTCCTGCAAGGCACAAAAgtaaacattgtcttgcagccaagcgtgggggcaggaggcaggggccagcgtagccaggtggaggacttgccaggcttggtgg

ccagtcccattgtcccctccctcctgccaggaccccagcagcactcccgggcccatgcttctcacaggagggaggcaggcacgggggcaaagcgccccag

ccttgctgtgttccccacttactccctgCCAGCAACAGCTCTGGAACCACAGGCCTCCCTCCTGGAAAggaagggcccggagcaaccagacgtctcgagc

agtggcacagggagcccttgtcatcagcac------------------------------------------CTGACTTGTTCTGTCTCATGTCTTGGAC

TTGCACACAAAActtggcttgcaaaggacttctgcaggggccggccctgtggtgcggtgggttaagccgccatctgcaatgccagcatctcggaagagag

tgctggttcgagtgccagttgacctgcgtcggatccggctccctcctaatgtgcctgggaatgcagcagaggatggcccatgtgcttgggcccctgcacc

cacatgggagacccggatggagttcctgggtcgtgaccctgtcattgtggccacttgggaaataaaccagtggatggaagatgtctctctctttctatct

ctccctttatctatgttactcaacctttcaaatacataaaaatacatcttaaaaacaaacaaaaaaaaccttctgtggtgaaatgaaagtcagagaaaga

tttggagatggactgcaagctgactggctttctcctctgggggccccaCTCATTTCCTGTGAGATCTGGGGCAAGTTATTTAATCTCTgcaccTCAGTTT

CCTCGTCTGTAAAATGGGCATGATAGTCATGGTgtgcacctgctcaggactaaatatgcagcacgcACAGAGCCCAGAGCACAAGGCTCTTCCCCTCCTG

GACTTAactttccctggtgttggaggcagcaagcatgggagcgctccctgggcggcgtccgaggtgagcgcctggcagcccgtctgtgtcctcctct---

-------TCCTCTTTGGCTCCTTGTGGTCTGtctgggcggctgcaacccaacagcctgtg----------CAGCCTCCCTCTTGCATTTCCTAGAAAAcc

ctcagcg--CCGGCTCCAGCTCAGCAGATTTGGGGCCCTCCTcctcatgacttgacctccacctaaccaggaaaaggtcttgtgagacaccccccgcccc

caccgtctgCTTCCTATAAAAGGCAGGCAGGATGGCCAGGGAGGAAGAGaagcagagagcaaccctgaggctggaacttggctctcccgtggcagcagcc

cCAGC---ATGAAGGTCTCCGCAGCTCTGCTGTGCCTGCTGCTCctagcggccgcctgcagCTCCCAGGCACTCGCCCAGACAG-GTGAGGCCCATCTCT

TCctccaCTCCCTCTCCTTCCTTAGGTTTCTCTcgccttctaagccaaagggccccactttatatatatatatgtatatatatatatatatatatataat

gtatttatttatttgaaagtaagagttgcagaaagagagaaagagagagaggcagacagagagaggccttctatccactggttccctccccagatgactg

caatgcccagtgttgggccacaccaaagccaggagccaggagcttcttctaggtctcccaggtgggtgcaggggcccaagcacttgggccatcttccgct

gctttcccaggccacggcagagagctggatcggaagtggagcagccgggactccaggtttacccactgcgccatagtgctggccctgggGCCCCACTTCT

AAACCCAGCAGAGCAGCAAGGCCCCTTAGagcttcactc----------------------AACGCTGAAACCCAGATCGGAGACAGGAGGGGCCCagcc

caccttgctgcctggagcccagtgtcttt---------------------------------------------------TCAGGTCATCCCGAACTCTG

CAGCTCTGGGGCCACTCCtggcgcctgtcccagggctgcccctggccacctccaggcagtgcagtctgagccacaggtcacagcaggggtgctgggctcc

aggacggatgaggtctgcttctgccttcgcctctggtcacttcctgctgcactcaagcccacccgc----------------------------------

---------------------------------------------------cttcagaggctttctctttgtgactttgcttctag-aaacgaagccagc

cctgaccgcttgctgcttcagctttgtCAGGAAGAGGATCCCCCTGCAGAGGCTGGTGAGCTATcggaagaccagcaaggccTGTGTCAAGGAGGCTGTG

AT-GTGAGTAGAAAA--CCCCGCCCA--TGCACCCCTCCGCAC-CCCATGCTCTCCAAAC-AAAGCTGGGCCC-AGGTGG--GCCCTGCGA--GACT-GA

AGCC--AGG-C-AG--CT--CCTGGCTGC-TAGCCTGAGGACTCTAACTCTC-CCGCTGTAACAG-TGCAGAG-GCAC-TGGGGTCCCTTCACAGGTGGC

TGCCC--ATG--CTCTGGTCT-C-CGCTTTAGTGGACACCAAGATGACTTCCCCCGC--CACCAAAAGCAGCCACGGCCTCCAGGG-CCCTTTCT-TCC-

CACAA-TCCT----CC---CC---------GCTCC--CAGCC-CAG-----GGACCA-------------CCCA------C----CCTG------CCAT-

--GGGGT-CCACCTGACTGTGCCGGGCC-CCTTCCTCCACAG-CTTCCGGACCAGACGGGACCAGGAacTCTGTGCTGATCCCATGCAAAAGTGGGTCCA

GGATaccatgagggtcctggat-GGAAACGTCACTCCCTGAGGACTGCAATTCTTCCACCCacatgcagatgtgacctgagtaggggacatgattgctgc

ctcttctctggcttcccatgtgcccccagcctggttttcctgtaacc---TTCAAATCGAATGCTTTCTCTTTTATGATGCAAAATGcattctgtcttca

gtaatgttagctaagactccctcccccccaccccaccaacccatacctgctgccaccgctctccctgtccctgtgcctcctccttcaggcagcctcccag

cctgagatgagtaacccatgtactcctggtccccagaggaaaaccaggggcccgatggaggcaatggcactgggcagaactgactgtggggggtggcgct

gtacaaccagaggatgagcaagttaggacgaaagggagagggccagagtctggggtccagaatgtaaagttgaaggcatttagataggctgggctgcagg

gggattcaaataca----gggcaggagctggacccagctctgaaggcagccagaggtgtgatgatttcaaggaagagagactagggagagcagttgtgcc

ttgagtgggatgggtggggccgggaacattgagaagggctccctgcacctgtccaggtaagaggaaaaggggcagttct---------------------

----------------------------------------------------------------------------------------------------

----------------------------------------------------------------------------------------------------

----------------------------------------------------------------------------------------------------

----------------------------------------------------------------------------------------------------

----------------------------------------------------------------------------------------------------

----------------------------------------------------------------------------------------------------

---------------------------------------------------GGGGTGCTGGGGAGGAAGCAGATGGCCAAGACTGGGCCAGattgtgaca

cactgaatgctggggacacagggtggagcccggggacacaggacatgccaccttgctccccacagccacagagctggcctgcacaggggcaccaggtctg

aggcccagtcttagctccctcccccgtcacttccctaggtcatacactgtggccattcagttcacagggacagggcctcttcttcccctgtggccccggg

gccctcctgacctgactcagctgtctgcccctcctgtatgagctgagttggatctctggaagcccagggtccactcatgcctacatccccacagaggaca

gggccccgaggccggtgtcccacggtaccaggtagcatgctgggcaactcgagacaccataaagagttcaggcctgag----------------------

----------------------GGCAGGAGCCCTGGGCTCCAAGGCCAGATCTGGCCCTGgcttgttccgtgttctatccAGCCCTCAGTCACCTCGTCT

GTGCAATGGGGTTAGCAGCAtctcacaggcagacgagctcacagccagggcattggatagcatcaccccctgcacctcctgcatccatcactgcttcact

gtgcctccctc----------------------------------------------------------------------------GGCCGTCAAACCT

GGAGGCAGAGAATCTGCcaatgcccccgcccccaacacacagcgtcacagcaaggcagggagtaagtcttttattggctcct-----------GTTCCCT

AGGTACCAAGTCCAGCATGTCTCCCTCCTCCcccacccttcagcccctagc----CCCACCCCTCAGTGCCAATCAGTTTTGCACCCTGAAGTCCccttg

ggatccaagaggcaccagccagcaggagtgcaggattctgggattaagTAAAGTGCAACATTGTAGCTTATCAAGTCCCAGTAGAaggaagggagcagga

ctagaggtaggtgtggcgaggaa--------------------------------------------CAGAGCTCAGGAGGGAAGGGAATCCGC---TCA

GGGTCCCTTTGGCAGGCAGGGCTTCATCTTTTTAAAGTAGCCCTGGAGCCAGCCCACTGTCTTCAAGGCACAGCTTTCTCTGCCTCCTCTCAGCTTGAAT

--CTGCGAGCAGAGAAaaaggagagaccgagtcacccactgcacaccacacacaaataccatgtctacctccttgctgatgggtgcccaagcctctccct

------------GGGCCTGGCTTCCCCTCTGTGAATGAAGGCGCTGTTTGAGAGCTGATCCATGGCTGGTACA--AGGGTGTGGGGGAGGCGT--GGGGT

TGGTAACTT--ACTTCGTGGACCC-TCTCCTAAAGGTTAGGGCTGCAG-ATGTGGGGGAGGGACT--ACAGGCTTTGCAGGCGCTCTGACAAT-------

-----GAA--CCTTGACATGGA-T--CTCTCTGCTCCTACTTCCAGTGTTTCCAAATCCATCTGG--CCCCAC--A-------GGA--CATGTGTGTGTG

TGTGTGTGTGTG---------TGTGTG---------------------------------------------------ACTATTGTGCAGTCTATAG-CC

AG----G-CTCT-GC-CTCTGAGTGAACTTAACACCAATACATTTGCCTCCCTGAGCCTCGATTTTCATGGTAATAGCAATGTGTGtcccaGTAGGGTCC

ATCGAGGTGATGTATGGACACCTGAAAATACCTTAAAATAATC-CTAGTATGTGAGAACAGCCTTTTTAAAATTCTTAACCACCCTTGATCCTGTGATGC

AGCTGTCAGTTGCACCCCATGTCACAGGTAAGGACAACAAAAGTGTGAATGG-TAAGGAGGGACA--GGCAGTTATCTGTCCAAGGTCACCCAGGACTCT

AGGTTagCAAATACAGAGGCAGGACTTGATCATGGACCCCTTGCTCTt-CCACTACACAATAACACCCTGTGGTTGTGGCTCGGCAGCTCCTTCCTCTGG

GCACACAATGCTGGGACCTGGTATCTGGCATCTTGGAGTTCCAGGAAACACAAGGTACCACAGGAg--------------------CACCTGCTTGGGAG

CTCGGCACTCCCCCTAGCGCCCAAGcgcggctctgcacgcagttgtagaccgcctggctggctaGGTTTAGAAACCTTATTCCGGTGGAACAGCGCCATC

TAGcgcttgctgttctaattcccgggccgccgcgttgggtcctgcaggggagtgggcaggg---GATCACTCAC-ATGATGGCGTCGTAGGGGCAGGTGG

AGCTGCTCCTTCTGTAGCAGTGGATGGTCTTCAGGGGAATCCTTTTCTCCACGAAGGAGAAGCAGCAGTTGGAGGAGGATACGTGCA-CTGGAAGAGAAG

CACAGAGGAgagtctgcatcgtttcccagcctctcacccgcagggtgatttttcctaaagcccctggcgagcagagccaggcccaggctttcaggccacc

cacatgcagagggcacggattcttcgttccggagcagtgaggacagcacaaggactggacctcacatcggagctccccacaagcctcctggaccctacct

tcagaggctcctccagccctgactgcaccacaaaggtgtggtcctaccctggagctcaggcccaa-----------------------------------

------------------------------------------------------------GGCTCTGGACCCCACACCTTGGGGgaggggcagcggtggg

ggcgaagctggtctgtcggacgcacacaggacactgcggtccttggtcacagacctggactgagctcaaaactgttgaggagtgcaggctggctaaccag

CAAGGGGAGTGCTCCCAGGAGCTGCCCATCCCCCCAACACcatctctcctggctctcagttctgcccagcacccaccctccacccctaagagacgtgtgt

tccagtcctcctggcttgatgtttcacacactcccttccatgtgggtcatgaggctggggtcctcccaccagccttggagacagtgccatcaacttcatc

tcaccacccacagagaaggcagaatctgaacagtaaacttgcccagggttagccaaccacagagcccccagtgtagcctcagcctctgcctgtcacccgg

cacagtgtacagccccgccccagtgcctcctctgagcccccccactacccagcttgctctaaatgaacggacaagtcctccctggtgtcaaaggctctga

gctaaatttcttcc--------------------------------------------------------------TGCTGGAGCTGGCGGCTCAGCTCC

GTGTGTTCGCCCCtgactcgccgttctccttcccgccccagcccatccaagtcctccttctaactc-------TTAGGTGAGAGGAGGGGAGATGGAGac

ggatga-tcaaggtgagccctgccccagcccaggaggaactccctgccttccagcccgcccacccagggaagggcagagggatggcagcccCACTCAC--

TGCTCTTGCCATCCACGTCTTGAGGCCACATCgcgaccagcagcaggcacgccagagccacggggacaagcttcat--ctcgggcagtcgggctccggag

acactgagatctctctcctggcccatccttcagtgcccaaagcttatatgagcccggggtggagtttcccaggccgtgcagaggt---------------

--------------------------GGGAGTCACAGGCAGAAGGGACACTTACTCACACCCAGTGgccgcaTGGAAAACTCCCTCCCCCTCCTCTCCTG

GCggtgctggaggtgtgtttgggggccgggcggggttgggaacttccccaagcgaggagtagataaggatctgataaagaagataCGTGGGAGCTGTCCT

GGCTGAGTGGGAAGCGGAGAAAGGTgacTGAGAGGAAGTGGCCTCTGTGTGGCTTGCCTGGCCCCAAATCGCTGCATCCAGATCTGCAGCTGCTGCCGCT

GGGACAGCACAGAGCAGCGGTGTTCTCTAGCCTGGAGCACCTGTGCCGGCACCTGCA--CCAGAATC--CAAACCAGCGATTGTGCAGACTTGCCCAAGC

GCGTGTGTGCACTAAGTGGAAGCTGTAACACGTGACT-GAGGGTGGGCAGCACGGAT-------C---CCCTAAGTCAGGTGCCTAGGCCAGAGGCTTGC

TG---------TTGCTATTTTTTTAATCCTCA

>hosa_*CCL2*-*rcCCL1*

ggcaccccatcctccccatttgctcaTTTGGTCTCAGCAGTGAATGGAAAAAGTGTCTCGTCctgaccccctGCTTCCCTTTCCTACTTCCTGGAAAtcc

acaggatgctgcatttgctcagcagatttaacagcccacttatcactcatggaagatccctcctcctgcttgactccgccctctctccctctgcccgctt

tcaataagaggcagagac---------AGCAGCCAGAGGAACCGAGAGGCTGAGACTAACCCAGAAacatccaattctcaaactgaagctcgcactctcg

CCTCCAGC-ATGAAAGTCTCTGCCGCCCTTCTGTGCCTGCTgctcatagcagccaCCTTCATTCCCCAAGGGCTCGCTCAGCCAG-GTAAGGCCCCCTCT

TCTTCTCCTTGAACCACA--TTGTCTTCTCTCTGAGTTATCATGGACCA----TC--CA-AGCAGACGTGGTACCCACAGTCTTGCTTTAAC-GCTACTT

TTCCAAGATAAGGTGACTCAGAAAAGGACAAGGGGTGAGCCCAACCACACAGCTGCTG-CTCGGCAGAGCCTGAACTAGAATTCCAGCTGTGAACCCCAa

atccagctccttccaggattccagctctgggaacacactcagcgcagttactcccccagctgcttccagcagagtttggggatcagggtaatcaaagAGA

GGGTGGGTGTGTAGGCTGTTTCCAGACACGCTGGAGacccagaatc-----------TGGTCTGTGCTTCATTCACCTTAGCTTCCAGAGACGgtgaCTC

TGCAGAGGTAATGAGTATCAGGGAAACTCATGACCAggcatagcctattcagagtctaaaaggaggctcaTAGTGGGGCTCCCCAGCTGATCTTCCCTGG

TGCTGATCATctggattattggtccgtcttaatgacacttgtaggcattatctagctttaacagctcctCCTTCTCTCTGTCCATTATCAATGTTATATA

CCCATTTTAcagcataggAAACTGAGTCATTGGGTCAAAGATCACATTCTAGCTCTGAggtataggcagaagcactgggatttaatgagctctttgtctt

CTCCTGCCTGCCTTTTGCTTTTTCCTCATGACTCTTTTCTgctcttaagatcagaataatccagttcatcctaaaatgcttttTCTTTGTGGTTTATTTT

CCAG-ATGCAATCAATGCCCCAGTCACCTGCTGTTATAACTTCACCAATAGGAAGATCTCAGTGcagaggctcgcGAGCTATAGAAGAATCACCAGCAGC

AAGTGTCCCAAAGAAGCTGTGAT-GTGAGTTCAGCACACCAACCTTCCCTGGCCtgaagttcTTCCTTGTGGAGCAAGGGACAAGCCTCATAAACCTAGA

GtcagagagtgcactatttaacttaatgtacaaaggttcccaatgggaaaactgaggcaccaAGGGAAAAAGTGAACCCCAACATCACTCTCCAcctggg

tgcctattcagaacaccccaattTCTTTAGCTTGAAGTCAGGATGGCTCCACCTGGacacctatagg-AGCAGTTTGCCCTGGGTTCCCTCCTTCCACCT

GCGTTCCTccTCTAGCTCCCATGGCAGCCCTTTGGTGCAGAATGGGCTgcacttctagaccaaaactgcaaaggaacttcatctaactctgt-CCTCCCT

CCCCACAG--CTTCAAGACCATTGTGGCCAAGGaGATCTGTGCTGACCCCAAGCAGAAGTGGGTTCAGGATTCCATGGACCACCTGGACAAGCAAACCCA

AACTCCGAAGACTTga---aca-CTCACTCCACAACCCAAGAATCTGCAGCTAACTTATTTTCccctagctttccccagacaccctgttttatttta---

ttataatgaattttgtttgttg----ATGTGAAACATTATGCCTTAAGTAATGTTAAtTCTTATTTAAGTTATTGATGTTTTaagtttatctttcatggt

actagtgttttttagatacagagactt--------ggggaaat-tgcttttcctct-TGAACCACAGTTCTACCCCTGGGATGTTTTGAGGGTctttgca

agaatcattaatacaaaga-attttttttaacattccaatgcattgctaaaa-TATTATTGTGGAAATGAATATTttgtaactattacaccaaataaATA

TATTTTTGTACAAAACCTGACTTccagtg-TTTTCTTGAAGGAAATTACAAAGCTGAGAGTATGAGCTTGgtggtgacaaaggaacATGATTTCAGAGGG

TGGGGCTTACATTTTGAAGGAATG----GGAAAGTGGATTGGCCCCGGTCTTCtccactgggtggtctcctctgagtctccgtagaagaatctttatggc

aggccagttaggcattaaagcaccacccttccagtcttcaacataagcagcccagagtccaatgaccctggtcacccatttagcaagagcccaACCCCCA

TTCCTTTTCTCACAGACCCTGACCCCTGCatgcaattcttcccttaacatattgCAACTGCCCCCTAACTGGGCTACCCACCCCCcaatctgtacct--C

TCCAATTAATACCCCAACCTGGAGTAATACAGACACTGccagtattaggaaataaggaaagag-------------TTAATCACCATAGATAAGATGATT

AGATTGAAGTTTCATAgagatgatgagacctgAACTTATTATTTATGAATGAAGAAGGCTTTTCTAGGAAAattataggatcattaagaaaggagaagga

agagtgggagcAAATACCTGGAGGTAGAAATGGTGATGATGTGTACAtcaagcagggagaaaaccaatgaaccagatgcgaattcgggcccacaccaatg

tcaagggatgacaa--------------------------------------------------------------------------------------

---TTAGAAAGGAAGGTTGAGTCAAGGGATTTGAAtgttagggtgAAAAGTTACTACTCAACTCTGTAGGTtaaaaggaaacgttgagAATCTTCAGTCC

AATGAGGAGGGATGTGCCATGTttagagattcagagataagtttcaggaaatgtaacttatagattttatacatacacagagaaatacggactagtgaga

agctattgccatggtccaaGCAAGAGATGATGAAGGCCTAAATATGGAGCCAAAGAGgcagcaat---GAAGAATGAGCCATGCAGGGTGAAATGCTGCA

TGTtgtaaatggaggagaaagacctgtgaCTTCAGATATGAAAACCTCATCTTCAACCCACATTTTAaGGGGGCAGCTTCCCTGAAACCAGAATGTGTTT

CCCTCCATtactatacccccatcccaatctcaggcacctggaatcatccatttaaacagatgagccttctattcctaaatagccACCTGAAGTGTGTATT

CCTTTGCATGATATTTGTCCCACc--TAAAGCATTCGACCTGCCTGGGCACCCACACCACGCcaacactcaggaaagcagatgtcttgctctgttgaata

aactgcatggttcttaacttcccagtctggtggggaaatgaccactgtgtcaacctagagcaggcagtgcttttggcagcatgaggtgctggggacaact

ttgactggcaagaagcacactcaggttctcaccccgcatccagcgctgactcgctttgtcagtcaagacaggtcagatattctgagcctacatcgatcat

acaggtaTGATAATGTGTTACAAATAGGAACCCAGAggaaaggttccctttcggatctgggagcaca---TCTGTTGGAAAACTTCCATTTCTACTAACT

GGAGTTGCAGagggagagaagggattctGCTTCTACATTCCTGAGCCAGTCCAGGGTCCCTGAATCAGACTACCGAATCCCTTCAAAGCTCCAAGTACCC

TGATATATCAGTCAGCAGACAATTTATTGACAGCTATTtagaaaACTCACTGACCCTCACTCCAGGTCAAGCAGCgtcccctgcctctcctctaccccta

cattccctggccttgatCACCAGTCAGGAGTGAAATCTCAAATTGCAGTAGATGCCAagagGCAAAAAGAGAATAGAATGCAAACAAATGAGACCTCATC

ATACGGCTTCCGAGCAGCAACCTTTTGACGCCAGGCAGATTTGAGGCAGACAGTCTGGGAGGAGAgGAGGCAGAGAAAGGGGGGATCCACATGCTCAAAC

CCCAAATTAATCTGCTTACATTCCCCTTGCAGGCCACATCTCTTCaTTTTCAGGAAGTCTTGACTCCATACTGTTTTCCACCCAAGcatggaattcctt-

----TC--ATGATGAAACTGAACACAGGGCATTGGCAGTGGTGAGACTCTGTTTTAGAAGAAAGTGCCAAGTGCAATGCATTCATTTCCTGTTGCTGCCA

ACAATCAGTTCCAGgaaatctaggcTTTTTATGTCATGCTCAAAATTCTTCCAGCCTATGCTCATtattcaaatccaaagccacatccacatctgtaggt

gttagttacagaagcaccatatttccaggtaccaaaatc-TGTATTAGTTTCTTATTGTTACTGTAACAAATtcccataagcttaatggcttaaaacaac

aaaaatacattgtcttatagttctggaggtcagagttatcatgggtccatagggctatattcattccttccagaggttctatgggagaatccattttctt

gcttttttccaactgctagaggctacctcaATTCCTTAACTCATGGTCTCTTCCTGTGTCTTCAAAaac-CTCTCTCTCTCTCTCTCTCACTCcttttct

ttcatagtcac----------------------------ATCTTGTCTCTGACTCTGACTTTCCTGCCTTCATCTTAAaaggtgattatattgtgattat

gctgagcccatttggataatccagGATAACATCTTCATCTCAAGATTCTTAAGTTAATCACATttataaaatttcttttgctatgtcaggtagcatactt

atagagcatagggattcgattaggatgtaggctttttttttttttttttttttttttggaaatggagtctcgctctattgcccaggctggagtgcagtag

cacaatctcagctcactgcaacctctgtctcctgggttcaagtgattctcctgcctcagcctcccaagtaactgtgattacagatgtgcaccaccacacc

tggctaatttttgtatttttagtagagatggggttttaccatgttggccaggctggtctcgaactcctgacctcaggtgatccacccgcctcagactccc

agagtacaggtgtgagacaccacgcctggctacaaggATGTGAGCATCTTTGGGAGGGgagcactactgggacaatcacagccaggagccaggtgaagat

ttacaataggtagttggacactaagacatgGCCTTTGAGATCTGTATCTGGTTTGGTGACTAGATTgtcaggtaaacttttgcaagtcaccttttatcta

tgggttcataggctgtggatttaatttgtgcattaaatgcttcctaagtgacctaaggtatattggAGTCACCTGAGGTCTTTCCAAAACATCACAACAT

CCtcaaagattctgatttagaatgttacaatgcagggaaggcTCCTCTTCCCATTGCTGTATTTTACGAGTTCCCTAGAAGAtggataCTGGTGCACAGA

TGAGTTTGATGCCATGTGCTCctcagTTCTCTCATATTCAGGTCATTGGAGCCAACAGCCAGTCCTggtAACCTCTGACTTGGGTTTTCATAGTGTCCTT

TGttcTGACCTAGCCTTGACCAATTTACAATCAGGAACAAAAGGGAGCATAGAGTCGAGACATTTAGGACATTGTAAAGTTTGAAATAC-AACTGTTC-A

CCATCCCAGGCAGTGGAATGGAATTTTTAAAAATGATTTGAACAACAAAATATCCATAAAACctttcagtttgacagAGTGGCTTCAGGAAGAGAGTGGG

TTAACAATGTGGCATTC---CCATTCTAGCCCGGCAGCCTGATCCTAAAAGACAGTTATGGGTCGGGTGTGGAGGAAGCACAGAAATatgtcaagcactg

cgagtagaaagagattttacatgtctttactggcagattggaaaagtgctagtaattttttgagaaaatTATGTTGCCAGGAAAATCAGAAGCCAAGACT

GAAAAAGCCTTTGACTATTCTATCTATAAAACAACTTCCGAATCTTCCATGAGCTAGaAACAAGCCATGACAGCCACTGAATTAGGAGGGCATGTCCACT

GAGTTCCATGTACAGTATGGCCAAGGAGAAGAAATGGACAAGCAAGGGCCTCACAGAagGTGGATCT-GGGGCCCCGGACAAGGAAGCCCTTCCTGCA--

-AGTG-AGCTCAGAGCCCCTGTTCCTGTTCCAGGAAGAGTCCCTTTTGATGTCTGCCCAGCAGTATATCAGAATTGCTACAAGTCAGTCATTGCTGTGTG

TCCCCCAGTCT--TCCTTGTCCAATGAGAATGTTTACTTTAAGCATCTTGTTCCTATTCCACCTTGTAAATGAGTGTTTAAATTGAGCAGATAAATTGTT

CATTTTTTAAAAGTACTGTCAATCTTTCATTTGGAGGCTATAAAATTCATTTCCATAAGGTATCATTATCTctTTTTTAAAGTTTTCAATTTTGAAAAAT

TTTGGTTAAAAAATGCATACCATGAAATGTATCATATTTACCATTTTTAAG-TGTATACTTCAGTATTGTTAATGTTCATATACTTAACATTGTTGTGCA

ATAGATTTCCATCTTGTAAATCCATCTTGTAAAACTGAAACTGCATACTCATTAAACAATACCTGCTCATTTCCTCCGCTGCCCAGCCCCGGCAACCACC

ATTCTACTTTCCTCTTCTATGAATTTGACTACTTTAGGTACCTTCTGTAAGTGGGATTATACAATATGTGTCTTTTGGTGAATGGTTATTTTATTTAGCA

TAATGTTCTCAAGGTTCATTTATGTTAAGGCATGTGTCAAAATTCCCTTCTTTTTTAAGGTTGAATAATATTCCATTGTATGTATATACCACATTTTGTT

TATCTGTTCATTCAGTGATGGACAGCTGGGTTGTTTTCATCTCGTGGTTATTGTGCAGGATGCTGTTCTGAATGTGGATATGCAAATGTCTCTTCCAGAT

TCTGCTTTCAGTTCCTTTGGATATATACCCACAAGTGGGATTGCTGCATCATTTGGTAATCATATTTTTATTTTTTTAAGATCTACCATATTGTTTCCAT

AGTAGCTGCACCATTTTACATTCCCACCAACAGTAAACAAAGATTCCAGTTTTATCGCATCCTTGTCTTCGCTTGTTATTTTCTGTCTTTTTGATACTAG

CCATCCTAATGGAGGTGAGGTGATACCTCCCTATGGTCTTGATTTGCAACTTTCTAGTATTAGTGATATTGAGCATCTTTTCAGATGCATGTTGGTCGTT

TGTATATCATCTTTGGAAAATGTATATTGAAATCCTTTGCCTATTTTTAATTGGGTTATTTGTTTTGTATTGTCGATGCTGTAGAAGTTCTTTTTTTTCT

TTTGAGACAGAGTTTCACTTTGTTGCCCAGGATGGAGTGCAGTGGTGTGATCTCAGTTCACTGCAACCTCCACCTGCTGGGTTCAAGCGATTCTCGTACC

TCAGCCTTCTGTGTAGCTGGGATTACAGGCACACACCATCACACCCAGCTAATCTTTTGTATTTTTAGTAGAGACTTGGTTTCACTACTTTGGCCAGGCA

GGTCTCTAACTCCTAGCCTCAAGTGATCTGCCTGCCTCAGCCTCCCAAAGTGTTGAGATTACAGGCGTAAGCCACTGTGCCCAACCTGAAATTCTTTATA

TATTCTAATGTTAACCCCTTATCAGTTACATGATTTGTAAGTATTTTCTCTCATTTCATAGACTGACTTTTCATTCTGCTGATTGTGCCCTTCAATACAC

CAAAGTGTTTAAGTTTGATGTAATTCCATTTATCTATTTTTGCTTTGGTTCCCTGTGCTTTTGGTGTTATATCAACAAAAAAAAAAAAAAAAAAAAAAAA

AAACAGCCAAGCGCAGTGGCTTACGCTTATAATCCCAGCACTATGGGAGTCTGAGGCGGGCAGATCACTTGAGGTCAGGAGTTCAAGACCAGCCTGGCCA

ACATGGTGAAGCCCCATTTCTACTAAAAATACAAAATTAGCCAGACATGGTGGTGAATACCTGTAAT-CC-CAGCTACTTGGGAGGCTGAGGGAGGAGAG

TCATTTGAACCTGGGAAACAGAGGTTGCAGTGAGCTGAGATCGTGTCATTGCACTCCAGCCTGGGCAAAAAGAGTGAAATTCCATTTCAAAAAAAAAAAA

TTGTCAAATCCAATATCATAAAGATTTCCCCCTAC-TTTCTTCTGGGGGTTTATAATTTTACATCTTGCATTTAGGTCTTTGATCCATTTCGAGTTGAAT

TTTGTATATGGTATAATATAAGGGTCCAGCTTTTTTTTTTTTTGAGACGGAGTTTCACTCTTGTTGCCCAGGCTGGAGTGCAATGGCGTGATCTTGGCTC

ACTGCAACCTCCGTCTCCCGGGTTCGAGCGATTCTCCTGCCTCAGCCTCCCGAGTAGCTGGGATTACAGGCACGCGCCACCACCCGGGCTAATTCTGTAT

TTTTAGTAGAGACGGGATTTCTCCATGTTGGTCATGCTGGTTTTGAACTCCCGACCTCAGGTGATCCACCCGCCTCGGCCTCCCAAAGTGCTGGGATTAC

AGGCGTGAACCAGTGCTCCCAGCAAGGGTCCAGCTTTAATCTTTTCATTTGGATATCTAGTTTTCCCAACATcaactgtttaagagactgacatttcctt

actgagtggtcttgacatcCTTGTCAAAGATCATTTGACCATAtacaagagattttatttctgggctctctattctattctactggtctatatgtctgtc

tttatgccaataacacattattttgattattgtagaa-----------TTGTAATATGTTTTGAAATTGATAAGAGTGAGACCTTCaagtttgtttttct

ttttcaaaattgtTTTTGACTATTTGGAATTATTTGATATTCCATGTGAATTTtaagatgggtttttctattttggcaaagataccattgggattctgat

----------------------------------------------------------------------------------------------------

----------------------------------------------------------------------------------------------------

----------------------------------------------------------------------------------------------------

----------------------------------------------------------AGGATTGCACTGAATCTGTAGATTACTTgagtagcattgtta

agctgtca--------------------------------------------------------------------------------------------

----------------------------------------------------------------------------------------------------

----------------------------------------------------------------------------------------------------

--------------------------------------------------------------------------------ATCTCAACAATATTAATTCT

TCCAATTCATACACCTgggatgtcttctcatttatttgtatcttctttaattttgggcaatattttgtagtttttgttgttcaagtttttacttctagat

attggcttatccctaatattttattcttttcaatgctattgtaaatggaattgttttattaattttcttttcaaagtgtttattgttcatgtatataaaa

tgtctgatttttgtgtgttgatttgttgtgttgtatttgTACTCTGCCGAATTCCTTTATTAGGTGTAACAGTTCTTTTgcatgtgtgaAATTTTCAGGG

TTTTCTACATATAAAGTCATATCAtctggaaacagataattttacttcttcctttccaatttagatgactttttttcttgttttgttcagatgggtctca

ttccagtactatatttaactgaaatgacagaagcaagaaagtgctttcagtctttttctattgtgtatgtttttaa--------------------CTGT

GGGCTTTTCATACATGGACTTTATTATGTTGAGGTAgtttctatttccacactattttcctagtg-----TGTTGAGTATTCTTATCATGAAAAGATGTC

GAATtTTGTCAAATGCTTTTTTGTCATGAATTGAGATTATAATGTaatttttgttcttcattctgttaatgtggcatattacattgattggtttttgaaT

GTTGAATTATCCTTCCATTCCAGAAATAAATCctacttagtgatgGTATATAATCCTTTTAATGTGCTTTTGAATTCGGTTTGCTagtgTTTTGTTGAGG

ATTTTTGCATTAATATTCATCAGGTATATttgctggtagtTTTCCTTTCTTGCAGTGCCTTTGTTCAACTTTGGTATCAGggtaatcctgGTCTTACATA

GTAAGTTTGGAAGTGTTTCCTTCTCTTCAAtatCTTAGAAGAGCTTGAGGAGTATTGGTATTAAATCTTCCTTaaatatgtggTAGAATTCTCTAGAGAA

GCCATCTGATTTTGAACTTTTCTtgcttgggaggtttttgattactgataaaagcttttt----------------------------------------

----------------------------------------------------------------------------------------------------

----------------------------------------------------------------------------------------------------

----------------------------------------------------------------------------------------------------

--------ACTAGTTATAGGTCAGTTGATATTTTCTATTTCTTAATaactgtgtttctaggaatttatccatttattatggttattcaatcagttgg---

--CATACAATTATTCATAATATTCTCTAATAATCCTTTttatttctggggCATCAATTGTAATGTTCCCTCTTTCATTTCTGATTTTAGTtgagtcttct

ctcttttcttcttacttaatctagctaagggtttgtcatttttgttgatcttttcaaaacaacaattttttgtgttgttgattttttcttttgtttcatt

tatcactgatccattctttattattttcttctttctggtagctttgggt--------TTAGTTTGTTCTTTTTCTAGGTTCctcaagatataaagTTAGA

TTGTTCATTTCAGATCTTTCTTCTTTTTTAATgtaagcacttacaaCTGTAAACTTCCCTCTTAGCATTGCTTTCTCTGCACCACAcaagttttgttata

ttgtgtttttaactttttattctgaa-CAAACTTTAGGCTTACAGAAAAGTTGCAAAAATAGTACAGagggtttccttatattccttgctcagcttcccc

tgatactaacctcgtactttcttcatgtctggtaAACTTTGCTGTGTCTCTGCTCCATATATCTTCTGCAGTATggcctctgagtcaAAGGGGCACCCTC

ATCTGCGTCAAGTCATCCTcatagttaagggagggtggaggaattcatgagaatattaacttctgctcagagatgacatatgtgacttctgcttgcaccc

aacagggcaaagctaaattactggttgcACACAGAGAATGTCCTCACTGGGCTGAACGACCAAAATcccactaaggCCCAGAATAATTCTCATCTCAAGT

TCTTGCAGGACTTCTAggacttcacctgtcccttctttctaagacactcatctccttttcttatatccaatgcagtatttcctgaaagtcaatccccctt

tcaatATCTCTGTTTTGAAAAAACAAGACAGATTTAGgaacaACTTGCTCAGGAAGCTATCAGTATCAGTTTGAGTTCCTCCttagtatctcttcatcca

ctacccatccttatttcctaatactaataataatgagagctagttcttactgagcactaactgcattctgattattttcatttcacttttaatttttaac

tcattcttcacaacaaccaagaaaagaagttacttttattatctctattgttacagatgtggaaactgaataacagatagaataagtagcttgaccatgg

ttatgcaggaaaaccaggcaatgatgcaggcaaccatttcttaatcacgtcgttcaactgcctctcctggcactaagcagcaaatacttttctctgcctc

ctatttcccaagacattttggtccttgttcagataacatagaacaatgtggctcaaaggaaacctcagcctatcaaattcacacctggtaaatgtagggt

atggatgggaccattacctgaatctgtgctcatggcaaacattgcatgccagtcacagcaccatgaactcaaactcaaagcaaggtctcacagtgctctc

caggacatctctctgggctgccctcactagtcaaaagagttgatacctgaatggcaccctataaagtctattgtctatggtgcacataacaaaacagaat

ggtggttacctcttggcccctggtgaccaccgttctattttctgcttctatgagtctgttttacgtacctcatataagtggaatcattcagtatttgtcc

ctttgaaactaattccacttagcataatgtcctcaaggttcatccatgttgtcacaagtggaaggattccttcttttttaaggctgaataatatcccatt

gtgtgtatataccatatattctttatccattcatctattgatggacacttatgttgtttttataacttgactattttgaataatgtaattacaaacatgg

gaatgtagtggcatgccatattaaaaatactgaaagaaaaaaatcaatcaacaattctagataaggcaaagctacccttttcaaaagtgaataagaaatg

aagacattccagccttccaaaaaatactaaagagagttcttcaggctgaagcaaaagatcactaaatgataattcaaataaataaagagcattggtaaaa

gtaactacataagtaaatataaaagacagtataaatacttttgtttatttgtttaaaattatttgtttcttgtatctgatttaaaaggcacatgcataaa

gcactaactataaatctctgttcagggggacacaatgtataaaggtgtaatttgttatgataacaatgttaagatgaggagtggcatggagctacatagg

atcaagttttatacactattgaaattaaaatattaatctgaaataggttgttataaattatgatgttaatgataatccccagggtagccattaagaaaat

aactcaaacatatataataaaaatgggaaatacaatggtacagtagaaaatatttatggttcataaagtaaagtagtaattaaaaaagtgaggaacaaaa

gagacatcaatatataaaaaagggaaaatatcagatggtaatcctatttcatcagtaattacactaaatgtaaaagtgttaaaacccccaagaagcaaag

gttgagtaaatgaatttttaaaataaaccaactgtatgctatctataagatacatgttgtaaatctgaaatctcagataacttgaaagtaaaagtatgga

aaagtcaccccatggaaatgataatcaaaaaagagctagtggctatactgttaataatatcagacaaataaattttaagacaaaaattgttactggagac

aaagaaggacattcttttgtgactaagtgttaaaaatatatattttttaatctggaaggaaattaaccacttacatcttctgatgttgataatgtacaac

aatttttttaaataagtgtttctaattttgccatattttctacaaccagcttatattgtttttaaattatttaaaagtttttacaacattctgcaacaat

tcagcagccttttatttttaccattcccaggtcttgtaggtgacaggtacaggtctttccatcataaatataagcaggcattttctctgggatcttaggc

agggacatcttggggtacttcacttaaggattttctgaaggaaaaaggtttaaccaactggaacccatgagtacttccttgagctcctgacctgtctggg

caggacccccgtgttctactccaacttcatcgatcattcagcaggttatccatgatagttgtcttccaaaataaaagaaaaaagaaaaagaaagaaaagt

caaaggaaaggattctgctttttagaagcatcaggtggatggaacgtacaccctgggttagaaaatatgctgttctaatgtgttacattagagtgagctg

ggaagactccataaataaggagatagaaagtaggccaactcttcaccaatttctggagctgggagtaaaaacaatttgaaaaagagataatcgagagaat

gagtagcctgagcaaagtcataggggtaagataaaacgacataaagctaaaaccagaatcaaagccttgcattgctcccaaattaatattcaaagttgta

taatgctcaattcccccattgaataattttccttttttgcattccatgtccatgatccatggcccagttataggcatgaggaccccacccatggtctctc

atcaaaccccttttcactgatatggaaggcgattagttggtgattttgtaatgtgtatgtgagatgaggagctagttgtatgtgagatgaggagctagat

tagtggtttccaaaatttattctttgtatttctgagatatgtgtgtgggaagaatgacaagaagtctcttgtctctgattcaaactgaaggtctctgatt

ttgtcgataaatttgccttccctatattcttttacttggaaaaatattttcacagctcattagatgttttagaaacacagtattttaagatcttgatatg

ctgtgcagttccgatagcctataaaacaaatagtggttttaaaacatatcctcaaattattttacactcttcccattaagaggtatgactatgttcccta

accttgcacctgggatccttaagccatcatgcaAAATATCTAGCCATGTGGAGGGACCACTGGAAAGATcacacacacacacacacacacacacacacac

acacacatacAGAGAGAGAGAGAGAcacctgagaaattc---------------CATCTGGTCCAACTGCTGGCTGTTGAAGTCCTCccAGCCCAGACCC

CAGACACTGAATGAAGGCCTTGTGATatagtcaagagcaaaaaaacccagaagattgttattgtttt---------------------------------

---------------------------------------------AAGTCACTGTTTTCAGATAGATTGCTATGCAGCAATAGATtattgaAATAGACAC

TACAATTTTAGGTACTATTATTCTAagaatattgaattttatttTTCTGCTAATGTTCTATTATTTTACTTTTCTCTGGGTTTTAGAAAGCCACCAGGAT

TTAA-GACAGTGAAGAATCT-TTGA-GTCCTTTGTAGAGTTGAACCAAAGTTTGAATGTCTCTTTGTGGA--ctcgtgtcctagggataccactccaaag

ggaaaaggggaatATCCCTTACATATCTTTGACTTTGGTATCCCTGATTCCTTcct-TTTTCTATAGAATGTGTCTCATTTCAGAGAAACTGGTCTCttg

ataatagccatagattacatactgtggtcttcctctacatagaCCCTACCTCACCTACCACTCCTGGTCTTAGCTGAAAAACAggctagcctcgactcat

actgtcatttcctatcctcccactgaagt-------GCACTGGCTCAGCAGATTTAttactccata----gatttattactccattctatgatt------

----------catcctctctgcttccTATAAAAGGCAGAGACAGAGCTTCCAGAGGAGCAGAGGGGCTGAGaccaaaccagaaacctccaattctcatgt

ggaagcccatgccctcaccctccaac---atgaaagcCTCTGCAGCACTTCTGTGTCTGCTGCTCACAGCAGCTGCtTTCAGCCCCCAGGGGCTTGCTCA

GCCAG-GTAAGGTCCCtctctccttctccttgaagcacattgccccctctctgggttatcctggaccaatcaagaagacctgata------CCCACAGTC

TCACTTTAACAGCTACTTTTCCAAGATAAGGtaacttagaaaaaggataaggggtgagcccaaccacacagctgctgttGGGTAGAGCCTGAACTAGAAT

TCCAGCTGTGAACCCCAaatccagctcctTCTAGGATTCCAGCTCTGGGAACACCCTCAGtgcagttaccactccagctgcttccagcagaatttgggat

cagggtgatcaaagacaggaggcttctggggatgggtgtgcgggctgtttccagataccgggagacccagaatctggtctgtggaagcccagcttccaga

aacagcagctctgcagaggtggtacgtatcagggaaactcatgaccaagcattgaatgctcagagcctaaaaggggatccatagttggggtacccttgct

ctaaggaattggattattatattagcccctcctagcaatgcccagagtagccatcaattcctcttccg--------------------------------

-------------------------------------------TCTTTCAACTGGTGATGGTGCATCCCTATTTCACAGTCCAtaaaagtga--------

----------------------------------------------------------------------------------------------------

--------------------------------------------------------AAGGGAGTTTA-TGAA--ATGCCTC-------------------

----------------------------------------------------------------------------------------------------

----------------------------------------------------------------------------------------------------

---AAAGGGCAGAGACATTGGGTTTGGGATGGGCAGCTTTTCCctccacctcttcctttctttctgattccttcttcttaccATTCCCTGTTTTACAAAC

AGAAAGACCCAGGACACACCCTcaatggacttttcttcttgTTGTTTCATTGCAG-TTGGGATTAAtacttcaactACCTGCTGCTACAGATTtatcaat

aagaaaatccctaagcagaggctggagagctacagaaggaccaccagtAGCCACTGTCCCCGGGAAGCTGTAAT-GTATGTGGACGATGaccacccaccc

ctcacacctcagtcctaggttcttccctgggcagggaataggactagtatcagaatgagttggagtcaaatactgtgatgcatacagcatctctaacctt

atcccagacatttgccagtgagaaacaatacaagtaaagaaagtggcttctcactctcagctcc--ctttccagctatcattttacatctcagttcgttc

cttcatcctggaaccaagagagattcacttgggctaccaaaaagagctgcttc-tctgagtcCCCTTCCTTTGTTTTATCTTCTTCCTTCATCCCTGAGG

CAtccccatcagctaggctgatgGGCTAGACAGATTTCCCATAGACTTGGTCACACTCCCAGGctgaaccctcaaggtgttccatctgactgtctccttt

ctgctCCACAG-CTTCAAGACCAAACTGGACAAGGAGATCTGTGCTgaccccacacagaagtgggtccaggactttatgaagcACCTGGACAAGAAAACC

CAAACTCCAAAGCTTTGA---ACAtTCATGACTGAACTGAAAACAAGCCATGACTTGAGAAACAAataatttgtataccctgtcctttctcagagtggtt

ctgagattattttaatc--TAATTCTAAGGAATATGAGCTTTATGTAATAATGTGAATCatggtttttcttagtagattttaaaagttattaatatttta

atttaatcttccatggattttggtgggttttgaacataaagccttggatgtatatgtcatctcagtgctgtaaaaactgtgggatgctcctcccttctct

acctcatgggggtattgtataagtccttgcaagaatcagtgcaaagatttgctttaattgttaagatatgatgtccctatggaagcatattgttattata

taattacatatttgcatatgtatgactcccaaATTTTCACATAAAATAGATTTTTGTATAAcagctgccattcatggttttttaaaaggataaagtaata

aagctggtgggggtaaCAGGGTGACAAGGTGAAGGGCGTGGTTGCAGAGGTTGGGctcacattgtggagaaatgagcgcgtaatggactctgggctttcc

cccaggccaggctcacctgcatgttagggagaggcagccacaggctggggccagatgaacccaccccccagctccagatctgACATCTGCTTCAGCCACT

TGTTCAAATACCTTTCTTTTTTcccaaactcattctttttcTCACCATCCCACGCTCTACCCAAAAGCCTGTCCCCTCTTCtattctatcagccttgtga

ctggctggttttcccccttctaatcaatcctctagtctagtaaatacaaatgtcactttcattaaaaaatcaagtaggagaccaacagatgatttggcaa

gtgcctgccatcattgatttctgccaccattggtgtt---------------------------------------------------------------

----------------------------------------------------------------------------------------------------

----------------------------------------------------------------------------------------------------

----------------------------------------------------------------------------------------------------

----------------------------------------------------------------------------------------------------

----------------------------------------------------------------------------------------------------

---------CAGACCAAGTCCAACATCCCTTTCTACCCCTTCTTCAGCCCACCAGTTCTCACCCCCAAACACCAAAGTCAACCTTAAACAATGAGATTCA

GAAAATGTGAATAAGTATTCAGTTCATTCAAGGACAAAGCTTGAGGGTGGCCACTTGGAAAAGCATCAACTCCAAACAACCGGAAAAGTTTCAGTTACAT

AAGTAAAGACAGAAGTTCCAGCCAGATTACAACATTTTTCATACTAGACAAGGTGCATACACCACAGTGATTTGATTGGTTATAGGTTGCTATGTTCTAA

GGAAGATTATTTGCTATTCCTTAAGGAGAGGTAATGATCTGAGGGGTCTTACCTCTGCCATGGCTCAGTCTTCTTAATTGTTTACAGGAAACAGCAAAAA

TGCAGAAGTTGTAGGTGCATGCCACACAACTCAGATTGCATAGCCACATTCCTCTCAAGGCTCAGAATAATTAAAGTCCTAATAGCTTTAAGTTTAAATT

ATTTGAAGTTTGACTTATTTAATTTAATTTCACACCAATAAGGAGGGTAATCTTAAATTCCAGGAGAGAACAACCAGCAGGCAGACCAGGAGTAGAGGTG

AATGAATAGATACTGTGAGACACAGTATTTGATACATGCAAATATTAAGCAGAGTGTTTGATGGTAGGAcaggaAAGGAGAGAAGGGCTACATGTGCTCA

AGGTCAAACAGAATc-----------------T---GCTGAAAC--TTCATTTTCAAAAAATTATAAAATCATGTGTTTTCATGCAGGCCttgtactctt

gg----------GCACAGAGAATTAGAAAGAA--CAGGAAC-AGAG-AGGCTGGTATATAACTGGGCATTGAGTTCAATGGGATTCAAAGATTAGgctag

ttacaccagcAATGTGCCTAGTAAATTTAGGCAAGTTACTTTACCTGAGCAAGCCTCC---ATAGATT-G-AGATGT-AGAGTAG-GTCAATGAGTCGTA

TTC--ACATCAACA--TCACTTAGATG-CTA--ATAA-AAAAACAC-----------AGA--TT---T------C---CAGAT-ACC-ATCA-G------

--CAAAGACTCTGATATGGAGTGTTGGGTGGGGAAGGTGTCACTTACACATTTTTAACTATTTCTCAGTCATGTCTTGGGACCAGT-----G--AG-TTT

-------CCCTGACACTCTCTTTTTCAGATCAATGTAGTCTCCAGGCTTGATCTGTGACCTCTGGCTTAGGCGTAAGGAGTGTTTTTTGCTGGAACTTTC

CCACCTCCTAGACTTTGCTGCTGGACATGtgccagtcat-AAGTAGCAGAGACTGCAAATGTCCCCCAGATAtctgTGGGCTCTTCTATATTCTCCAGCC

CAAATTGCATTTacccaGGGGCTGTGTGATTGAATTCTAACCAGTTGGGTGAGGG------CTAAAATGATATGAGCCACTTTCAGACCTTGTC-CCTAT

AAACAGCTC----------TCTCTTCCTCTGTTG-TGGTAATGTTAGGGGTAATGTGTTCCATATAGCTCAATTTCAAGATGGAAGCAGTCCATATCcac

aagtcactggtcac------------------CTGGCCTACCTTGAACTGTGACATGAGAAAGAAATAAACATTTGCTGTGTCAAGCCACTAA-GCCTAG

TTTGGAGCCTATTTTAACCCTGATTAATAGAGGAATTagtagcacaatcatGTGCTACCATTAAGAACATGAAAAATGTAGCATTAACTAGATATTTAGT

AGCAGGCAGTGAGGAAGCTGATGTCACAGTTTGAAGAAAATAAGAGATTCATGTTACAAACTGTCAAACCTGTCAGGTGCTATCTGCTGAAATAGCAGCT

CCAAGGGGAAAGTTTGATAAAGATACATTAGTAGCATGTTGATTGTTTTTG-TCTTCCTTTTTGAAGGGATTCCGAGAAAGAGATGAGCTCAGAAAAAAA

AATCAACTGGTCATAAGGTAGAAATGAAAGATAATAAAATCCACAAATCCAGGACCTTATGTTGTTGGAA-AAGCCATCTGTTTCTGGGTCCCAAATATT

AAGAGAAGGTATTTAAGGATGAAAAAAAATCAGCAATAAAAGTCTATGGGGAAAAAAAAAAAACTCTTAGTTGGATGAAATAACATAGGGCAAAAATCAG

ATTGAAGTTGTAGCCTAACAT-TCAAGCCT-CACAGCCTTCCCTATTAAGAAGAGAAGGAGAAGGAGAAGGAGAAGAAGAAGGAGAAGGAGAAGGAGAAG

GAGAAGGAGAAGGAGAAGGAGAGGTAGAGGGAGAGGGAGAGAGAGAGAGGaaaaataattggaaaattataccaagaaagaaacttgggctgtggctgct

ggcatatggaattgactgagagtgaagatatcagaagcccactgttattaccagagctgtactatattgtcagtgaaaccatgagtggtcctgacaagtt

caaaaagaagacaaactccaggccttgctgtacagcttgcagtcctcaaagacagcagagcccccaagaagagcatgtctctcttcaatactcacttcag

aggtgaccaaaggggaataatggaaaaggagggaactcacaaaggaaaccagaaaccaggatcaacaagacaacagagctcccccagaaagagagaacca

gtctaatcaaggatgggggactttttttttctttttattatactttaagttctcgggtgtgtgtgcacaacgtgcaggtttgttacataggtatacatgt

gccatgttggtttgctgcactcatcagcttgtcatttactgtaggtatttctcctaatgctatccctcccccagccccccaccctccaaaaggccccggt

gtgtgatgttctggatggcagacttttataacatctgtctgaaggattttaggcttggcacagtgcattggccattgatgaatgccatctttctttccat

aaatgagagtgttgattacaagtaccctgtccttttcaaacttgaaactttaggtaagtgacaaattatttgtctttttagctcatagggtcactggaca

aagtagatccataatctcagctgatggagataactaaccatcatcaagagatcctgtccatttaggttggtgcaatgactaaatggcaccttgggttgtc

tctcttgtgggtggattatttgtgtattctacatgtgagaagaagtaagcaaaaggcagattctaacagaaattattagtcttcaccaaatattcatgtg

atcctcttcatgtctcagctcctctgcagttattctggagttggatgatgagtgaaggccaatgtgaagtaggcagaagtgacgtaagcagcttttatgt

ctggttctttaaaacatcctacacaatctatcaaccctctctccactcacatctgggaacttggaagccatatgttccataggaaagctgtaattgacaa

acagcctgaatctatgagtcattccttacaagagagcacttttggagagctgcctaaccaacatcaggctacaatacaaacaaaaagtaaatattgcagt

aagccactgaggttgttgttgttatagaagcctagactagcattaccctggccagttagcccagaaaagtagctatgacattctgtgcctcagttttcca

cctctgtacaaaaaagtaaagttgattgccctaagaggattataacaaaaagaacattttaggccaggtactgtgtctcactcctgtaatcccagcactt

tgagaaaccaaagtggaggattacttgaagcaaggagttcaagaccagtctaggcagcaaagcgagacccccatctctacaaaaaataatattagacaga

tgtggtagcacacctatagtcctagtgactctggaggctgaggcaagaggatctcttgaggcccaggagttcgaggctacagtgagctatgatggtgcca

ctgcactccagcctggacgacagagtgagacaccatctctaacaaaataaaatattttaaaaattttttaaaaagaatattttaaataaaaaggctttgg

gaaacctcaaggcaatctgggaagcaggcttcactttcataaataatttcacagaagaggaagatatttaacttctctaagtctcctttctctcatcttt

aaaatggacttaattagaataagtttgttcggaggattaaataagtcatctcatgtgaaatgaagtaagatagagccttgcatagattgcataggttagc

attgccattgataacttactatctgctggacacattgttagcatgtttacactaacatcgagtttggtgtaattctcttccaaaagtgataagaaactgc

agaaatgagctcagtgatgcttgagaaggaggagtaatgaaccctacgttggtcctcttttgcacaaagggagattgagactaagagttacgaaacattt

cagaagacagggagtcaaccaacaggggctctagaatcaactcagcctaaagcacacagttcccttaaggattttttcactagtctccaagcctctggtg

tctattcttttatttttattttttaaAGAGAGGGTTGGAATAAAATCAACGTCAATGTCTCtttccagtttgtctataactttgcaatattagaggagat

ctggaacacagctacagaacaaagacaggagtcctgattcctgggatccttgtctgctgatcactgatcactcacctctccctgaaaaattccttttttt

ccttttttctttcttttttcttttcttttattttttctttttagaagtcagccgggaagttcgtcctgggcagaacctacagtagcacagcaaagccgct

gtagcctgactgcctctgttgtttcatctgggcagggtatctctgaaagaaaggcagcagcctcagttacaggcttatagataaaattcccatctccctg

ggacagagcacctcggggaagggttggctgtgggcacagcttcagcatacttaaacgttcctgcctgccagctctgaagagagcagagggtctcccagca

cagcacttgagctctgctaagggacagactgcctcctcaagtgggtcactgaccccagtgcctcctgactgtgagacacctcccagcaggggtcaacaga

tgcctcacacaggagagctccagctggcatctggcaggtgcccctctggaatgaagcttccagagaaaggagcaggcagcaatctttgctgttctgcagc

atccgctggtaatacccaggtaaacaagtctggagtggacctccagcaaactccagcagacctacagaagaggggccgactgttagaaggaaaattaaca

aacagaaagcaataacatcagcatcaacaaaatggatgcccacacaaaaaccccatccaaaggtcatcagcatcaaagatcaaaggtagataaatccaca

aagatgaggaaaaaaaagagcaaaaatattgaaaattccagaaaccagaatgcctcttctactccaaaggatcacaacccctcgccagcaaggaaacaaa

actggacagagaatgagtttgacaaattgacagaagtaggcttcggaaggtgggtaataacaaactccttcgagctaaaggagcatgttctagcccattg

caagtaagctaagaacctcgataaaaggttacaggaactgctaactagaataaccagtttagagaagaacataaatgacttgatggagctaaaaagaaaa

gcacgagaactctgtgaagcatatacaactatcgatagccgaattgatcaagaggaagaaaggatatgagagattgaagatcaacttaatgtaataaact

gtgaagacaagattagaggaaaaaaagagtgaaaaggaatgaacaaagcctccaagaaatataggactatgtgaaaagaccaaacctatgactgattggt

gtacctgaaagtaacggggagaatgcaacgaagttggaaaacacacttcaggatattatccaggataattttcccaactagcaagacaggccaacattcc

aattcaggaaataacagagaacaccactaagatactccttgagaagagcaaccccaagacacataatcatcagattcaccaaggttgaaatgaagaaaaa

aaatgctaagggcagccagagagagaaaggtcaggttacccacaaagagaaactcatcagactaacagtggatctctctgcagaaatactacaagccaga

agagagtgggggccaatattcaacattcttaaagaaaagaattttcaacccagaatttcatatccagccaaactaagctccacaagagaaggagaaataa

aatcctttaccgacaagcaaacgccaagagattttttcaccaccaggcctgccttacaagatcccctgaaggaagcactaaatatggaaaggaaaaaccg

atacaagccactgcaaaaacataccaaaatgtaaagaccaatgacactatgaggaaagtacatcatgatgacaggatcaaactcacacataacaatatta

gccttaaatgtaaatggactagaggccccaattaagagacacagactggcaaattggatgagtcgaaacccattggtgtgctatattcaggagacccatc

tcacataaaaagacacacataggctcaaaataaagggatggaggaatatttaccaagcaactgaaaagcaaAAAATAAATAAATAATTAAAAAATAAAAA

AGCagggcttacaatcctagtctctgataaaacaggctttaagccaacaaagatcagaaaagacaaagaagggcattacatgatggtaaagggatcaatg

caacaaaaagagctaactatcctaaatatatatgcacagaatacaggagcacccagattcataaagcaagttcttagagacctataaagagacttagact

cccacacaataatagtgggagactttaacaccccactatcaatattagacagatcaaggagacagaatattaacaaggatattcaggacttgaactcagc

tctggaccaagcggacctaatagacacctacagaactctccaccccagatcaacagaatatgcattcttctcagcacctcatagcacttattctaaaatt

gaacacacaattggaagtaaaacacgccttagcaaatgcaaaagaacggaaatcataacaaacagtctctcagaccacagtgcaatgaaattagaactca

ggattaagaaactcactcaaaacagcacaactacatggaaactgaacaacctgctcctgaatgactcctgggtaaataacaaaattaaggcagatataaa

taagttatctgaaaccaatgagaacaaagacaaaatgtacctgaattcctgagacacagctaaagctttagagggaaatttgtagcactaaatacccaca

tgagagagcaggaaagaactaaaatcaacaacctaacatcacagttaaaagagctggagaagcaagagcaaacaaattcacaagctagcagaagacaaga

aataactaagataagagcagaactgaaggagatagagacatgaaaaacccttcaacaaatcaatgaatccaggagctggtttttttgaaaaaattaacaa

aatagatagactgctaaccagactaataaagaagaaaagtgaaaagaatcaaatagagacaataaaaaatgataaaggggagatcaccactgatcccaca

gaaatacaaactaccatcagagaataccataaacacctctacacaaataaactagaaaatctagaagaaaaggaaaaattcctggacacatacacactcc

caagactaaaccaggaagaagtcaaatccctgaacagaccaataacaagttctgaaatggaggcagtaattaatagcctaccaaccaaaaaaaggccagg

atcagacgaattcacagccgaaatctaccagagatataaagaggagatggtaccattccttctgaaactattccaaacaatagaaaaagagagactcctc

cctaactcattttatgaggccagcatcatcctcataccaaaacctggcaaagacacaacaaaaaaagaaaatttcaggccaatatccttgatgaacattg

atgcaaaaatcctcaataaaatactggcaaagcgaatccagcagcacaacaaaaagcttatccaccacaatcgagtccgcttcatccctgggatgcaagt

ctctttcaacatatgcaaatcaataaacataatccatcacataaacagaaccaatgacaaaaaaaccacatgattatctcaatagatgcagaaaaggcct

tcaataaaattcaacacctcttcatgttaaaaacactcaataaactaggtattgatgcaacatatctcaaaattgtaagagctatttatgacaaactcac

agccaatatcatactgaatgggcaaaagctggaagcattccctttgaaaaccagcagaagacaaggatgctctctctcaccattcctgttcaacatcgta

ttggaagttctggccagggcaatcaagcaagagagagaaataaagtgtattcaaataggaagagaggaagtcaaattgtctctgtttgcagatgacatga

ttatatatttagaaaaccctgtagtctcagcccaaaaactccttaagctcataagcaacttcagcaaagtctcaggatacaacatcaatgtgcaaaagtc

acaagcattcctatacaccaataatagacaaacagagagccaagtcatgagtgaactcccgttcacaattgctacaacgagaatagaatacctaggaaca

caaattgcaagggatctgaaggacctcttcaaggagaactacaaaccactgctcaacaaaatgaaagaacacaaacaaatggaaaaacattccatgctca

tggatagggagaatcaatattgtaaaaatggccatactgcccaaagtaatttatagattcagtgctattcccatcaagctaccattgactttctttgcag

aattagaaaaaactactttaaatttcatatgaaaccaaaaaagaccccatatagccaagacattcctaagcaaaaagatcaaagctggaggcatcatgct

acctgacttcaaactatgcaacaagcctacaataacaaaaacagcaaggtactggtaccaaaacagacatatagaccaatggaacagaacagagcctcag

aaataacaccacaaatctacaaccatgtgatctttgacaaatctgacaagaacaagcaatgcggaaagggctccctatttaataaatagtgttgggaaaa

ctggctagccatatacagaaaactgaaactggaccccttccttacaccttatacaaaaattaactcaagatggattaaagacttaaaggcaagacataaa

accataaaaaccctagaagaaaacccaggcaataccattcaggagataagcatgagcaaagtcttcatgactaaaacaccaaaagcaatggcaacaaaag

ccaaaattgacaagcgggatctaattaaactaaagagcttcttcacagcaaaagaaactatcgtcagagtgaacaggcaacctacagaatgggacaaaat

ttttgcaatctatccacccaacaaagggctaatatccagaatctacaaggaccttaaaaacatttacaagaaaaaaaaatcccatcaaaaagtgagtgaa

ggatatgaacagacacttctcaaaagaagacatttatgtgatcaacaaacatatgaaaaaaagctcatcatcactggtcattagagaaatgcaaatcata

accacaatgagataccatcacatgccagttagaatgtcaatcattaaaaagtcaggaaacaacagatgctggaaaggatgtagagaaataggaacacttt

tacactgttggtgggaggagtataaattagttcaaccgttgtggaagacagtgtggtgattcctcaaggatctagaatcagaaataccatttgacccaac

aatcccattaccgggtatataaggattataaatcattctattgtaaagaaacatgtacacgttatgtttactgcagcactatttgcaatagcaaagactt

caaccaacccaaatgcccatcaataatagactggataaagaaaacgtggcatatatacaccatggaatactatgcagccataaaaaggaatgagttcatg

tccgttgcagggacgtggatgaagctggaagccatcactctcagcaaactaacacaggaacagaaaaccaaacactgcatgctctcactcgtaagtggga

gttgaacaatgagtacacatggaaacagggaggggaacatcacacactggggcctgtcggtggatggcaggcaaggggagggagagcattaggacaaata

cctaatgtagatgacgagttgatgggtgcagcaaaccaccatggcacatgtatacctatgtaacaaacctgcacgctctgcacatgtatctcagaactta

aagaataataataaaaataaaagataccttttccaattaaaaaaaagaaaaAAAACTCTTTAGAAGTCAATATTTTGTTTTCACAAggggGGATCCATTT

CCTGCCCATCACTCAGGATCATTCCTgatcAAGGCCCCTCATTCATCAGGCACCAAATCACAGGCaagaaattggtctactctacttcattgctgaaaag

aaagcctccaagaaagaaagagaaaaagcccttgaagctgcccctgcttgctagggatggccagtgagctggatgaaacagctgcagagCTTGGATATCT

AAAGATTGATTTTATTTATTTCCACTGAGCCACTGAGGTTTTTCAGGTGTAGAGTAAATCCAAAGCCCATTAACTCCAAAGCCATTTTTCAAGGAAAACA

CAA-AAATGAATAATCGCTTGCTGATAAT-TCC-TTTAAATTCCCGCCAGAGTTATGAAATTATT-CTTACTACAG-AAACAATCATTAAACCACGTCAG

ACAAA-CACCTCTGAGGTCAATAATATTC----CAAATTTATT-C--TGCCGAGAATTTTTTAAAACTTCGTATCTGCCACATGTGCAACTCAG-GCATT

CTTGGAAACCATCCCTGCGGTAGCATATCTTTACTTGGAAGCAGTGGGAAAACTTGGAAGAAT-ATTTGGAGAAAT-ATC----CAGACTCTGAGG--TC

TTTCCAGCAT-TCCCAATCTGCCCT--TCTTTTTTTTTTTTTTTTGA-GACACAGTCTCACTCTGTTGCCCAGGTTG--GAGTGCA-GTGGCATGATCT-

-CTGCCCATTGCAA-C-CTTTGTCTCCTGTGTTC-AAGCAATT-CTCATGCCTCAGC-----TT-C-CTGAGTAGCTGAGACT---ACA-AA-----CA-

CC----ATCCAC----CA--TGGC-----TGGCTAATTTTTTGT--ATTTTTAGTAAAGA--CAGG--TTTTCACCATGTTGGCCAGGCTGGTCTTGAAC

TCCT-GACCTCAAGT-GATCTGC-CTGCCT---CAGTCTCC--CAAAGTGCTGGGATTACAAGTGTGAGCCACTGTA-CCCGGCCCTATTTCCTAATAAT

AAAAAA-CAGTAACTTCTACTCACTGAGTACCTA---CAGTG-ATT-TAGTCATT--CTTCTAAGCAGTTTAGATACATGAACTCATTTAATTCACACGA

CAATTCCCTAAAGTACACATGATTAGTATCCTGC-TCTTACCCTAGC--------AGAGAGT-GGT------------TAAGTAAGTTGTCCATGGTATC

ACAGCTAGTCAGTCACAGAGCCATCATCCAAATGCAGATATCCTGAATTCAGGTTCTACATTAGACTAACCCACCAGGAATGGAGCAGGAAAGAACAGGG

AAGACTCCACATTTTTGGCCTCTATTTGGTAATTATAGTTAACTTTTTAGGTAATTATAGACCAATTATCCTAGATGGGCACTTAGAGACTTTGCAGGAC

AGCAAGAGCTGTCTCTAATCCTGTGCCCATGACAGACATCACCAGTCAACCACAACACAGTATTTAACTAACGCAAGTCAACTCCTCAGAAT--CT-TTA

ACATTCTTGTTTGTGCTACTGTACCAATCAATCAATTTGATATGAGAGTGTGCAGGAAAAAACAGGAAACAGGTTTGCAGTACCTCCACACCAGTATTCA

ATGCTGTAATCCGCTGCAGTGACTCCATTAAAGACTTTGCCTCC-CTTATACCCTCTCCAACTAGGGTGCCTA-GTGTTATGAACAAAGGGATATGTATA

GGTTCTTGTGTTGCCTCTCTCTTTGATATTTTTAGCCATCAGATACCTTGTCTGCAATGTGTGCTCAGAGAG-TGAGGGGGGAACTAGATGATTGATTTT

CCAAATGTGTTCCCTAAATGTGTTCCCTGGGGAATAAGGGCACGAGAGGCTGCCTATTCTATTTCAAACAAATCCCCTTCACTACAGTGTATTTGATGAG

TTGGGGTTTGTTTTAATTCCATTTGGAAAAGGGCTTTAGCAGCTAAGCAAATGGTTTTAAAGTGCctcaGAAGTCAAGATTAATAGAAACTATCCAGTTC

TGATGTCCTatcatgctaaaatttcagggactaagattctgtgatcattacattgaaacacagcagcaaagctgtggtgtgttgtccttcctggttcaga

gatgcaactatgtgcagggctgctgagctCTCTCTGCATCTGGGTGGGAGCCTAATGGAAGTTTTGGGGctccttcctggtctccaaAATCCTCAAGACC

ACCATGTGAACACAGGAATCAAGGAAGGTTCTTAGATCGACTCATCCCC-CAGGCCTTTGGTTTC-CTTGCTCCTTTCCCCAACTACAGGTGTTTCATTT

CAACTCATCCCCTAGGGCCTTGGTTTTCTTGCTCTCTTCCCCCACTACAGATGTTTAACTTCATTTCATAACCACATATTCCCCTCCTTTTCCAAGGCAA

GATCCAGA-TGGATTAAAAAATGTACCAAGTCCCTCCTACTAGCTTGCCTCTCTTCTGTTC-TGCTTGACTTCCTAGGATCTGGAATCTGGTCAGCAAT-

CAG---G-------------A-ATCCC-------TTC-ATCGTGACCCCCGCATGGGCAAAGGCTTCCCTGGAATCTCCCACACTGTCTGCTCCCTATAA

AAGGCAGGCAGATGGGCCAGAGGAGCAGAGAGGCTGAGACCAACCCAGAAACCACCACCTCTCACGCCAAAGCTCA-CACCTTCAGCCTCCAAC-ATGAA

GGTCTCCGCAGCACTTCTGTGGCTGCTGCTCATAGCAGCTGCCTTCAGCCCCCAGGGGCTCGCTGGGCCAG-GTAAGCCCCCCAACTCCTTACAGGAAAG

GTAAGGTAACCACCTCCAGAGCTACTAGGTCAGCAAGAATCTTTACAGACTCACTGCAAATTCTCCATTTGAAAAATAGGGAAACAGGTTTTGTGGGTGG

ACAAGAAATGCCTCAACCTCACATCCAGTCACTGGAAGAGCCAGAACTAGAAA-----GC--TCCC---GAGTC----T---TTT-CCCC-ACAT-----

TCAAGAGGGTTGCTGGGTGCATCCATACCCAGCTATCCTTACAGTGTTTGGGAATGGGG------AATG---GCTCTG-TCTTACTGTGGGCATGGTGGG

CATTTTTGGCAGTGGGAGAGAAGGAAAA-TCTGTTGATTAGAAGCTCAGTATGTTAATTCGACTCCAGGACAGCTTTCAGAGACAGTGGCTAAGAGAAGa

acgaggtcccaggggg--ATCTCTTGAGGTGACTTATTTTGACACTCTTTGGGAAAgttatctaggagATTTGTTCCATAACTCATTTTCCCATACTCTG

GTGACAAAT--TTACTGAGTGTATCGGTCCCACTGA-GCCAGTGCATAGCATGGTAACAAACAGTTCTAAATTATCAATGACTTAACAGAAttaactAAA

TTAACAAAAGTTACTTTCTCACTTGTACTAAATatctataATGTATGGGCTCAGGCTTCTGCATTTTATACTCAGGATTCtagactgatggagaagttgc

ccaTGTGGGGGAACATTGATGGATACTGTGATAAAGCAGAAGAAAGCTCTCAGGAGTCTTGCATAGGCAATGCACTGTGGCTCAAAAATGACACccatca

ctttgtctccttctttattgatcaaaactaattaatgcctccAACCAAACAAAAGTGGCCAAGAaatgcaagtctaccttgtgtCTCAAAACAGAGGATG

GAGAATATTTGGTGAAAATTACCATGACCATCACATGGCCACGTAGGTCTTTATAATGACAGAGCTAGCATTTGTCACATTGACCAAGCTTTGTCCATAC

ACTCTACAGTAATGATGAGTCCTCAGTGCACAGGGGAGGATGCTGAAGACACAGGACAGCATCCTCCAGACACATAAGACTTCAGAGCAGAGGGATTCTC

CCTCCACCTCTCGCAATTCCTTGCTTTCTCCTAAC----TTCCT---TTACAAAGTCATGCTTGGAAATGTCTATGTATCATCATGTGGCTCATTTTTTT

CTCTGTTCATTTTTTTTCCCCAAAATTCAG-CTTCTGTCCCAACCACCTGCTGctttaacctggccaatagGAAGATACCCCTTCAGCGACTAGAGAGCT

ACAGGAGAATCaccagtggcaaatgtCCCCAGAAAGCTGTGAT-GTAAGTAAATAAAGTTCACCCTCcCCTAGACAAAAAAATAATGTCTAGGGCACAGA

GTCAAGAACTGTGTCACAGTTGCTGGGAGTCATAGACTCtgatagTTTGACCTCTATGGTCCAATTCATTAATTTTCACAAgtgag--TGTTCACTCCCA

GCTCCCTGCCTGG---GA--GATTGC-TGT--AG-TCATATCAATTTCTTCAAGTCAAGAGCAAAGATGGTTTTACTGGGCCTTTAAGAGCAGCAACTAA

C-CCAAGAGTCTCATCC--TTCCTCCTCTCC-GTAGCAACCCTTTGTCCAGGGGCAGATGGTCCTTAAATATTTAGGGTCAAATGGGCAGAATTTTCAAA

AACAATCCTTCCAATTGCatcctgtatctccc----ACAG---CTTCAAGACCAAACTGGCCAAGGATATCTGTGCcGACCCCAAGAAGAAGTGGGTGCA

GGATTCCATGAAGTAtCTGGACCAAAAATCTCCAACTCCAAAGCCATAa---ATAATCACCATTTTTGAAACCAAACCAGAGCCTGAGTGTTGCCTAATT

TGTTTTCCCTTCTTACA-----------------------------------------------------------------------------------

----------------------------------------------------------------------------------------------------

----------------------------------------------------------------------------------------------------

----------------------------------ATGCATTCTGAGGTAACCTCATTATCAGTCCAAAGGGcatgggttttattatatatatatattttt

tttTTTAAAAAAAAAACGTATTGCATTTAATTTATTGAGGCTTTAAAACTTATCCTCCATGAATATCAGTTATTTTTAAACTGTAAAGCTTTGtgcagat

tctttaccccctggGAGCCCCAATTCGATCCCCTGTCACGTGTGGGCAATGttccccctctccTCTCTTCCTCCCTGGAATCTTGTAAAGGTCCTGGCAA

AGATGATCAGTATGAAAATGTCA------------------TTGTTCTTGTGAACCCAAAGTGTGACTCa-----TTAAATGGAAGTAAATgttgTTTTA

GGAATACATAAAGTATGTGCATATTTTATTAtagtcacta-GTTGTA-ATTTTTTTGTGGG-----AAATCCACACTGAGCTGAGGGGGACAAAGATGGC

TGTGGCCAAGAGGGGCTTGGTTAAGGGGGTGGGAACTATGTCCCTGGGAAATGAGTTTTTGGCTTAGCTGGTCTtcATTGAAATGCAGGGTGAAACTGAC

A---------AACCCATTCCAGCCCTCT-ATTCCCATTTTCAACAGTATTTCCCA-GACCCCA-A-ACTTCAGCCACGAAAATATCTGGAGCTTTGCCAC

CATTCCTTTCCTCCCC-ACCTCATACT--T-----GCCTCTCCTGGGACCTATTTTAACAGCCCTGTGGTA-TCTCCCTCTACCCGACCCTGCTCCTCGG

TCTTCCAACCCAGACTAGGCAGACATGACAGACACTGGAAATCAGAGGAAGGCTAGGTGACCTTTGACCAGAGGTTTAggtgcaggTTTTACA----GGT

GTGGTGGAGCTTAAAGTGGGTCTTG-TGCATGCACAAAGTGGAGAGGCTCTGCTGGAGGTATTTCAGTCTAGGTAGAGGAGAAGGGGTGTTGCTTGGGCA

AAAT-CCAAGAAGTAGGAATGATCATGCTGCATTTACCCAGTTGGGTGGGAGCC-CATCAAAAGATGTCAGTGTTGGAGTATGTGTG-AAA---------

------TG---GTGG-GGAAATTTG--GAAAGAAGTTTGAGACCAGGCTGTCTAGGT-GG-GGAGTTTGTGCTC-----CAT-G---TA-----AGGTGA

ACATATGGTTCTTC-CCAGCCCAGTTAGGATGCTTTTG-AGAGCAGAAAAGTGGTATTATTCATAATTATTCT-GGAACAACGGATATAAATTAACACTG

TCCTAAGCAAACTGGAGCATGTGGTCACtctaggaatagaatgcattgcaagttttgattcagtgga---GAGAAGTGCCATGACCACACTATGAGGGGC

TTCAGGAAGGGCAGCCAGGTGGCcatgggCTGAATTGGGAGGAGAAGGGAGAAGCACTTAGCTCACTATTGCAGTGTATGGAAGA--TAT--------GG

AACTGGGGCATTGGTAGTTGGGA-GGGGAGAAGATAATTGGAAGAATGGCTGAGGC---AGGGTGGAATGCCAAATGTTG-TGGTTGAAGAAGTAGAACC

AATGACATGGAAACTTTCTAAATGC--CAAAGCTGCAATTAAAGGTTAGCTTCTTAGACCCAGAAAAttaatttctctctgtCACTGTTTACTCACACCT

TAACTTTACTAGCTTGAGAGCTTTCAATCAAATGGACAGGACCTCATCTTCCTGTATAAGCAACTTAAATCAGGATTCCCTTTCAGCATACAAAATCAGC

TTGCGTTTCCATTGGTCACATTA-CCTATGTACCTGAACGCAGAGACCAACTATGTCCCCAAGGGt-AGAAAAGGGTCACAGGTGCCCTGCTGGTATAAC

TctgcatggtctaaggccattgATCTGGAGGGCCAATGGCCTACTGTGTCA-TGCATGCCCAGCAGGGTCGCTGCAT-GCAAAGCTCCCAGAGGCAC-CA

TTCATA--AGGCTGTCCCCCTTGG---AC--TGTGTGGTGGCAATGGCACAAGGCAATATAAAGAACAGAATGAGACATTAGGAAGGGCACTGATCTAGG

AGGCAGATGACCTCAGTT-TCATCACAGACAGAGA-ACTGGTCTCTGTCACTGTGTTACTTTTGGCTTTAGTTACCCCATCCA-TAATAGGGGTATATTA

ATATC--TTTTAAGG-GATCT--GACCAA-TGACCCACCAAGATCTCCTGTTCTGAGAGCACTACCTCTTGGAAAGCATCACT--ACCTCCACCCATTGA

GGGAGGG-AATGTTGTCAGAGGGTTCCACACTCAC--AAGCCCAAGTCGGACAGGGGCCA-GGAT-AACTGGCACACTTACTCTATTTCTAGAAGATTTC

TCTGTGAGAGCC--G--CAAGTAGAAGCA-TGGAT-C---CCTAGA-AC-CATCAG--AG---AGCTTTTTGTATCTACTACAGAAGGGC--TCCCACTC

AGAGCAATCACT--GGCCCT-CACTCTGAGTACAAAAAACC----TCTCTGCCT--CTCCTATAGCCTAGCAGACTTTAGCCCTAAACATCAATCAGGAT

GTACCT-CCAGAGGTCAGCATGTCCAGGAG-GCATGGGATGGCCACAG-AATAGCTCACTGGGATCAGTAGACCCTCAGGTTTCA-GGTC-AGTCTTCAC

GAGGAGATCAAGAAGGGGAGTAGAGGGAAGAGGTGCCCTCTGTTTGTGAAGGACTGAACAGAATTTGCTCATATCCCCTattcctctctcaaggaatcct

gaacctaattaccctacaattgggaatagcatcaccagggcctt----------GAGTATAAAACTGTGGGCACAAAGCATTGGAGGTGTTTAAgtTTTC

TGATGGCACACAGATCTCAACCAGGAAtCAGGGATGTCACCAACTCATTTCTCTGGAAAGCATACTATTAGATGAAGATGTTCATG--CAGGGTTCACTG

-AGAGTATGGGGAAGCTGAACTAAGGAGAGGGAGAAT-TGATATTGTGGTGCAATTGCAGCCCAGGTCTCAG-CAATCCCATAGGGAGCTTTAGAACTGG

gatagcaCAAATGAGGTCGAGGGGCCAGGCTTTTATACCCCCATCTgaaccagcCCTGGCTGCAGGATGCTCCTGGAAGAGGACACAACTCTGGgcaagg

cagctgtttttagctgacggcggGCCTCATAGAGGAAATTCCGCTGAGAAGCATTGGCTGCC----------------------AAC-ACTTCTAGCAGC

TGGGGAAGAAGCACT----T--TAGA--CCTGGGAGGGAAAAACTTGGGCAGCACACTATGAACTCCACCATGACCCACCCCTTGTGCCATTCAAATCCA

CTTGCTTATATACAAAGTTCTGGAAAACTCATCCA---GAAA-----------TCTGTTGAGCCTCATTTTTTAAGGAAACTTGCAAGAGGAAGGTTGGT

GGAGTGAACAGTAGCCA-CCTACAACAGCTGCAACTGGTCTTGAGGTTATAAATGATGCTTATCACCCCACTCCTAGAACACTCATTCTAGATTTTCCTC

AACCTCAGCCCATGCCTCTGCTGGC-CTAAGTGGCTTGCCTT-CAGGATTCACCCAGACCCTCATCCCTGAGGGGTTCA-GCC-CCTGGT--CT----TC

ACACTCCTCTCAGGACAAGGATATGGCTGCTACT-GTTGTCCATTTGCCATTAAAATTGG----GTCAAGAGACATCCAAATGGGTCA-C---CCAGATG

CCAA-ACATAT-TCTTATCTAACCCCATTGAGTAAAAGCAGCTCCA-CTCACTTCTGATGACCAGGGCCCATTACCCTGGCCCGGATAGTGACCTCTTCC

TCTCCTTGCCTGTTAGTTTCTTGGCCTGCAA--GTCTA-AAGAGATAGAGTGCAGCCACAGCTGAGGATTA-ATGAGACTCCCTCTAGGAACCAAGGGGT

AAGCATTCTTCC-TCTGG-GA-AACAGGATTTCTAAATCCG--TACAGCCC-TGATGTGCACAAATCCCCCAAAGTGGATCACTAGGAGTAATGGTAAGT

AGGGCAATTTTACTTCCACCTTTGACATGAATCTATGAATTTTACCTATTGGGAATACAGCTTCACTTATTATTTGATTTAAGGTCTATATTGAATCCTA

GAGGATAGTATTTCTTCCTTGTAGGATACTCTTTCCAAGCTGGTGCCTTAACTGTGCCTTCAGCAGGCCATTCCATTACTGTATCAGCCTGGAAACCTGT

GGATAACACAGGTATATAGTGGGATCAGTGGATCCGATGACCATACACCCAGTGCTGCACCTCATTTGCTGTAGTGTGTCTTCCAGTCTATAGTGATGTT

ATGTGGCATGTTGCGCCAGTGAACAAACACTTGTAAGCTCTCAGATAATGGTGCTGTCTAAGGTACTGGAGACAGAAATGGCAAACTCATACCTGGAAAG

TGTTTCTACTCCAGTTAAGATGAGTCATTGCTCTTTTCAGAGTAGAATAGGTGCAATGTAATCAAACATGTACTTTCCACCAAACGGCTGGTTAGTCTCC

CTGATGGATGGATGATGCCATAGCCGGGGATCAACAATGGGCTATGTTACTGGCAGGTTGGACATTCAGCTACAGGGCCACGTGTCATCTGAACTCCTGC

TACCATAATGACTTTGTTCATATTCTTACTGTCCCATTACTGTGGTGACAAATAACAGAAGCTGGCTGATGCCAACTGGCTGAGTCATCTTGTTACCTTA

GTCATTCTGTATGTCCAAATTCCTGATGGGATTAGTACCACATCTTTACATACTGTGTCCTGTCTCATAGGTTCGTCCACTTGCCTCTCTGCCAGAACCC

CTTTCCCCCAAAATTTTTACCTTTGTCTTTCCAGGCCCCTGCCCAATGATGCAGACCATTCAACACTATCTATCTACGAGTCCATATATATATATATATT

TTTTTTTATTTTTTTATTATACTTTAAGTTTTAGCGTACATGTGCACATTGTGCAGGTTAGTTACATATGTATACATGTGCCGTGCTGGTGCGCTGCACC

CACTAACTCGTCATCTAGCATTAGGTATATCTCCCAATGCTATCCCTCCCCCCTCCCCCAACCCCACCACAGTCCCCAGAGTGTGATATTCCCCTTCCTG

TGTCCATGTGATCTCATTGTTCAATTCCCACCTATGAGTGAGAATATGTGGTGTTTGGTTTTTTGTTCTTGCGACAGTTTGCTGAGAATGATGATTTCCA

ATTTCATCCATGTCCCTACAAAGGACGTGAACTCATCATTTTTTATGGCTGCATAGTATTCCATGGTGTATATGTGCCACATTTTCTTAATCCAGTCTAT

CATTGTTGGACATTTGGGTTGGTTCCAAGTCTTTGCTATTGTGAATAATGCCGCAATAAACATACGTGTGCATGTGTCTTTATAGCAGCATGATTTATAG

TCATTTGGGTATATACCCAGTAATGGGATGGCTGGGTCAAATGGTATTTCTAGTTCTAGATCCCTGAGGAATCACCACACTGACTTCCACGATGGTTGAA

CTAGTTTACAGTCCCATCAACAGTGTAAAAGTGTTCCTGTTTCTCCACATCCTCTCCAGCACCTGTTGTTTCCTGACTTTTTAATGATTGCCATTCTAAC

TGGTGTGAGATGGTATCTCATAGTGGTTTTGATTTGCATTTCTCTGATGGCCAGTGATGATGAGCATTTTTTCATGTGTTTTTTGGCTGCATAAATGTCT

TCTTTTGAGAAGTGTCTGTTCATGTCCTTCACCCACTTTTTGATGGGGTTGTTTGTTTTTTTCTTGTAAATTTGTTGGAGTTCATTGTAGATTCTGGATA

TTAGCCCTTTGTCAGATGAGTAGGTTGCGAAAATTTTCTCCCATTTTGTAGGTTGCCTGTTCACTCTGACGGTAGTTTCTTTTGCTGTGCAGAAGCTCTT

TATTTTAATTAGATCCCATTTGTCAATTTTGTCTTTTGTTGCCATTGCTTTTGGTGTTTTGGACATGAAGTCCT-TGCCCATGCCTAT-GTCCTGAATGG

TAATGCCTAGGTTTTCTTCTAGGGTTTTTATGGTTTTACGTCTAACGTTTAAATCTTTAATCCATCTTGAATTGATTTTTGTATAAGGTGTAAGGAAGGG

ATCCAGTTTCAGCTTTCTACATATGGCTAGCCAGTTTTCCCAGCACCATTTATTAAATAGGGAATCCTTTCCCCATTGCTTGTTTTTCTCAGGTTTGTCA

AAGATCAGATAGTTGTAGGTATGCGGCGTTATTTCTGAGGGCTCTGTT-CTGTTCCATTGATCTATATCTCTGTTTTGGTACCAGTACCATGCTGTTTTG

GTTACTGTAGCCTTGTAGTATAGTTTGAAGTCAGGTAGTGTGATGCCTCCAGCTTTGTTCTTTTGGCTTAGGATTGACTTGGCGATGCGGGCTCTTTTTT

GGTTCCATATGAACTTTAAAGTAGTTTTTTCCAATTCTGTGAAGAAAGTCATTGGTAGCTTGATGGGGATGGCATTGAATCTGTAAATTACCTTGGGCAG

TATGGCCATTTTCACGATATTGATTCTTCCTACCCATGAGCATGGAATGTTCTTCCATTTGTTTGTATCCTCTTTTATTTCTTTGAGCAGTGGTTTGTAG

TTCTCCTTGAAGAGGTCCTTCATGTCCCTTGTAAGTTGGATTCCTAGGTATTTTATTCTCTTTGAAGCAATTGTGA---ATGGGAGTTCACTCATGATTT

GGCTCTCTGTTTGTCTGTTGTTGGTGTATAAGAATGCTTG-TGATTTTTGCACATTGATTTTGTATTCTGAGACTTTGCTGAAGTTGCTTATCAGCTTAA

GGAGATTTTGGGCAGAGACAATGGGGTTTTCTAGATATATAATCATGTCGTCTGCAAACAGGGACAATTTGACTTCCTCTTTTCCTAATTGAATACCCTT

TATTTCCTTCTCCTGCCTGATTGCCCTGGCCAGAACTTCCAACACTATGTTGAATAGGAGTGGTGAGAGAGGGCATCCCTGTCTTGTGCCAGTTTTCAAA

GGGAATGCTTCCAGTTTTTGCCCATTCAGTATGATATTGGCTATGGGTTTGTCATAGATAGCTCTTAT-TAT-T--TTGAGATACGTCCCATCAATACCT

AATTTATTGAGAGTTTTTAGCATGAAGGGTTGTTGAATTTTGTCAAAGGCTTTTTCTGCATCTA---TTGAGATAATCATGTGGTTTTTGTCTTTGGCTC

TGTTTATATGCTGGATTACATTTATTGATTTGTGTATATTGAACCAGCCTTGCATCCCAGGGATGAAGCC-CACTTGATCATGGTGGATAAGCTTTTTGA

TGTGCTGCTGGATTCGGTTTGCCAGTATTTTATTGAGGATTTTTGCATCAATGTTCA-TCAAGGATATTGGTCTAAAATTCTCTTTTTTGGTTGTGTCTC

TGCCCGGCTTTGGTATCAGAATGATGCT-GGCCTCATCAAATGAGTTAGGGAGGATT-CCCTCTTTTTCTATTGATTGGAATAGTT---TCAGAAGGAAT

GGTACCAGTTCCTC--CTTGTACCTCTGGTAGAATTCGGCTGTGAATCCATCTGGTCCTGGACTCTTTTTGGTTGGTAAACTATTGATTATTGCCAC-AA

TTTCAGCTCCTG-TTATTGGTCTA-TTCAGAGATTCAACTTCTTCCTGGTTTAGTCTTGGGAGAGTGTATGTGTCGAGGAATTTATCCATTTCTTCTAGA

TTTTCTAGTTTATTTGCGTAGAGGTGTTTGTAGTATTCTCCGATGGTAGTTTGTATTTCTGTGGGATTGGTGGTGATATCCCCTTTATCATTTTTTATTG

CGTCTATTTGATTCTTCTCTCTTTTTTTCTTTATTAGTCTTGCTAGCGGTCTATCAATTTTGTTGATCCTTTCAAAAAACCAGCTCCTGGATTCATTGAT

TTTTTGAATGGTTTTTTGTGTCTCTATTTCCTTCAGTTCTGCTCTAATTTTAGTTATTTCTTGCCTTCTGCTAGCTTTTGACTGTGTTTGCTCTTGCTTT

TCTAGTTCTTTTAATTGTGATGTTAGGGTGTCAATTTTGGATCTTTCCTGCTTTCTCTTGTGGGCATTTAGTGCTATAAATTTCCCTCTACACACTGCTT

TGAATGCATCCCAGAGATTCTGGTATGTTTGTTCTCATTGGTTTCAAAGAACATCTTTATTTCTGCCTTCATTTCGTTATGTACCCAGTAGTCATTCAGG

AGGAGGTTGTTCAGTTTCCATGTAGTTGAGCGGCTTTGAGTGAGATTCTTAATCCTGAGTTCTAGTTTGATTGCACTGTGGTCTGAGAGATAGTTTATTA

TAATTTCTGTTCTTTTACATTTGCTGAGGAGAGTTTTACTTCCAACTATGTGACCAATTTTGGAATAGGTGTGGTGTGGTGCTGAAAAAAATGTATATTC

TGTTGATTTGGGGTGGAGAGTTCTGTAGATGTCTATTAGGTCCGCTTGGTGCAGAGCTGAGTTCAATTCCTGGGTATCCTTGTTGACTTTCTGTCTCGTT

GATCTGTCTAATGTTGACAGTGGGGTGTTAAAGTCTCCCATTATTAATGTGTGGGAGTCTAAGTCTCTTTGTAGGTCACTCAGGACTTGCTTTATGAATC

TGGGTGCTCCTGTATTGGGTGCATATATATTTAGGATAGTTAGCT-CTTCTT-GTTGAA-T-TG-ATCCCTTTACCATTATGTAATGGCCTTCTTTGTCT

CTTTTGATCTTTGTTGGTTTAAAGTCTGTTTTATCAGAGACTAGGATTGCAACCCCTGCCTTTTTTTGTTTTCCATTTGCTTGGTAGATCTTCCTCCATC

CTTTTATTTTGAGCCTATGTGTGTCTCTGCACGTGAGATGGGTTTCCTGAATACAGCACACTGATGGGTCTTGACTCTTTATCCAATTTGCCAGTCTGTG

TCTTTTAATTGGAGAATTTAGTCCATTTACATTTAAAGTTAATATTGTTATGTGTGCATTTGATCCTGTCATTATGATGTTAGCTGGTGATTTTGCTCGT

TAGTTGATGCAGTTTCTTCCTAGTCTCGATGGTCTTTACATTTTGGCATGATTTTGCAGCAGCTGGTACCGGTTGTTCCTTTCCATGTTTAGCGCTTCCT

TCAGGAGCTCTTTTAGGGCAGGCCTGGTGGTGACAAAATCTCTCAGCGTTTGCTTGTCTGTAAAGTATTTAATTTCTCCTTCACTTATGAAGCTTAGTTT

GGCTGGATATGAAATTCTGGGTTGAAAATTCTTTTCTTTAAGAATGTTGAATATTGGCCCCCACTCTCTTCTGGCTTGTAGGGTTTCTGCCGAGAGATCT

GCTGTTAGTCTGATGGGCTTCCCTTTGAGGGTAACCCAACCTTTCTCTCTGGCTGCCCTTAACATTTTTTCCTTCATTTCAACTTTGGTG-AATCTGACA

AT-TATGTGT-CTTGGAGTTGCCCTGCTCGAGGAGTATCTTTGTGGCGTTCTCTGTATTTCCTGAATCTGAACTTTGGCCTGCCTTGCTAGATTGGGGAA

GTTCTCCTGGATAAT-ATACT-GCAGAGTGTTTTCCAACTTGGTTCCATTCTCCCCATCACTTTCAGGTACACCAATCAGACGTAGATTTGGTCTTTTCA

CACAGTCCCATATTTCTTGGAGGCTTTGCTCATTTCTTTTTATTCTTTTTTTCTCTAAACTTCCCTTCTCGCTTCATTTCATTCATTTCATCTTCCATTG

CTGATACCCTTTCTTCCAGTTGATCGCATCAGCTCCTGAGGCTTCTGCATTCTTCACGTAGTTCTCGAGCCTTGGTTTTCAGCTCCATCAGCTCCTTTAA

GCACTTCTCTGTATTGGTTATTCTAGTTATACATTCTTCTAAATTTTTTTCAAAGTTTTCAACTTCTT-TGCCTTTGGTTTGAATGTCCTCCCATAGCTC

AGAGTAATTTGATCATC-TGAAGCCTTCTTCTCTCAGCTCGTCAAAGTCATTCTCCATCCAGCTTTGTTCCGTTGCTGGTGAGGAACTGCGTTCCTTTGG

AGGAGGAGAGGCGCTCTGCATTTTAGAGTTTCCAGTTTTTCTGTTCTGTTTTTTCCCCATCTTTGTGGTTTTATCTACTTTTGGTCTTTGATGATGATGA

TGTACAGATGGGTTTTCGGTGTGGTTGTC-CTT-TCTGTTTGTTAGTTTTCCTTCTAACAGGCAGGACCCTCAGCTGCAGGTCTGTTGGAATACCCTGCC

TTGTGAGGTGTCAGTGTGCCCCTGCTGGGGGGTGCCTCCCAGTTAGGCTGCTTGGGGGTCAGGGGTCAGGGACCCACTTGAGGAGGCAGTCTG-CCGGTT

CTCAGATCTCCAGCTGCGTGCTGGGAGAACCACTGCTCTCTTCAAAGCTGTCAGACAGGGACATTTAAGTCTGCAGAGGTTACTGCTGTCTTTTTGTTTG

TCTG--TG-CCCTGCCCCCAGAGGTGGAGCCTACAGTGGCAGGCAGGCCTCCTTGAACTGTGGTGGGCTCCACCCAGGAGCTTCCCGGCTGCTTTGTTTA

CCTAAGCAAGCCTGGGCAATGGCGGGTGCCCCTCCCCCAGCCTCGTTGCCGCCTTGCAGTTTGATCTCAG--ACTGCTGTGCTAGCAATCAGCGAGACTC

CGTGGGCGTAGGACCCTCCGA-GCCAGGTGTGGCATATAGTCTCGTGGTGCGCCGTTTTTTAAGCCGGCCTGAAAAGCGCAATATTCGGGTGGTAGTGAC

CCAATTTTCCAGGTGCGTCCGTCACCCCTTTCTTTGACTCGGAAAGGGAACTCCCTGACCCCTTGCGCTTCCCAGGTGAGGCAATGCCTCGCCCTGCTTT

GGCTCGCGCACGGTGCGCGCACCCACTGGCCTGCGCCCACTGTCTGGCACTCCCTAGTGAGATGAAC-CCGGTACCTCAGATGGAAATGCAGAAATCACC

CGTCTTCTGCGTCGCTCACACTGGGAGCTGTAG-ACCGGAGCTGTTCCTATTCCGAGTCCACATATATTTTAACCTCAGGCCACCTCTCTGTATATACAA

GTCAATGACTAGTTTGAGAAAACTGCTCAAACTCTATCCAT---TG-AGAGGATTTCCCCTCACTTCTCCCTTTCAAGGCCATCCCTGAGTTGATCTGTA

GAGCAATTGTCATCGTTTTTTGTTTGTGTCCACATGCTGAGCTGAACCATCTTTGAACCATGCTCAGCCTTTTTCTTCCTCCATCACCTTGTCATAAGTG

ACTCCCAGGCAGCCATG--GGTGTGAAGTGAGTGAGTGGCATG-CAGTATGGATGACATGGGGGTCTGAGCCATCTGCTCATATGGTTTACTGTGCAATG

CCCTTTGGCACTGCTGTGCCCAATCCCAATGCACTGCCTCAATCT----TAC----CTCTGG-GCCCACCTGGCATTATGATTTGGTAGGTTTGACTGAA

CCCAGATCATGGCGAGCAAATTCTGCAAAATTCTGGCTACGAAAATCATTTGATTTCCCATGGCCAGGCTCTCTGTCTCTGCAAGAGTTCAGTAGCACAC

CGGAAACTTTTTTTTCAAAAGGTACAAGTTCTCCTCTTCAGACAGCCTGACTTTTCTCCAAAACCCCAGGTTCTTCTACTAAAGCGTAGATTTCACAGGG

CCCAGTGGGT-TTCTTTTCCCATCCACG-TTAACTCTAATACCATAAGGTCTGCCTGGTTT-ATGATccaagccacaggatctcttgtgccacagcCTGG

ACCCATTGCAGAGCCCATTCCTACTCAGCATCCcAGTCACTACTGGTAACCTTCTGTGTATGTGAGTCAGAGTAgccctgcctcctaagtagagaaaatc

tacctactgggcactgtgcttctttttaaatgggtggaggcatgagatgcaattattagtcttttacttttgaggaagtgtccctgtatgcccctagact

cattgtatggggtattctggagctgaaatgacccttcagaattgaacaggcccaatttaggagacatggaactggagtttctatacctacatcaaccaat

tattgcacgtggactacacccagaaatgggacgtgaCCTTGAGGAAGGCAGCTCTCTGCAGCCCAGAGCAACTCACagagaggcactcagcagacactta

ttgatcattgatgctgcagaagccaagaaaatgactaccttggtccaggcaggTAGGGGGGGCTCTAGGTAAAGCAACATAGCAactactatgtgatgca

gggcctgggcctcatgcatgatctacggctcctggctttgctgTGTAGCTACCTTGTAAGATAACTTTGGGAAACTTCTGTTTtctcactgggcaTTAGT

TTACTCAACCATAAAACAGGTTCTGAAACTAGATAtatggcttctaattggctgtgtATTAAAATCACCAGGGACTTGTCTAAAACTACAGATTCTAaat

tatcttccccaa-AGATTCTAGTTCAATGTATTAGAGTAGGGATGGGGCTAgcGGATCCTGTTGTACCTCCTGGATTTGCTCCAGCTCTGGtgtgccccc

aaatcacttaatttccctggtaaatagggataaagacatc--TGCCACTGGAGGATTATGTGAAATAATAGGCAAATAGCACTGTAAACAAAGGCATGTG

GGTGCATGCTCAGCATAGTTAAAGAGTGTGTTCACGGGCCT-----TGTGAGTAAGATACTTGGCTTCTCTAAGCCTCAGTTCTCTTC--TTCATGATAA

GCACCATAAAAGC---ACTTACTTCAGAG-TCAATC-AGAAGGATTAAATTAGATAACACACATAAATTTGGTAGTTCCTGGcctggagaacaATTCAAT

ACAAGTAGCCATCATTATTTATTGAGTGTCTATT-GC---ATGTCAGCTGCATCACTA-CATG-CTATGCAAATAAGCTTA-TGGAATGTTTCTTCCAGG

AACTTCCAAGCAACCCTGGGAAAGgctagtagcaaataccagctggttctgttttGCGGAAGGGATAAATGAG-GCCCAGGATGAGAGAGTACACTGTAG

TAAAAGACTCGTGGGATATATCC-AGGACCTGAGCACTGAGACACCTGGGCCTATTAACAGGGATATTAATCCTGAATGACTTTAAGGATTTTGTTTCTG

TTCTCAGGTCTCAGTTTT---TCTGAGAAAGGACAGGGTGGAAAACAA-CAGTTCTCAGGTCCTATTTAATTCTTCCTCTAATTGTGTAATCTCAGAGGG

AATCTGAGAca-----A-AGAACAAAGCTGGGAGTCCATGTTTTCAGGTTTACTTCCATCAAATGGTTCTGCATTCCAGGAAGCCA----GGAGATCT-G

T-TTTCA---CCATA-AGTG-ACTCATTTCCAGCCCCCATCAGgagttatttctgaccaCAGGTTTCTCATTCCCCAGCAGCAGAAAATGTGAATTGAAt

c-ACTTTAGATGGCTACTGT----------CAAG---GAGTCAC-CA-AAAATGAGAAACAGAAAGAAGCTGGACAGCCACCCACTCTCTGCAGAAGACA

GCTGAGCTGGGCAGAACAGCTG---AATCCAGATGGTCTGAAATGCTGGCTTAACATTCTCACATTCAACTTCTGCAGGTAGCATCAAGTTGAAGTC-TG

GGAAGGTAATCTGCAAAATGAAAATAGCAAACAAACTGCCTGTTGTCACTGAAGTCC-TGCCTATTTATGACACTGATATCACTCAGAAGCTGCTGTtcc

tggaccacattagCCACATGCCTCTGAAGCCTGGAGTACTCCCGACGCTTGAtCCTTGGCAAGATATCTGGGACCTCTTCCCTTTCTCTGGCAttcttca

tcatttcttttccacaacacaaggcattccttGGAAAACACAGCCCGCTGCAACATGTTTCAATTTGGAAACATGAGCTTCTGTTTGGGAGAAATCATGA

GAATGTTATTGTCTTACACTTTGAGTCCCTTCCTAGCATTTCCTAGCCATACTTCTTCTGAAGTTCC-TAAAAGTAACAACTG-TTAAGCTTACTGAGAA

CTTA-ACTATGTGCTTGTCACTGTTTAAAGCACTTTTCATGTGTCTATTAATTTAACCGTTATAACACTATAATCCCACTGTTTCCAGAAGTATAAACTG

AGATACAGCTTC--TTATTTTTCATCCTTTCCCACATCCAGTGATTGATAAGCATAGGGAATCAGTCTAATAGATACAAGTGGGCATTTTGCCTGGCCTA

GGGACAAGTACAACC-TAAGGCTTTACCTAGAGGAAGTTCAGTTGAAGGTTATAAGCTGTTGACTTAACCTTGATCCAACATCCAAAA-GAGAATGCTGG

TCCCCTCTGGGCAAGGCT----------------------------------------------------------------------------------

----------------------------------------------------------------------------------------------------

----------------------------------------------------------------------------------------------------

----------------------------------------------------------------------------------------CCTGTGCCTTCT

TCTATGCCCATATGTCATTCGCAGGTTTtcaatgaagactgctggtctgtagagggccTGAGGAAAACCTCCTCTTTTGATTAGAAAAGAAAatataggc

ctggcctGGCAGGTTTACTGTCAAATAAATGAAATGTACAActcaggcagtagaaagggtgttgtctaatgaaatctgagcagtctagtagggctacatg

gaggagggaggagggaagtcaagttagcaataatttctagaATTTGGAATAGTGGAGGAAAAAAaagacaagaattcaaggagttaatggtgagtatctt

catgagcaagatggaggcagggatgagtgtgcagtgcAGGGGTAACTAGACAAATATCCATGACACGCTTCCAAGCTggcatttgatgcagctatgctac

tgaatttctcaac---------------CCTGCACCAACCGTCCACTGGAGCAAGtagaagctatgtcagt-----------------------------

TTGTCTACTAACATTGCATCAGATACCTTGCCTTGATatgcatttttgggagagagagctttaatggatcagtccatcccacactttgttgttccctgga

gctgggtgtgagggacagggGCCTCCAGCTCCTGCATCACACAGACCACTCCACTGGCCctggtgatatattggaATTCCCCTTTGATTTTACTTGGGGA

AAGACTTTGACAGCTaaagcaaggtttgAAAACCACAGCACTCCAAGATCTTCATGTGctacccagcatcacatatcctctgataccaagataaagctat

aagatagcttta----------------------------------------------------------------------------------------

----------------------------------------------------------------------------------------------------

----------------------------------------------------------------------------------------------------

----------------------------------------------------------------------------------------------------

----------------------------------------------------------------------------------------------------

---------------------------------------------------TATCTGTAGGATTCCTGGAGTCCCTCATTCAGCCAAagttctaattaat

accaccctgggttgtgaagagtggtattacttagaaccctgctagcaaggtttaaaaatatacaggttttcctaatctCTGGTTGGAATCCCAGCCACGG

TTTAAgtccatgatgaggatgctgacagagttgaggatagtgcaaattttgtatctactcacctctctgatgaccacaatttactttcttcctctctttt

ctacagaaaacatttaagtgcaaaggAAGTGCAGCCCATGAGATGATCCAACTTGGAGCATTTCCtctttttaggctgggcataactgcacctccctttc

ctatcccatccccattagtctcagcaATGGATGGAAAATGTGCCCAGTCCCAGCCCACTGCTTCCCttttccacttcttggaaacctccccaggacccaa

tgcccactctgcagattttggatctcatcTCATAATTCTTTGACAATAATTCTGCCAAGGAATTCTCTctcttctccctaattctttacatcctataaaa

taacatcatgatttagtttaAAAAATTAAGTAAAGCAGCCAAAGGAGCAGAGAGGCCtagaaaatcccagaaacctccaactcttgccctctaacatgaa

ggtctccccagctcttctgtgctgactg--ccaccatgagttcccaggtgtttgctgaaccag-gtaaggcctctctctcctttcaggaaagttaaCCTT

CAGGACAATCTGATCAATAAGGGCCCTCAGAATGtcactgcaataagggtcttcagaatttaggggaaattttttttccctaaaaaatgggaatatgttt

caagagaaaccag-attggtacaagctggaactggaattctaggccaccgaagccagtctctg-ggctcttcccaggacacca-gtttggggaacaggtc

tactctattgtatagctctgagattttcaagagaacattttgtttataaaattaggcagttggtccggcatagtggctcacacctgtaatccaccatttt

aggagcccaaggcgggtggattacttgagcccaggagtttgagaccagcctgggcaacatggcacagccctctctctataaaaaacacaaaaagtagcca

ggtatggtgacacacacctgtattcccagctactcaggaagctgaggtgggaggattgcttgagccaggagattgaggctgcagtgtgctgtgaacatgc

cactgcattccagcctggggaaaaaagtgagaccttctaccaagaccagttcagtcatggaggccccaacccagcggtgctagaggaattgaagataaag

acacagaaatagagtgcaaagtgggatcagggggctaacagtcttcagagctgagagccacgaacagagtttgatctacatatttattgacagtaagcca

gtgatacacattgtttgtataggttatagattagctaaaagcattccttaggggaaacaaagaattcttagcaaggagtagagaaataggctctggctga

ttatccgcagcaaaaacatgttaaggcgcaggccgctcatgctattgcttgtggtttgagcagtttcccgctccagatgggccaggcattccttg-ccct

gctccagtaaaccaacaacttctag--cagtgtgcatcatagccatcatgagcatgtcacatttttgcagaaatcctgtttatggccagtttctttaagg

cctgtttatgacaggcttaggtctggttgccagcaaccttctctaataaagtataaataacaaaataaaattgggtagttatattttctttCTGAAAAAG

ATGAAGCTGGAAGAATGAAGCTGGGGGTGAGGGCATGGCTTTAGGAAGTGGAGGTCATGATCCCATCGAATGG-CTTTTGACTTGGCTGGTCTCCATGCA

GGTTGAAGACAGACaagactgccctggcactgtgattctagtccctaattccaacctcctgggaccaaggactgtggtcacttgttCAGCGTCAGCCCAC

AGCCATTTCCTTCCTCTCCTGCCCACcacaccttctttcctaccaggggagcttTCCCACCCAATCTATAGCAATAGCCTCTGAAATGTTCTCCctggct

gcagtcagtcccattcaacctatcatgaactccagaggaacaccaatggtgaattgaaacatcagaggaagagcaggtggcaatagattcagtggtcagg

ttacacttctCAGTGAAGAAGAGGCATAAGCAGGACCTCTCTTAAATGAtggg-----------------------------------------------

----------------------------------------------------------------------------------------------------

----------------------------------------------------------------------------------------------------

----------------------------------------------------------------------------------------------------

-------------GTGAGATCAAAGGAGAggttcgactgaactaatttagggtggacagaag----AGTAGGCAAGAAGCAGAACTCTTGTCTGGCTGGA

ATTTGGtccagaaataggaaaaacattttaaaaacttagaatgcaagctTGGAGCTACATTTTGGAGGGTCTTGAATGTCAACATGGACagtcgggttca

gTTCTGAATGTCATAGGAAGACAATGAAGGTGTGTGAAttagtggggaagagacgcaaactaatcagaagtatgcatgatgaagacagagcttctggctg

cctgaggatggaatgtGGAAAGGAGGACCTGAGAGCAGGTATCCGCAATAATCCAGgcaagaagggatggtagaGGTGGAGGAGAGGCAGCAGCTCATAG

GAAGAATGGGgtcagctggagcttactgaagatatttcgtcaagaggacacagtgggaggcctagggacccagctcctagaactcactaccaaaatatca

caaggcagtattgagagacatgcctaccctgAGCCCCAAGTTCACGTCCCTCTCTCACTAGCCCTGTAaGCCCCAACCGCAGAGCTTTTAAAACATGGAT

TCAAAAAGAcagggcctcctcttcctggatgacccctaagattcaactgcagtctatgagtttggccagctcctttttccaaacggggatccatttgtat

gaaaattagctataattttacctcctcaccaacagtaaagaccaatagcagggccaatacctgccagtcttggggagagaatgatagattctgtagaaag

cagagcactcagaagcatggagcactagaaaatctcaccctgtgaagcaggagacattgatgctgattgcagGAGTGGCACTGACTCCCTGAGCATTGTG

GAATATCAAATTctcCTTCTGGGCCTCAGTTTTCCTCTCCATTAAATGGGGATAAtgCTGTGTTACAGGCAGCAAGTGAAGGAATCTGGCAGATGAtctc

acatgttttcctagccccaggaactatgtcttggcaaacatcaccacatgcactggctagagaagagttggaaaggaaagtatttcaaatctgtcttcat

gtaccagccaggacctggattactgtgaacccactcctttctcaggcattgcctgcttagacacagatttttcattaaggccccccatccccacccccaa

attcaacaaaagtcctgggagcaattagcaaccagacaacttcattcgtcctcatgccagatcaagtagttctgtatacttcccctgccaccaagatctc

atctccaaataccaatcaggagtgaactctaaaactctaaccaagtgtagaagcacacagtgagttggaataaaaaaaaaaactaaaatatcaccatatg

cattgatactCAGAAGACCCTCTGGCACTCAGCAAGGCTTGGGCAGGAGgcggtggaaaatggtaaatgctgtatg------------------------

-------------------------------TCTCTATGTTGAAGAAGTTCTCAGGCTGTTTTTCCTCCAatTCCCATAGCATGAACCTGTAAACTCAAA

GAATTGGAGAtgttggggcctctaatagagcagtttccagtagggaccagcaaccagacaggaatgcattgcacatctttgagcattgcatcctcagctc

tttctttaggctgtgtgtcaggtaaatctgtgcaaatcattttttcttccatgaaacccagcttaccttttgcactagactgatggtttccaaactgagt

atgaatcagaatcaccaggactacccctgaaaatgcagatttcctg----------------GCCTTATCCCAAGATTCTGAGTCAGGACATTGGGTTGT

GGaacagtgcccccatcaggatttcaaccagttttcctggggatacagaggtatagctcagtatagcactggaacaccaatgctcTCTGAGGTTCCTTTG

TTCTCTCACATTTGAGTTCGGAccccacggctttgAACTTGCATGGCAGTCTTTGGAATGACCTGCCCAAGTCCTgtgtggcctctcacttcttctCCAG

TCCTGGATTGCCATAAACCAGCTGTGcgacctgctgtgacttcatctctccctcttagtgtctctttccattacttctatgacttttgtgatCTCTGAAT

AAATCTGAGATGAAGATGAAGAATGTAGCCAttctccagaacttgcgatttgcagaacATTCTTCTACCTGACCCTGCTATGTTCCTTGGGAATCACTt-

--AAGACAGAGAACAGCTTCCattgcaggagatttgttt-CTTTTCCAATGCTAATTGATCCCTGTCttctactagcttATGGTCTAGACCATGGTCTCA

CCATTCACCACCTCCTAAAttatgagCACGTTTTTGCATCCGCTTTAGATAATGGCTAACTAGGAAggagttagagaaaaggaggagagagagaaagggc

agaatggccacctccccagggaatgcattctggctgctggaacaaacaactgcaggaatTGGGCAGTCTTAAGTTTCATTTTAGTTTTTCACGCTAAGgt

ttttgaaatccacattaaaaccaaaaccaaaggaagggaaattgcaaaacacagggactttccaactgcaggctctgaaccatctgcagaacatgagcat

ggtgtctctgggtatgtgtcatacctgggatctttctcctattctTTCTGGGGTCCAGAGCATCCTGCACCTCTGGCTGATGTGAagaTCCTGGGGTCTC

TTGCTTCCCTGTTTCCGGGAGACTCTttttatttccttatttacagcacaggcattcctgaaggtcCATCTCTATCACATGTCTTCATCTGAATGCATGA

AGacaattaagggagtccttcttcaatagtccacat-CCTGGGTCTCTGTTTAGCATCCTCTGATCACTATATTCTCactatcaatatctataacaatga

---GTTTATTAAATACTTACCATATGCTAACACCTCACTTTTCAATGCCCTTTTCCTGTGTCAACTAGTTTAATTCACACCTATTTTGCTGATAGGAAAA

CAGTGGCACAGAGACCTGAAATAAGCTTTCAAAACATCTAAGCATCCAAATCCTGGCAATCTGGCTACAGAGTCCATTGTTTTAACCACTATCCTGCCTG

TCCTACAGCTAAACAGCAAAGAACACTTCTCTGCTGATTCCTAATGCCCTAAGTGTCCAAGTCTCAGATTGGACTCATTTCAAGATGATAATCAATCATA

GGAAAATAAAATTGAGAGGAACACAGATAATCTCCTAGCCCA-CGAATGGCCAACATGTCCCAGAGCT-GGGCTCCTCCCTGATCCTGTACTGATGGAGA

ATATCAGTAAAGAATCACATCACTATT-t-ctcatggccgac-ata-aggc-ctcatt--atct-tttc-----caataa---------------acctc

-tcgagttgacacttgcattgaaa--acctatttgacatccttga---tc----ttgtat----------atccc---cac--att---t-----gata-

-ca-cgaagc-agg--tggacc----taaa---aata-tcta------aat--aggt-tcc---atgg---------------ctact---a--------

ag--------cagg---ca-a----------gctgggat-------------------------taggcact-tg--gcg---tacacgc-cct---gca

---ttcta----c----t--aac-cca-ctttgc---t--ttctcata-attg---acttttaccacta--ttttc-----atttcttagctctgaa--t

gca-cct--ttgt-------------gt----------caagccacat-c---ac--tggag--aatgaaa---a-tt-----ttctgagt--tta----

-----------tttt---tgt-gtg--ac--acttgccatg------cat-gtggac-tgggg-----atttat---------ata-aactcaat-tta-

------t--------aaagaaaa----ct---tga--c---------tctgta--t---cagtg---ttatatga----ggcaattggcagggacagtcc

cagatgcttttgtgtaagagtg-------------------------------------TAAGTTCCCTCTTGTGAGAGGAAATCCTGAG-GTTAGGGAT

ATGTTTCAAGTTCTGGAACTTCAGTATTGTTAAAAGATGATC--CAG-AGTTTTTTAAAAAA---GAAATTAGATAAACAAGAGGTAGAAGATTTAAGGT

CTCTTATAAATAAATTTAAAAAAATAATTGAACT--CCTGAGCTAAGAAGACTACCAGATAATTCACCAGATAAAAACATAAACCATTGAACATGTACAC

AAAAATACCAACCAGTAGACAC---AA---AT-TTA-AACAAGCTC-AT-ACTGGAATAGTTTCTTTCTGCTAACTT-------TTAAATTGAGGCAT--

-TGG-----------------------TT-TCC--TA---CAAT----AAA-GTTCA--A-AG--AAG-TTAA--A----TCA---CTT----TTACATA

CATA-TGCCC-CA--GGAA---TCAC-CAT-----C--CA--AA-TCAAT-ATTTGCAGAACATTTCTAA--AC---------CCCAAAGTCTC--CCTC

A-CAT----------TC----CCT---GCCAG---TCAA---TA--CCCCA--CAACATCTAGTCTCAATGCT--ACCACT-A-T-A--------GGT-T

AGT-TTTGCCTATA-TA-----CAA--AA--TAA-TACTTTTTAT-TA-ACA-AAGTA-------GC--ATA--TACTTTC--AGTTAATAAT--GT--A

TTACTTTT-----CTAT---TTCTGC-A-TTAATAAATTACCA-------CAAAATT-------AGTGGTTTAAACA-----ATATAA-----ATGTATT

ATGTTATAATT--CTGGAGT-TCAGAAGCTGAA----CA-T--AGGCCTCAGC-AG---GC-TAA-AACCA----AGG-TATTATCAGGAA--TGTGTTT

CTG-T--C----TAGAAGTTCTAGG---GAAG-GATCCA-TTTT--TT-CGATTG-C-TCTGGG---C--ATTGGCA-----GAATTCA-GTTC------

-CTTTGGGT-TAT-TG-GT-T-TAT-TGA--GCCTAAATGT-TC--------------------------------------------attttcctgatg

gctgtcagacaagagattttcccagcttctagacgcctctgtattctgtggcccagagcccccttctccattttcaaagtcagcaacagtgggtcaagtc

cctttcatactatgcatttcttcttcttccatctcctctctctgttgagactcagaaaagagtctccactttcaaggactcaaggtattagattgagcat

acctggataatctctccatctcaaggtctgtaccctggatcccatccacaaagatccttagtgtgaaaagtgaaaatgaaacttaggactgcccaaatcc

tgtagctgtttggtccagcaacctgaactcattccccagagagatggccattctgccttttcgctcactccttttctctaactccttcccagttagccat

tatctaaaggagatatgataaaaaactcaccccctcatccctggtaacagagtcacaggttccagggatcagggagtgaacttttaggggttacttattt

attgagacagagtctcactctgtcgtccaggcaggagtgcagtggtgcgccctcagcaacctccatctcctgggttcaagcaattcccctgcctcagcct

cccaagtagctgggattataggcatgtgccaccatggccggctagtttttgtatttttagtagaggcagggtttcaccatgttgccaggctggtctcgaa

ctcctgacctcaagtgatccacccgcctcgacctcccaaagtgctgggattacagatgtgagccaccgtgcctagccaaaggggtcctttattcgcctac

tacaactaataaatgttgtcctgagtaaaaactttatggaaagcgatatggcaatatctactaaagcttcacatttcaacagcagtaaagacatggggga

gaatttttaaacttgggttttcaaaataatgttaattaaaattatttttaaatgaaaatataaattatatatgcctaatcataaagaaatgctttaatta

tgtatgacatttttataatgaagcagtatgaagccactaaattcaaccaattgaagaatatttaatgacttgagagagtgcatgactcattaagtgaaaa

aagctgtaaatatgatcataagtacattatgtccatatatgtgtgtatttatatacatgtgtatatgtatatgtttgtatatttatatatacatacctat

gtacatatatatgcacacatatgtatatacacacagatatacacacagagtgagatggagagagaagaaagggagaaaatacataaatagagcagccaga

tccaatcttagtgtttaaagcacagatagagtgaccaactgtcctgatttgcccagaactgagaggtttcctgggacactggactttcattgctaccgag

aaagtccaggctgaagcagaatgaactgattaccctaaatgcagatacatttttgtcctttcctttagggaacaatatattttatgttttctaaaagaaa

aatgcaaccatttaaaatttattacaagttcttcaggtacctcattctgtgttgggggcaatgccagacacatttcagaagctgttcttagcaacagcac

acatcctggaaatgcttcagataagtgatggaagtgtagtatcctccaccaatgaaatagaaacctcttgacttttgaaggtagattcttgaagaaacag

tatttctgcagttcgcagcagccacatgtcagttaaaaaagactttatctcctttagttatgtatgattagaccaatctcagaaatttgaatgggaaaag

caatgaaatttaaaaccacaaagctggagttgggcacagtagtgagtgcctgtaatcccagcactttgggaggctgaggcaggaggatcacttgagccca

ggagttcgagaccagtcagggcaacatagcgagactccgtctctaaaattaattaattaattaaaaccacacagctaaaattgggctacttggaagcttc

aagttgcaatttcttcttgaagatccctcttgtgggagtgactctggcatggactctgcaggcaaatgttggaggcacatgtgggagcaggcaccacact

gcagccgtccatcgctacatgccaatgttggcaggtgccttgagagagatgttttacgttttttttttaactagttcatgtagtctaaaacacactaatg

tttattatactgacctaaagactctgcagttgacttcatattgtactttcttatctttaacaacatcgaatagaaatgaactgctgattttcacacaaag

aaaatgaaacactatttggctcagaatgctattatggagtaaaatacatattaatcatattaataaatcaagtagactacttggtttcagtttttaaaaa

acagtgatacaaacaaaaccgactattttgtatgtgatgtggaacatttatttgtgcaatgactccttcacagtgcctaacagatattaacctagaggct

gttagccttgatgAGCTTAATTGTGATCTTAATAAATAATGAAGTAAAAataccaccctgagtaccaattaacaccttgtcattgagggctttatAATAA

TTTCCCAATTTATTGAGTTGATGTGAATAACttgtggaatcattaacactattct----CTGACATTTAGATAAAAGTCAATTGTGGGTTTggtttttat

gtcgacaaaattaatatgt-------TTTATTTAGAATGCTGTGGAGTGCACTAAATATTATGAAatatttTAGACCCATCACCCTGCCTCAAGTTAAAG

TAAAagacatttttgataaatgagttggactatttaaatgtgaagtatgagaaattgcttagccaagaacgtcat------------------AAGGCTC

AAAGGTTACTAAGCTGACATGGATAACATGGAAcATCTGGTAGTGATTAGAGACGTCTGATATTTCCAGTTCTTCCTCTTCCGTGCACAGGGCAGGGTTG

TACTTCCAGGCCATCTTGAAAACCGACAACCTGTGTGATCCACTTTGGGCAATAAAATGtAAGCAGAAGTGGCATATGTCCTTCCAGGCAGAtacattta

agagctaggacaagagtctccatgtctctttcacctgccagtgaccaggagtggtccagtgataaagactccagcagcccaaataaggagaaagtagact

ggtgagaaaactcttgaaatctggataagaggcagcccacattatgctgtgtcaaacc---------------GTACCACACTGTTCCAGGCATTACAGT

CAAAACCAGGAGCcctgagaAGAGCTTGGCCTGGACAACCAGACCCCTGGGTGCTCTGTTtcacctctagcatatcaattccctaatctcatcctcagct

gctccatcaataaagttgagatgataatctgtatATTTAGGAAATGAGGGGATTCAACAGACAAATTCCATTTtcacccctagtccttaaatgtgtgtct

tg-GACCACATCACCATGGCCACCAGCAAAGACAGAGCGGgggaagacagtaaccagaatctacactaccaagcaagccagtgacccagcattatgcaat

atttgcccaaataaccagcagggcaggccaggagacttctacgcaCATCCCGATAGCACCCCGAAAGCCCCAGGGGCAACTGGACagttcactgtcatca

agacaatttcttagttctcataataaaacagttctgcccacctctcctccttacacctagccacatacccaaacctcattcaagagtttatcctaaAATT

CCAATTGATTCCAGGGAGTTACAAAGGACAAGAATAgacatccctgtacagtttattggggttcagaagttcCCCTGTGCCAAGCAAATATTAGGCATTG

AGTGGGGACTGgtgatacaaaagcttgaaatagtaaattccgtgttcccaagtctgaatggaatctgttcatgctcctttgttgagacatgttaagtaac

cctccttgaaattattcatctgttttccagctgggcccaggATTGCTTATCCTTGCAACATGAACTTACAGAGACAAAAaaataaAGATTCTGGAGCCTC

CATTACAATGAAGACTCTACCAGGTaccagggaccaaacgagatagtccactgtgtaacagggcatggtgaccccgtcgctgaattctggttttgatTCT

TTACTAAATTGTCAAAGTGACTTTGGGGAAATCATTTtcccactctgtgcCTCAGTTTATACATCTGTAAATGAGGCATTTGAACTAGattaatgactcc

caaaatggctTTGGTTAAAAATTACCTGGTCACCTAGACCACTTCagaaatagagaattcggggtttcatctctgaagattctgactctgtattttggtg

taggttaaggcatctcttctgCATTATTAATAATTGCCCCAGGCATCTGGATATTCCAGGaggcaggtctgaaaagaccagtgcactggagagtctctga

ggtttctagacacctcactgtaggttttctgcatggttcttgCCCTCTAATTGGAATTCCATGATGGCTTCTGTTCTGAcattcccacttgccagtgcta

gcttgtcatgaagtagccgcacaacctacaataaatcatttcacttctgttgttgtttcatttgtaaagggaaatacttgaataaattaaatgcaactgC

CTCTTTCAACTCATTCTATAATATTGTGAtcattaaggacagttggagCAGAGCTGAAAAATGAAAGCAGAAATTCTGCTTCCCAGgatgCTCTCCCACC

TAATTTCACTCCTTGAGAACCAGGTAAGAatctagaaggtctggttccatagcaaggtatatatttcctgccctTCATTC---ACAGACCGTTGTTTCTG

ATCTCAGGCTCTGCTCCATTAGCCACTGAGCCACAGACATATTTGTTCTATATGTCCTATTTCACATAGTTGTGAAGTCCACCTTAGGAAACTGCTCATG

AGGAAGCCACCGAAGAGAAAAGAAACAGGAAAGGCAGAAGGACCACTtctctgaacaacacagccaGGCCACTGGACAGAACAGCTGCTGAATTTGCATG

CCCTAagtttcg-TTTTAACTTTTTACATAAGGATTTCTCAAATTCAGTTTAAaaacaa--GAGAGGGAAATTGCGTAATGAAAGCGGCTTGCCAACTGA

GATTCCTAAACTCCCTGCAGAATTAGAATTCTGATGCCCCTTGGCATCTGTTAGCACTGG--G--ATCTGACTTCCATTCCCTCTGA-GTTCCCAGAGCC

ATCTAGTATTTTGGTTCATGGCAGGATCCCAGGATCTCACTttgctcccgttcctgggagacacttttttttccttcatccacagcacaaatattcccca

aggtCCATCCCTATAACACATCTATATCCACTTATCTTCTGGACactacacccaccttacttaactattgattACAGTTAGTTTATTGATCACCTACCAT

ATGCTACTCACTCTTCAAAGCAAGTTTTGGGTATCAGATAATATTCATAAAATGCATAAAAATAAATATCAATTTCCGCACAAGAAAATTAAAGAATAGG

TAGGAAATTGGAATATAAAGAAGTTAAATAACCTAAGCCTTCTCTGACAAATATATTAACCAGTGTTCCCAGTATCCAAATGAAGACTTTTAA-CCATCA

TGCTATTCTGCCTGTCTATTTTAGGACTGACAGCCAGGAACACTTCTCTGACATTTCCTAGCATCTGAGCCTCTCTGCTCTTTGATT-T-GATTATTTGC

AGACAATCACAGGTGAATCAAGTTAGAGAAGAGCTTAAGTACTATTTAATTCTGGAATAGGTTACA-TAGATGGTACCA--CTCCTTATTACTGTACTAA

TAGCAGACATCA-CT--AATCAAT--CACAGCATTATATTCACGGT---AA-TGCCT--CATTGTTT-TAGTTTTA-CAACA--AAACTTTCTAGTCATC

-TATCACCAAT---TCATAAAGTTG--GC--ACATGATCAAAAACTACTTGTCATCCCAAAACCTGTCAAAACCC-CATACTTCATAGATGAGGATA---

-GTGA--ATC--TCAAAGT-CA---ATA---AGACTTGCCCAAGGCC--ACTTTGGGCAGGTT--ATTGGTGAAATCAAGACTAGACTCAAGTtccttgA

C-ACTTAATTCCCTTCA-TT--TTTACTAGCCTT-TTTTGATTCCCAATAATTATAATTTTCATGACTATTTCTGTTGTCTG--TGCA--TGGATGCACT

TTTCGTTTGAA-GTCAGCTCAGaggaaaattaacaaagtcctcagggtattacataagacaatggctttattgctgcttggaaaatacacacaaactcca

cccctaaaccaataccccc------------ACCCCATGCACAC---ACTC-AGATTGA-T--GCTACA-C-A--GTA--CACCTGGCAGAG-AACCATC

-CCCTAAAA-TGATTAAAAAGCTGCCATCCTGAAACAACTCAATTTCCTTTCTGATTAA-AGG-AAATC--CAAATGACACACAT-G-TTCCATATACTG

CAAATTCTTTACTGTTAAAAGTTCACA-TAGTAATTGTAATAAATGAAAAATGCA-TGA--GGTAAATG-TTTAAAAATCTCTTCTGAATCAAAACAGTA

AAAACCCACCTGATTTAAG-AACAGGAGCAT-ATTAACCACAAGAAA----AAAATAAAGCAACTAACATACATATTCAAAAGC-AAATAA-AGTAAAAT

GTAAGCAAAATAATGGTGAGCCTTTTGCTTCGATTTA-CTTTATTTCGAAGTATAAT-TCAATATACTGTCCAGTAAAGTGCAGGCATTTTAA--ATGTA

GAGTTTCATGAATTTTACA----TGTGTAGTTAA--CCATTTAACTACCTC-TCTGATCAAAATCAGAGAGG--GAAGCTTCCAA---AAGTTTCCCTCA

TGATGTCCATACCTGCAAAGGAACCAC-TGTTCTAACCTCTATCACTGTACGTTTCTTTTGTCTTTTAACAAGAAA--AATATCTTCTTCTTAGCAACTA

TAAAATGTTTTTA-AGAA--T---AATACACTCACATTT-AAGTGA-AGGGGCA--AATTTTCCAGCAATGTGTTCATTTGATTA-A-AAGTC-TTAATA

ATATCCACATATTTTGATTT-ATTAATTCTTTGG-C--T-AGAAAATTAAAC-GA-AGACA----TTAATA-ATAAT--GTTATATCAGCTTTTCCACAA

GTATTGTGTAA--AAC-CATAAAAATGGA--AATGGTATGA---------TTC-CC-AA-CT---ATGGAGAAGTAAATTCTTAAG---TTAA-TTATGG

TGCACTGATA-TATATGAACTCC-TATAGAGCT--TTTA-AAGTTCAAGATTATTGGGG-AATATTTACTGTCTTG--TGAGAAATCTAAACACACTA-A

TTGAAA-----AGGCAG--TATTCAATC-TTGGATT-TGGTGGTGAAATTTTAGATATACACCCTATAGTTCAACAATACATT-GCCAATTACTAACCGA

TGTTATTTCTATAAAA-C-AATGAGAATTCCTGAGGAACAAATTTTGTAATTGCCCCCTCTCCTCCCTTCCTAATTCATTCTGAGTCCAGAATGACCCTA

ACATCAAAACCAGATA--AAGACATTACAACAAAAGAAAACTATAAACCA-ACA--GAAATCTA-TAAACCAAAC---T--CATGA---AC-AA---AAT

TCTCAAC---AAAATATCAGCAAATAAAATCCGA----CAA-CA----TATAAAAAGAATTATAAGCTAC-AACCAAGTGGA---ATTTA-TTCCAGGTA

T-GCAAGGCTGATCCAGCGTTCAA-AAATTGGTTGAT--ATAACCCATCA---TATCAA-CTTTCTAAAGAAGGAAAATCTTAT-TATAATATCAATAGA

----TGGAAAATTTTTTTTGACACAATTCAACAGCCATTCATGATTTAAAAAAAAAAACTCTCAGCAAACTAGGAATAGAGGGATACTTCCTCAGCTTTA

TAGAGTGCATT-AAAAAAGACCTACAGCAAATATCATACTTAATGGTGAGAAACTAGATATTTTTCTCCTAAGATTGAGTATAAGGCAAGGATGTTTCTT

CTCACCACTCCAATTCAACATAATACTGGAAGGCCTAGCCAACACAACAGCACAAGACAAGAAAATAAAAATATATACAGATTAGAAAGAATAAATAAAA

CTGAATTTATTTGCAGAAGACATGATTGTCTGTGTAGAAAATTACAGAGAATCAGCAACAAAAAAAAGTCATGCCACTAATAAGCAATTTTAGCTACAGG

ATACAAGGCTAATAGATAAAATTCAATAACTTCCTGTATATCAGCAATGAGCAACTAGAATTTGAAATTAATACACAATACTATTTACATTAACACCAAT

AAAATGAAGTGTTTAGAAATTAGTCTAAGATGTACAATATATATGAGGGAAACTACAAAC-TTCTGATGAAAGATTAAA-GACGATCTAAATAAATGAAG

AGATATTCCACATGTATGGATAGGTGGAGTTAATGTTGTCAGCATGTCAGTTCTTCCCAACTTGATCTGTACCTTCAACACAATCCCAGTCCAAATCCCA

GCAGGTTCTTTTTGGATGTGATAGACTGATTTTAATGTTTATATGGAAAGGTTAAAGATCTAGAATAGCTAACACAGTACTGAAAAAGAATAGTCAGAGG

ACTAACATTACCCAACTTCAAGATTTACTATTAGGAAACAGTAATCAAGACGGTGTGGTATTGGTGAAAGAATAGACAAATGGATCAATGGAACAGAATA

GAGAGCCCTCAACAGATGAATGGATACATAAAATGTACTATATACATACAAGGGAATATTACTCAGCCTTAAGGATATGTAGCATATCATATGCTGCCAC

ATAGATGAACCCTGAGATCCTTACGCTAAATGACATAAGCCAGTCACAAAAGAAAAATACTATATGATTTCACTCACATGAGGTAGTTAGAGCAGTCAAA

ATCAGAAACAGTAAGTAGAAATGGTGGTTGCCAGGGGCTTGGGGGAGGGGAAACTGGAGATCAATAGGTATAGAATTTCTGTTTACAGATGAAAAGAGTT

ATTGAGACTGTTGCACAACAGTGTGAATGTTCTTAATGCCACTAAACTATGCACTTAAAAATGGTTAAGATGGTATGTGCATTTTTACCACAATAAAAAT

AATAAATGAATAAAGAACAAAGGAAGTAATTAGCTATCAAACCACAACAAGGCACGGAGAAACCTTAAATGCATATTGCTTAGTGAAAGAAGCCAGTCTG

AAAAGGCTGCCTACTATATGATTCCAACTATGCATATGATACTTGGGAAAAGACAAGCTCACAGAGATAGTAAAAAGATCAGTGGTAGCCAGAGATTTGT

GGAGAGGAAAAAGGATGAATAGAAGAATACAGTGAATTCTTAGAGCAGGAAAACTTCTATATGATATTGTAATGATGGATAGATCAGGCCTGCAGGGGGA

GTAGATGAGGTCAGTCTTTTGCAAACCTGGTTCCTTCTtgccacgccccgcccacagccccactgtgtgtgaaccaagaGAGTTGGAAGGCCTTCTGCAG

ACGGACCCATTCCACCTGaatctccatgacatattggacttCTCCATATAATTTCATTTGGAAAGAAACTTGAGCATCTAaataaaaTGTTTGGAAACCA

CCATGCTACAAGACCAGCATGTGCTGTCTAGTTCTTACCCTCTGCAACAGTAAGATTCTGGGGCATTAAGACTTA-G-TTCCAGGATTCTGTCATTCTGC

CAACGTTCTGTGG------CTGGGGTTCTAAAGGAGCTTGCCTGGCTTAGAactgcaagtgactctAGTGTGATGGAGAGCACCAGCAAAGCCTTAGGGC

CCATCcctggcCTCCTGTTACCCACAGAGGGGTAGGCCCTTGGCTCTCt--TCCACTATGACGTCAGCTTCCATTCTTCCTTTCTTATAGAcaATTTTCC

ATTTCAAGGAAATCAGAGCCCTTAATAgttcagtgaggtcactt---------TGCTGAGCACAATCCCATACCCTTCAGCCTCTGCTCCACAGAGCCTA

AGCAAAAGATAGAAACT-CACAACTTCCTTGTTTTGTTATCTGGAAATTATCCCAGGATCTGGTGCTTACTCAGCATATTCAAGGAAGGTCTTACTTCAT

TCTTCCTTGATTGTGACCATGCCCAGGCTCTCTGCTCCCTATAAAAGGCAGGCAGAGCCACC-GAGGAGCAGAGAGGTTGAGAACAACCCAGAAACCTTC

Acctctcatgctgaagctcacacccttgccctccaag--ATGaaggtttctgCAGCGCTTCTGTGCCTGCTGCTCATGGCAGCCACTTTCAGCCCTCAGG

GACTTGCTCAGCCAG-GTaagacctctccctttttaag--------------------------gggagaccaaaagaggaattaagaagagccattatg

tcacagctcattaggaacaaaaccagaactaaaggc--------------------------tcag-gtca------------------CTGAGGCTGGT

TCCCTTGATCTTTCCTGACCCCAGTTTTGgg-AGG-AGAC--AGTGGAGCCGCTACAGCAACAACCCTCCCATTGTTTGGGGAAATAATCCAGAACGAAG

AACTGTTTCTcactGTGGGTGTAAAGGACATTTCAGGCCGTAGTGGAGAGGGAGaaactattgcctgaagcttcaaatttt-GGTTATGGTTCAGTGTAC

CTTCCAGAACAGTGGCTGTGTAaagaggatgagga-CCCAGAGGAATCTCAGCGTATGGCATAGGCTAACT-CTAAAGCCCATGAGGATGAAAGACTGGG

AAGCAAGGTAT-TGGAACTTATGTTCCCAGTGTCAGAAGTTTTGGGTTAGTAGACAAGGACTAGCTTGTTACTCAAAATGTTTCC--AAACCCAGTCAAC

AATGACGGGCCGCAGAGTTCAATAGAGGAAAGAGACTCACAGGCAACATTTTATCTCTGGGATCTGGACTAAGACACTGAACTTGGGATGGTGACTTCTT

GGTCTTCTCCTTCCTTCT-CTTCTTTTCCTTACAAATGCACACTTACGGTGGGTCCTAAATGTCTCATTCTTTGCAAAATTTCTTTCAG---ATTCAGTT

TCCATTCCAATCACCTGCTGCTTTAACGTGATCAATAGGAAAATTCCTATCCAGAGGCTGGAGAGCTACACAAGAATCACCAACATCCAATGTCCCAAG-

--GAAGCTGTGAT-gTGAGTGGACagtgcc------------------TG---GCACC--CCCA----TTCAAAAGTTCTGA-TGGACAACATAGAGAAG

T---CAAGATTCATGTCCATATGAGTCGGATGCATATAACTTCTATCCAAAGGGCCCCTCTACCCCATAGAGAAACTCAGTCCATGAGAAGGAGTCCATA

ACTGCTCTAGGATTCCCTTCTAGGGGCTTGGTGAaactaacccaatatctgtAGCCAGGACCCTGGAGGGTTTCACCTGGACAGCAAGAGCAgagcttcc

ttctggagcttcttcctcccactcttcccctccctcctctcccgggtccgggtcctTCACCTAAGGACCAAGGGCTGATCAGTTCTAGGgaccaatggcc

cacagtcctgtgcaggatcttcaaagtcttccatctaattgtgccctctctcccccaCAG-CTTCAAGACCAAACGGGGCAAGGAGGTCTGTGCTGACCC

CAAGGAGAGATGGGTCAGGGATTCCATGAAGCATCTGGACCAAATATTTCAAAATCTGAAGCCATGA---gccttcataca-----TGGACTGAGAGTCA

GAGCTTGAAGAAAAGCTTATTTATTTtccccaacctcccccaggtgcagtgtgacattattttattataacatccacaaagagattatttttaaata-AT

TTAAAGCATAATATTTCTTAAAAAGTATTTAATTATATttaagttgttgatgttttaactctatctgtcatacatcctagtgaatgtaaaatgcaaaatc

ctggtgatgtgttttttgtttttgttttc-CTGTGAGCTCAACTAAGTTCACGGCAAAATGTCATTGTTctccctcctacctgtctgtagtgttgtgggg

tcctcccatggatcatcaagg--TGAAACACTTTGGTATTCTTTGGCAATCAGTGCTCCTgtaagtcaaatgtgtgctttgtactgctgttgttgaaaTT

GATGTTACTGTATATAACTATGGAATTTTGAAAAAAAAtttcaaaaagaaaaaaatatATATAATTTAAAACTACTTAGTCTTATTCTTCTTGGGGTAac

atttagctgggagtgagttttgggcatcatgggtgacagtttgggcatggacgggccatttttcaagaatgtcttctggctacgctggactcaaccaagg

ttctcagagaacttggtgggaccaggccaggatgttccagctctctgactctagtccctaacttcagcagccctgattcgctagcctctcttgtttctct

tgtttatatattatccagcctaaggtattttgttatagctgcccaaaaagactaagataatctccatcactctacccccaaccccaatcccaagaacttg

caagcatccatttaaaggcgtggaacctcttctttttga-------------------------------------------------------------

----------------------------------------------------------------------------------------------------

----------------------------------------------------------------------------------------------------

----------------------------------------------------------------------------------------------------

----------------------------------------------------------------------------------------------------

----------------------------------------------------------------------------------------------------

----------------------------------------------------------------------------------------------------

----------------------------------------------------------------------------------------------------

----------------------------------------------------------------------------------------------------

----------------------------------------------------------------------------------------------------

----------------------------------------------------------------------------------------------------

----------------------------------------------------------------------------------------------------

----------------------------------------------------------------------------------------------------

----------------------------------------------------------------------------------------------------

----------------------------------------------------------------------------------------------------

----------------------------------------------------------------------------------------------------

----------------------------------------------------------------------------------------------------

----------------------------------------------------------------------------------------------------

----------------------------------------------------------------------------------------------------

----------------------------------------------------------------------------------------------------

----------------------------------------------------------------------------------------------------

----------------------------------------------------------------------------------------------------

----------------------------------------------------------------------------------------------------

----------------------------------------------------------------------------------------------------

----------------------------------------------------------------------------------------------------

----------------------------------------------------------------------------------------------------

----------------------------------------------------------------------------------------------------

----------------------------------------------------------------------------------------------------

----------------------------------------------------------------------------------------------------

-------------------------------------------------CAGCCTTTTAAGGTCAAGATTCCCCTGTACTTAGTGAGCTtagctgaatct

tcttacaaacatgtgacccgccatattgagccatacataccagagcttattatttttccagcttattgggaaaacacgtctaaggcaaacaaatttattg

tactgttgaaccaactctgcagggcctactgttaatgttcatgtaaaggtgccacaattccagtatatttggaagtatagagaggaaagagatttgggtt

ctgctcctaagagtaaccttaactctcttggtcacttagggtgacataatttttcccttcagcattaagatgccccatccaaaaatcaggatcatactgt

gtcacaggcaggaagtgagaggatctagcagatgactacacaagtccctccagctctagagccatgtctcttagaaacaaggtcatgtttgttgtctggg

gtagagtggggacataaagttcctgggATTAAAGCCATAAAGGCAGCCAGGGCCTAGCTTATTGGAAaaacacatctaagtcagataccccttatttgga

gacaagctttccattaaagttcaaagtagtacacccacagttccaagagcaataaagcagctaagtgtcagatggactagaggggcacctcccaggcagg

gtccacaaaaaagctccatcaaactctaagctctttcttcaatagcttcaaggtcaattgggcacagatatgtgggtcctgggcttcatgaaatcctgGA

CCACAAATCCCAAATTCTAAAGGCAAGAACACTCACacaaaactgacaccaggccacagcaaaaagacacaaatttgttcttctcaaattTTACCCAGCT

TCACTTTGGGATTATGTCATTATATTTCAAatgacaatgagcataatacaccttaagtaacatgtcatcttatttaagtggttgatgttttatgtgccct

gacttgaatatatgtgttttaaaAATCCACATCCTTCCATAAACTGGTTCAGATCTGTAAGCcctgctttgctgtcttgtaaactgggggaaagacctgg

aaaggtcactcaaagctgacatacaacctcaggctcacctcagtttcaccctcagctaaggctgaccatttgtggcagtccatgactcacttgattcact

caatatctgctttctgcctcttctcacttctcacttgctgcccccaggcttctcctgggtgtcatcttgggtcaggagttgactccactgcacacttgtc

caagtttcagaagtgtggaagagtgaacattttgggtggtaaccgtccacagtgaagggtgaaggcagatgttcaaatgcttctgccatcccttttacca

gttctgctctgatcttggttatttcttttcttctgctggggttggatttggtttgttcttgtttctctagttccttaaggtgtgacattagattgtctat

taatgctctttcagactttttcttttaaatataggcatttaatgctatgagctttcctcttagtaccgtttttgctgtatcccaaaggttttgataggtt

gtgtcattatcattgttccgttcaaagatttaaaaaattttcatcttgatttcattgttgacccaacaatcattcaggaatacattatttaatttccata

tatttgcatggttttgaggtttccttttggagttgatttccaattttattccactttggtctgagagagtacttgatataatttcaattttcttaaattt

gttgagacttgttttgtggcctatcataatgtctatcttggagaatgttccatgtgctgatgaatagaatgtatactctgcagttgttgggtagaatggt

ctgtaaatatcccttaagtccatttgctctagggtatagtttaagtccattgtttctttgttgactttctgtcttgatgacctgtctagtgctgccattg

gagtattgaagtcccccactattattgtgttgctatctcatttcttaggtctagtagtaattgttttatagatttgggagctccagtgttaggtgcatat

atatttaggactgatattttcctgttggactagtccttttatcattatataatgtctctttttgtcttttttaattgctgttccttcaaaatttgttttg

tctgatataaaaatagctactcctgctcatttttggtgtccatttgtatggaatatcttcttccacccctctatcttaagctcatgtgagtcctaatgtg

tcaggtgagtcccctgaaaacagcagatacttggtgaattcttatccattctgccattctgtatttttgaagtggagcatttaggctatttacattcaat

gttagtattgagatgtgaggtactactctattcataataccatttgttgcctgaatacctttttttcattgtgttattgttttctaagtcctgtgagatt

tatgctttcaggagattctagtttggtgtattttgtggatttgtttcaagatttagagctccttttagcagttcttgtagtgccggctcggcggtggcaa

attctctctgcatttgtttgtctggaaaagactatcttgccttcatttatgaagcttagttttgctggatacaaaattcttggctgataattgttttgtt

taagggggctaaatatagaaccccaattccttctagcttgtagggttcctgctaagaaatctgctgttaatctgataggttttcttttataggttacctg

gtacttttgcctcacagctcttaagagtctttccttcctcttgactttagataatctgatgactacttgccaaggtgatggtctttttacaatgaatttc

ccaggtgttctttgagcttcttttatttggatgtctgaatctctagcaagtctgaggaagttttcctcaaatatgttttctaaacatatttggggaacat

atttctaaacattccctcaaatatgttttctaaacttttagatttctcttcttccttgggaacaccaataattcttagatttggtcgtttaacataatct

caagcttcttagaggctatattcattttttaaattcttttttcttcacctttgttggattggtttaattcaaaagccttgtcttcaagctctgaagttct

ttcttctgcttgtttgattctactgctgagactttccagcacattttgcatttctctaactgtgtccttcatttccagaagttgtgattgtgatttattt

atgctatctatttcactggagatttttccattcatatgctgtatctttttttttatttctttaagtaggacttcacctttctctggtgcctccttgatta

gcttaataattgaccttatgaattctttttctggcaattcagagatttcatcttggtttggatccattgctggtgagctagtgtgatcttttgggggtgt

tgaagagccttgttttgtcatattaccagaactgtttttctggttccttctcatttgggtaggctgtgtcagagggaagatacaggactcaaggactgct

gttcagattattttgtcccacggagtgttcccttgatttggtgctctttcctctccccaaggatgaggcttcctgacagccaaactgtagtgcttgttat

ttctcttctgtatctagccacccagcagagctactgggctctgggctggtactggagggtgtctgcaaagagtcctgtgatgtgatctgtcttcaggtct

ctcagccatggataccagtacctgctccagtggaggtagcatgggagtgaagtggactctgtgagtgttcttggttgtatttttgttaagtgcctaattc

attgttttatacagttaatatttctttcCCTTCTCTTTGGTCAATACCTGGATCTCAAaaggcaagctcactagagtCAGGGCCAGGAACTCAGTTGGGT

ACTTGGTTGGTGTGTTctCCAGGGTTGGGGATAAGACTAGGACTGGGGACTTACCCAGcatgatctttggagccaggactggggctgacattgcagactc

agtcgacgtgcttggtaggtttgggttcaagaggacagcccacaaatcaggagcaACAGGGCTGGGGCCAAGTTAGCCACATAACTAGGagtatgaagac

acaaaaatggattgagcaaataatggaaatggaaatgtatcaaagataatggaagtcaagtcactcaatgttgaagaagcttctataaaaaatacagaaa

gttggagggaaaaaaaataaagcaaaacaacaacaacaacaaaaaactctgggatgttggcctggaattggaggtgaactcactgttcattaacttatta

gaggtaaactcatggttttaaatatagataggtagatggaaggaaggaaagaagaaaggaaggaaggaagaaaggaaatggtgattagtgggaacacata

aacataggtatactagTTATGTTTACTGAGAAAGCCTAGAAAGAATGACACATGgggtggtaatgtgcgcttcagtGCCCAGTTTATAAATACTATCCTC

CACTATAGAAACAAGAgctcctgggataagtGGCTGACTCCAGAATTAGAATATTCTATCGTGTCAAAgagaaagggattactcaaagaaagatggagac

gtgtcaaaaacatttaaaaaccaatctgggctgggtacagtggctcatggccaggcttggtggctcatgtctgtaatcccagcaacttagaaggccaagg

cgggaggatcacttgaggccaggggttcaagaccagcctggccaacatggtgaaaccccatctgtactaaaaaactacaaaaagtagatgggtatggtgg

tgcatgcatgtagtcccagctattgaggaggctaaggcaggagtatcgcttgaacccgggaggcagaggctatagtgagccgagatcgtgccacttcact

ccagcctgggtgacagagtaagactccgactcaaaaaaaaaaaaaaaaaagagccaacctgaagagtctcaCACTGGCCAATTTGGGACAATTTGATAAC

CAAAATAAATAcattttttattacaa--AATAATTTATTTTAGCCCATTGAATAAAATCAAAGAAGatAAAGTTTTATCGTAGAATAAAATGCTAACCAA

tgcatatagatggaatggtgaagttagaaaatcaccaacttctaaCCATCAGAGTAAAAATTGATTCAGGCAAGAATCATCTATGgATACTAAAACTCAT

GGGTGAAAATTTAACTAttgac---------ATAGCCTCAAAGTATCTCCCCACAacataattaataacatgaattaataagtacaaaacacttattaat

taacatatataattaagagaagagaagagacttgaCAACATTTTAACCAAGTAATAAAAGCTAACATCACCAGTAatggggcaaatcagcctgaatatct

cctgataatggtgcactgagaaggacacagcattacttcaatTACTTTCCTGCTAAAAATACATCAACTGAATCTAATCaggagaaaatatcaggcaaat

caaattgagggagattctgctaagtaactgatcttactgtcaaggtcacaaaatataaggaAAGACTGAGCAACTATTTCATATTGATGGAAACTAAAGa

gacatgacagctaaatgcaacatgatcttggattggaccatgaacctgtaaaggatatgattaggacaatgaaaaaagccttaatgaaatatttggatca

gatggtgacattaaactgatgttaatttcctgatttcatatttttaatgccgttgttctgtagcagagtatctttgtttttaggaaatatactgaactat

taaggtataattaggCATTCTGTCTACAGTCTAATCTCAAACGGTTCAAgacacaaaacactccctttaaagatagagagatagatcattctatatcaca

tatttatgtgtgtgtatgtttgtaagtgtgtgtgcgtgtgtgtgggggggggcgtgtaacaccagatgctccacatctggtattgcaacacttggtattg

ttgttcttttaaaaaagctggccgtgcagccataaaaaaggatgagttcatgtcctttgcagagacatggatgaagctggaaactatcattctcagcaaa

ctaacacaggaacagaaaaccaaactctgcatgttctcactcataagtgggagctgaacaatgagtacacatggacacagcgaggggaacatcacacacc

agggcctgttggtgggtggtgggctaggggagggatagcattaggagaaatacctaatgtagatgacgggttgatgggtgcagcaaaccaccatggcaca

tgtatacctatgtaacaaacctgcatgttctgcacatgtatcctagaacttaaggtatatttttaaaaaagctagccattattattatgtgcatggttcc

atctcatacggtttcaaattgcatttcactcatgaataatgaggttgagcacctgttcaaatgtttggtggctatcctgttttataaagttcaaggccat

gtcttttgtcatttttatttgggat---------------------------------------------------------------------------

----------------------------------------------------------------------------------------------------

----------------------------------------------------------------------------------------------------

----------------------------------------------------------------------------------------------------

----------------------------------------------------------------------------------------------------

------------------------------------------------------------TGTCTGTCTTTTCATTATGAATTAGAAGTATTTTCGACAt

tccagattggaaccctttgtcaggcataagaattgaaatatcttctcataatctgtgatttgactgtgtactcccttaaagatgctttttgattaaaaaA

AACAAGTTCACAATTTTAATATAGTCCATTTATCATgttccttcatggtttata---CTTTTGTTTTCTGTTTAAGAAAACTTTTTAAAacctaATGTCA

TTAAGGTATTCTCTTATGATATTTTCTAGAAacttcatttctttttctgTTCACATTTAGAGCTACAATCTACCTGGAATTAATTTTTGtgtatgctgaa

agataggaaagataggaatcaagattatattttttcctataaatatctgattcctataaatatctttactaactaatcaaagcacacgagttatttaaaa

gactatttttcctaacttctttAGAATGCAAACTTTGTTATAAATCTGATGTCCATATACGTatgattccacttctgtaccctctaatctgttcccttgg

tataTTTTCTATTCTTGGAGTAATGGCACATGTTTTAATGACTacaaCTTTATAATAAGTGTTAATATCTGGTAGTATAAGTTCTAcatctttgttcttt

atgaTTGTCTTGATGATTTTTGGCTTTGTATTTCTCCTTAtataaagtttagaata--------------------------------------------

----------------------------------------------------------------------------------------------------

----------------------------------------------------------------------------------------------------

----------------------------------------------------------------------------------------------------

-------------------------------------------------------------AGCTTATCAATTTCCATGAAATACTTGCTTGGATTTTAa

ttgagattacattgcatttgtgggggaattgacaacttgattattcagagtcttccaattcgtgtgcatgccatatccctctgcttattcaagtctcTTG

ATTTCTCTTAGTAATGCTTAGTAGTTTCCGAGGTATTgcacgtgtcttgtcaga---TTTATTCTTAGGTATTAAGGTTTTTATAAACTATTGGAAAtcg

tgtcacttatgaaatgtcatctcttaatgttagttacatgtaaaagtaaaatcaatttttgtattattaatgagcaattgtgctaaattcactcaattat

ttatagcttatgtgtagactcagatgttttttatgaaacccatgtttcatgccaaaatatgaca------------------------------------

----------------------------------------------------------------------------------------------------

------------------------------------------------------------------------GCTTTATTCCATCCTTTACAATCATGAT

AATTTCATTTCAgattcttatatctcatttactaggacctccagtgcaatgttgaataaaagtggacagctcttgtctcagtCCTGCTCTCAAAGGGAAG

TTTTCAATATTCTACtatgaagcGTTATGCTTGCTGGAGGTATTTTGTATATGCTGTTTATcaaaTTAAGGAAGTTTCCTGTATTTGTTAGTTTGCTTAG

GGTTTtttttttcttttagctttgaaatggctttgaaactcaataaatgattTTTTAATTCAAATGATCATATGATTTTTcta---CTTCATTTTATAAT

TGTGATAAAATACATTCATTGGTTTTtccaattttaaaacaagctaatATTTTAAAAATCAAACcaacctagttacaatgtatcattttttcaggtatta

ctggattccatttgctattatttaattcaggactgttacatctatgctcatgaaagatactgccctataatttttctttcttgaaag-------------

----------------------------------------------------------------------------------------------------

-----------------------------------------TCCTTCTCAGGGTTTGGCCTCAACATTATAATGATTTTAtcaaataaactggactgtgt

ttcttgtttaattattgtctgaatatatttttataagaaTTCATGTTTTTGCTTCCTTTAGTACTTAGAAGTAAAGCATtctggaatttacctgtc-AAA

AGTTTTTAATTACAGATTCCACTAATTACTAGAattgaaatattaaaatgttctatctttcttggttctctttaataaattgtgttttt--CTCACATTT

TATCCATTTCATAAAATTTTAAAATATATctatctgaagttgttcactt----TTTTTATTATTTTGTAACATCTGTAGTCTCTG---TAATAATGTCTT

CCTTTTGATCTCTGATGTTGCTAAATTGgaacttttcactTTTGATTCCTTGGTCAGTCTTGCAAAGGGATTAACATTTTattaaTCTTTTCAAAGAGCA

AACTTTTTGCATTGGCATTTTTcttcattgcatgtttattttatgtTTTCATTAATTTCTGCTTTTATtttttttgttccattttttggatttgattttc

taccttttttctaattcttgagaaaaatacctagatgact-----GATTTTAAGTTTTTCTTTTTAATACATGCATTTAATTctatgaatctctgtcttt

ttcattgggtaaaaccactttattaactgatcaaagtacttcattttgtttcctgattcgaggttcaaccattaaacacagttcacaagaaaatacaatg

attacttaactaactgcaattacaagagttagaaatctccctagttgttcacataatacttgtacaaattcacacaatcgtgtggaccacttaagcttag

tgtgaaccaaaagagcaaaccatagtcatggctagagttttgtaataacagaagagtaattgttaccttatgaatatactttaaaaatcacttggctagt

aattgtactgtttcctcattgctctggggtgtgtatgaaggctcttaggagagcaaacatctatttctgttctgtatgcctctctctcatttcaaactgt

gtaaaataactattgccccaccatgaacattggggattggaaagatagtcctacaatcttcttcataggttttggcttttaggcaagccggctggttttc

tccaaagctttcttttgaatcttcagatactattttaatcctaaatgtagattactgtgtttgtgagggtatctaagtgaccatgtgatggcaaggacaa

caaagtagtccaggaacacagtaagtgagtgtgtgtgtgtttgtgtgtgtgtgtgtttccaactgagcccggctttgagatttcattttgttagttgaca

gctctttaatcaataccaaagactctggaacactgcagatttgctatggaggtagataaaacaaatcaggcagttaagtcaattgagaaaaaaagggatt

ttccatctttagataatatcacgactaacagttgattctttattcttggtgcacatttaacatttctacctattttgggaaatattcaagtcttcTTCTT

TTTTTTTTTTTTTattgttcattcttgggtgtttctcgcagagggggatttggcagggtcataggacactagtggagggaaggtcagcagataagggtct

tcttacctgtctctcagaacaaacacatttaaattcagcttgtattaactacagataaaagaaaaaaacagtattaacatttgtatgacagcactgccgt

tcactttctggatttgtgacacacaaacatcatgcattctaagacagaagttaattttatcagtgtcactagtgttgttaattttaaaagatacagcaag

ttcaaagcttttccaaaaattgaaatcaccatgttttaagacagactggaatgttacaaatgattctgtaaaataatcatttttctgtacattctcatct

tttggttcttccttatctgaatccacagtgggtacctcccatccttcagcagtttatccaggtgagaatacttgaatgtgaactgagaggcccagtcact

cagggtctcctcctgggcaggagggaggtcagaaaggtcatcatgcttgtcctgcagtgctcccttatccaggcaaaatgtggcagggcccctggatgta

tctctttcggcaaagatcctgtgtggccccctggcccttggaacttgcaacctctggtcacgctgaacactttgcccttgctggtgtggccatgagaatg

tgcaggtcctaaaccccattgaagtgctgcagctcggcaagggagaagttgtacctgttgccaccacatctttggcagcaaagtttaggccaggctaggc

agggctctggagctccctgctttctcctccctctcaatgagtgagttgccgctatgaatctcttttttttttttgagacggagtttcactctgttgccca

ggctggagtgcaattgtgcgatctcagctcactgcaacctccacttcctggcttcaagcgattctcctgcctcagcctcccgagtagctgggattacagg

cggccaccaccacgcccggctatttttgtattgttagtagaaacagggtttcaccacgttggccaggctggtctcgaactcctgacctcaggtgatccac

ccgccttggcctctcaaagtgctgggattacaagcgtgagtcactgcaaccggtcgccactatgaatctcttaaacacagcttttaaagctcacacattt

taatatgacacattttcatcataatttacctcAAAATATATTCTCATTTCCATTGTGATTTCTTCTTTGATCtatgagttatttagacaaaattgactaa

agttttaagctttgagatattataaaaataaatCATTTTATTGTTGGTGTTAATTTAATTCcataATGGTTAAAGAACACATTCTATgtgttcaaacctt

ttctatttttgagattagctttgtagtccaacatagagAGTCAGTTTGATAAATGTTCCacgtgtatttgaaaaaatgtatatgctgaagttgttggtta

cagacatgtcaactaagtaaattttgcttgtagtgtttttctactct-----------------------------------------------------

---------------------------------------------------------------------AATGACATTTTCAGAAAAAAAAATGAAAGCt

gaatgaatttgttactagaataACAATATTTCACTATGAGAAAATCTGAAGG-------GAAATGATTCCAGTGGAAACACAGATCTTCAGGAAGTAAac

aacagcactgtaaatgatcAGGAGCAAGACAAGAAGCATCTCCTATTCCTTTTcaacattgttctcaacgtcctaggcagtgcaataaggtaagataaaa

agaaacagagacataaggattagaaagaaagaacttaaaatgtcatatacaggtaacatgattatatacataggaaatacaaggagatctacaaaaacta

ctagaatgaagtgaatttctcaagatcacaggaattaagctaatatacaaaaatcacttgaatttctatatactaataagaaattagaaattaaagtttt

aaaaatccatctattgtatcatcaaaaatagaataagaataagaataaatttaacaaaagatgtgtaagacctctatactgaaaactaccacacatactc

aagggaaaatgaagaaggacctaaataaatgaagagctatataccacatttatggagcagaaggctcgataatgttaagctgtcaatttaggatggagct

ggaggccattatctttagcaagctaacacagagacagaaaaccaaatactgcatgttctcacttataagtggaagctaaatgatgagaagacatggtcac

atgcagggaaacaacacacactggggcctttcagagggaggatctgaaagatcctacccacactgggcggagggagaggatcaggaaaaataactaatgg

gtactaggcttaatgcctgtgtgacaaaataatctgtacaacaaactcccatgacagaagtttacctatgtaacaaacctgcacttgtatccctgaactt

aaaagtttaaaaaaagtcaatataatcccaattaagtccccatggtcttttttttcttaattttttccaactgttattttaggttcaaggggtacatgtg

tagatttgttacatgggtaaattgcatgtcgtggatgtctggtgtacagattattttgtcacccgagtaatgaacatattaatcagtcgttaattcttca

aatttcaccttcctcctacccttgacccccaaatagtccctggtgtctattgttcccttctttgtgttcatgtgtactcaatgttttcctcccacttata

agagagaacaagcagtctttagttttctgtttctgtaccaatttgcttaaaataatgacctccagctccatctatgttgcctcaaagaatacgattttat

accttttaatggctgtatagtactccgtgatgtatatgtaccaaattttctttttaaaaaaattttatttaactttaagttctgggatacatgtgtggaa

tgtgcaggtttgttacataggtttacacgtgccatggtggtttgctatgcctatcaacctgtcatctaggtttgaatccctgcatgcattaggtatttgt

cctaatgctccccctccccttgtcccccacccctgataggccctggtgtgagatatcccctccctgtgtccatgtgttctcattgttcaactcccactta

tgagtgagaacattcagtgtttggttttctgtttctgtgttagtttgctaagaatgatggcttccaccttcatccatgtccctgcaaaagacatgaactc

attctttttttatggctgcatagtactccatggtgtatatgtgccacaatttctttatccagtctatcattgatgggcatttggcttgcttccaaatctt

ggccatttcaaatagtgctccaataagcatacatgtgcatgtgtcttaacagtagaatgatttataatcctttgggtatatacacagtaatggaattgct

ggatcaaatggtatttctggttctaaatccttgaggaattgccacactgtcttccacaatggtggaactaatttacactcccaccaacagtgtaaaagtg

ttcctatttctccacatcctttccagcatctgttgcttcctgactttttaataatcacccttctaactggtgtgagatggtatctcattgtggttttgat

ttgcatttctctaatgaccagaggtgatgagctttttttttttatgtgtttgttgactgcataaatgtcttcttttgaaaagcatctgttcatatcctta

acccactttttgatgggttttttttttgtaaatttgtttaagttccttgtagattctggatattagacctttgtcagatgggtagattgcaaaatttttc

taccattctgtaggctgcctgttcacactgatgatagtttcttttgccgtgcagaagctctttagtttgattagatcccatgtgtcaattttggcctttg

ttgcaattgcttttggtgtattagtctttaagtctttgcctatgcctatatcatgaatggtattgcctaggttttcttctagggtttttatggtttgggg

ttttacatttaagtctttaagccatcttgagttaatttttgtataaggtataaggaagggatccagtttcagttttatgcatatggctagccagttttcc

cagcatcatttattaaacagggaatcctttctccactgcctgtttttgtcagatttgttgaagattagatggttgtagatgtgcagtgttatttctgaga

cctctgttctgttccattggtctatatatctgttttttgtaccggtaccatgctgttttggttactgtggccttgtagtgtagtttgaagtcaggtagca

tgatgcctccagctttgttctttttgcttaggattgtcttggctatatgggctcttttttgattccacatgaaatttaaagtagttttttctaattctgt

gaataaagtcaatggtaatttaatgggaatagcaaagaatctataaattactttgggcagtatggccattttcatgatattgattcttcctatcagtgag

catggaatgttttcccatttgtttgaatcctctcttacttccttgagcagtggtttgcagttcttgaaaaggtcctttatgtcccttgtaagttgtaacg

ttgtattgctaggtattttattctctttgtagcaattgtgagtgggaatccactcatgatttgactccctgcttgcctattgttggtatataggaatgct

tgtgatttttgcccattgattttatgtcctgagactgctgaagttgcttatcagcttaaggagtttttgagctgagataatggggttttctaagtataca

atcatgttatctgcaaacagagacaattttacttgctctctttctattttaatactttttatttctctctcttgcctaattgccctggccagaacttcca

ataagatgttgagtaggagtggtgagagagagaatgcttgtcttgtgctggttttcaaagggaatgcttccagcttttgcccattcagtatgacattggc

tatgggtttgtcataaatagctcttattattttgagatatgttctatcaacgcctagtttattgagagtttttaccatgaagggatattgaattttattg

aaggccttttctgcatctattcagataatcatgtggtttttgttactggttctgtttatgtgatggattacatttattgatttgcatatgttgaaccagc

cttgcatctcagggatgaagctgacttgatcgtggtggataagctttttgatgtgctgctggatttggtttgccggtattttatggaggattttcacgtt

gacattcatcagggatattgcccttaaattttcttcttttggtgtgtctttgacgcattttggtataaggatgatgctggcctcataaaatgagttaggg

aggaatccccctttttctattgtttggaatagtttcagaaggaatgttaccagctcctccttgtacatctggtagaatttggctgtgaatccatttgttc

ttgggctttttttggttggtaggctcttaattactgcctcaatttcagaacttgttattggtctattcaggcattcaacatcttcctggtttagtcttgg

gagggtgtatgtgtccaggaatttacacatttcttctagattttctttttttttaattacaatttatttttattttttatttttgagacagagtctccct

ttgttgcccaggctggagtgaagtggcatgatcttggctcactgcaacctccgacccccaggttcaagtgattctcctgcctcagcttcctgagcagctg

ggattattggcgcccactaccacacccggctaaatttttttgtattttcagtagagatagggtttcaccatgttggccatgttggtctcaaactcctgac

ctcaagggatccacccaccttggcctcccagagtgctaggattacaggcgtgagccaccataccttgtctcttctagattttctagtttatttgcataga

ggtgtttataatattctctgattatagtttctatttctgtgggatcagtgttgatatcccctttatcattttttcattatcacattttccttattttgtc

cactgttgataggcatctaggttaattccacatctttaatattgtaagaagtgctgtgatgaacatgtgtgcatgtgtttatggtagaatgatttgtatt

cctttgggtatatacccgttaatgggattgctgggtcaaattgtagttctgttttaagttctatgagaaatctgcaaactgctttccacagtggctggat

gagtttacattcccagcagcagtgcataagcattcttttcttcacaaccttgtcatcatctgatatttttttgactttttaataatagccattctcagtg

atgtgagatagtatctcactgtggttttaatttgcatttctctgatgattagtgatgttgaacattttttcatatgcttgttggccacatgtatgtcttc

ttttgaaagacatctattcatgttttttgcccattttaatgggttgttttatgctttttaatttaagttccctatagattctgcacattagacccttgtt

gaatgcctagtttgcaaatattttctcccagagaacaaagactgtctgtttactcagagatgatcatatggttttgttgataatttcttttgctatgcag

aagctctttggtttaattaggttcacttgtaaatttttgtttttgtggcaattactttcggagactttgtcataaaatctctgccagggcctatgttcag

aatgatatttcctaagttttcttctagggtttttataattttaggttttacattgaagtctttaattcatcttgagctgttttttgtgtatggtgaaagg

aaaaagtccagcatacaccttctgtatatggctagccagttatcgcagcaccatttattgaatagggagccctttccccattgcttgctattgtcgactt

cgttgaagatcagatggttgcaggtggacagttttatttctgggttctctaacctgttccattggtctatgtgtctgtttttatatcgttgccatgctgt

tttgttttgtttgttgtagccttgtagtataatttgaaggctggtaatgtgatgcctccagctttgttttttgtttgttttttgtttgtttgtttgctta

ggattgctttgattattcaagctctttttttttggttccatatgaattttataattctttttctaattttctgaaaaatatcatgtgtattttgatagga

ataccttttaatgtgtaaatttccttggggagtatggcattgtaacaatattgattcttcctatccgtgagcgtggaatgtttttccatttgtttgcatt

acttctgatttatttcagcagtattatgtaattcttgttgtagaaatcttcacctccctggttcgctgcattcctaggtattttattcttttttgtggct

gttgtgaatgtgattgcattctcaatttggctatcagcttgaacattattaatgtataaaaattgctactgatttctgtacacttatcttgtatcccaaa

actttgctgaactggttatcagatctaagaggctttgggcagagaatatgcagttttctaggtgtggaatcatatcgcctgtgagtagagatagtttgat

ttcctcttcttatttgaatactttctatttcttccttttgcctgatcgctcaggctaacacttccagtactatgttgactaggagtggtgagtgtgaaca

cccttgtcttattccagttctcaagggagatgcttcttgcttttgcctgttttgtttgatcttatctgtgggtttgtcaaagaaggctcttattattttg

aagtatgttcctttgatgactagtttgttaaggtttttaacatgaaggagtgttggattttattgaaagcctttcctgcatctattgagatgatcatgtg

gtttttgtttttagttatgtttatgtgatgaatcacatgtattgatttgcatatctttaaaccaacctttcatcccaggaataaagtcttcttgattata

gtgaattagctttttcatgtgctgctggattcaaattgctagtattttgttgaggatttttgaatttatgttcatcaggaatattggcgtgaggtttttg

ttgtttttttttgtgtctctgccaggttttggtatcagaatgatgctggcgggggagaaatcagttagggaagagtccctcctccttaatttttgggaat

agttttattaggattggtaccagctctttatacatttgatagaatttgactgtgaatccatctggtctgggttttttttggttggttggtttttttaact

gattccattttgaaactcattattggtctgttcagggattcagcttcttcctggttcagtcttggaaggttgtatgtttccaggaatttattcatttatt

ctaggtttcctagtttgtgtgcgtgaagtgttcataatagtctttgagggttttttttgtatttctgtggggtcaatgataatgtcctctttgtcatttc

tgattgtgtttatttagatcttccttctttttttctttattagtctagctattgtctgtcaatcttatttattctttcaaagaaccaccttttggttttg

tttatcttttgcatggtttctcatgtctccatttcattcagttcagctttgcttttggttatttcttttcttctgctagttttggggttggtttgctctt

gtttttctagttcttctagatgtgatgtcaagttgttaatttgagatctttctcacttttttatgtggttatttagccctataaacttccctcttaacac

tgccttagctgtgccccagagattctggtgcaatgtatctttgttttcattagattcaaataatttcttgatttcttccttaatttctggtttacacaaa

agtcattcagaccgagattatttaatttccatgtaattatatggttttaagagctcttcttggtattgatttctatttattgtactgtggtctgagagtg

tgattggtatgatttcgattttctttgatttgttgagaattgcttcatggttaagcatggtgtcgattttagagtatgtgccatgagcggatgagaagaa

tgtctattctattgttgttgggtattctatagatttctgttaggttcatttcatcaagtgtcaagtttaggtctcaaatatcataattagttttctgcct

cgattatgtctaacactgttagtggggttttgaagtctcccattattattatgtagccatctaagtgtctttgtaggtctctaagaacctgttttatgaa

ttttagtgctccagtgttgggggcacatatatttaggatagttaagtcttcttggtgaattgaaccctttatcattatgtgatgccattttttgcccttt

ttgatcattgttggtttaaagtctatttgtctgaaataagaatagcaaccccgcttttttattttctgttttcttgacaaatttttcgccatccatttaa

tttgatcctgagggtgtcactgaatgtgagagatgggtctcttgaacacagtatacagtgggtcttgcttctttatccaacttgccattctgtatctttt

aagtggggtgtttagcccatttacattcaaggttaatattgatatgtgcagatttgatcatgttatcaccttgttagctgattgctatgcagacttgact

gtagagttgctttataatgtcaatggtctatgtgctttgtgttttttgccacagtcttttttatagagaaattgatatgtatgtgaaaaggacctagaat

agccaaaacaatctttaaaacaataaaatagaaggatgggcactaccagatttcaagacttcctataaggtgataattattaggatagtataatataggc

ataaggatagacaaacagatcaatagagcaaagtagagagttcagaaatatacatatatatgtatgtatgtatctaactatatatagattaggtatctat

tgatagataaataaatggatctgtattagtcgtgactcttcagagaaatagaacatatacatacatacacacacatatgtatttatctatctatctatct

atatatatatagAGAGAGAGAGAGAGAGAGAGGTtgattgattgattgtaaggaattggctcacagaattatgaaggctgacatgtctcaggatctacag

aatgagtcaggaagatggagaccaggagaaccaatgaagtggctccagtcaaaacccaaagacctgagaaccatgagaattgatgggtagttccactgca

aaggccagctggctagacacctaggaagagctattgtttcagtttcagtccaaaaggatggaaaaagctgatgtcccagtccataggaagtcaggcaaaa

taattctctctaactcaagtgagggtcagcctttttgtttaattcaagccttcaactgattggacgaggcccac--------------------------

---------------------------------------------TCACATTAGAGGAAACAATCTACTTTAcaccgtctacctatttaaatgttaatct

catccaaaacactctca-----------------------------------------------------------------------------------

----------------------------------------------------------------------------------------------------

----------------------------------------------------------------------------------------------------

----------------------------------------------------------------------------------------------------

----------------------------------------------------------------------------------------------------

----------------------------------------------------------------------------------------------------

----------------------------------------------------------------------------------------------------

----------------------------------------------------------------------------------------------------

------------------------TAGAAATACCCAGAATATTGTTTGACCAAATATtagggcACTCCATGGCCCAGTCAAGCTGACACATAAAAGTAAC

CATaactataagtacacacccagacacattcacatattg------------ATTTCATTTTTAAGAAAGTCATCAATCCAATGAAGAAATA---------

----------------------------------------------------------------------------------------------------

----------------------------------------------------------------------------------------------------

----------------------------------------------------------------------------------------------------

-------------------------------------------------AAAAGTCTTCCAAATAAATTATGCTGGAGCAACCAGATATctgtatgaaaa

aaaaataagtttctcccttacctcacactatagacaaaaattAATTTAAGATGGATGATATACCTAAATACAAAAGCTaaagctAGAAAACACCTAGAAG

AAAGCGTAGGAGAGTATCCTTAcaacttgtggcagaaaacctgtcttttttaagcaaaacagaacacataaccttacacttttagaaacgtatatattag

acttt-------ATCAATATTGAAAATAATTACTCATCAGAAGATACTATgaagaatgtaaaaagataagttaaagattaggagaaaatatattgtatta

catagaaaatactttaattcagactggatagtaaattttaaaaattaataacagaaacagaaaacaacccaaataaaaataggaaaaaaaatgtgaacag

gaagtcctaaccagagcagtcaggcaacagaaagaaataaagggcatccaaactggaaaaacggaagccaaaccaattctgtttgccaatgatataatgg

tatatgtagaaaaccctaaagattccttcaaaatactcctagatttgataaatgaattcagtaaagtcttaggttacaaaatcaatctgcacatcagtag

cactgctatacaccaacaacaaccaagctgagaatcaaataaagaactgagtcccttttacaatagctgcaaaaataaagtaaaatatctaggaatatac

ttaactaaggaagtgaaagatctctatgaggagcactacaaaacactgctgaaataaatcatagatgactcaaaaaaacgaaaatacatctgatgctaat

ggattggaaaaatcaatactgtgaaaatgatcatactgcccaaagcaatatacagattcaatccaattcttatcaaaatactaacttcatttttcacagg

attagaagaaactattctaaaattcacatggaaccaaaaaagagcaaattctaagcaaaaggtacaaaccaagaggtattgcactgacttcaaattatac

tacagggccatagtaaccaaaacagcatggtactggtgtaaaagtagatactgagaccaatggaatagaatagagaacccagaaataaagacaaatactt

aaaaccaaatgatctttgaaaaagcatacaaaaacatgcattgaggaaaggacactctattcaataaatggtgctgggaaaatttgatagctacatgtag

aagaatgaaactgaatccctatttctcagcatatacaaaaatcaactcaagatggactaaagatttaaatctaagattgaaatcataaaaattctagaag

aaaagccaggaaaaacccttccggatattggcctatgcaaagaattcatggttgagaccacaaaagcaaatgtaacaaaaacaaaaataaaagaaatggg

acctaattgaactaaacagctgctgcatagcaaaagaaataattatcagagtaaacagacaacccacagaacgggagaaaatatttgcaaactctgcatt

caacaaagggctaatatccaggatcttcaaggaattcaaacaaatcagtagataatattaataataatcatcccagttaaaagtgagcaaatgtcatgaa

tagagagttctcaaaagaagatagataaacggccaagaaatatgaaaagaatgttcaacatcactaatcatcaggtaaatgcaagttaaaaccacaaaga

gataccaccttacctcagccagaatggccattattaaaaagttaaaaaacaataaatgttgacatggatgtggtaagaagggaacacttgcacactgcta

gtaggaatgtagattagtacaacctctatggaaaacagtatggtgatttctcaaagaactaaaagtagatctatcacttgatccagcaatcccactactg

agtatctacccaaaggaaaagaagtcattatatcaaaagatccttgcatgcatatgtttatcgcaaaacaattcacaattacaaagatatggaaccaacc

taagtggccagcaacctataaatggataaagaagatttgatatatatcaccatggaataccactcagctatataaaaaagaatgaaatatcttcttttgc

agcaatttggatggaaacggaagtaactcagaaatggaaaaccaaatgctatatgttctcactcataagtgggaactaagctatgggtatgcaaagacat

acagagtggtataatagacattggagattcagaagggggaagggtggaaggagggtgagggatagaaaagtacatattgggtaaaatgtacactaatggg

tgcactaaaatttcagacatcaccgccatacaattcatccatgtaatcaaaaaccacttgtacccctaaagctattgaggtttttttagaaaaaatgtga

acagtcgtttcacaaAAAATATTCAAATAACCAATAAAAATATAAAAAGGAGCtgaacatcacaagtcatcaagaaaaggcaattgaatacctaataaaa

cactactacaacccattaaaatggctacaatagggaagattgataataccatgtattgatggaaatatagagcaaaaggaaatctgatatgttgctggag

gaaagtttgcaaaactgtttagtgctttgtgataaaag-----------------TAAACATATGTTTACCCAATGACCCAGAAATCCCACTTctaagta

agtatttcaatcaatgtattcctatatgcatAAAAAGATTGGAACAACAGTGTTCATCACAACTTTATTCAtaacagcagaaaactagaagcatttcaaa

gatctaggagaatggataaagtgtggcgtattcatcaaatggaatattacctagcaataaacacaacaaaaaactgacatttttttaaaattacattgag

cagctggagtgcagtggcgtgatctcggctcactgcaacctctgcctcctgggttcaagtgattttcctgcctcagcctcccaagtagcagactacaggc

gcacaccatcacacccagcaattttggtatttttagtagagatggggtttcaccatgttggccaggctgctctcgaactcctggcctcaggtgatccgcc

tgcctcagcctcccaaagtgctgggattgaaggcatgagccaccgcgcctggcctgtaataacttcataaccatagtaaagttattttagagctctcttt

gaaggaaacacattttgattgattaaatatctgaaacttcagaaagcctacatttttcttccttaaccatgtgacattagtgtgagtggaaaccaagagt

ataaagctaagccctatgtactaaagaaataacataagttaacctattactaccacatcttacatttggatttaaatacgaaaaacacaacacagaaaac

agaaactttttttttttaattaagatcataaataggtcattgttgtcacaacacatttcagaattttaaaaaaacaaacattttggctttctaagaaaaa

agactttaaaaaaaaagtcaattccctcatcactgaaaggacttgtacatttttaaacttccagtctcctaaggcacagtatttaatcaggatgccaata

ttaccaccctgctgtagcaggaataagaggcaaggtattagcaccaagaaaaacagcaaaatttctggacaaaatcatctgtcattaaaaaacaagagag

agaaaaagatagactaagtggtaagcattttaatgaagaaggtccagtgttgttattcagtactttctgttaaaaaaaaaaaaaaaaagaaatcgaagta

ttttaactcaagtaggtttaattcaccacaaaagagctatgagaaaagagagggggttggtaaaatacagtatactttctttaaaaaggacttgggagga

aaaaaatcagcttagaagatcatatgaaagggcaaacatcgcatggaacccagttagtgtcctagtttattactagtcttcagacagatccagaaaatag

agtaagatactaggtgttatgtctacctagtatcttactctattttctggatgtctgaagactagtaataaactaggacgagttaatgggtgcagcaaac

caacatggcacatgtatacatatgtaacaaacctgcacgttgtgcacatgtaccctaaaacttaaagtataaaaaaaaatttcactgagcataagacttc

agacacaaaagagtgcatgCCATATAATTCCATTTATGTGAATTTCAAGAACAatcagtgatgacagaagtcaaagtagtggtcacctctggaaggtggg

acattgaccaataaatatttcttatgtgtatcatagcatcaagcatcacttattgtcccagtaaaatagactaaatgccaaatagcactgtatggtcata

gataagttctagttctcccatttaacggaacactattcaatcattaagaaattcag--------------------------------------------

----------------------------------------------------------------------------------------------------

----------------------------------------------------------------------------------------------------

----------------------------------------------------------------------------------------------------

----------------------------------------------------------------------------------------------------

----------------------------------------------------------------------------------------------------

----------------------------------------------------------------------------------------------------

----------------------------------------------------------------------------------------------------

----------------------------------------------------------------------------------------------------

----------------------------------------------------------------------------------------------------

----------------------------------------------------------------------------------------------------

----------------------------------------------------------------------------------------------------

----------------------------------------------------------------------------------------TTATGGACATAT

GTAAAATGACTTTTGAcatagcattaactaacaaacggagaccaacatttatccagtaaaattcctaatatgctcacatggaaagaaaaaaagaacaaca

gaaattttactgtgattctgtgtcaacattGAGTGTACATGTTGTTTTATAATATTTTTATgatttttgagatatacttttcaagagTTCTAAGTCATCG

TACATTATTTT---TAAAAACATAAAtgtgTTTCTACAAAACGCTGCTCCAACAGAAAAGCTTCtgcttctttgcttATTCTTAGTACCAGCATGTGCTG

AATACACGGTTGTTCCTgttggcattttccacttggtcaTGGGAAGGGGCAGCTCCAGGCAACCCCCCTCGGAGTTCCccag-AAGGAAAGGGCAGCAGA

AGCTAGAAAtgtaaacaactaaaatgatgatgcaacaactggtaagatgcgaagtccccactggagaggaagatggtacacctcaatt-----TGTCTGG

GACAACCCTGGTTTATTCCTGTCTGgcttgcaagataaagtatataatagttctcatacagaggagagcccttatggcttcttagacactcatatcataa

gtaagtatTCATTTTTGACTGTGTGCTTGTCCAGAGAATGCCCAGAAAagggactggattttctagagtagaagcagtaaagcttgccctgggaggtgac

caacaatttatttcagatttggcctcctttgacatctg--------------------------------------------------------------

----------------------------------------------------------------------------------------------------

----------------------------------------------------------------------------------------------------

-------------------------------------------------------------------------TGTAGTTTGAGAAGTCTTCATAAATGA

GACATGATTTATAccaggtctcaaatgattcctgtttgggaaggaattacgggttgatggaatattataagcaatgcatatagtgatggatataagaact

gcaggtggtatgaaggaagaagtaaggaagtggtcttgtaatgttttcatgggcaatgcatgggtttttctcccccagcataccaatggatgtctcctct

tctgcaggcttgcctttctggagagattcagccctggaggctgaacatattgatTAGTGCTGGCTTCCTCCCCTTTTCAGTAGCCACTCCAGCAgttaga

catgtttcctataaaatgaaaaatttgcaccatcgtggtgctcctcagagccttaacaagtgaaatgagataaacagggaaaagtaaaggcacaaggtgc

cCCAGCTCTGCTTCCCTGAGCAGTtttggttttaatctgttacgtcagaacttaccaataagttactccttgagaata----------------------

----------------------------------------------------------------------------------------------------

----------------------------------------------------------------------------------------------------

----------------------------------------------------------------------------------------------------

-------------------------------------GACTTTAGAAGGTAAACAAATATCTGAAAACCACAAcattacatacatttgccaacactatta

tgttactctctaagattgtgggcactaagattgcctagtactatagtgggca---AGATGGCAGGATGCTTGGGTTCTAAGATTCTACAGCAACAtaatg

attctaaaataacaGGATTCTATAGATCTTGGAGACTTCTGTtatataaagattattgGGTTGGGAAACAGCTGGCTGAACTTCTACTCTAGACATAAct

gcattACTAACATAGGACCTTCGAAAGTTCTCACTCTGATTGGTGAACCAGGGTAGATTTTCAGGTCTtcataggacatcaTCCATTCAGAACCATCAGC

AAATAATCCCAGAAGGTCTCtatttctttccccagtacagagtctccctgATTTATGGCAGCAGGTTCTTCTGGAAAAGGCATGGaagaagatttatagt

att-AGACCTATGAGTTGCCAGATTAAGCAAAAATATTGCAAGaggtatatctatgcttaaaaagaaaaatcactccctgtttatttgcaagtcacattt

aactaactgccctgtatttcatctggcaatccagcattgcagggcccttcctcaaaaagacctggcctacccatccatcaggagacttcaggtctataaa

ctggcTTAGATCTGATAACTGGGTAAGAGatatgagcatactagctttcTGCCCACTAGAATTTTCTACCTTAAAGTCAAGAAGCACCTTAGTAAGCATC

TCATCT-ATTTCATTTCTA-GGGATTCCATAGTCTTAGCTACAAATCCCTGCCAatcaaagcactcagaTTCCAACTCTGGACCTGTGGcttcttggggt

aattgtgtccaccctgtctccgctgtttaataaacattgctgcctggcttttgttgctcctggaacccatttctctcactgagatcagcaaccccagtct

cattgcagcagtgattctccaagagtggtcctcagacaagccttatcggtatcacctgagaacttgttagaaatacacaattttggcactgttctaagaa

ctctccatgcattatcttattgactctcccaacatcgtaatgagatagcgattaccaatcagtttcctcatggaggttcagagagggaagtaatttgctc

tgaatcacataaccagtaaacgatggatataaaacttgaacccatgcttaactgaccccagttcccaggctcacattctcctatgtcgtattaagtcctg

aatcacaggaAAGGACAGACAGGAGATTTTACAGCTGGGAAGATTTGAATgtcctctcactcatccagtttataggaaAGTTTGGGGCATATGTGTGTGA

GTCAATTTTaagagtgttagactgagaaaaataacatatagggttcaatcgcccaaattcagaatttaatgtactggcacaa------------------

----------------------------------------------------------------------------------------------------

----------------------------------------------------------------------------------------------------

---------------------------------------------------------------------------------------------GTACCTG

GAAGGGATATTAATGGTCAGCTAGATTGgctaattgaagtctagacttaaaaagaagtctaagagaaggaagcagagcaagatggctgaataaaagtctc

cactgatcatcctccctgcaggaacaccaaattggacacctatctacacacaaaaagcaccttcataagaaccaaacttaggtaagtaatcacagtacct

ggctgtaactttgtatcactgaaagaggcactgaagagagttggaaagacaatcttaaattgccaacaccacccctcccctatctcttggcagtggctgc

atggagcggaaagaaaatgtgggcggggggagggaaagtgcagtgattgggtgacttgcactggaactctgtgctgccaacactgggcagaactcaacca

gcgcccacaaagggagcatttagacctgccctggccagagaagaattatctgtgccaggagttggaatgttaattctggcaagccttgccaccatgggct

aaattgctctggctaaattgttctaaataaacttggaaggcagtctaggccacaaagactgcacttactaggcaagttctagtgctgtgaactggtgggc

aacacgcaacctagtgagacatcagctggggcagccaagggagtgcttatgttacgcttcccccaaccccatgtgtcacagcttgcagctctgaaagaga

ccccttcttttcacttgaaaagagaggtattaaaaaaaaaaaaaaactttgtcttacaagttggatgctaaatagctcagccacagtaggaagccacagt

aaggcactggggagggttgtgaggctcccattccagggcgtagctccaggatatttccagatacaccctggagctgaaaggaatccactaccttgaaggg

aaggacccagtcctggcaggattcattacctgttgactaaagagccccggggagctgaataaccagcagccataatcaggtagcacatgctataggcctt

gggtgagactctgagacatactggcttcaggtgtgacccggcacattcccagatatgatagctacaagaacaggcccccttgcttgagaaaaacagagag

aggagtaaagaggactttgtcttgaacttagcagctcaactacagtagagaagagcaccaagtgggcattggggtcccccgttccaggccttggctcttg

tacagcatctctggacctaccttgggccagaggggagcccactgccctaaagagtgagtcccaggcctggcagcattcaccacaagctgaccaaagagcc

cttaggccttaagtgaacattggaggtaccctggcagtactccttatggacctgtggtggtggtggtgaacatgaggagagactcctctacatagggaag

gggaaggaaaggatggaaaaaacttcattttgtggcttgggtgccagctcagccacagtggaacagagcaacaggtatattactaaggtttctgactcca

gaccctggctcctggacagtatctctggacctgcctaaggcctggaagaatttgctgcccttaagggaaggacataagcctggttggttttaccacatgc

taattgtagagccctagggccttgagtgaacatagccagtagtcagctagtggttccagttggtgctgcttttaggtccaacccagttcagtcccagtag

tggtgaccatgggggtagttgtgtaacctctcttccagctccaggcatctcagcacagagagagataatctgtttgtttgggacaaagtaagaaaaaaga

acaagagtttctgcctggtagtccagagaattcttctggattttatccaagaccaccaaagtggtacacagaaaaccacagaataacattgtaattgtgg

tgtgtaaactatgcataagttacgtagaaagacaaaaagatgaactgatccaaatatctgcaacaacttttgaagacatagatagtacaataaaatataa

acagaaacaacagaaagttaaaaagtggggagatgaagttaaaatgtagaagttttatcagttttctctttgtttgtttatgtaatcagcattaaattgt

aatcaatttttttaaaatgggttataagacactatctgcaaccctcatggtaacctcaaatctaaaaacacacaacagatacacaaaaaataaaaagcaa

gaaattaaatcataccactagagaaaataatcttcaccaaaaggaagataggaaggaaggaaggaaagaaagaagagaagaccacaaaacaaccagaaaa

caaataacaaaatggcaggagtaagtccttacttattaataataacattaaatgtaaacagactaaagtctccaatcaaaagacgtaaaacgtggctaaa

aggatgaaggagcaagacctgatgatccgttgcctacaagaaacacacttcacctataaaggcacacaaagactgaaaacaaaagggatgcaaagagata

ctccaagcaaatgaaaaccaaaaaagagcaggagtggctatactaatatcagacaaaataaatttcaagacaaaaaccataaaaaaagagacagagggag

ttattatatagtgataaaggggtcaatttaacaagaagatataacaattataaatatataggcacccaacactagagcacccagatatataaagccaata

tcattagagctaaagagagagatagactccaacaggataacagctggataatttaacaccccactttcagaccctgaaacagatcatccagacagaaaat

caacataggaacatcagacttaatctgcactatagaccaattggatctactagatatttacaaaacatttaatccaacagctgcaaaatacacatttttc

tcctcagcacatagatcattctcaaggatatatcagatgttaagccacaaaacaagtcttaaaacattcaaaaaattgaaatagtatcaagtatattctc

tgaacacaatggaataaaactagaagtcaataacaagaggaattttggaaactccacaaacacatggaatgtaaacaatatgctcctgaacaactggttg

gtcaatgaagagattaaaagggaaactgaaaaatttcttgaaacaaattatgatggaaacacaatataccaaaagctatgggttacagcaaaagcagttc

taagaggttaatttatatagctctaagtgcctacatcaaaaaagaagcaaaccttcaaataaacaacctgaaaataaacgcttgaagcaatggatatccc

atttactttgatgtagttattacacattacatgactgcatcaaaatatctcatgtaacccataaaaatacacacctattatgtactcacaaaaattaaaa

attaaaaaatAAGAAGCCTACAGTTAATGAAGTTGAAAtgccagaaattctctggaatgacaggaaggagtccagagactcagggaagtggatctgttgg

aatggatctagtctatctgttggatctgttggaatggatctagtctatgtgcaagctgctgggaggcccagggggacactcccttcaatccaggtattag

gaaatgttttgatgagttgtgggaggagggcaggatgcttaagagctctgtagtggctattctttatagatcagtgatgacagtgggacatgatataatc

ccatagttctcaaacttttgtgtatttcaggatcacctaatgggcttgttaaagcagtctgccatgctactactggtggtaattaaagtaatcatgatat

gttgacagatcctattcctgtgcccctttccatggtgcacctaaaacatacgagcaatccaatcccagaatcccatttggaagttctattttatggggcc

aattcaatgtcagtaagtgtcccagcaagaaatagataacacttaaaatggaagacttggagaaaggtttagtaaagaagctatttacaaagatgtaagt

gggtgaaaagagaccacaggggtgtttggtaccatgaggctattaagaacatggttagactgcaaaaaatttctccagttctgtaggatatctgttcact

ctgatgatagtttcttttgctgtacagaagctctttagtttaattatatcccatttgccaatttttgcttttgttgcaattgcttttgacattttcatca

tgaaatcttttcctgtgcctatgttttgaatggtattgcctagagtttcttctagggtttttatagttttgggttttacatttaagtctttaatccatct

tgatttcatttttgtatgaggtgtaaggaaggggtccagtttcagtgttctgcatacggctagccagttttcccagcaccacttattaaatagggaatcc

tttctccattgcttatttttgtcaggtttgtcaaagatcagatggttgtagatgtgtagtcttatttctgagatctctattctgttccattggtctgtgt

gtctgtttttgtaccagtaccatactgttttggttactgtagccttgtagtatagtttgaagtcaggtagcatgatacctccagctgtgttctttttgtt

taagattgtcttggctattccactgcggcctctcagggagtgagggaaagggagagcgtcaggataaatagctaatgtggatggagcttaatacttgggt

gaggggttgataggtgcagcaaaccaccatggcacatgtttacctatgtaacagacctgcacatcctgcgcatgtatcccaaaatttaaaataaaataaa

aattttttaaaaagaacatggttatattcctaaatctaaaaaggaaaaacaggggatagctcagcaaacctggataggagagtcatggagaaaCCACCTT

GAAAGAAGTTAAAACCATTAGTTGAGGGATGtatccaggttgaggaacacaGGGAGACAACAGAGGAGAAGAACCTCAACTTAACTCTTCTccctgctct

aatgtcctcagaagctcccttgtgcctaaacccagtcagaagctagaaggtcagcaacctcactgatgtaacccaccaagattggcttcctagggcagag

aatagaatggaaaagaatggaatgtgtgtctgaagggacaagcttataatatccttttctcaggtgagtcttgacacatttttgtgtcaaagttaggtag

gtctcaaaatgaattgagtggtgtacaatcttttgatattctctggaagagattgcacaagattgaaatgaccacttccttcagtgcttggaGGAATTAG

CTGTTTGTGCCCTTTGAGCCCAGAGAATGTTTtgtgggaagattttcaattcctgattccatttctttaatagctttagaatgactttctattttttatt

tatttgtgcaccctggtcagttaga-----TTTTTAAGAAATTCATCCATTTCCTCTAAACTAAATttttggcataaggttcttatcattttctttttgg

tgtctgcaggctctgcaactggttatatgtacctttttccttttttcttttctttttttttttttctgggacagagtcctactctgtcactcagactggg

gtgcagtggtgcaatcttggctcactgtaacctcggcctcccaggttcaagcaattctcctgcctcagcctcccaagtagctgggattacaggcatgtgc

caccatgcctgggtaatttttgtattttttttagtaaagacagggttttaccatgttgtccaggctgctctagaactcttgacctcaagtgatccccccg

cctcagccccttttttcttttttttttcattttcagttcaggagagttttagcttaattataggctacagaaccagctttgggcttcatctatcctttct

aatatttactgtttcctatttctctaatcctagctctttatttcttccctttactttcactgggcttattttgctatagtGGAATGCAGAGGGATGAGTA

TTCCAGGAAGGCACAAAAactgtgccaagtcttggagctagggatgagtgggaaagggacatgttcaaccattttaagccattccctccccacctcccag

ctcccagatatgtgcccctcgcaggaggagcccaggaatgggccaaacacctcacttctttgctctgagggccaccccagccctc---------------

----------------------------CCATCAACAGCTCTAGAAACCCAATGGTCCTTCCTGGAAAcacggggcctgcatcaatcagaggtgtttgaa

ccatgtccctctgggcctgaggggcagaaggggacacaatatgtaatgtaaggagcccctgtcatcagaaatCTGACTTAATCTGTTTCAGATATTAGAC

TTCCACATAAAAgttgacttggaaaaagacttctgctgctaaacaaaagttgaaactgccttggtgataaaatataagcagaccagctttctcttctagc

tttccct---------------------------------------------------------------------------------------------

----------------------------------------------------------------------------------------------------

----------------------------------------------------------------------------------------------------

------------------------------------------------CTCATTTCCCATAAGATTTTGGTCAAGTTATTTAATCTCTctgcaTCCGTTT

CCTCTTCTATGAAATGGGCATGATAATAATGGTatgtacctcctcaaactaaatatataacgtga-ACAGAGTCCTTAGCACAGCACTCTTTCTCTACAG

GAGTTAattttcattgtttttctctttcctgttggagaaagtaagaagaaaacagctcctttatggcttcccatggtgaatggctggggcgcgtctgtgt

ccctttcTCCTCTCTGGCTCCTTGTGGCCTGaacagccagaaggaagccatgccatgctgtttcagccctCAGCTTCCCTCTTGCATTTCCTAGAAAAgt

ctttggtgcCCAGCTCCAGCTCAGCAGATTCAGGATCCCCCTtcatcatgacttggtcaacgccctgctcaggccaaggtcctctgagagttccaagctt

ctcca----CTCCCTATAAAAGGCCGGCGGAACAGCCAGAGGAGCAGAGaggcaaagaaacattgtgaaatctccaactcttaacctt------------

-CAAC---ATGAAAGTCTCTGCAGTGCTTCTGTGCCTGCTGCTCatgacagcagctttcaaCCCCCAGGGACTTGCTCAGCCAG-GTAAGTCACCTCCCT

TCgact-CTCCCTCTCTTTCCCTCTGTTTCTCTattcaaggaagacctaagcccgagtgctcctccactttttttttagattgagtctcattatgttgcc

caggctgaagtgcaggggtgcgatcttggctcattgcaaccttcacctcccaggttcaagcgattctcttgcctcagccttctgagtagctgtgattaca

ggcacccgccatcacgtgcagctaatttttgtatttttagtagagaaggggtttcactatgttggccaggctggtctcaaactcttgacctcaagtgatc

ctcccgtctcggcctcccaaagtgctgggattacaggcgtgagccaccaggcccagccaagt---------------------------GCCCCACTTCT

AAGCCCACCAGAATAGTAAGGCTCCTCAGaggttcactttaacatctaattttaaagatagAAAGCTGAAGCCCATGTTGGAGGCAGAAGGGACCCtagc

catccacctccaggttattgcagagcaagaatgaaacctaagcttctgactccagatttagggccttttctttgacctcaTCTGATCGTCCCAAACTCTG

CAGATCTGGGACCACACCcaggacctttcccactggccttgcccgtggcctcccctagatggctgtgacatgtctccaccatgcagctgagcctttgaga

tcctgaggcacatgtcacaggtcccacctcacctcagggtctagggtgggagtgctgggcttgggggtgagtaagatctatttcttcctctttgctttgc

atcccatacag-atgctccctgctgtattcaagctgagaaaagcctaacacatcctcaaagtctttttctttgtaactatttctag-atgcactcaacgt

cccatctacttgctgcttcacatttagCAGTAAGAAGATCTCCTTGCAGAGGCTGAAGAGCTATgtgatcaccaccagcaggTGTCCCCAGAAGGCTGTC

AT-GTGGGTAGAAAAATCCCTGCTCACCTG-GCTCCTCCCCACTCCCACATTCCCCAATCCAAAGTTCTGCCCCAGGAGACAGACGTCAGACTGACTTGA

GATCTTAGGATGAGATCTAGCCAGACTGTGTGATGCAAATCCTCCAATTTTGGCTGC-ACAACAGGTCCAAAGAGGACCTATAATTTCC-CACACCTTGT

TTCCTGGATGGGCACCAGCCCACACCCTTTAGCAGATGCCAGGATCAGTTTCCCAGGGGCAGCAAGAGCAGTGGCTGCCTCCAGAGACCCCTTCTGTCCA

CACACCTCCTACTTCCTGTCCTGGAGGGGTGCCCCTTCACCTGTAGTAGGTGGACCAGGCAGGTTTAGAACCCAGTGTGTCATCTCCTGGGTAAACCCTC

AAAGGGTTCCATCTAACTGTGCCAGATCTCCTTCCTCCACAG-CTTCAGAACCAAACTGGGCAAGGAgaTCTGTGCTGACCCAAAGGAGAAGTGGGTCCA

GAATtatatgaaacacctgggccGGAAAGCTCACACCCTGAAGACTTGAACTCTGCTACCCctactgaaatcaagctggagtacgtgaaatgacttttcc

attctcctctggcctcctcttctatgctttggaatacttctaccataattTTCAAATAGGATGCATTCGGTTTTGTGATTCAAAATGtactatgtgttaa

gtaatattggctattatttgacttgttgctggtttggagtttatttgagtattgctgatcttttctaaagcaaggccttgagcaagtaggttgctgtctc

taagcccccttcccttccactatgagctgctggcagtgggtttgtattcggttcccaggggttgagagcatgcctgtgggagtcatggacatgaagggat

gctgcaatgtaggaaggagagctctttgtgaatgtgaggtgttgctaaatatgttattgtggaaagatgaatgcaatagtaggactgctgacattttgca

g-----aaaataca----ttttatttaaaatctcctacacagtggtgttttcttcaggagtaactgccaaccagtaggggctctcagaggtgtgggtgga

tggcatgccagtggaaggcagatgtacagaggccctggccatggggctagcctggctcatctgagtttcaaggagggcctgcctcaccagcccaggctgt

tctggaacaatggctccagtccccagcatgggcgctcctggctcgcaactcatccgtttcatattcagttcatttctttctttaaaaccacactcctgcc

aaaagtaaacccatccctccccctctagtctcccaccccaatctatgcctccccactccaagcagcaagtgattcagacttcatgtgcacctgacactca

gagggaaaccagatgaccaagtcctagtgagcctcaggattggtagggaagagggtgcttgggcctaatggactttagaggatggggaagtatggtcata

gcaaagattcttttaggggaggtccaagatagcaatgtggggagagggcctggggggaatatccccaaacggtccgatatgggcttccagaatgtaaaag

ttggagacatgtagaaatgaaagttgagggcaggttctagaggaattagaatatcagggtggggagtttagactcagttctgaaggcaggaggaaggtgc

aaaggtttttgagtcatccagagggaagaatggatgccaggaagactagctattggctgtggataagatggagttgagaagggagggcggaaaacagcct

actgcaatagtccaagcaagagaaaataggggctgaagaagggcagtcatgGGGGTGGAGAAGAGGAGGCAGATTAAGAAGACTGGGCCAGat-gtgtga

ca-tgttgaatggggaatgagggaaacacagcatgagacctttgccctctaaaagtgatctttaaaggcgtgccaaccccaagacctagaaatacttcct

tccttggctattcttccttgaatcacacgttgtacaaattgaagcttgtgattgtcccttttaaagcgttggccctcctcttcctatatgtccaccaagg

gtttatcctgctcttcccaagacagcctcactcctccagataagctgggtgggcatctcaatcagacccagaacctacccatgcctggacacccactgag

atcttcctcaagaagagatgggcactgctgacaacagcagggcctgtcccccagatctggtaccacactgcatcaggcagtacacttggcaatgtgggac

acctggaggagttgtcctaggaGGCAACAGCTCTAGGCTCTAATGCCAGATCTGCCCCTGaattgctgcatct-ctctttAGCCCTCAGTCACCTCATCT

ATAAAATGGGTTTAGCAACAttttacaggcagacaacctcacatgatcccctccagctagggcagttgtgatttggtgataacatcaccccccaagccct

ctgggcagggcagggagaagaagagtcctgcacccaaatgtggatccagctgtgaaccccagctttctgttccactgtaccactcctGGCAGCCAAATCT

GGAGGCAGAGAATCTGCaccaccaccctgactcaaagcttacaccccaggacaaaggcagtcactgagagctagtgagtcacttattagcccaGTTCCCC

TGGCACCAGGTGCAACATGTCTCCCTCCTCCtgacccatccttcagcccctggctCCCACCTCTCAATGCCAATCAAGTTTTCACTCTGAAATCCaagag

acccagagggttgggggttgatgattgtataatttaaatgtt------TAAAGTGCAACAACATAGCTTATCAAGACCAAGCAGAtcctctgtgacctag

caaaagcagggcagaaggaatggtgtagggctggtagtttcggggacaggtgaagccatgtggtttcCAGAGCCCACAATGGAAAGAAATCTGC---TCA

TTTTCTTTTTGACGGGCAGTGCCTCAGCATTTTTCTGTGCCTCTGAACCCATCCAACTGTGTCCAAGGCGCAGGCCTCTTTGCCTCTCTTCAGCTTGAAT

--CTGTGAACAGAGAAtaagagagaaggctgagccacccaagccaccactgggtgggcaacgcacaagccccgcctcctccggctgccaggtcccctaaa

ctgctcttgctaGGCCTTGGGCTCCCCTCTGTAAATGAAGTTGCTG---CACAG--GATGTGTGGTTTGCACGCTGGGGAACAGCCAAAGCACCAGGAGC

TTATGAATTGAATTTCCTGGACCCCTCTCCT---GGTTTCAATT-TGGTGTATCAGGGTGGGGCTGAACAG-CCT-GAAAACATTCTGACGATTAGGTGA

GATGAGAAATTCCCATCCTGGAATAGCTCTTTGCACCAACTTT-AATGTTTCTGTGTCCATCTGGGGCCTTACTTGTTGCGTTGGACTCATGTGTTTGTG

TGTGTGTGTGTGCGCGCGCCTTGTGTGTGTGTGGGTGTGTGTATTTTTCTCTTCTGATCTGTCTACTTCCTAAACTGGACTATTGTCCAATCTATAGACC

AACTCTATCTCTTACTCTCTGGTTGATCTTAG-ACAAATATCTCTACCTTCCTGAACCTCACTTTTCATGATAACAAAAATATGTGccccaAGAGGATCG

ACAGAGA-GA-ATAT--ACA--TGAAAAAGCCTTAAAATACTGACTGGTTTGTGAAAGCTACTCCAATTAAGTTTGTAA--A-----GACCCTGTGATAC

AGCTAT-GTTTATGACCC-T-TCACAGATAAGGAATACAGAAGGATACACAGACAATTCAGTACAAAGG-AGTTACCTGTCCACGGTCACCCAAGACTAT

GGGTTc-CAAATCCAGGGGCAGGAACTCATCCTGGTTCCCTTGCTCTtTCCAGTGCACAATAACCCCTCATGCTTTTG--TTA--AAC------CTCTGA

GCTCATAATGCTGGGATACAATACTTGGCACCTTGGAGTTTCAGGAAACACAAGCCACTGCAGGAgcacagatgcatccttcctcaCACCTGCTAGGAAG

CTCAGCGCTCCCTCTAGCGCCCAAGtgtggcttgcacccaggtgctggcagctctg--------GGTTTAGAAACCTTATTCTGGTAAAATACTGTCATC

TAGtgtctactcttgtaattccaggctggcggggttctgttctctagggagagattgagcaggtGATCACTTAC-ATTAAGCCCTCATTGGAGCAGATGG

AGCTGGTATTTCTGTAACACAGGATTGCCCTCAGGGGAATCTCTTGCTCCGCAAATGAGAAGCAACATCTGGAGAAGGGTACCTGCA-CTAGAAGAGGAA

CACAGACGAtggtttgcatccatttctcaacctctcacctgtagcacaatttttaaaaaacaaacaaaaaaacgcctcccaggacaagccctggcttggg

gaccacatccagacggtgccggcatagctctgcctcatcagccaccttgttctggggctaggtaaagagtcagaagattggggttcagatctgagctccc

cgcaccccagctgagtggccctgaggaagatgtgccttcaccctggccccagacttcccatctgtaaaataaaactttttggagcctggtttctaaaggc

tcatccagttttggtatttcaccacatgtgcatggttctgccccagtattcatgtccaagGGCTCTGGACCCCACACCCTGGGGacctggttagtctgat

gcaggacacagtggtccacagttcacagacctgatctggggaagattaggaattgttcgaggagtccaaactgatgagatga------------------

CAAGGGAAGAGCTCCCAGGAACTAGCTAGCTGCCCTACTCagtctcttgaatcccctcctgccctggtccttagctctttcccagcccccaacccccact

ctgtccatttgtgcattctcctcccacaccacatcccgcaagagacctgcattccaatcccagcttcttcctcctagtttttctgtttgcaccccctttc

atgcctcttactaggttggtattctccaaacagtcttggagggcagacatcatcaactccatttcactgatagagaaagtagagtttgagagctaaagtg

acttgcccaagagtgaccaattgttagagcccggtcttgccttctgactctacttggtgtctgtcacactgtagaagctacacccaggctgtcccctgga

ccttaggcctcccagctttacccgagtaacctcacaggttcccttccttgggtctggatgaagcagagcctccttgTACTGAAGCTGTCAGTTCAGTTCT

CTGTGTTTGCCCCaactcatggttctcctactagccctcaccccagcctgtccaagttcaccattcctcctctTTAGGTAGGAAGAGGGGAGATAGAGtt

ggtgagttcaaggagaagcctgctccaaccccagagtggctgaccacgtttctgccctccccagagagaagcaaaatgatgcctgcca---CACTCAC--

TGCTCTTGCTGTCCACATCTTCCGGCCACATCccagctagcagcaagcacaccagggctgtggtgatgatctgcat--gtcttctggtctggcttgggcc

ttcctggagcagctttgatgagcctggtgaagctaagagctcaccactgtgccgtcctgcaatgctgagggcttttatttgaaaaactccacccaaaact

ccaggccatggtgggagccacaagtgGGAAGCCATAGGGACAGGGAACAGTTGCTCACACCCAGCGaccatgTGGTAAACTCCTTTCCCCACCTCTCCTG

GCtggtattcgtggtggtgatggattgataggttgcattgggaacttccctaagtgtgggagcagataagaatcttgaaaaagtgCGTGGGAACTGTCCT

GGTTGAGTGGGAAGCAAAGAAAAGTaagTGAGAGGAAGTGGCCTCTGTGTGGCTTGTCCAACCCTACGTCATTCCATCCA-A---GTA-CTACTGCTTC-

AGGGCAG-A-AGAGT--CTG-GACCTG-AAC--AGAG-A--TACACCTGCATTTGAATCCCAGT-TCTGCAGAGGAGGGATTACATAAACTTACCCAAAT

TCATATTCACACAAAATGCAAATAGCAATACATGTCTCAAGGACGTGCGATAAGAATTAAATAACATTCTCTATGTCCAGTGACTGGCCTAGATATTTGC

CATTTctatttTTTCTATTTTTTTAATCCTCA
