# Supplementary material for: Pseudogenization of the MCP-2/CCL8 chemokine gene in European rabbit (genus Oryctolagus), but not in species of Cottontail rabbit (Sylvilagus) and Hare (Lepus)
Source: BMC Genet. 2012 Aug 15;13:72. doi: 10.1186/1471-2156-13-72 (PMC3511233; doi:10.1186/1471-2156-13-72)
Supplement: Additional file 6 — Rabbit CCL13 ortholog named ‘ CCL8’ or ‘ CCL7’. [file 1471-2156-13-72-S6.doc]

Additional File A6

Rabbit *CCL13* ortholog named ‘*CCL8’* or ‘*CCL7’*

A: *CCL13* ortholog named ‘*CCL8’*

Link: <http://www.treefam.org/cgibin/TFseq.pl?id= ENSOCUG00000013412>

Descriptions

| Sequence ID | ENSOCUT00000013408.1 => [Inparanoid](http://inparanoid.cgb.ki.se/cgi-bin/eid_search.pl?id=ENSOCUT00000013408&idtype=enst&start=on&species=All&conf=0.05) |
| --- | --- |
| Transcript ID | [ENSOCUT00000013408](http://www.expasy.ch/cgi-bin/sprot-search-ac?ENSOCUT00000013408) |
| Gene ID | [ENSOCUG00000013412](http://www.expasy.ch/cgi-bin/sprot-search-ac?ENSOCUG00000013412) ([ENSOCUT00000013408.1](http://www.treefam.org/cgi-bin/TFseq.pl?id=ENSOCUT00000013408.1)) |
| Symbol | ENSOCUT00000013408 |
| Species | [Oryctolagus cuniculus](http://www.ncbi.nlm.nih.gov/Taxonomy/Browser/wwwtax.cgi?mode=Info&lvl=3&lin=f&keep=1&srchmode=1&unlock&id=9986) (RABIT) |
| Description | Small inducible cytokine A8 precursor (CCL8) (Monocyte chemotactic protein 2) (MCP-2) (Monocyte chemoattractant protein 2) (HC14) [Contains: MCP-2(6-76)]. [Source:Uniprot/SWISSPROT;Acc:P80075] |
| Display ID | ENSOCUT00000013408_RABIT ([TF334888](http://www.treefam.org/cgi-bin/TFinfo.pl?ac=TF334888)) |
| Related Family | [TF334888](http://www.treefam.org/cgi-bin/TFinfo.pl?ac=TF334888) ([ENSOCUT00000013408.1](http://www.treefam.org/cgi-bin/TFseq.pl?id=ENSOCUT00000013408.1)) |
| Xrefs | | RefSeq_peptide | [ENSG00000181374](http://srs.ebi.ac.uk/srsbin/cgi-bin/wgetz?-e+%5BREFSEQP-alltext:ENSG00000181374%5D) | | --- | --- | |
| Peptide | MKVSAALLCLLLLAAACSSQALAQTETKPALTACCFSFVRKRIPLQRLVSYRKTSKACVK EAVIFRTRRDQELCADPMQKWVQDTMRVLWKRHSLRTA |
| Nucleotide | ATGAAGGTCTCCGCAGCTCTGCTGTGCCTGCTGCTCCTAGCGGCCGCCTGCAGCTCCCAG GCACTCGCCCAGACAGAAACGAAGCCAGCCCTGACCGCTTGCTGCTTCAGCTTTGTCAGG AAGAGGATCCCCCTGCAGAGGCTGGTGAGCTATCGGAAGACCAGCAAGGCCTGTGTCAAG GAGGCTGTGATCTTCCGGACCAGACGGGACCAGGAACTCTGTGCTGATCCCATGCAAAAG |

B: Rabbit *CCL13* ortholog named ‘*CCL7’*

Link: <http://may2010.archive.ensembl.org/Oryctolagus_cuniculus/Transcript/Sequence_cDNA?db=core;g=ENSOCUG00000013412;r=scaffold_23:1301667-1303020;t=ENSOCUT00000013408>

Transcript: ENSOCUT00000013408

chemokine (C-C motif) ligand 7 [Source:HGNC Symbol;Acc:10634]

Location: [scaffold_23: 1,301,667-1,303,020](http://may2010.archive.ensembl.org/Oryctolagus_cuniculus/Location/View?db=core;g=ENSOCUG00000013412;r=scaffold_23:1301667-1303020;t=ENSOCUT00000013408) forward strand.

Gene: This transcript is a product of gene [ENSOCUG00000013412](http://may2010.archive.ensembl.org/Oryctolagus_cuniculus/Gene/Summary?db=core;g=ENSOCUG00000013412;r=scaffold_23:1301667-1303020;t=ENSOCUT00000013408) - There is 1 transcript in this gene

Show/hide columns

Search:

| Name | Transcript ID | Length (bp) | Protein ID | Length (aa) | Biotype |
| --- | --- | --- | --- | --- | --- |
| Novel | [ENSOCUT00000013408](http://may2010.archive.ensembl.org/Oryctolagus_cuniculus/Transcript/Sequence_cDNA?db=core;g=ENSOCUG00000013412;r=scaffold_23:1301667-1303020;t=ENSOCUT00000013408) | 267 | [ENSOCUP00000011538](http://may2010.archive.ensembl.org/Oryctolagus_cuniculus/Transcript/ProteinSummary?db=core;g=ENSOCUG00000013412;r=scaffold_23:1301667-1303020;t=ENSOCUT00000013408) | 89 | Protein coding |

Top of Form

Bottom of Form


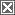
Transcript and Gene level displays

In Archive EnsEMBL a gene is made up of one or more transcripts. Views in Ensembl are separated into Gene based views and Transcript based views according to which level the information is more appropriately associated with. This view is a transcript level view. To flip between the two sets of views you can click on the Gene and Transcript tabs in the menu bar at the top of the page.

.

[« Exons](http://may2010.archive.ensembl.org/Oryctolagus_cuniculus/Transcript/Exons?db=core;g=ENSOCUG00000013412;r=scaffold_23:1301667-1303020;t=ENSOCUT00000013408)

[Protein sequence »](http://may2010.archive.ensembl.org/Oryctolagus_cuniculus/Transcript/Sequence_Protein?db=core;g=ENSOCUG00000013412;r=scaffold_23:1301667-1303020;t=ENSOCUT00000013408)

[cDNA sequence [
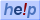
](http://may2010.archive.ensembl.org/Help/View?id=175)](http://may2010.archive.ensembl.org/Help/View?id=175)

Key


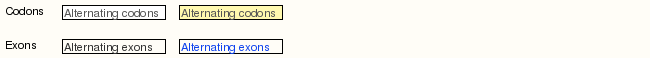


1 ATGAAGGTCTCCGCAGCTCTGCTGTGCCTGCTGCTCCTAGCGGCCGCCTGCAGCTCCCAG

1 ATGAAGGTCTCCGCAGCTCTGCTGTGCCTGCTGCTCCTAGCGGCCGCCTGCAGCTCCCAG

1 -M--K--V--S--A--A--L--L--C--L--L--L--L--A--A--A--C--S--S--Q-

61 GCACTCGCCCAGACAGAAACGAAGCCAGCCCTGACCGCTTGCTGCTTCAGCTTTGTCAGG

61 GCACTCGCCCAGACAGAAACGAAGCCAGCCCTGACCGCTTGCTGCTTCAGCTTTGTCAGG

21 -A--L--A--Q--T--E--T--K--P--A--L--T--A--C--C--F--S--F--V--R-

121 AAGAGGATCCCCCTGCAGAGGCTGGTGAGCTATCGGAAGACCAGCAAGGCCTGTGTCAAG

121 AAGAGGATCCCCCTGCAGAGGCTGGTGAGCTATCGGAAGACCAGCAAGGCCTGTGTCAAG

41 -K--R--I--P--L--Q--R--L--V--S--Y--R--K--T--S--K--A--C--V--K-

181 GAGGCTGTGATCTTCCGGACCAGACGGGACCAGGAACTCTGTGCTGATCCCATGCAAAAG

181 GAGGCTGTGATCTTCCGGACCAGACGGGACCAGGAACTCTGTGCTGATCCCATGCAAAAG

61 -E--A--V--I--F--R--T--R--R--D--Q--E--L--C--A--D--P--M--Q--K-

241 TGGGTCCAGGATACCATGAGGGTCCTG

241 TGGGTCCAGGATACCATGAGGGTCCTG

81 -W--V--Q--D--T--M--R--V--L-
