# Supplementary material for: Pseudogenization of the MCP-2/CCL8 chemokine gene in European rabbit (genus Oryctolagus), but not in species of Cottontail rabbit (Sylvilagus) and Hare (Lepus)
Source: BMC Genet. 2012 Aug 15;13:72. doi: 10.1186/1471-2156-13-72 (PMC3511233; doi:10.1186/1471-2156-13-72)
Supplement: Additional file 7 — Nucleotide variation at CCL8 genes within and among leporid species. [file 1471-2156-13-72-S7.doc]

**Additional File A7**

**Nucleotide variation at *CCL8* genes within and among leporid species.**

TATA-box |->UTR Forwar-

Orcu *CCL8ps* WGS CAAGGCTCAGTTCCCTATAAAGGGCAGGCGGGGCCCCCAGAGGAGCAGAGAGGCTGAGACCAACCCAGACACCTGCAGCTCTCCCTCCAA 90

Orcu *CCL8ps* gDNA .......................................................................................... 90

Bumo *CCL8ps* gDNA ................G.G..A.................................................................... 90

Syfl *CCL8*  gDNA ....C................A.....................G.............................................. 90

Leti *CCL8*  gDNA .....................A.A.....A.......................................G.................... 90

Legr *CCL8*  gDNA .....................A.......A......G.C..............................G.................... 90

ds cDNA primer FrameShift

Orcu *CCL8ps* WGS GCTCGGCTCCTTGAGTGCCAGC ATACAG----------TGCTTCTGAGCCTGCAGCTCGTGGTGGCCGTCTTCAGCCCCCAGGTGCTC 168

Orcu *CCL8ps* gDNA S..................... ......GTCTCCGCAr....................A............................. 178

Bumo *CCL8ps* gDNA ............A......... ......GTCTCCACAG....................AC..C.............T........... 178

Syfl *CCL8*  gDNA ....R................. ..G...GTCTCTGCAG....................A...C......T......T..........T 178

Leti *CCL8*  gDNA ...............C...... ..G...ATCTCCGCAG....................A...C.............T........... 178

Legr *CCL8*  gDNA ..............AC...... ..G...ATCTCCGCAG.......T............A...C.............T........... 88

Leeu *CCL8*  cDNA ...............C...... ..G...ATCTCCGCAG....................A...C.............T........... 88

Legr *CCL8*  cDNA ..............AC...... ..G...ATCTCCGCAG.......T............A...C.............T........... 88

**Intron1**->

Orcu *CCL8ps* WGS ACCTAGCCAG GTGAGGCTCTTCCTTCGCTAAGTCTTAGCACTTGAGCTACCTCAGCTACCACCATCCAAGTGGGAGCCATCCCAGAC 255

Orcu *CCL8ps* gDNA G..C...... ................C............................................................ 265

Bumo *CCL8ps* gDNA G..C...... ................C.....G....................G................................. 265

Syfl *CCL8*  gDNA G..C...... ...........T....C.....G..................A................................... 265

Leti *CCL8*  gDNA T..C...... ................C.....G......C.......G...A........G.........................T 265

Legr *CCL8*  gDNA G..C...... ................C.....G......C.......G...A.......TG.........C................ 265

Leeu *CCL8*  cDNA G..C...... 98

Legr *CCL8*  cDNA G..C...... 98

Orcu *CCL8ps* WGS AGGCTGCATCTTCTTCATTATGAAGAAGGAGAGAGGCCAAGACAGGAGCCAAAAAAGAGCCACCTCCCATCAGAGCCACAACTTAGTTCC 345

Orcu *CCL8ps* gDNA G......R..........y.........R....................M........................................ 355

Bumo *CCL8ps* gDNA .................................................T.......................................T 355

Syfl *CCL8*  gDNA ............................................A....T.............................C..G....... 355

Leti *CCL8*  gDNA G................................................T.....G.................................. 355

Legr *CCL8*  gDNA G................................................T.....G..............................C... 355

Orcu *CCL8ps* WGS GAGGTTCTGAGGCCAGTCCCTACGATCATCCTTGACACCAGCTTTGGGGAGTCAGGCTGAGTGCAGCCACTACGTTTACGCCGCCTTGTT 435

Orcu *CCL8ps* gDNA .....C................Y............................W....................Y.y.A..R.Y........ 445

Bumo *CCL8ps* gDNA .....C................T......................A.....A...............Y........A.....A....... 445

Syfl *CCL8*  gDNA .....C.......................................A...G.A.................T......A............. 445

Leti *CCL8*  gDNA .....C...........................T...........A.....A.....................R..A..A.......... 445

Legr *CCL8*  gDNA .....C...........................T...........A.....A.........C..............A..A.......... 445

Orcu *CCL8ps* WGS TGGGGAAGTGGTACAGAAGGAAGCACCATTTCTGGTCGTGGGTGTGGAAGACTTTTCACGCAGCAGTGGGGAGGAAGAAGTCCCTGCCTG 525

Orcu *CCL8ps* gDNA ..........R...............................................S............................... 535

Bumo *CCL8ps* gDNA ...........C.......................A.T....................G.............T..............M.. 535

Syfl *CCL8*  gDNA ...........C.A............................................G.......A....................... 535

Leti *CCL8*  gDNA ...........C..................-...........................G............................... 534

Legr *CCL8*  gDNA ...........C..................-...........................G............................... 534

Orcu *CCL8ps* WGS TGGTCTCACATTTTGGGTTATGGTCTAGTGTAGCATCCAGCACGGTGGCTGTGTAAGGAGGATAAGCACCCCCGAGGAATCTCAGCACAT 615

Orcu *CCL8ps* gDNA ...........................R..............Y.........R..................................... 625

Bumo *CCL8ps* gDNA ......................A..C.................A...............A..G.......................-... 624

Syfl *CCL8*  gDNA C..........................................A.....C...............................--....... 624

Leti *CCL8*  gDNA .........G.................................A.....C........................................ 624

Legr *CCL8*  gDNA CA.......G.................................A.....C..A.................................G... 624

Orcu *CCL8ps* WGS GACACAGGTTAGCTACCAGCCCACGGGGTCGACTAGCCGAGAACCAACGCATGTGCAGCTTTGCATGGGCCAACAGGGAAGAGCCTTCTT 705

Orcu *CCL8ps* gDNA ......S...................Y....................Y...................S...................... 715

Bumo *CCL8ps* gDNA ........................C......................Y.......................G....T............. 714

Syfl *CCL8*  gDNA ...T.........................T....G..T.....T.......C.....A..................T............. 714

Leti *CCL8*  gDNA ..................................G..T....T.......G.........................T............. 714

Legr *CCL8*  gDNA ..................................G..T..A.T.................................T............. 714

Orcu *CCL8ps* WGS ACCCAAGTTTCCCGGGATCCACCAACACGGCTGGGTTGTGCGGGCGAGGTTTTACCTCTGGGGTCAGGACTTGGCAGGGTGACCCCTGGC 795

Orcu *CCL8ps* gDNA ......................Y....Y.............R.......Y....Y................................... 805

Bumo *CCL8ps* gDNA ....T......................T............T...T....C............A..........................T 804

Syfl *CCL8*  gDNA ............................A....A......T...T....C............A........................... 804

Leti *CCL8*  gDNA ............................A...........T...T....C............A.....................T....T 804

Legr *CCL8*  gDNA ............................A...........T...T....C............A.....................T....T 804

Orcu *CCL8ps* WGS CCCTTCCCACCTTCTGCCTCCTTCGCGGGTGCATTTTTCCTGTGTATCCTGTCTCACTTGTTGTGAAATTTCTTTC**AG** ATTCCGTTTCC 884

Orcu *CCL8ps* gDNA ...............S.......Y.....................................---.............. ........... 891

Bumo *CCL8ps* gDNA .T.C.....T.C...C.......T..A..................................---.............. ....T...... 890

Syfl *CCL8*  gDNA ...............C.....A.T..A..................................---.............. ....Y...... 890

Leti *CCL8*  gDNA ...C.......C...C.......T..A..................................---.............. .A...A..... 890

Legr *CCL8*  gDNA ...C.......C...C.......T..A..................................---.............. .A......... 890

Leeu *CCL8*  cDNA .A...A..... 109

Orcu *CCL8ps* WGS ATCCCAGTCACCTGCTGCTTTGGTGCGGTCAGCAGAAAGATTCCCATCCAGAGGCTGGAGGGCTACACGAGAATCACCAGTGCCCAG**CGT** 974

Orcu *CCL8ps* gDNA ................**S**........Y.............................................................**.R.** 981

Bumo *CCL8ps* gDNA .....................C...T.............................................................**...** 980

Syfl *CCL8*  gDNA .........G...............T.........G.......................A................G..........T.. 980

Leti *CCL8*  gDNA .........................T......A..G..................................................TT.. 980

Legr *CCL8*  gDNA ..................CC.....T.........G..................................................TT.. 980

Leeu *CCL8*  cDNA .........................T.........G..................................................TT.. 199

Legr *CCL8*  cDNA ..................CC.....T.........G..................................................TT.. 199

**Intron2**

*fs direct repeat*

|*cccggg gcagctgtgat atgagtgcaccaggcc*🡪------------------------------🡪

Orcu *CCL8ps* WGS CCCCGG**G--**GCAGCTGTGAT **AT**GAGTGCACCAGGCC*CCCGG****G****GCAGCTGTGAT****A****TGAGTGCACCAGGCC*AGCTCTCCCAAACTTCTC 1059

Orcu *CCL8ps* gDNA ...Y...--........... ......R.........---------------------------------.................. 1033

Bumo *CCL8ps* gDNA .......--........... ............A...---------------------------------...A........T..... 1032

Syfl *CCL8*  gDNA Y.....---..Y........ **GT**......G.......---------------------------------...A........T..... 1031

Leti *CCL8*  gDNA T....A---...........---GT..............---------------------------------............T..... 1031

Legr *CCL8*  gDNA T....A---........... GT..............---------------------------------............T..... 1031

Leeu *CCL8*  cDNA T....A---........... 216

Legr *CCL8*  cDNA Y....A---........... 216

Orcu *CCL8ps* WGS TCTGAGAAGCAAGGGAAAGTGACCAGGATTCACAGCCACATGAGCCAGATAAATAGACCATCTAATCCGCAGGGACATTCACCCCACAAA 1149

Orcu *CCL8ps* gDNA ........R..........W...............................................YR..................... 1123

Bumo *CCL8ps* gDNA .........T.........A......A..............................T..........A.......T............. 1122

Syfl *CCL8*  gDNA ..G................A.......................................................GT............. 1121

Leti *CCL8*  gDNA ..G................A........................................................T....A........ 1121

Legr *CCL8*  gDNA ..G................A..................--............................A.......T....A........ 1119

Orcu *CCL8ps* WGS GGAGTCCACAGTCGCCCCAGGCTCCCTTCTAGGGGCTTGGTGAGATGGCTCCAGGTGCTTCAGCCAGGAGCCTGGCCGGTGTCACCTGGG 1239

Orcu *CCL8ps* gDNA .............A............................................................................ 1213

Bumo *CCL8ps* gDNA ........T....A... ..............................T..........................T.A..........A. 1211

Syfl *CCL8*  gDNA .............A....................C....................C.............A. ...........G...... 1210

Leti *CCL8*  gDNA A............A..................................Y......................................... 1211

Legr *CCL8*  gDNA A............A..................................T......................................... 1209

Orcu *CCL8ps* WGS -CAGCAAGAGCAGACCTTCCTCTAGAAGCCACCCTCTGC CTCCCCTCCCTCACTCCTGGACCAGGCCTCTCACCCAAGGAGCAAGGGCT 1327

Orcu *CCL8ps* gDNA -.............M........G............... ....Y................................G...R........ 1301

Bumo *CCL8ps* gDNA -G............A........G............... .......T.............................G............ 1299

Syfl *CCL8*  gDNA AG............G........G.....TG.....C..C..G.........T........................G............ 1300

Leti *CCL8*  gDNA -G............A........G............... .....................................G............ 1299

Legr *CCL8*  gDNA -G............A........G............... ...S.................................G............ 1297

Exon3

Orcu *CCL8ps* WGS GGCTAGGTCTAGGACCCCCTGGGCCACACCCCTGGGCGGACCCC TCAAGAGGCTCACCTGGTTGTCCCCTTTCTCTTGC**AG** CTTCAAG 1415

Orcu *CCL8ps* gDNA .............G..Y........................... ....................Y.........Y...... ....... 1389

Bumo *CCL8ps* gDNA .............GG......................A...... ......T.......A......G............... ......R 1387

Syfl *CCL8*  gDNA .........C.-.g.......................A...... ......T..............GT.............. ....... 1388

Leti *CCL8*  gDNA .............G.......................A......C......T...T..........G............... ....... 1388

Legr *CCL8*  gDNA .............G.......................A......C......T...T..........G............T.. ....... 1386

Leeu *CCL8*  cDNA ....... 223

Legr *CCL8*  cDNA ....... 223

Orcu *CCL8ps* WGS ACTAAGCTGGCCAAGGAGGTATGCGCTGACCCCAGGGAGAAATGGGTCCAGGATTCCATGAAGCTCCTGGACCAAAAGTCCCTAACTCGG 1505

Orcu *CCL8ps* gDNA ..................................R....................................................... 1479

Bumo *CCL8ps* gDNA ................................T...............A...............................T.CC...T.. 1477

Syfl *CCL8*  gDNA ................................................A.A.........................R.....CC....CA 1478

Leti *CCL8*  gDNA ................................................A.........................G.......C.....A. 1478

Legr *CCL8*  gDNA ................................................A.........................G.......C.....A. 1476

Leeu *CCL8*  cDNA ................................................A.........................G.......C.....A. 313

Legr *CCL8*  cDNA ................................................A.........................G.......C.....A. 313

Reverse cDNA primer

Orcu *CCL8ps* WGS AAGCCT**TGA** CCTCCCCATGCACACCTGGACTGAGACTCAGAGTCTGAGGCCAACATTATTTATTTCCCAGCCTTCC 1581

Orcu *CCL8ps* gDNA ......... ................................................................... 1555

Bumo *CCL8ps* gDNA ....M.... ...--------------............................ 1517

Syfl *CCL8*  gDNA ......... .......G.....................................G..................... 1554

Leti *CCL8*  gDNA ...T..... ................................................................... 1554

Legr *CCL8*  gDNA ...T..... ................................................................... 1552

Leeu *CCL8*  cDNA ...T..... .................................. 356 Legr *CCL8*  cDNA ...T..... .................................. 356

PCR fragments of leporids were obtained either with gDNA or cDNA and are aligned with the rabbit WGS fragment orthologous to mammalian *CCL8* genes. CDS regions are highlighted in grey. ‘Orcu *CCL8ps’* represents the consensus of 11 haplotypes of *Oryctolagus cuniculus* specimen of both subspecies (*O.c. cuniculus* and *O.c. algirus*), excluding the one that was identical to the WGS sequence. Nucleotide differences occurring only once (singletons) were ignored for establishing the consensus. The pseudogenizing indels were excluded from consensus building, they are present in some but not all wild specimen of both subspecies. The position of primers used for cDNA amplification is underlined. Occ: *Oryctolagus cuniculus;* Bumo*: Bunolagus monticularis;* Syfl: *Sylvilagus floridanus*; Leti*: Lepus timidus*; Legr: *Lepus granatensis*; Leeu: *Lepus europaeus*.
