# Supplementary material for: Pseudogenization of the MCP-2/CCL8 chemokine gene in European rabbit (genus Oryctolagus), but not in species of Cottontail rabbit (Sylvilagus) and Hare (Lepus)
Source: BMC Genet. 2012 Aug 15;13:72. doi: 10.1186/1471-2156-13-72 (PMC3511233; doi:10.1186/1471-2156-13-72)
Supplement: Additional file 8 — Detailed PCR procedures. [file 1471-2156-13-72-S8.doc]

**Additional information A8**

**Detailed PCR procedures**

**A._Genomic DNA** (by Sandra Afonso):

***CCL8* amplification.**

The pair of primers:
**FwPrCL8e1** (Fwd 5’ AGC ACA CGC AGG GTC TTG CT 3’) and **RvPrCL8e1b** (Rev 5’ TCG ACC CCG TGG GCT GGT AG 3’)
was used to amplify a fragment of *CCL8* Exon1 with approximately 600 bp.

With the combination of the pair:
FwPrCL8e2a (Fwd 5’ GCA TCC AGC ACG GTG GCT GT 3’) and RvPrCL8e2a (Rev 5’ GCC AGC CCT TGC TCC TTG GG 3’)
we amplified a fragment of approximately 700 bp of Exon2.

Finally for the Exon3 we used the combinations of primers:
FwPrCL8e3b (Fwd 5’ GGC TCC AGG TGC TTC AGC CA 3’) and RvPrCL8e3b (Rev 5’ AGT ACC CAG GGA AGG CTG GG 3’)
to generate a fragment with approximately 350 bp.

A touchdown PCR was performed and the thermal profile used was the following: initial denaturation (95 ºC for 15 min); denaturation (95 ºC for 30 s), annealing (65 ºC for 30 s, 1ºC decrease/cycle) and extension (72 ºC for 45 s) for 6 cycles (from 65 to 60, this is the touchdown); denaturation (95 ºC for 30 s), annealing (60 ºC for 30 s) and extension (72 ºC for 45 s) for 30 cycles ; and a final extension (60 ºC for 20 min) for Exon1.

A touchdown PCR was performed and the thermal profile used was the following: initial denaturation (95 ºC for 15 min); denaturation (95 ºC for 30 s), annealing (65 ºC for 30 s, 1ºC decrease/cycle) and extension (72 ºC for 45 s) for 6 cycles (from 65 to 60, this is the touchdown); denaturation (95 ºC for 30 s), annealing (60 ºC for 30 s) and extension (72 ºC for 45 s) for 30 cycles ; and a final extension (60 ºC for 20 min) for Exon1

For Exon2 and Exon3 a touchdown PCR was performed and the thermal profile used was the following: initial denaturation (95 ºC for 15 min); denaturation (95 ºC for 30 s), annealing (66 ºC for 30 s, 1ºC decrease/cycle) and extension (72 ºC for 45 s) for 5 cycles (from 66 to 62, this is the touchdown); denaturation (95 ºC for 30 s), annealing (62 ºC for 30 s) and extension (72 ºC for 45 s) for 35 cycles; and a final extension (60 ºC for 20 min).

***CCL13*** amplification was only done for exon1 to check for lack of QP motif (Glutamine-Proline) at the 3’ end of the exon.

The combination of primers:
**F1CL13e**(Fwd 5’ TTG GCT CTC CCG TGG CAG CA 3’) and **R1CL13e1** (Rev 5’ GGC CAG CAC TAT GGC GCA GT 3’)
was used to produced a fragment about 500 bp with rabbit gDNA. A touchdown PCR was performed and the thermal profile used was the following: initial denaturation (95 ºC for 15 min); denaturation (95 ºC for 30 s), annealing (66 ºC for 30 s, 1ºC decrease/cycle) and extension (72 ºC for 45 s) for 5 cycles (from 66 to 62, this is the touchdown); denaturation (95 ºC for 30 s), annealing (62 ºC for 30 s) and extension (72 ºC for 45 s) for 30 cycles; and a final extension (60 ºC for 20 min).

To amplify samples from *Sylvilagus*, *Lepus* and *Oryctolagus* we used the combination of primers:
F9CL13e1 (Fwd 5’ AGG CAG CAA GCA TGG GAG CG 3’) and R9CL13e1 (Rev 5’ GGG CCC TTT GGC TTA GAA GGC G 3’),
resulting a product with about 500 bp.

For the rabbit samples we used the following program: touchdown PCR was performed and the thermal profile used was the following: initial denaturation (95 ºC for 15 min); denaturation (95 ºC for 30 s), annealing (64 ºC for 30 s, 1ºC decrease/cycle) and extension (72 ºC for 45 s) for 5 cycles(from 64 to 60, this is the touchdown); denaturation (95 ºC for 30 s), annealing (60 ºC for 30 s) and extension (72 ºC for 45 s) for 30 cycles; and a final extension (60 ºC for 20 min).

For *Sylvilagus* and *Lepus*: touchdown PCR was performed and the thermal profile used was the following: initial denaturation (95 ºC for 15 min); denaturation (95 ºC for 30 s), annealing (60 ºC for 30 s, 1ºC decrease/cycle) and extension (72 ºC for 45 s) for 6 cycles (from 60 to 55, this is the touchdown); denaturation (95 ºC for 30 s), annealing (55 ºC for 30 s) and extension (72 ºC for 45 s) for 35 cycles; and a final extension (60 ºC for 20 min).

All PCRs were performed using Taq PCR Master Mix by Qiagen and the primers were at 10 pmol.

# PCR cleaning prior to sequencing reaction was performed with Exonuclease I and FastAP™ Thermosensitive Alkaline Phosphatase from Fermentas, Thermo Fisher.

# B._cDNA (by Ana Matos)

**RNA samples, PCR amplification and sequencing:**

Two liver samples from each of the hare species (genus *Lepus*) used in this study, Iberian hare (*Lepus granatensis* - Legr2, Legr6) and European brown hare (*Lepus europaeus* - Leeu1, Leeu2) were used to extract total RNA. Hare samples were supplied by CIBIO, Vairão, Portugal.

Total RNA was prepared using the guanidinium thiocyanate-phenol-chloroform extraction method (TRIzol) according to manufacturer’s instructions (Molecular Research Center, Inc., Cincinnati, OH, USA). Next, first strand cDNA was prepared from 5 µg of RNA and synthesized using oligo (dT) primers (Krug and Berger, 1987).

**CCL8cDNA**

*CCL8*cDNA was PCR-amplified using the set of primers
*CCL8*_F (5’-CTCCAAGCTCGGCTCCTTG-3’) and *CCL8*_R (5’- ACTCTGAGTCTCAGTCCAGGTG-3’).
Cycling parameters consisted of an initial denaturation at 95ºC for 3 min followed by 40 cycles of 95ºC for 45 s, 57ºC for 45 s and 72ºC for 1 min, and a final extension step of 72ºC for 10min. A final PCR product of approximately 300 bp was obtained.

For *CCL7*cDNA two sets of primers were used:
*CCL7*_F1 (5’-TGCTGCTCACAGTGGCTGCC-3’) and *CCL7*_R1 (5’-TCCTCCCCCATGCCAGTGCA-3’);
*CCL7*_F2 (5’-GGCCAGCACAGGAACCTGCA-3’) and CCL7_R2 (5’-TGCAGGCAAGACTGCTCAAGGC-3’).

PCR thermal profile consisted of an initial denaturation at 95ºC for 3 min followed by 40 cycles of 95ºC for 45 s, 63ºC for 30 s and 72ºC for 45 s, and a final extension step of 72ºC for 10min. For the first set of primers the PCR reaction generated, approximately, a 480 bp product. With the second set of primers, a final PCR product of approximately 370 bp was obtained. All PCR products were visualized on 1% agarose gels staining with ethidium bromide.

Sequencing was performed with an ABI PRISM 3130 Genetic Analyser (PE Applied Biosystems), following the ABI PRISM BigDye Terminator Cycle sequencing protocol. PCR products were sequenced in both directions.
