# Supplementary material for: Pseudogenization of the MCP-2/CCL8 chemokine gene in European rabbit (genus Oryctolagus), but not in species of Cottontail rabbit (Sylvilagus) and Hare (Lepus)
Source: BMC Genet. 2012 Aug 15;13:72. doi: 10.1186/1471-2156-13-72 (PMC3511233; doi:10.1186/1471-2156-13-72)
Supplement: Additional file 9 — Genbank Accessions and Links of MCP-Eotaxin mRNA sequences of Placental Mammals used or consulted. [file 1471-2156-13-72-S9.doc]

**Additional file A9**

**Genbank Accessions and Links of MCP‑Eotaxin mRNA sequences of Placental Mammals used or consulted**

| Taxon name | Description | GenBank ref. Accession |
| --- | --- | --- |
| *CCL2aime* | PRED: Ailuropoda melanoleuca c-C motif chemokine 2-like (LOC100474348), mRNA | [XM_002912354.1](http://www.ncbi.nlm.nih.gov/nucleotide/301753167?report=genbank&log$=nucltop&blast_rank=6&RID=HJDNH2WK01N) |
| *CCL2bota* | Bos taurus chemokine (C-C motif) ligand 2 (CCL2), mRNA | [NM_174006.2](http://www.ncbi.nlm.nih.gov/nucleotide/31343056?report=genbank&log$=nucltop&blast_rank=32&RID=HJDNH2WK01N) |
| *CCL2cafa* | Canis lupus familiaris chemokine (C-C motif) ligand 2 (CCL2), mRNA | [NM_001003297.1](http://www.ncbi.nlm.nih.gov/nucleotide/50979119?report=genbank&log$=nucltop&blast_rank=7&RID=HJDNH2WK01N) |
| *CCL2caja* | PRED: Callithrix jacchus C-C motif chemokine 2-like (LOC100387612), mRNA | [XM_002748333.1](http://www.ncbi.nlm.nih.gov/nucleotide/296202023?report=genbank&log$=nucltop&blast_rank=19&RID=HJDNH2WK01N) |
| *CCL2eqca* | Equus caballus chemokine (C-C motif) ligand 2 (CCL2), mRNA | [NM_001081931.1](http://www.ncbi.nlm.nih.gov/nucleotide/126352515?report=genbank&log$=nucltop&blast_rank=15&RID=HJDNH2WK01N) |
| *CCL2hosa* | Homo sapiens chemokine (C-C motif) ligand 2 (CCL2), mRNA | [NM_002982.3](http://www.ncbi.nlm.nih.gov/nucleotide/56119169?report=genbank&log$=nucltop&blast_rank=27&RID=HJDNH2WK01N) |
| *CCL2mamu* | Macaca mulatta chemokine (C-C motif) ligand 2 (CCL2), mRNA | [NM_001032821.1](http://www.ncbi.nlm.nih.gov/nucleotide/74136202?report=genbank&log$=nucltop&blast_rank=23&RID=HJDNH2WK01N) |
| *CCL2mumu* | Mus musculus chemokine (C-C motif) ligand 2 (CCL2), mRNA | [NM_011333.3](http://www.ncbi.nlm.nih.gov/nucleotide/141803162?report=genbank&log$=nucltop&blast_rank=2&RID=HJDNH2WK01N) |
| *CCL2orcu* | Oryctolagus cuniculus chemokine (C-C motif) ligand 2 (CCL2), mRNA | [NM_001082294.1](http://www.ncbi.nlm.nih.gov/nucleotide/126723025?report=genbank&log$=nucltop&blast_rank=3&RID=HJDNH2WK01N) |
| *CCL2patr -* | PRED: Pan troglodytes chemokine (C-C motif) ligand 2, variant 2 (CCL2), mRNA | [XM_001174551.2](http://www.ncbi.nlm.nih.gov/nucleotide/332848039?report=genbank&log$=nucltop&blast_rank=8&RID=HJDNH2WK01N) |
| *CCL2poab -* | Pongo abelii chemokine (C-C motif) ligand 2 (CCL2), mRNA | [NM_001132340.1](http://www.ncbi.nlm.nih.gov/nucleotide/197100249?report=genbank&log$=nucltop&blast_rank=14&RID=HJDNH2WK01N) |
| *CCL2rano* | Rattus norvegicus chemokine (C-C motif) ligand 2 (CCL2), mRNA | [NM_031530.1](http://www.ncbi.nlm.nih.gov/nucleotide/13928713?report=genbank&log$=nucltop&blast_rank=1&RID=HJDNH2WK01N) |
| *CCL2susc* | Sus scrofa chemokine (C-C motif) ligand 2 (CCL2), mRNA | [NM_214214.1](http://www.ncbi.nlm.nih.gov/nucleotide/47523511?report=genbank&log$=nucltop&blast_rank=16&RID=HJDNH2WK01N) |
| *CCL7cafa* | Canis lupus familiaris chemokine (C-C motif) ligand 7 (CCL7), mRNA | [NM_001010960.1](http://www.ncbi.nlm.nih.gov/nucleotide/58219497?report=genbank&log$=nucltop&blast_rank=24&RID=HJDNH2WK01N) |
| *CCL7eqca* | PRED: Equus caballus c-C motif chemokine 7-like (LOC100071714), mRNA | XM_001501551 |
| *CCL7hosa* | Homo sapiens chemokine (C-C motif) ligand 7 (CCL7), mRNA | [NM_006273.2](http://www.ncbi.nlm.nih.gov/nucleotide/13435401?report=genbank&log$=nucltop&blast_rank=17&RID=HJDNH2WK01N) |
| *CCL7mumu* | Mus musculus chemokine (C-C motif) ligand 7 (CCL7), mRNA | [NM_013654.3](http://www.ncbi.nlm.nih.gov/nucleotide/226958664?report=genbank&log$=nucltop&blast_rank=42&RID=HJDNH2WK01N) |
| *CCL7patr -* | PRED: Pan troglodytes chemokine (C-C motif) ligand 7 (CCL7), mRNA | [XM_511407.3](http://www.ncbi.nlm.nih.gov/nucleotide/332848038?report=genbank&log$=nucltop&blast_rank=9&RID=HJDNH2WK01N) |
| *CCL7poab -* | PRED: Pongo abelii c-C motif chemokine 7-like (LOC100453525), mRNA | [XM_002827249.1](http://www.ncbi.nlm.nih.gov/nucleotide/297700516?report=genbank&log$=nucltop&blast_rank=11&RID=HJDNH2WK01N) |
| *CCL7rano* | Rattus norvegicus chemokine (C-C motif) ligand 7 (CCL7), mRNA | [NM_001007612.1](http://www.ncbi.nlm.nih.gov/nucleotide/56090536?report=genbank&log$=nucltop&blast_rank=39&RID=HJDNH2WK01N) |
| *CCL8aime* | PRED: Ailuropoda melanoleuca c-C motif chemokine 8-like (LOC100474856), mRNA | [XM_002912356.1](http://www.ncbi.nlm.nih.gov/nucleotide/301753171?report=genbank&log$=nucltop&blast_rank=10&RID=HJDNH2WK01N) |
| *CCL8bota1* | Bos taurus chemokine (C-C motif) ligand 8 (CCL8), mRNA | NM_174007 |
| *CCL8bota2-* | PRED: Bos taurus C-C motif chemokine 8-like (CCL8), mRNA | [XM_001255312.4](http://www.ncbi.nlm.nih.gov/nucleotide/358417215?report=genbank&log$=nucltop&blast_rank=56&RID=HJDNH2WK01N) |
| *CCL8cafa* | Canis lupus familiaris chemokine (C-C motif) ligand 8 (CCL8), mRNA | [NM_001005255.1](http://www.ncbi.nlm.nih.gov/nucleotide/52546719?report=genbank&log$=nucltop&blast_rank=21&RID=HJDNH2WK01N) |
| *CCL8eqca* | Equus caballus chemokine (C-C motif) ligand 8 (CCL8), mRNA | [NM_001081864.1](http://www.ncbi.nlm.nih.gov/nucleotide/126352331?report=genbank&log$=nucltop&blast_rank=13&RID=HJDNH2WK01N) |
| *CCL8hosa* | Homo sapiens chemokine (C-C motif) ligand 8 (CCL8), mRNA | [NM_005623.2](http://www.ncbi.nlm.nih.gov/nucleotide/22538815?report=genbank&log$=nucltop&blast_rank=20&RID=HJDNH2WK01N) |
| *CCL8mamu* | Macaca mulatta chemokine (C-C motif) ligand 8 (CCL8), mRNA | [NM_001032851.1](http://www.ncbi.nlm.nih.gov/nucleotide/74136262?report=genbank&log$=nucltop&blast_rank=22&RID=HJDNH2WK01N) |
| *CCL8mumu* | Mus musculus chemokine (C-C motif) ligand 8 (CCL8), mRNA | [NM_021443.3](http://www.ncbi.nlm.nih.gov/nucleotide/255708468?report=genbank&log$=nucltop&blast_rank=41&RID=HJDNH2WK01N) |
| *CCL8patr-* | PRED: Pan troglodytes chemokine (C-C motif) ligand 8 (CCL8), mRNA | [XM_523600.3](http://www.ncbi.nlm.nih.gov/nucleotide/332848037?report=genbank&log$=nucltop&blast_rank=18&RID=HJDNH2WK01N) |
| *CCL8susc* | Sus scrofa chemokine (C-C motif) ligand 8 (CCL8), mRNA | [NM_001164515.1](http://www.ncbi.nlm.nih.gov/nucleotide/256838114?report=genbank&log$=nucltop&blast_rank=12&RID=HJDNH2WK01N) |
| *CCL11bota* | Bos taurus chemokine (C-C motif) ligand 11 (CCL11), mRNA | [NM_205773.2](http://www.ncbi.nlm.nih.gov/nucleotide/93205065?report=genbank&log$=nucltop&blast_rank=38&RID=HJDNH2WK01N) |
| *CCL11eqca* | Equus caballus chemokine (C-C motif) ligand 11 (CCL11), mRNA | [NM_001081871.1](http://www.ncbi.nlm.nih.gov/nucleotide/126352625?report=genbank&log$=nucltop&blast_rank=47&RID=HJDNH2WK01N) |
| *CCL11hosa* | Homo sapiens chemokine (C-C motif) ligand 11 (CCL11), mRNA | [NM_002986.2](http://www.ncbi.nlm.nih.gov/nucleotide/22538399?report=genbank&log$=nucltop&blast_rank=37&RID=HJDNH2WK01N) |
| *CCL11mamu* | Macaca mulatta chemokine (C-C motif) ligand 11 (CCL11), mRNA | [NM_001032874.1](http://www.ncbi.nlm.nih.gov/nucleotide/74136304?report=genbank&log$=nucltop&blast_rank=46&RID=HJDNH2WK01N) |
| *CCL11mumu* | Mus musculus chemokine (C-C motif) ligand 11 (CCL11), mRNA | [NM_011330.3](http://www.ncbi.nlm.nih.gov/nucleotide/157909793?report=genbank&log$=nucltop&blast_rank=43&RID=HJDNH2WK01N) |
| *CCL11orcu-* | PRED: Oryctolagus cuniculus small inducible cytokine A11, transcript variant 1, mRNA | [XM_002719226.1](http://www.ncbi.nlm.nih.gov/nucleotide/291405568?report=genbank&log$=nucltop&blast_rank=48&RID=HJDNH2WK01N) |
| *CCL11v2orcu* | PRED: Oryctolagus cuniculus small inducible cytokine A11, transcript variant 2, mRNA | [XM_002719227.1](http://www.ncbi.nlm.nih.gov/nucleotide/291405570?report=genbank&log$=nucltop&blast_rank=53&RID=HJDNH2WK01N) |
| *CCL11patr-* | PRED: Pan troglodytes chemokine (C-C motif) ligand 11 (CCL11), mRNA | [XM_523599.2](http://www.ncbi.nlm.nih.gov/nucleotide/114668175?report=genbank&log$=nucltop&blast_rank=36&RID=HJDNH2WK01N) |
| *CCL11poab-* | PRED: Pongo abelii eotaxin-like (LOC100453160), mRNA | [XM_002827248.1](http://www.ncbi.nlm.nih.gov/nucleotide/297700518?report=genbank&log$=nucltop&blast_rank=35&RID=HJDNH2WK01N) |
| *CCL11rano* | Rattus norvegicus chemokine (C-C motif) ligand 11 (CCL11), mRNA | [NM_019205.1](http://www.ncbi.nlm.nih.gov/nucleotide/11276088?report=genbank&log$=nucltop&blast_rank=45&RID=HJDNH2WK01N) |
| *CCL11susc* | PRED: Sus scrofa CCL11 (LOC100038010), mRNA | [XM_003131725.1](http://www.ncbi.nlm.nih.gov/nucleotide/311267859?report=genbank&log$=nucltop&blast_rank=34&RID=HJDNH2WK01N) |
| *CCL12mumu* | PRED: Mus musculus c-C motif chemokine 12-like (LOC100504977), mRNA | [XM_003085794.1](http://www.ncbi.nlm.nih.gov/nucleotide/309262600?report=genbank&log$=nucltop&blast_rank=4&RID=HJDNH2WK01N) |
| *CCL12rano* | Rattus norvegicus chemokine (C-C motif) ligand 12 (CCL12), mRNA | [NM_001105822.1](http://www.ncbi.nlm.nih.gov/nucleotide/157786667?report=genbank&log$=nucltop&blast_rank=54&RID=HJDNH2WK01N) |
| *CCL13aime* | PRED: Ailuropoda melanoleuca c-C motif chemokine 13-like (LOC100475112), mRNA | [XM_002912357.1](http://www.ncbi.nlm.nih.gov/nucleotide/301753173?report=genbank&log$=nucltop&blast_rank=28&RID=HJDNH2WK01N) |
| *CCL13cafa* | Canis lupus familiaris chemokine (C-C motif) ligand 13 (CCL13), mRNA | [NM_001003966.1](http://www.ncbi.nlm.nih.gov/nucleotide/51556232?report=genbank&log$=nucltop&blast_rank=33&RID=HJDNH2WK01N) |
| *CCL13eqca* | Equus caballus chemokine (C-C motif) ligand 13 (CCL13), mRNA | [NM_001163887.1](http://www.ncbi.nlm.nih.gov/nucleotide/255522910?report=genbank&log$=nucltop&blast_rank=31&RID=HJDNH2WK01N) |
| *CCL13hosa* | Homo sapiens chemokine (C-C motif) ligand 13 (CCL13), mRNA | [NM_005408.2](http://www.ncbi.nlm.nih.gov/nucleotide/22538799?report=genbank&log$=nucltop&blast_rank=44&RID=HJDNH2WK01N) |
| *CCL13mamu* | PRED: Macaca mulatta chemokine (C-C motif) ligand 13 (CCL13), mRNA | [XM_001113462.2](http://www.ncbi.nlm.nih.gov/nucleotide/297272368?report=genbank&log$=nucltop&blast_rank=29&RID=HJDNH2WK01N) |
| *CCL13poab-* | PRED: Pongo abelii c-C motif chemokine 13-like (LOC100453895), mRNA | [XM_002827250.1](http://www.ncbi.nlm.nih.gov/nucleotide/297700520?report=genbank&log$=nucltop&blast_rank=25&RID=HJDNH2WK01N) |

Source: Genbank reference RNA sequences (Refseq_rna)

Taxon names marked with ‘-‘ are not included for phylogenetic construction shown in Figure 3.
